# Mouse Paternal RNAs Initiate a Pattern of Metabolic Disorders in a Line-Dependent Manner

**DOI:** 10.3389/fgene.2022.839841

**Published:** 2022-03-28

**Authors:** Guzide Satir-Basaran, Leila Kianmehr, Ecmel Mehmetbeyoglu, Kezban Korkmaz Bayram, Mehmet Memis, Zeynep Yilmaz, Esra Tufan, Serpil Taheri, Fahrettin Kelestimur, Minoo Rassoulzadegan

**Affiliations:** ^1^ Betul Ziya Eren Genome and Stem Cell Center, Erciyes University, Kayseri, Turkey; ^2^ Department of Biochemistry, Faculty of Pharmacy, Erciyes University, Kayseri, Turkey; ^3^ Departement of Animal Sciences and Marine Biology, Faculty of Life Sciences and Biotechnology, Shahid Beheshti University, Tehran, Iran; ^4^ Department of Cancer and Genetics, Cardiff University, Cardiff, United Kingdom; ^5^ Department of Medical Genetics, Medical Faculty, Yıldırım Beyazıt University, 06800 Ankara, Turkey; ^6^ Department of Medical Biology, Erciyes University Medical School, Kayseri, Turkey; ^7^ Department of Endocrinology, Yeditepe University Medical School, Istanbul, Turkey; ^8^ INSERM-CNRS, Université de Nice, Nice, France

**Keywords:** sperm, diet, mouse, epigenetic, Dnmt2, non-coding RNAs, miRNAs, DNA/RNA hybrids

## Abstract

A wide range of diseases result from environmental effects, and the levels of many native transcripts are altered. The alteration of non-coding RNAs (ncRNAs) and transmission of the variation to the next generation is increasingly recognized as a marker of disease. However, the determining signals and mechanisms of RNA-induced heritability remain unclear. We performed functional tests with four different genotypes of mice maintained on a high-fat diet to trace the transfer of the obesity/diabetes phenotype to the next generation in order to detect common signals. Two founders of four mouse lines (*B6/D2* hybrid and *Dnmt2* ^*−/−C57BL/6*^) resist and do not change their phenotype while their sperm RNAs after microinjection into fertilized mouse eggs transfer the newly acquired phenotypes in a susceptible inbred line (*C57BL/6* or *Balb/c*). Unexpectedly, in the same line of experiments, sperm RNA from animals raised on a normal diet when mixed with the sperm RNA from animals raised on a diet high in fat or synthetic miR-19b (inducer of obesity) affects or prevents the development of obesity and diabetes. However, it remains unclear what happens to ncRNA signaling under diet. With a comprehensive new analysis of the transcripts maintained as an RNA/DNA hybrid in sperm, we suggest that a fraction of the RNAs are stably attached to the genome. Thus, we propose that changes in the dynamics of ncRNA retention on DNA by factors such as transcriptional variations or lack of adequate methylation could serve as molecular markers to trace these epigenetics events.

## Introduction

Parents’ diet can be a concern for future generations, ultimately contributing to the development of diseases (Buettner et al., 2007; Hur et al., 2017). In mice raised on a high-fat diet (HFD), the offspring may develop obesity/type 2 diabetes (OB/T2D) like phenotypes (West and York, 1998; Pomp et al., 2008; Massiera et al., 2010; Sims et al., 2013; EASD, 2015). Inbred laboratory mouse models with known genetics raised under defined conditions allow investigation of this problem. However, unlike other lines (Winzell and Ahrén, 2004), some laboratory mice are resistant to the development of OB/T2D like phenotypes when fed on HFD (Montgomery et al., 2013). To address a common mechanism that determines susceptibility to HFD, we analyzed the phenotypes of four lineages of laboratory mice and their two successive generations F1 and F2. We studied the influence of genotypes on the response to HFD and its transgenerational maintenance. We followed four lines of mice, two inbred, *C57BL/6* (Rossmeisl et al., 2003) (often used) and *Balb/c*, one hybrid *B6/D2* (F1 cross of *C57BL/6* and *DBA2*), and a mutant *Dnmt2* ^*−/−C57BL/6*^ (missing of functional RNA methyltransferase see for review (Lyko, 2018)) maintaining all mice under the same housing and diet conditions. Individuals with apparently similar dietary intake varied in weight and expression of phenotypes. Our results indicated that despite varying levels of obesity, the lineages differ markedly in the transmission of regulatory factors of obesity, glucose homeostasis, and insulin sensitivity.

Several studies now show changes in diet induce RNA variations also in the sperm cells (see recent review (Zhang et al., 2019)). The RNA content of sperm is complex (Kawano et al., 2012; Burl et al., 2018) and varies considerably with diet (Chen et al., 2016a; Chen et al., 2016b; Klastrup et al., 2019; Nätt et al., 2019). A comparison based on RNA-sequencing of sperm from founder males raised on a normal diet (ND) versus HFD confirmed variations in several transcripts, including small noncoding RNAs (sncRNAs) (Klastrup et al., 2019; Zhang et al., 2019). Previously, it had been observed that the total RNAs of spermatozoa obtained from males (founders) raised on HFD after microinjection into naive fertilized eggs induced the same pathological variation as that observed in the founders and was transmitted to offspring (Grandjean et al., 2015; Chen et al., 2016a).

In addition, Chen et al. (Chen et al., 2016a) identified purified RNAs from aqueous phase of total sperm enriched in transfer RNA fragments (tRNA) derived from 5′ tRNA as inducers of phenotypic changes. HFD-mediated phenotypes (HFD-MPs) are transferable simply by microinjection of RNAs (total or fractionated) from spermatozoa into naive fertilized mouse eggs. On the other hand, our laboratory studied the variations induced by food on the miRNAs of spermatozoa, one in particular (miR-19b) with increasing levels under HFD (Grandjean et al., 2015). In fact, microinjection of synthetic miR-19b into naive fertilized mouse eggs can transfer disease to offspring, thus showing a role of miRNA in the establishment of the HFD-MPs (Grandjean et al., 2015). In contrast, microinjection of synthetic tsRNAs is not sufficient to induce disease (Chen et al., 2016a; Zhang et al., 2018), indicating that the tsRNA fraction of sperm contains more information such as RNA modification (see below).

In addition to changes in the level of ncRNAs (miRNAs, piRNAs, cRNAs, and lncRNAs etc.), modification of RNAs such as C methylation is also involved in the induction and transfer of disease heritability. Initially, the Bestor’s group (Goll et al., 2006) demonstrated that methylation of tRNAs at position 38 in tRNA^Asp^GUC occurs by the Dnmt2 protein. Subsequently, a group of RNA modifying enzymes has also been reported [see recent review for more information (Jeltsch et al., 2017)]. In addition, we have previously reported a role of the Dnmt2 protein in the transmission of epigenetic traits from one generation to another (Kiani et al., 2013). In fact, homozygous *Dnmt2* ^*−/−*^ mice fail to transmit the epigenetic variation in coat color initiated by alteration of *c-kit* transcripts (Kiani et al., 2013). In preparing this report, colleagues reported that the absence of the methyltransferase, Dnmt2, also abolished the transmission of obesity and diabetes to offspring (Zhang et al., 2018). According to this latest report, in the absence of the Dnmt2 protein, the 30–40 nt RNAs in the spermatozoa of obese animals do not show the elevation of the modifications (m^5^C, m^2^G) normally induced by HFD (Zhang et al., 2018). Thus, in addition to the changes in the proportions of transcripts in sperm, RNA modifications are also important for conveying hereditary information. However, it is not yet clear why RNA levels (tsRNAs fragments, miR-19b or other noncoding RNAs) vary depending on HFD and the underlying mechanism. Alterations in tRNA fragments (tRFs) have already been reported in several circumstances (stress, drugs, and food uptake), yet it is difficult to predict the specificity of tRFs in HFD-MPs, while miR-19b is a unique sequence in the mouse genome with multiple predicted mRNA targets in several normal tissues and the development of the disease (Li and YaoMicroRNAs, 2012).

On the other hand, we have shown that the sperm ncRNAs in the *Dnmt2* ^*−/−C57BL/6*^ inbred background are found at varying levels from male to male regardless of diet. These varying levels of ncRNAs in *Dnmt2* ^*−/−C57BL/6*^ mice are suspected to be produced by uncertain changes in RNA stability in the absence of Dnmt2. In addition to *Dnmt2* ^*−/−C57BL/6*^, we reveal here another line *B6/D2* hybrid mice (wild type) resistant to HFD. Organisms with hybrid genetics are known for their variation in the levels of transcripts (Botet and Keurentjes, 2020) and hybrid vigor commonly used in transgenesis (Clark et al., 2020). Thus, data from these two HFD resistant lines suggest a tempting explanation that predisposition to ncRNA variability, either by transcripts proportions or by stability, may allow unlimited possibilities of unsuccessful heritability.

Finally, we recently developed a direct protocol to detect DNA-associated RNA molecules in sperm, which reveals stable DNA/RNA hybrid regions in the genome as an R-loop structure. We performed sperm RNA preparations followed by RNA-sequencing combined with a bioinformatic analysis comparison between two fractions of RNA (attached to DNA versus free RNA molecules) in the same male (Kianmehr et al., 2019; Rassoulzadegan et al., 2021). Our results suggest changes in sperm RNA ratios between free and DNA-associated RNA fractions in the *Dnmt2* ^*−/−C57BL/6*^ genome compared to wild type. Hybrid DNA/RNA annotation in spermatozoa may represent a signal to be retained from life experiences.

Together, these results highlight the combinatorial effects of ncRNAs in the field of RNA-mediated epigenetic hereditary variation and its consequence on cross-generations.

## Results

### Health and genealogical monitoring of four founder lines of mice (males) raised in a diet rich in fat

To answer the question of susceptibility to HFD (21% butter) among different genotypes and its transgenerational maintenance, we maintained two inbred lines, *C57BL/6* and *Balb/c with B6/D2* hybrids (F1 cross between the *C57BL/6* and *DBA2* lines) and the *Dnmt2* ^*−/−C57BL/6*^ mutants. To minimize the effect from the environment, all experiments were conducted using animals born during the same week under the same housing and feeding conditions. From founder males (F0) raised in HFD, we derived two generations by sexual mating (F1 and F2). All mice other than F0 males were kept on normal diet (ND) and were followed by monitoring their body weight and health for up to 20 weeks. During this time, lineage-dependent differences of mice in test results for weight gain, glucose and insulin tolerance were observed. A summary of the experiments with four lines (*C57BL/6*, *Balb/c, B6/D2* and including *Dnmt2* ^*−/−C57BL/6*^) is presented in the Supplementary Figures: founder (F0) Supplementary Figures S1–S4, F1 generation Supplementary Figures S5–S8, F2 generation Supplementary Figures S9–S12.

Additionally, all glucose tolerance test (GTT) and insuline tolerance test (ITT) plots with area under the curve (AUC) were analyzed before and after normalization to baseline glucose levels (see the guidelines provided in PMID: 34117483). Analyses of AUC for GTTs and ITTs involve first normalizing the data (i.e. subtracting the initial glucose reading at time 0’ from all subsequent readings). This is thought to be important, because a GTT and an ITT the response to a glucose and insulin boost, respectively. The calculated AUC, after normalizing data to baseline glucose levels, is proposed to reveal a much more subtle effect than usually reported without normalization. However, there are conflicting opinions on the application of AUC in chronically treated animals [see references (KC et al., 2018)]. Basically, both methods reveal the differences between animals maintained on HFD and ND. The GTT results are all in agreement but the difference between ND/HFD are more supported with the normalization. The ITT tests while keeping the differences between the two conditions (HFD vs. ND) had however a less pronounced difference with normalization.

**FIGURE 1:**
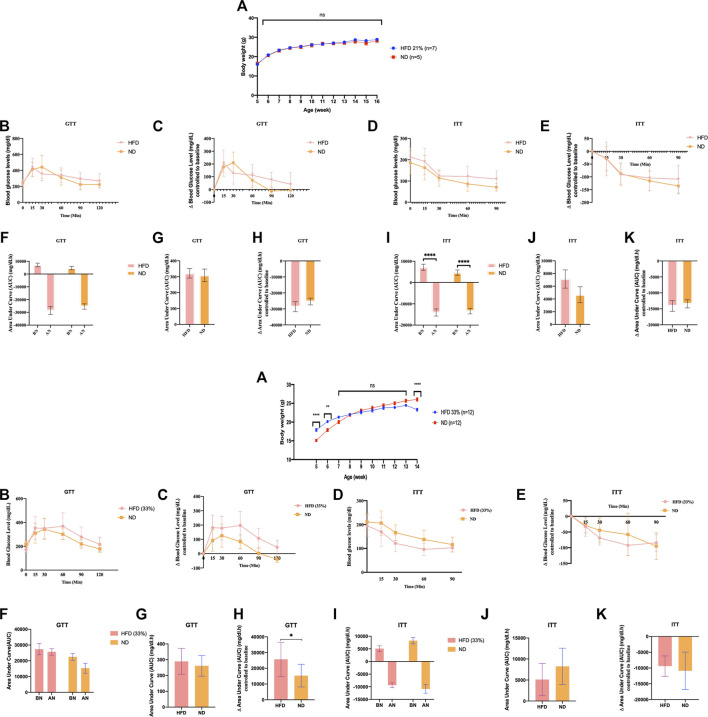
Founder animals *Dnmt2* ^*−/− C57BL/6*^ are resistant to high-fat diet. Thirty-six *Dnmt2* ^*−/−C57BL/6*^ males (*C57Bl/6* background) from 20 litters weaned at 4 weeks old were divided into two groups. One group was maintained on an ND, and the other group was on HFD containing 21% (Figure 1-1) or 33% (Figures 1-2), butter (see Methods) and monitored for body weight **(A)**, fasting blood glucose **(B,C)**, and insulin tolerance test **(D,E)** at 15 weeks. The area under curve (AUC) (Virtue and Vidal-Puig, 2021) **(F–K)** was also calculated after normalization based on the glucose levels of each time point to 0 point (HFD and control ND), before normalization (BN) and after normalization (AN). Statistical analysis was performed by two-tailed, two-way ANOVA, uncorrected Fisher’s LSD. **p < 0.01, ****p < 0.0001 (ND vs. HFD), ns, not significant. All data are plotted as mean ± S.E.M. The raw data are presented in Supplementary Table S1 and individual raw data in Supplementary Table for Figure 1.

Variations in test results (*C57BL/6*) for body weight gain, glucose, and insulin tolerance were noticeable in the F0 generation (Supplementary Figure S4) and are clearly perpetuated in the F2 generation, see Supplementary Figures S8 (F1) and Supplementary Figures S12 (F2) in *C57BL/6* according as previously reported (Grandjean et al., 2015). Glucose and insulin tolerance tests demonstrated prominent pathology in the founder animals and showed larger differences in a lineage-dependent manner in the F1 and F2 generations (Supplementary Figures S5–12). Two founder inbred lines *Balb/c* (Supplementary Figure S1) and *C57BL/6* (Supplementary Figure S4) are sensitive to HFD, and the mice show high fat diet mediated phenotypes (HFD-MPs) and transmit to the F1 and F2 generations. On the other hand, the founders of the *B6/D2* line (Supplementary Figure S2) and particularly *Dnmt2* ^*−/− C57BL/6*^ (Supplementary Figure S3 and Figure 1) are resistant to HFD (no change in weight). The *B6/D2* F2 generation (Supplementary Figure S10) differs from that of the founder due to genetic segregation (visible on coat color). The founder’s *B6/D2* hybrid animals (Supplementary Figure S2) are not affected in their weight and are unresponsive to GTT and ITT challenging, but the next generation develops HFD-MPs (Supplementary Figure S6, F1 and Supplementary Figure S10, F2). These results suggest that the initial signals that program the health of the offspring are perceived differently in the somatic and germ cells. The results on the *Dnmt2* ^*−/−C57BL/6*^ line are also shown in Figure 1 and in Supplementary Figure S3 (F0), Supplementary Figure S7 (F1), and Supplementary Figure S9 (F2). *Dnmt2* ^*−/−C57BL/6*^ mutants exhibit a defect in methylation detectable with highly expressed tRNA molecules, which disrupts their metabolism (Lyko, 2018). We have previously reported a role of methylation of RNA other than tRNA in the transmission of hereditary epigenetic signals (Kiani et al., 2013). Our results here confirm a role of the Dnmt2 protein in the hereditary modification of the HFD-MPs.

A summary of the number of mice used in Supplementary Figures S1–3 can be found in the Supplementary Figures.

### The Dnmt2^−/−C57BL/6^ line did not develop complete metabolic diseases even when fed a diet of up to 33% butter fat

In addition to the above experiments with the *Dnmt2* ^*−/−C57BL/6*^ line, two independent experiments with *Dnmt2* ^*−/−C57BL/6*^ males are shown in Figure 1, one again as above with 21% and one at 33% fat diet. Raw data for each group of mice in Figure 1 can be found in Supplementary Table S1 and in individual raw data of Figure 1.

We examined the ability of *Dnmt2* ^*−/−C57BL/6*^ mutants to gain weight under the same conditions on a 21% butter (HFD) diet as previously described using a larger number of animals, 10 males and eight females; neither sex gained weight (see Figure 1 for males and Supplementary Figure S3 for females). Using a higher concentration of sugar and 33% butter (see Materials and Methods), we tested 12 males in each condition as shown in Figure 1. *Dnmt2* ^*−/−C57BL/6*^ mutant males did not gain weight. The physiology of the *Dnmt2* ^*−/−C57BL/6*^ mutant was different from that of wild-type mice, and even under 33% HFD, the mice did not get weight but are sensitive to glucose and insulin without symptoms. A higher concentration of up to 60% HFD has been reported previously (Zhang et al., 2018), and *Dnmt2* ^*−/−C57BL/6*^ mice at this higher concentration of HFD could eventually become obese but are unable to transmit this phenotype to the next generation.

### Microinjection of total RNAs from spermatozoa of different mouse lines transfer obesity and diabetes in the susceptible Balb/C line

**FIGURE 2:**
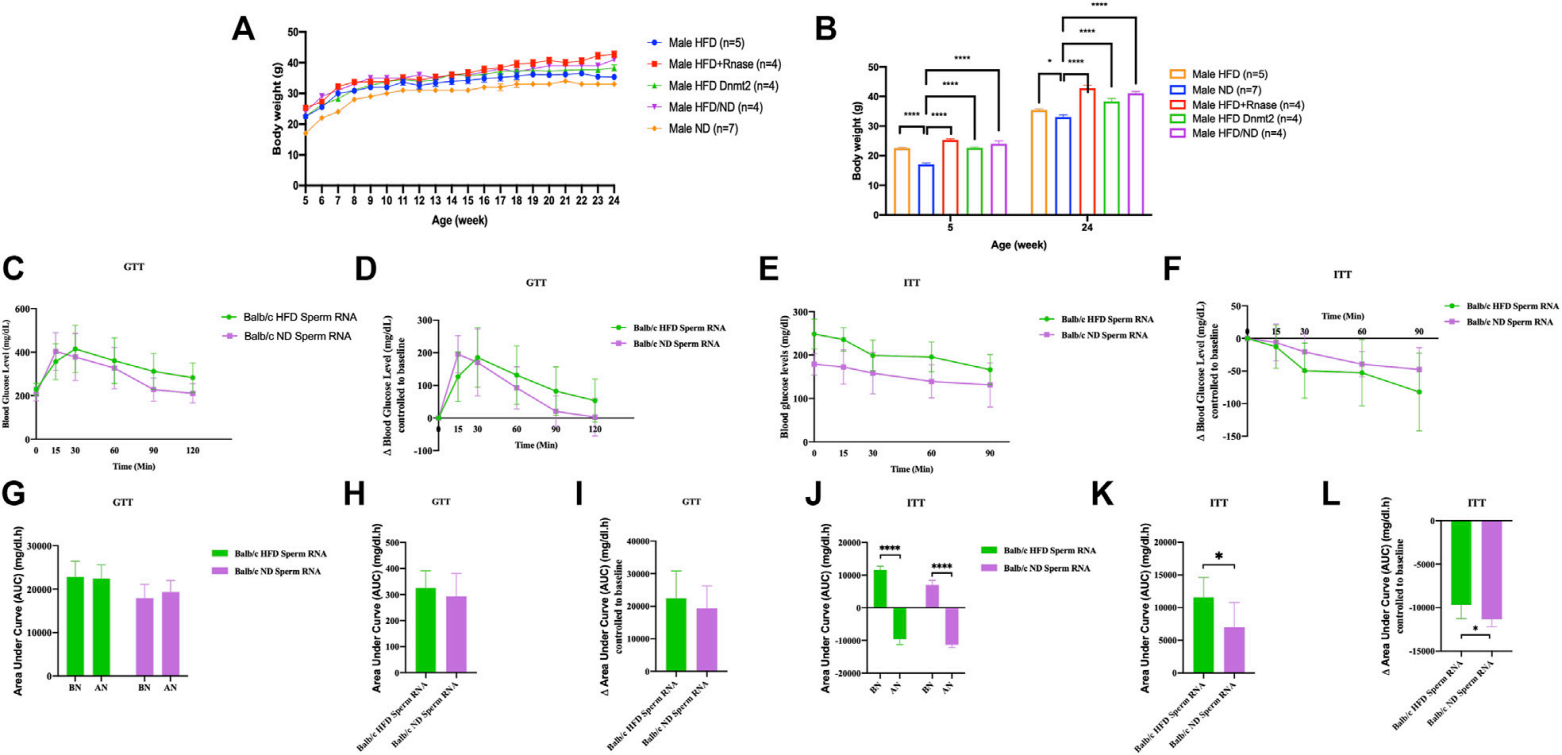
Microinjection of total RNA of sperm from HFD-resistant males perturbs the health of susceptible offspring (Balb/c). Total sperm RNAs prepared from males raised on ND or HFD were microinjected into Balb/c fertilized eggs, as described in the Methods section. Microinjection performed with total sperm RNAs for the three lines, Balb/c, ND, HFD, Mixed ND/HFD and HFD RNA added RNase before microinjection and finally HFD Dnmt2-/-C57BL/6 RNA. n = number of mice in each group. All mice were raised on an ND. Body weights **(A, B)**, fasting blood glucose (GTT) **(C)** and insulin tolerance (ITT) **(D)** at 15 weeks are presented for males. The area under curve (AUC) 30 after normalization were also calculated based on the glucose levels of each time point **(E, F)** (Each group vs control ND). Females are shown in Supplementary Figure S13. Comparison with or without normalization of GTT **(G–I)** and ITT **(J–L)** challenges analysis. Statistical analysis was performed by two-tailed, one-way analysis of variance (ANOVA), uncorrected Fisher’s least significant difference (LSD). * *p* < 0.05, ** *p* < 0.01, *** *p* < 0.001 ****p < 0.0001 (ND versus each group). Ns, not significant. All data are plotted as mean ± s.e.m. All raw data are presented in Supplementary Table S2 and individual raw data in Supplementary Table for Figure 2.

Another generation (G1) was obtained in Figure 2 by microinjecting total sperm RNAs from different founders into fertilized mouse eggs (naïve *Balb/c*). We observed that lineage-dependent phenotypic variations in the disease spectrum (weight, GTT and ITT) were transferable by microinjection of total RNA from spermatozoa of *C57BL/6*, *B6/D2*, *Balb/c* and *Dnmt2* ^*−/−C57BL/6*^ mice in fertilized eggs from naïve *Balb/c* mice. A higher effect is observed on female mice born after microinjection with sRNAs.

Thus, the total spermatic F0 RNAs of HFD-resistant founders, in particular the *Dnmt2* ^*−/−C57BL/6*^ and *B6/D2* hybrids, also contained RNAs that could recapitulate the metabolic phenotypes after microinjection into fertilized eggs of a susceptible line (*Balb/c*). We assume that unmethylated sRNAs from *Dnmt2* ^*−/−C57BL/6*^ are methylated by wild-type Dnmt2 protein present in fertilized *Balb/c* eggs. The dynamism of methylation remains to be assessed. These results again confirm the role of RNA methylation mediated by Dnmt2 protein in the transfer of hereditary acquired epigenetic traits. In addition, these results reveal signals from as yet unexplained sRNAs from the *B6/D2* hybrid line which required further investigation.

In addition, we microinjected RNase enzymes with sRNAs to initially abolish RNA inducing signals. The results of the RNase enzyme microinjection are however contradictory with the expected results. Under these conditions we believe that the RNase treatment removes more than the sRNAs at the time of microinjection into the fertilized eggs. We report this negative result to signal in future experiments that the RNase enzyme should be removed from the aliquot prior to the microinjection step.

**FIGURE 3:**
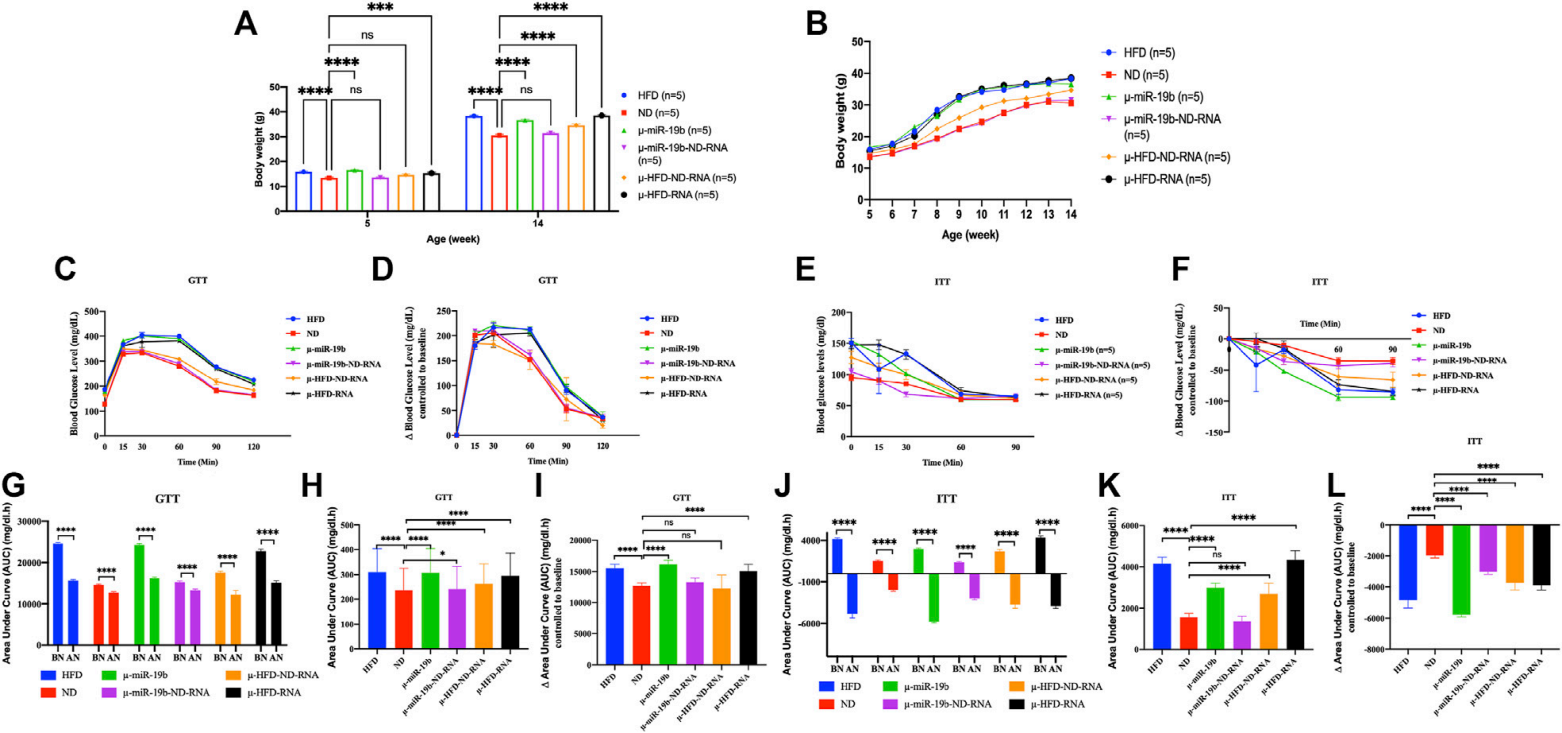
Induction of obesity and diabetes in mice by microinjection of miR-19b-5p or total RNAs of sperm into fertilized mouse eggs. Microinjection of miR-19b-5p (synthetic RNA) into fertilized mouse eggs (one-cell embryos) leads to obesity and diabetes in derived adult mice. Here, we compared the activity of miR-19b-5p RNA alone or in combination with total RNA as carrier from spermatozoa from animals raised on ND. All male animals were fed an ND and monitored for body weight, fasting blood glucose (GTT) and an insulin tolerance test (ITT) at 15 weeks. Total RNA from spermatozoa from males raised on a normal diet (ND) suppresses the activity of miR 19b-5p in the induction of obesity and type 2 diabetic (T2D) phenotype. We also compared the activity of the total RNA of sperm from animals fed with HFD alone (1 g/ml) or in combination with total RNA from sperm (1 g/ml) as carrier from animals raised in ND. Total sperm RNAs from ND animal do not completely suppress the induction of HFD-Mediated Phenotypes (HFD-MPs) of total RNA from sperm from HFD animals but a reduction is observed in the expression of HFD-MPs. n = number of mice in each group. **(A)** Body weight of males born after microinjection of miR-19b-5p in each group at the age of 5 weeks to 14 weeks. Statistical analysis was performed by two-tailed, one-way analysis of variance (ANOVA), uncorrected Fisher’s least significant difference (LSD). ****p < 0.0001 (ND vs. each group); ***p < 0.001, ****p < 0.0001. **(B)** Body weight of males in each group from 6–14 weeks of age. **(C)** Blood glucose during the glucose tolerance test (GTT). Statistical analysis was performed by two-tailed, two-way ANOVA, uncorrected Fisher’s LSD. ****p < 0.0001 (ND vs. HFD); ^####^p < 0.0001 (ND vs. µ-miR-19b); ^★★★^p < −0.001 (ND vs. µ-HFD-ND-RNA), ^xxxx^p < 0.0001(ND vs. µ-HFD-RNA). The area under curve (AUC) (Virtue and Vidal-Puig, 2021) were also calculated after normalization based on the glucose levels of each time point **(D,F)**. **(E)** Relative blood glucose during the insulin tolerance test (ITT). Statistical analysis was performed by two-tailed, two-way ANOVA, uncorrected Fisher’s LSD. ****p < 0.0001 (ND vs. HFD); ^####^p < 0.0001, ^#^p < 0.01 (ND vs. µ-miR-19b); ^★★★★^p < 0.0001, ^★★★^p < 0.0001 (ND vs. µ-HFD-ND-RNA), ^xxxx^ *p* < 0.0001(ND vs. µ-HFD-RNA). ^•^ *p* < 0.01, ^••••^ *p* < 0.0001 (ND vs. µ-miR-19b-ND-RNA). ns, not significant. All data are plotted as mean ± S.E.M. All of the raw data in Figure 3 are presented in Supplementary Table S3 and individual raw data in Supplementary Table for Figure 3.

Finally, co-injection with sperm RNAs from ND could compromise obesity (Figure 2). Previously, we reported that microinjection of total sperm RNA (aqueous phase free RNA fraction) from males raised on HFD into fertilized mouse eggs (one-cell embryos) transmitted HFD-MPs to the offspring. We investigated how to counterbalance the activity of HFD sperm RNAs. To examine this question, we combined founder sperm RNAs raised on HFD with sperm RNAs from animals raised on ND in a 1:1 ratio. As shown in Figures 2, 3, ND sperm RNAs (sRNA-ND) affected the activity of sperm RNA (sRNA-HFD) of animals on HFD. In fact, the animals weighed less but maintained the difference throughout the growing period. However, the activity of HFD RNA is not completely suppressed by sRNA-ND.

### Total RNAs of sperm from males raised on normal food withdraw miR-19b activity in early embryos

In studies reported from our laboratory, we have shown that microinjection of miR-19b-5p is sufficient to recapitulate HFD-mediated phenotypes (HFD-MPs) (Grandjean et al., 2015). Indeed, we have shown that a high number of miR-19b-5p molecules microinjected into fertilized mouse eggs leads to phenotype like HFD-MPs (see Figure 3). After microinjection into fertilized mouse eggs of a high copy number of miRNAs, most (>90%) of them are released from eggs within 24 h (our unpublished results). This high copy number scenario is however difficult to imagine and if to occur would be very rare *in vivo* except under extreme conditions. Under natural conditions this scenario does not occur for a given miRNA of spermatozoa from animals reared in HFD. It is unlikely that a small amount of changes in individual RNAs is immediately responsible for the complete phenotype. On the other hand, several ncRNAs (microRNAs, piRNAs, siRNAs, cRNAs, tRNAs, lncRNAs, etc.) are also increased during HFD; as such, we expect a cumulative role of ncRNAs in the inheritance of diet-induced metabolic phenotypes. To answer the question of the specificity of miR-19b-5p, we used the same amount of miR-19b-5p (see Materials and methods) as that used previously, now in combination with total sperm RNAs (sRNA) as carrier from animals raised in an ND. As shown in Figure 3, the activity of miR-19b-5p was completely suppressed by sRNA-ND in the induction of metabolic disorders. With ND sRNAs, the induction of HFD-MPs pathways by miR-19b-5p is compromised. This indicates that a certain amount of miR-19b-5p is required, and here sRNA-ND carrier mixture (mRNAs, lncRNAs, sncRNAs, piRNAs, cRNAs, etc.) affects the activity of miR-19b.

Under these conditions sRNA-HFD mixed with sRNA-ND always maintains a detectable level of activity. RNA-mediated transfer of phenotype is confirmed by variation in the phenotype expression of weight gain and GTT and ITT tests (with or without normalization). Microinjection of a synthetic miR-19b is completely abolished by sRNA-ND; on the other hand, under the same conditions, sRNA-HFD maintains detectable levels of activity. We predict that synthetic miR-19b-5p is less stable than endogenous sRNA-ND or sRNA-HFD. The stability of RNA molecules would secure their activity.

### miRNA-19 Expression profiles of spermatozoa from male (raised on HFD) compared to controls

**FIGURE 4:**
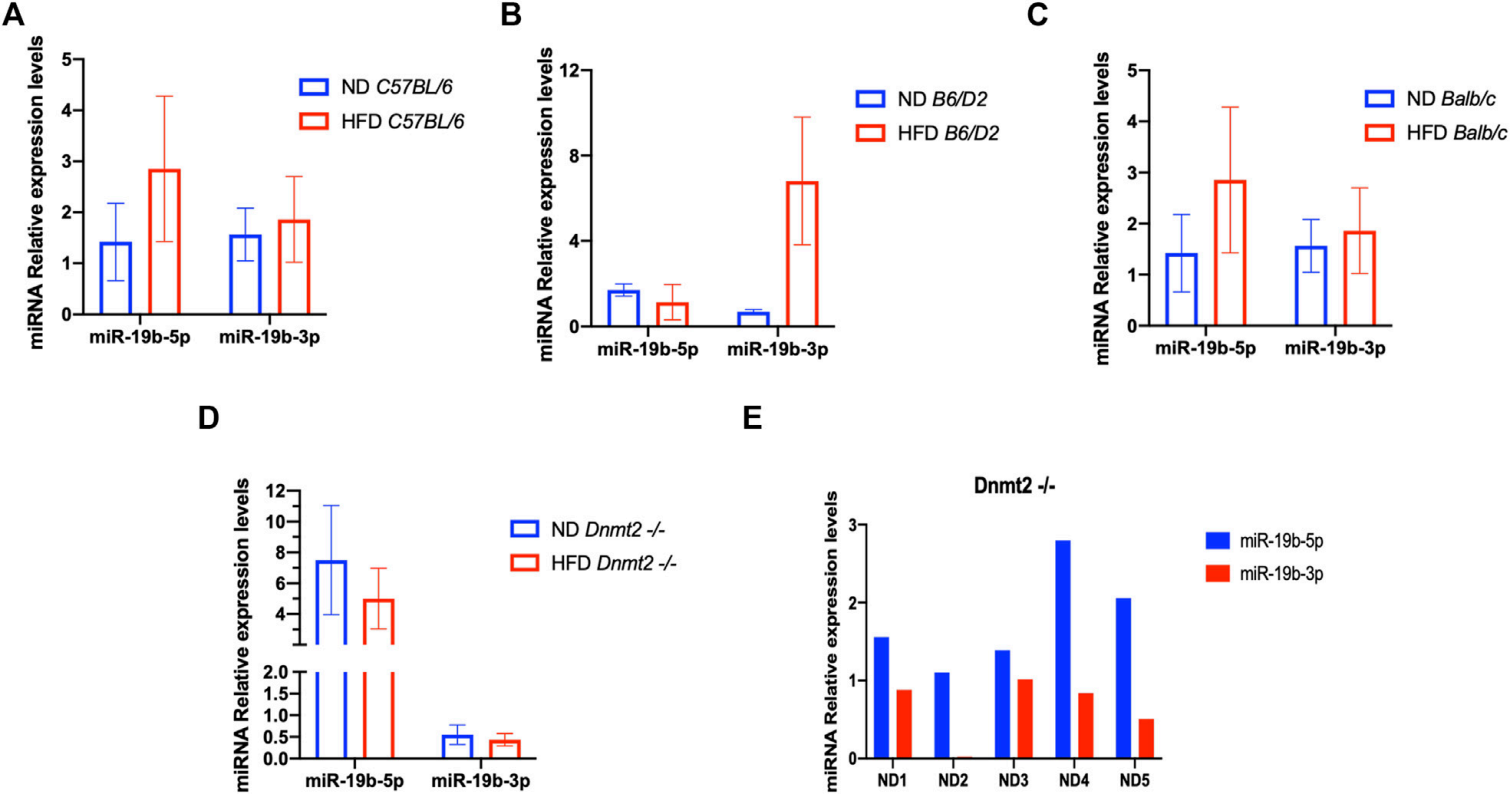
Level of miR 19b in the total sperm RNAs of different lines founders mice (*C57BL/6*, *Balb/c, B6/D2*, and *Dnmt2* ^*−/−C57BL/6*^) fed with HFD (21% butter). Figure 4 shows the expression levels of miRNA from four males in each group. By quantitative RT-PCR analysis, total sperm RNAs of two ND males compared to two HFD males, in *C57BL/6* **(A)**, *B6/D2* **(B)**, *Balb/c* **(C)**, *and Dnmt2* ^*−/−C57BL/6*^ **(D)**. **(E)** RT-PCR results for total sperm RNAs from male-to-male in five independent males in ND.

From a previous study, we found a short list of deregulated miRNAs in the sperm of males raised on HFD. By quantitative RT-PCR analysis, we confirmed the deregulation of miR-182, miR-19a, miR-19b, miR-29a, and miR-340 in testis and sperm RNA of the HFD males compared to ND males. Therefore, we focused our study here on the analysis of one miRNA (miR-19b) given its HFD-MPs inducing activity. Figure 4A is a confirmation of our previous results (Grandjean et al., 2015) and shows that miR-19b-5p in sperm RNA samples was consistently up-regulated in males raised in HFD compared to ND in two inbred lines (*C57BL/6 and Balb/c*), while that in the *B6/D2* F1 hybrid miR-19b-3p is increased. In contrast, in *Dnmt2* ^*−/−C57BL/6*^ males, none of the mir-19b was increased under HFD conditions. Supplementary Figure S14 shows deregulation of sperm miR-19b in *Dnmt2* ^*−/−C57BL/6*^ mice from male to male, regardless of food. To exclude effects of litter in the expression levels of miR-19 in sperm of *Dnmt2* ^*−/−C57BL/6*^ under ND, we tested five males from two separate litters and the results are shown from male to male in Figure 4B. We confirmed varying levels of miR-19 in sperm RNA of *Dnmt2* ^*−/−C57BL/6*^ mice in ND. This means that in *Dnmt2* ^*−/−C57BL/6*^ mice, an alteration of miRNA-19b already exists in males raised on ND, and this marker suggests sporadic changes in the proportion of miR-19b-5p and -3p in its sperm RNA. We also confirm, see below, by RNA-seq analysis with four different *Dnmt2* ^*−/−C57BL/6*^ RNA samples from sperm, the presence of other transcripts variations.

### Sperm transcripts: analysis of free and DNA-associated RNA fraction reveal differences between Dnmt2^−/−^ and control

To get an overall view of sperm RNA profiles, we addressed the issue of examining different sperm RNA fractions. In particular, the RNA fraction associated with the genome in hybrid DNA/RNA structures. Recently, we reported that with a conventional sperm RNA extraction protocol, two sperm RNA fractions could be obtained (Kianmehr et al., 2019). Unlike the classic free RNA fraction extracted with the TRIzol protocol in an aqueous phase (annotated total RNA extract), a sperm RNA fraction consisting of DNA/RNA hybrid molecules is closely associated with the genome (Kianmehr et al., 2019; Rassoulzadegan et al., 2021). The RNA fraction associated with the DNA in the phenol/chloroform interface phase is isolated and purified after treatment with the enzyme *Dnase*. The RNAs associated with the DNA and the free RNA fraction from the sperm are recovered in equivalent quantities, with the hypothesis that the comparison between the proportion of strongly maintained RNA with the DNA fraction on free RNA of the spermatozoa could reveal a change of the hereditary epigenetic marks.

**FIGURE 5:**
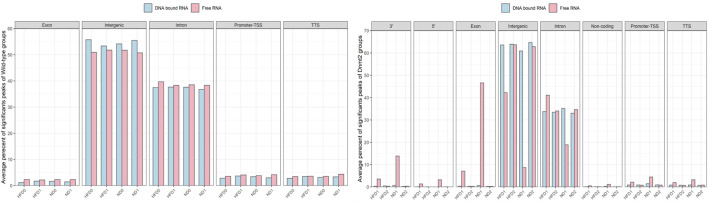
Average percentage of significant peaks per genomic regions of RNA fractions (DNA bound and Free RNA) in spermatozoa from C57BL/6 and *Dnmt2* ^*−/−C57BL/6*^ maintained in high-fat diet (21% butter) and normal diet. Two RNA fractions (DNA-bound and free) are prepared from each male, four wild-type and four *Dnmt2* ^*−/−C57BL/6*^. For each group of samples, a fraction corresponds to the RNAs released from the sperm during extraction in the aqueous phase which we have called the free RNA fraction. Another fraction is released from the purification of genomic DNA after treatment with *Dnase* which we call DNA-bound fraction RNA. The two samples used as template for the RNA-seq analysis and the data were analyzed (see Methods). After obtaining sets of peaks, peak annotation was performed using Homer annotate peaks to relate each peak to nearby genes and genomic features. A false discovery rate (FDR) cut off 0.001 was used for identifying significant peaks. The positions annotated for promoter-TSS, exons, introns, and other features were based on the mm10 transcripts. Males on high-fat diet (HFD) and normal diet (ND), as well as for Dnmt2 samples.

Two populations of sperm RNA were isolated from the founder animals (*C57BL/6* wild-type and *Dnmt2* ^*−/−C57BL/6*^): free (F) and fraction associated with DNA (D) [for more details see Methods and references (Kianmehr et al., 2019; Rassoulzadegan et al., 2021)]. With deep RNA sequencing, we generated data (see Figure 5 and Supplementary Table S4 and Supplementary Figure S15). All sperm transcripts are present in both fractions (F and D).

To identify significant peaks, we searched for peaks throughout the genome, and a peak annotation process was used to relate the location of the peaks/regions to neighboring genes (see bioinformatic analysis methods). This analysis aimed to efficiently assign peaks in terms of important genomic characteristics. Regions of the genome were annotated by sperm RNA fractions of DNA-bound sperm RNAs versus free RNA. The analysis was performed to obtain information if a peak is found in the promoter TSS (transcription start site), 5′UTR, exon (coding), intron, 3′UTR, TTS (transcription termination site), and intergenic and non-coding regions. In normal mice, there was no difference between the number of counts in the samples of ND/HFD and the results are homogenous between the replicates of each sample group Figure 5 (four samples with F and four D fractions, altogether eight RNA-sequences). This means that diet probably does not affect overall peak ratios. More peaks are enriched in F relative to D in the region of the promoter TSS, 5′UTR, exon, intron, 3′UTR, and TTS, and except for the intergenic, more peaks are observed in fraction D. However, in the samples (four samples with F and four D fractions altogether eight RNA-sequences) of *Dnmt2* ^*−/−C57BL/6*^ animals, the profiles are more complex, as a sporadic variation in all regions is observed between samples regardless of the diet conditions Figure 5.

The variations in the peaks of the intergenic region are particularly important, on the other hand, the average percentage of significant peaks of the intergenic region is higher in the samples Dnmt2^−/−C57BL/6^ > 60% than in the samples with wild-type around 50%. In contrast, transcripts from intron regions are somewhat lower in Dnmt2^−/−C57BL/6^ samples (35%) compared to normal animals (38%) regardless of diet conditions (Figures 5A,B). This may suggest that RNA signals induced by HFD should be sought among RNAs derived from intron regions and resistance to diet in intergenic regions.

In addition, transcripts are more abundant as expected in free fractions than in those associated with DNA in all samples. Evaluation of sperm transcripts here by deep sequencing revealed that the amount of transcripts is generally decreased in mature sperm of ND/HFD *Dnmt2* ^*−/−C57BL/6*^ compared to wild-type sperm (Supplementary Table S4).

The loss of two cytosine-5 RNA methyltransferases (Dnmt2 and NSun2) has been reported to be associated with reduced rates of protein synthesis (Tuorto et al., 2012) and genome integrity (Genenncher et al., 2018). In addition, methylation of tRNA at the 38th position cytosine improves the stability of certain tRNAs under altered environmental conditions (Schaefer et al., 2010) or improves the viability of the virus in host cells (Dev et al., 2017). What`s more Dnmt2 activity is required for the establishment of an inherited epigenetic change (altered RNA levels) with “paramutation in c-kit loci” in mice (Kiani et al., 2013). Finally, whereby Dnmt2 is maintained throughout evolution, a role in adaptation to a new environment is assigned (Ashapkin et al., 2016). It is also found that tRNA fragments are in higher levels in the sperm of HFD males (Peng et al., 2012). Small tsRNA fragments of 30–34 nucleotides are derived from the 5′ end of tRNAs. In *Dnmt2* ^*−/−C57BL/6*^ sperm; the profile of tRNA-derived sncRNAs and rRNA-derived sncRNAs is not altered with HFD (Zhang et al., 2018). In other words, in the absence of the Dnmt2 protein, the levels of 30–40 nt RNA fractions in spermatozoa are not changed by HFD.

**FIGURE 6:**
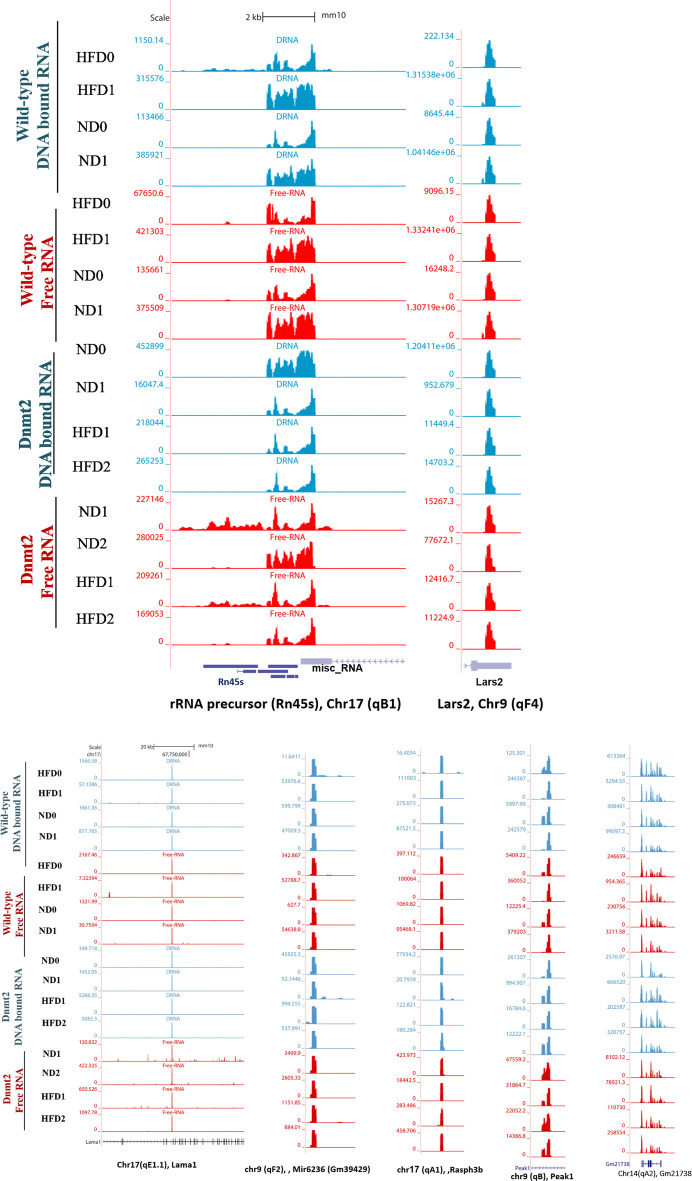
Visualization of highly-expressed signals of DNA-bound and free-RNA moleculees in GRCm38/mm10 in the sperm of wild- type C57BL/6 and Dnmt2-/- mice line in ND and HFD (21% butter, high fat diet). Expression signal tracks of different highly expressed transcripts are visualized which are demonstrated at the bottom of each panel.

Here, we focus on the set of abundant mouse transcripts presented in Figure 6, the AY036118 gene (ETS-linked transcription factor *Mus musculus* ERF, Erf1), Gm42418, and Gm26917 (pre-ribosomal 45S RNA, serving as a precursor for rRNA 28, 18 and 5.8S). The transcripts are visualized using the UCSC genome browser. The expression signal is very high and comparable between samples according to the scaling of the data view on the left (Figure 6), and overlaps with repetitive elements. The pre-ribosomal 45S RNA transcript profiles in the free fraction of *Dnmt2* ^*−/−*^ show changes compared to the wild-type samples (Figure 6). We reveal sporadic changes in the levels of transcripts in the F in *Dnmt2* ^*−/−C57BL/6*^ compared to wild-type sperm. In addition, Figure 6 shows variation in the expression signal of *Mus Musculus* leucyl-tRNA synthetase, mitochondrial (Lars2), transcript variant 1 on chromosome 9 of *Dnmt2* ^*−/−C57BL/6*^ to wild-type *C57BL/6* in HFD/ND.

Unlike the wild type (four samples), each biological replicate of the *Dnmt2* ^*−/−C57BL/6*^ samples exhibits a unique profile and shows variation in transcripts among the four samples. The unpredictable state of transcript levels from *Dnmt2* ^*−/−C57BL/6*^ animals would suggest altered RNA stability as a cause of variation in *Dnmt2* ^*−/−C57BL/6*^ sperm transcripts. Our working hypothesis is that the loss of the Dnmt2 protein triggers random changes in the overall stability of the transcripts and thus influences the half-life dynamics of DNA/RNA hybrids, which may be one of the causes of loss of acquired inheritance in *Dnmt2* ^*−/−C57BL/6*^ line.

The snapshot of the genome browser of pre-rRNA transcripts and leucyl-tRNA synthetase, mitochondrial (lars 2) of normalized BigWig files of two types of sperm RNA fractions (DNA bound RNA and free RNA), shows a normalized expression RNA-seq signal in two mouse lines, wild-type and *Dnmt2* ^*−/−C57BL/6*^ in normal diet (ND) and 21% butter (HFD) through generations. The transcript set comprising the mouse gene AY036118 (transcription factor linked to the ETS of Mus musculus ERF (Erf1), Gm42418, and Gm26917 (A 45S pre-rRNA transcripts) serves as a precursor for 28, 18, and 5.8S rRNA on chromosome 17, overlapping elements repeated by Repeat Masker on the mouse assembly (GRCm38/mm10).

## Discussion

The complexity of the genetic contribution to a common phenotype of weight gain with a diet high in sugar and fat poses many challenges. One question raised here is whether the RNA alterations produced in the sperm of laboratory mouse lines reared on the same HFD (high-fat diet) vary from line to line. In other words, what are the common signals (RNAs) produced for the transmission of high-fat diet-mediated phenotypes (HFD-MPs) between different genotypes? Here, we show that RNA variations are produced in male mice maintained on HFD and are transported by sperm regardless of the genetic background, at least in the four mouse lines tested.

The data indicate that four laboratory lines (*C57BL/6*, *B6/D2*, *Balb/c* and *Dnmt2* ^*−/−*C57BL/6^) nevertheless under the same conditions respond differently in the expression of HFD-MPs and that all founders even with the wild-type genome could be resistant, like the *B6/D2* hybrid*,* and yet pass the changes on to their offspring. Two of the four founder mouse lines do not develop HFD-MPs as indicated previously for the *Dnmt2* ^*−/−C57BL/6*^ mice, and here the hybrid *B6/D2. C57BL/6* is a commonly used inbred mouse line that responds effectively to HFD by increasing fat and body weight, being insulin-resistant (Type 2 diabetes-like), and transmitting all metabolic alterations to at least two generations as previously indicated (Winzell and Ahrén, 2004). *Balb/c* (founder) is another laboratory inbred line which, in HFD condition that does not increase body weight but develops Type 2 diabetes-like phenotypes and passes on HFD-MPs to the F1 and F2 generations. Thus, with *C57BL/6* and *Balb/c* two inbred lines maintained in the same homing, the same food and the same conditions respond differently to HFD. *B6/D2* is a hybrid between *C57BL/6* and *DBA2*; under the same feeding and environmental conditions as the above inbred lines, founder males appears to be resistant to HFD, but in their offspring certain aspects of the phenotype are found as predicted by genetic segregation. Transmission of the phenotype from *B6/D2*-resistant male founders in backcrossing is reminiscent of genetic anticipation (Carpenter, 1994; Martinez-Delgado et al., 2011). This means that despite the founders’ *B6/D2* resistance to HFD, their germ cells have retained a diet-induced change which is then passed on to the next generation. In the F1 generation from *B6/D2* (F0 founder), the Type 2 diabetes-like phenotype is detectable, and in the F2 generation with different genotypic segregation, obesity is displayed. This means that the body weight and Type 2 diabetes-like characters are separately segregating. Finally, a *Dnmt2* null mutation in the *C57BL/6* background abolished the sensitivity to HFD (21–33% butter), and the animals do not gain weight and do not exhibit Type 2 diabetes-like traits, but unlike *B6/D2* the transmission of obesity and diabetes in the offspring is also well abolished. Likewise, using a higher concentration of butter (33% fat), the *Dnmt2* ^*−/−C57BL/6*^ founder mice maintained the same weight, but without symptoms, profiles in both conditions glucose and insulin tolerance tests are changed (more pronounced changes are visible by normalization of the data, Figure 1). Based on previous reports (Zhang et al., 2018), with a diet with a higher concentration of butter (60%), *Dnmt2* ^*−/−*^mice become also obese thus exceeding the controls by the Dnmt2 protein. The Dnmt2 protein methylate tRNAs (Lyko, 2018), and these results demonstrate that the involvement of RNA methylation is an important parameter in the control of health (such as weight and type 2 diabetes like phenotypes) (Lyko, 2018). RNA methylation is a means of modulating RNA stability and enhancing a phenotype (Jeltsch et al., 2017; Lyko, 2018).

Taken together, these results suggest that an additional mechanism governs the development of phenotypes in addition to genetic mechanisms, and this mechanism persists during transfer to the next generation.

### Heterogeneities in transcripts could be a way to escape the disease

Ribosomal heterogeneity is reported in several studies with environmental stresses and is generally accepted as a source of differential ribosome selectivity for the translation circuits of gene expression (Xue and Barna, 2012; Shi et al., 2017). In addition to the protein composition and multiple modifications, heterogeneity is also observed in the composition of ribosomes and ribosomal RNA (rRNA) as diversity generates an impact on translational activities. HFD and protein restriction in mice also affects the levels of ncRNAs in mature sperm, including increased amounts of 5′ glycine tRFs (Cassman et al., 2016). Additionally, here we report the heterogeneity of the levels of not only miR-19b in *Dnmt2* ^*−/−C57BL/6*^ mice but also the activity of miR-19b which is compromised by mixing with sperm RNA from animals raised in ND. This result indicates that overall RNA activities are modifiable by methylation or compensation with total sperm RNAs. Likewise, total RNAs from ND sperm modulates active RNAs from HFD sperm when co-injected in the mixed buffer into fertilized mouse eggs. Changes in the distribution of RNAs could be responsible for changes in activities among sperm RNAs from ND to HFD. The case of the heterogeneous distribution of miR-19b transcripts levels in *Dnmt2* ^*−/−C57BL/6*^ animals indicates that variation is one of the factors which globally influence regulation and signal transmission. These heterogeneities are not specific for miR-19b but are also observed for the highly expressed transcripts revealed here by the analysis of the peak of the two RNA fractions in samples of sperm RNAs from *Dnmt2* ^*−/−C57BL/6*^ mice. In addition, abundant transcripts which were previously reported to be altered in *Dnmt2* ^*−/−C57BL/6*^ mice (Chen et al., 2016a; Zhang et al., 2016) show more dispersed profiles in the free fraction compared to wild-type. The changes in the levels of the free RNA fraction (F) relative to RNA attached to the genome (D) revealed here strongly suggests that hybrid DNA/RNA signals are important in the transmission of epigenetic information from generation to generation.

The free RNA fraction of sperm from four lines induces HFD phenotypes described here. The DNA-bound RNA fraction is not yet tested *in vivo*; since global transcripts are also present in both (the free and DNA bound fraction), it is expected that both fractions are active in microinjection tests in fertilized mouse eggs. We suggest that the activities of RNAs could be dynamic and dependent on the half-life of each RNA as DNA-bound or circulating molecules. The robustness and mechanisms underlying these hereditary changes will require further investigation.

## Methods

### Mice

All animals (mice) and experiments were carried out in compliance with the ARRIVE guidelines (https://arriveguidelines.org). Mice were maintained in building with accord to European and international guidelines and policies in accordance with the animal welfare laws in Turkey in an animal facility at Genkok Institute, Kayseri, Turkey. The experiments were approved by the Turkish ethics committee (see file number below). 1) Institutional and/or licensing committee Erciyes University, Kayseri, Turkey; 2) we confirm all experiments were performed in accordance with relevant guidelines and regulations with permission from Erciyes University committee in an animal facility at Genkok Institute, Kayseri, Turkey. 3) All mice were maintained according to European and international guidelines and policies according to the animal welfare laws in Turkey in an animal facility at Genkok Institute, Kayseri, Turkey.

All protocols in this study were approved by the Committee on the Ethics of Animal Experiments of Erciyes University, Kayseri, Turkey, on Animal Care, permit number: number 13/58, date: 13.03.2013 and permit number 14/116, date: 13.08.2014 from the committee on the Ethics of Animal Experiments of Erciyes University, in compliance with the Guide for the Care and Use of Laboratory Animals published by the US National Institutes of Health (NIH publication no.85-23, revised 1996).

The mice were maintained in a facility under controlled conditions (light from 06:00 to 18:00, 22°C temperature, 55% humidity). The animals were cared for and treated according to the Principles of Laboratory Animal Care (European rules). All tests were performed between 10:00 and 16:00 in isolated rooms.

Four lines of mice were maintained: *C57BL/6*, *B6/D2*, *Balb/c* and *Dnmt2* ^*−/−C57BL/6*^ (Charles River, Wilmington, MA, USA). After weaning, 4-week-old males were divided into two groups. One group was maintained on a normal diet (ND), a standard chow diet (7% simple sugars, 3% fat, 50% polysaccharide, 15% protein (w/w), energy 3.5 kcal/g) and the other was fed a Western diet containing, in addition, 17% casein, 0.3% DL-methionine, 34% sucrose, 14.5% cornstarch, 0.2% cholesterol, 5% cellulose, 7% CM 205B, 1% vit200, and 21% or 35% butter (Diet Western 1,635, Safe, Augy; France).

### Glucose tolerance and insulin tolerance tests

Overnight fasted mice were injected with glucose or insulin in saline buffer. The baseline glucose measurements were analyzed from tail blood before intraperitoneal (ip) glucose injection (2 mg/g body weight) using the OneTouch Vita (LifeScan, Johnson & Johnson, Milpitas, CA, USA) system.

Insulin (NovoRapid^®^, Novo Nordisk, Bagsværd, Denmark) diluted in 0.08 mU/μl saline was injected to a final delivery of 0.8 mU/g body weight.

Blood glucose measurements were taken using tail blood at 0, 15, 30, 45, 90, and 120 min after injection.

### RNA preparation and microinjection

RNA from purified sperm (centrifugation) was extracted using TRIzol reagent (Invitrogen, Life Technologies). All total RNA preparation from mouse sperm were treated with proteinase K 400 microg and Dnase 10–100 ng. The quality of the RNA preparations was verified by spectrometry on an Agilent Bioanalyzer 2,100 apparatus (Agilent, Santa Clara, CA, USA) (Grandjean et al., 2015). Microinjection into fertilized eggs was performed according to established transgenesis methods. Sperm RNA solutions were adjusted to a concentration of 1 μg/ml, and 1–2 pl was microinjected into the male pronuclei of fertilized eggs isolated after normal ovulation and mating to a *Balb/c* strain. Synthetic miRNA (purchased from Sigma, St. Louis, MO, USA) was prepared in a filtered microinjection buffer (10 mM Tris, pH 7.4; 0.1 mM EDTA) at a concentration of 3-5 × 10 (Pomp et al., 2008) molecules/pl.

### RT-PCR and quantitative RT-PCR

miRNA quantitation was performed using miScript Primer Assays (Qiagen, Hilden, Germany), following the manufacturer-recommended protocols. Real-time PCR was performed with a Light Cycler Instrument (Roche Diagnostics, Indianapolis, IN, USA) using the miScript SYBR Green PCR kit (Qiagen, Hilden, Germany).

### Isolation of RNA in R-loop structure as DNA/RNA hybrid for RNA sequencing

A classic TRIzol protocol produces two fractions of RNAs: one in aqueous phase (free fraction) and the other at the junction of the phenol-chloroform DNA fraction. The DNA fraction consists of DNA/RNA hybrid molecules and RNAs which are tightly associated with the genome (Kianmehr et al., 2019; Rassoulzadegan et al., 2021). The RNA fraction associated to DNA is then released from the column by *Dnase* enzyme treatment. Here, we have released sperm cells from epididymis and washed and separated from somatic cells by centrifugation and overnight incubation at 56°C in Tris buffer 20 mM pH8, EDTA 50 mM, with 0.5% SDS, 20 µM dithiothreitol, and 400 μg/ml Proteinase K. Total nucleic acids after enzymatic removal of the proteins was followed by fractionation of RNA and DNA-bound RNA. Extracts were fractionated by the ZR-Duet™ DNA/RNA MiniPrep Plus protocol according to the specifications of the manufacturer (www.zymoresearch.com). After digestion by DNase, 10–100 ng of RNA was prepared and sent to Eurofins for high-throughput sequencing on Illumina Hiseq 2,500 or Illumina MiSeq. Libraries of all samples corresponding to DNA-associated RNA and free-RNA molecules were generated. Small RNA high-throughput sequencing was performed by Eurofins (Europe). Number of mapped reads are summarized in Supplementary Table S4. Considerably, the map rate of *Dnmt2* ^*−/−C57BL/6*^ samples is lowered than HFD/ND samples.

### Bioinformatics analysis

FastQC version 0.11.5 was used to do some quality control checks on the RAW sequence data. RNA-seq reads adapters removed by cutadapt v1.16. Based on the FastQC results, trimming of bad-quality reads was performed using Trimmomatic version 0.36 (10 nucleotides were cropped from the 5′ end of each read, bases were trimmed with Phred score lower than 20 from heading and trailing of each read, and the trimmed reads with a size less than 30 bp were removed) (Bolger et al., 2014). Transcript quantification was performed using Salmon v0.12.0 (Patro et al., 2017) and imported into tximport for downstream analysis. High enriched transcripts were visualized to show difference of expression in HFD/ND and *Dnmt2* ^*−/−C57BL/6*^ samples.

To align trimmed reads to the reference assembly (GRCm38) Hisat2 was used (Pertea, 2016 #10321). Quality of alignment was assessed using stats and plot-bamstats utilities of samtools (Li, 2011 #10323). Bam files were converted into BigWig format using deep tools bamCoverage with the following parameters: ‐of = bigwig - 50- normalizeUsing RPKM. Visualization of normalized BigWig files were performed using the UCSC genome browser (http://genome.ucsc.edu) (Kent et al., 2002).

HOMER version 4.9 was used for peak finding {Heinz, 2010 #10325}. Statistically significant peaks of expression were identified using HOMER (10,000 size of region used for local filtering, fourfold over local region, Poisson p-value over local region <0.0001, false discovery rate (FDR) rate threshold (cut off) < 0.001). After obtaining sets of peaks, peak annotation was performed using Homer “annotate_peaks” to relate each peak to nearby genes and genomic features. Homer (annotate_peaks) is widely used to assign peaks to positions annotated for gene, exon, intron, promoter, 5′ untranslated region (UTR), 3′ UTR, and other genomic features. The promoter-TSS, exons, introns and other features were based on the mm10 transcripts.

### Statistical analysis

Statistical analysis was performed by two-tailed, one-way analysis of variance (ANOVA), uncorrected Fisher’s least significant difference (LSD). Data are expressed as the mean with sem. p < 0.05 was considered to be statistically significant.

## Data Availability

The datasets presented in this study can be found in online repositories. The names of the repository/repositories and accession number(s) can be found below: https://www.ncbi.nlm.nih.gov/geo/, GSE166636.

## Supplementary Material

Supplementary Figure 1 Founder males *Balb/c* raised with high-fat diet of 21% butter (HFD); A: Body weight, B: glucose, (GTT) and C: insulin (ITT) tolerance test (mg/dl).

Supplementary Figure 2 Founder males *B6/D2* raised with high-fat diet of 21% butter (HFD); A: Body weight, B: glucose (GTT), and C: insulin (ITT) tolerance test (mg/dl).

Supplementary Figure 3 Founder males *Dnmt2* ^−/−C57BL/6^ raised with high-fat diet of 21% butter (HFD); A: Body weight, B: glucose (GTT), and C: insulin (ITT) tolerance test (mg/dl).

Supplementary Figure 4 Founder *males C57BL/6* raised with high-fat diet of 21% butter (HFD); A: Body weight, B: glucose (GTT), and C: insulin (ITT) tolerance test (mg/dl).

Supplementary Figure 5 
*F1 generation* males *Balb/c* whose father was raised with high-fat diet of 21% butter (HFD); A: Body weight, B: glucose (GTT), and C: insulin (ITT) tolerance test (mg/dl).

Supplementary Figure 6 F1 generation males *B6/D2* whose father was raised with high-fat diet of 21% butter (HFD); A: Body weight, B: glucose (GTT), and C: insulin (ITT) tolerance test (mg/dl).

Supplementary Figure 7 
*F1* generation males *Dnmt2* ^*−/−C57BL/6*^ whose father was raised with high-fat diet of 21% butter (HFD); A: Body weight, B: glucose (GTT), and C: insulin (ITT) tolerance test (mg/dl).

Supplementary Figure 8 
*F1 generation males C57BL/6* whose father was raised with high-fat diet of 21% butter (HFD); A: Body weight, B: glucose (GTT), and C: insulin (ITT) tolerance test (mg/dl).

Supplementary Figure 9 
*F2 generation* males *Balb/c* whose grand-father was raised in high-fat diet of 21% butter (HFD); A: Body weight, B: glucose (GTT), and C: insulin (ITT) tolerance test (mg/dl).

Supplementary Figure 10 
*F2 generation* males *B6/D2* whose grand-father was raised in high-fat diet of 21% butter (HFD); A: Body weight, B: glucose (GTT), and C: insulin (ITT) tolerance test (mg/dl).

Supplementary Figure 11 
*F2 generation* males *Dnmt2* ^*−/−C57BL/6*^ *F2* whose grand-father was raised in high-fat diet of 21% butter (HFD); A: Body weight, B: glucose (GTT), and C: insulin (ITT) tolerance test (mg/dl).

Supplementary Figure 12 
*F2 generation* males *C57BL/6* whose grand-father was raised in high-fat diet of 21% butter (HFD); A: Body weight, B: glucose (GTT), and C: insulin (ITT) tolerance test (mg/dl).

Supplementary Figure 13 Microinjection of total RNA of sperm from HFD-resistant males perturbs the health of susceptible offspring (*Balb/c*). Total sperm RNAs prepared from males raised on ND or HFD were microinjected into *Balb/c* fertilized eggs, as described in the Methods section. All mice were raised on an ND. Body weights at 15 weeks are presented for females born after microinjection of total sperm RNAs for the three lines, *Balb/c*, ND, HFD, Mixed ND/HFD, and HFD RNA added RNase before microinjection and finally HFD *Dnmt2* ^*−/−C57BL/6*^ RNA. *n* = number of mice in each group. Body weight is shown in each group from 6 to 14 weeks of age for females, respectively. Statistical analysis was performed by two-tailed, one-way analysis of variance (ANOVA), uncorrected Fisher’s least significant difference (LSD). *p < 0.05, **p < 0.01, ***p < 0.001 ****p < 0.0001 (*ND* vs. each group). Ns, not significant. All data are plotted as mean ± S.E.M.

Supplementary Figure 14 miR-19 expression in sperm of individual males. Each line corresponds to the results obtained from a male (repeated with three aliquots). The miR-19b-5p and -3p were systematically dysregulated in all males raised on HFD, but not in *Dnmt2* ^*−/−C57BL/6*^ males. In the latter, we found that miR-19b deregulation was heterogeneous between males, irrespective of the food type. Diet does not affect the level of miR19b in the sperm of *Dnmt2* ^*−/−C57BL/6*^.

Supplementary Figure 15 RNA fractions (D and R) in the sperm of wild-type males in normal diet (ND) and 21% butter (high fat diet, HFD). Rn18s-rs5, n-R5s100 in sperm samples (13A) ND/HFD, wild-types, and (13B) *Dnmt2* ^*−/−C57BL/6*^.

Supplementary Table 1 (Raw data of Figure 1) (A) Body weight (g), glucose (GTT), and insulin (ITT) tolerance test (mg/dl), males *Dnmt2* ^*−/−C57BL*/6^ (21% butter high fat diet). (B) Body weight (g), glucose (GTT), and insulin (ITT) tolerance test (mg/dl), males *Dnmt2* ^*−/−C57BL*/6^ mice (33% butter high fat diet).

Supplementary Table 2 (Raw data of Figure 2) Body weight (g), glucose (GTT), and insulin (ITT) tolerance test (mg/dl) *Balb/c* animals born after microinjection with total sperm RNAs.

Supplementary Table 3 (Raw data of Figure 3) Body weight (g), glucose (GTT), and insulin (ITT) tolerance test (mg/dl), *C57BL/6* F0 males born after total sperm RNA and miR-19b microinjection. Data were analyzed via one-way ANOVA test and the differences among the groups were determined by Tukey’s post hoc test. There are no significant differences among the groups (p > 0.05). All results are expressed as mean and SEM.

Supplementary Table 4 RNA-seq of fractions (D and R) in the sperm of two different mouse lines (*C57BL/6* and *Dnmt2* ^*−/−C57BL*/6^) in normal diet and 21% butter (HFD).
