# Supplementary material for: Mouse Paternal RNAs Initiate a Pattern of Metabolic Disorders in a Line-Dependent Manner
Source: Front Genet. 2022 Mar 28;13:839841. doi: 10.3389/fgene.2022.839841 (PMC8996111; doi:10.3389/fgene.2022.839841)
Supplement: Supplementary file 1 [file Presentation1.PPTX]

## Slide 1
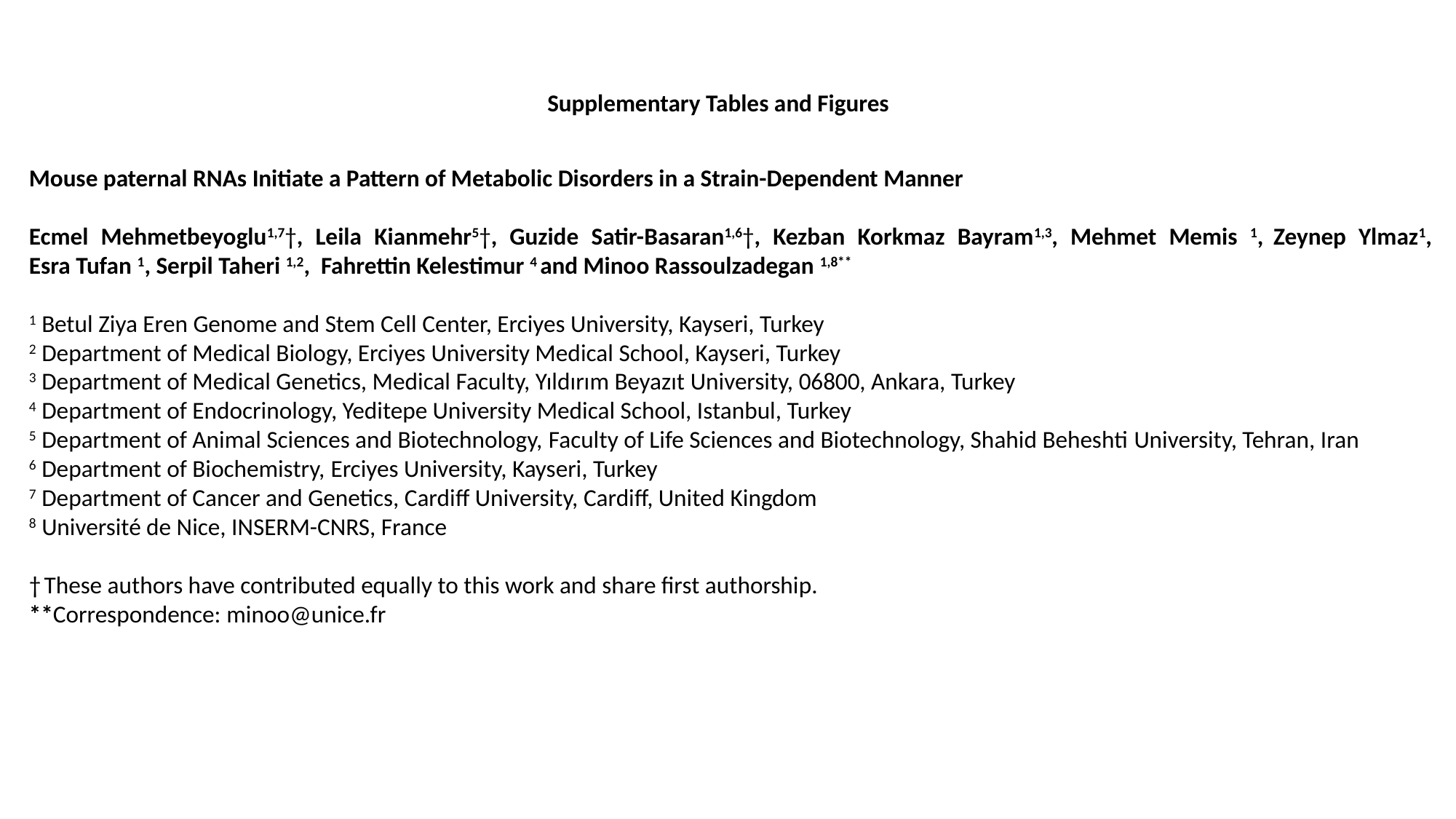

Supplementary Tables and Figures
Mouse paternal RNAs Initiate a Pattern of Metabolic Disorders in a Strain-Dependent Manner
Ecmel Mehmetbeyoglu1,7†, Leila Kianmehr5†, Guzide Satir-Basaran1,6†, Kezban Korkmaz Bayram1,3, Mehmet Memis 1, Zeynep Ylmaz1,
Esra Tufan 1, Serpil Taheri 1,2, Fahrettin Kelestimur 4 and Minoo Rassoulzadegan 1,8**
1 Betul Ziya Eren Genome and Stem Cell Center, Erciyes University, Kayseri, Turkey
2 Department of Medical Biology, Erciyes University Medical School, Kayseri, Turkey
3 Department of Medical Genetics, Medical Faculty, Yıldırım Beyazıt University, 06800, Ankara, Turkey
4 Department of Endocrinology, Yeditepe University Medical School, Istanbul, Turkey
5 Department of Animal Sciences and Biotechnology, Faculty of Life Sciences and Biotechnology, Shahid Beheshti University, Tehran, Iran
6 Department of Biochemistry, Erciyes University, Kayseri, Turkey
7 Department of Cancer and Genetics, Cardiff University, Cardiff, United Kingdom
8 Université de Nice, INSERM-CNRS, France
† These authors have contributed equally to this work and share first authorship.
**Correspondence: minoo@unice.fr

## Slide 2
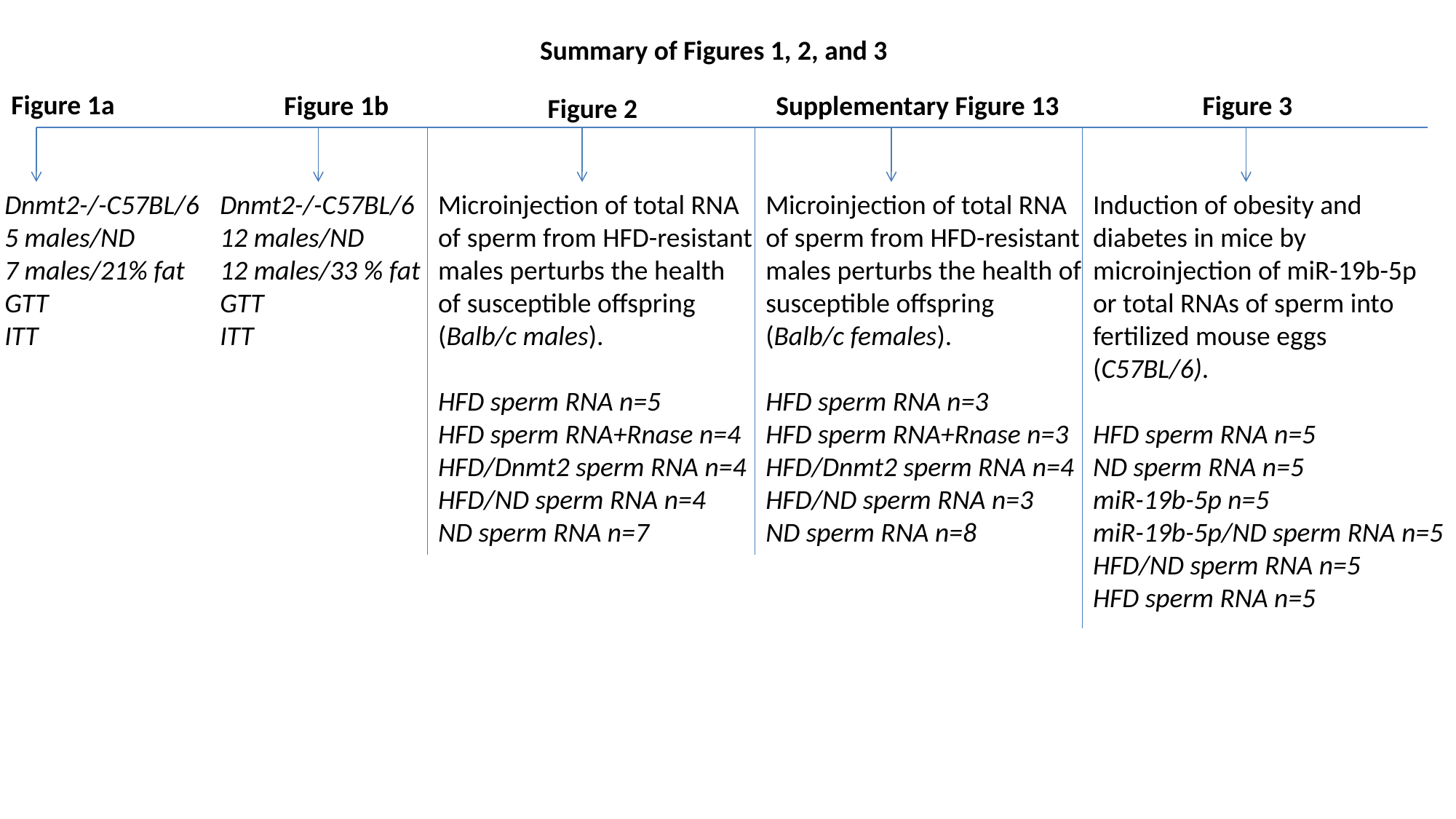

Summary of Figures 1, 2, and 3
Figure 1a
Figure 1b
Supplementary Figure 13
Figure 3
Figure 2
Dnmt2-/-C57BL/6
5 males/ND
7 males/21% fat
GTT
ITT
Dnmt2-/-C57BL/6
12 males/ND
12 males/33 % fat
GTT
ITT
Microinjection of total RNA of sperm from HFD-resistant males perturbs the health of susceptible offspring (Balb/c males).
HFD sperm RNA n=5
HFD sperm RNA+Rnase n=4
HFD/Dnmt2 sperm RNA n=4
HFD/ND sperm RNA n=4
ND sperm RNA n=7
Microinjection of total RNA of sperm from HFD-resistant males perturbs the health of susceptible offspring
(Balb/c females).
HFD sperm RNA n=3
HFD sperm RNA+Rnase n=3
HFD/Dnmt2 sperm RNA n=4
HFD/ND sperm RNA n=3
ND sperm RNA n=8
Induction of obesity and diabetes in mice by microinjection of miR-19b-5p or total RNAs of sperm into fertilized mouse eggs (C57BL/6).
HFD sperm RNA n=5
ND sperm RNA n=5
miR-19b-5p n=5
miR-19b-5p/ND sperm RNA n=5
HFD/ND sperm RNA n=5
HFD sperm RNA n=5

## Slide 3
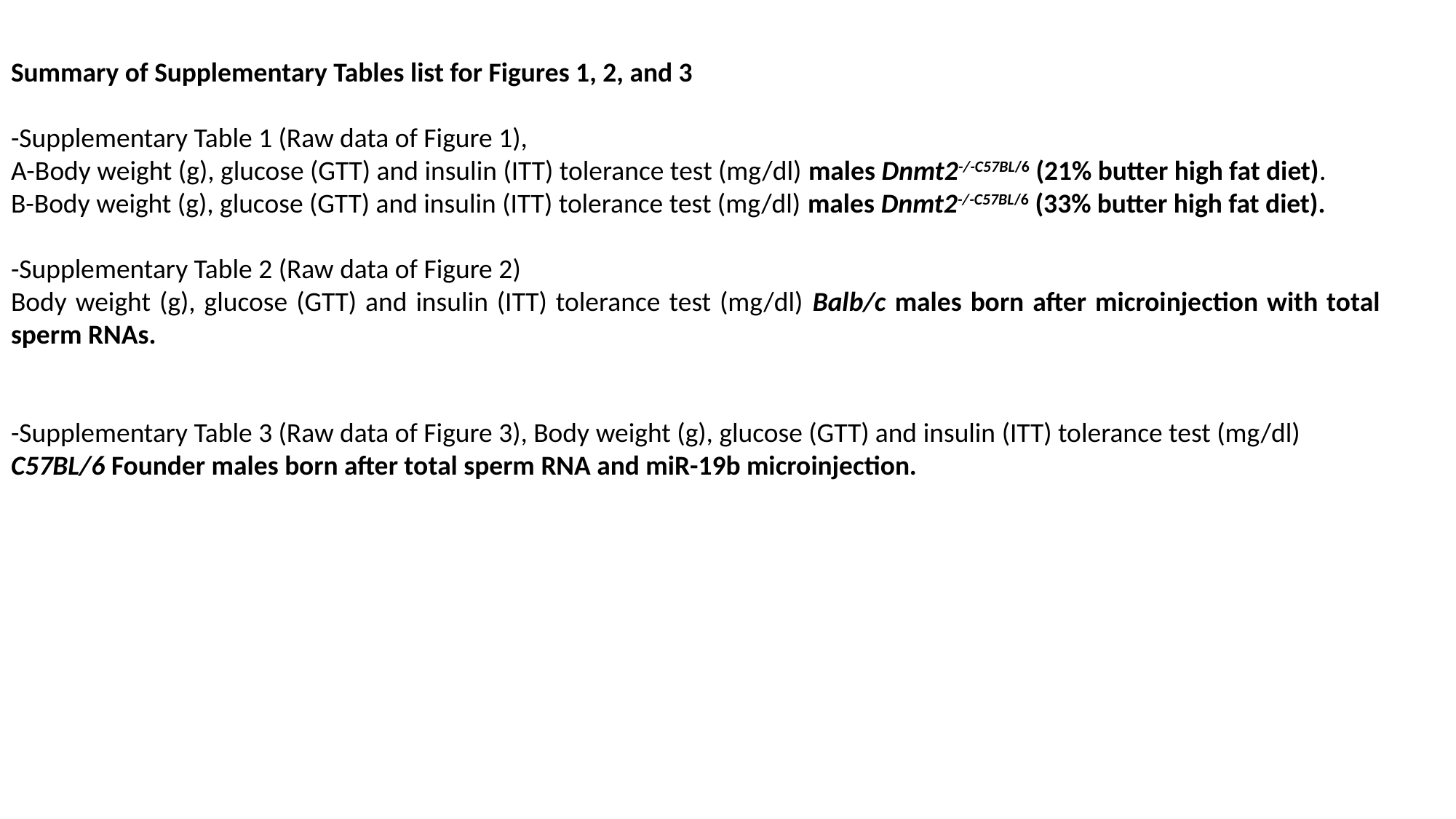

Summary of Supplementary Tables list for Figures 1, 2, and 3
-Supplementary Table 1 (Raw data of Figure 1),
A-Body weight (g), glucose (GTT) and insulin (ITT) tolerance test (mg/dl) males Dnmt2-/-C57BL/6 (21% butter high fat diet).
B-Body weight (g), glucose (GTT) and insulin (ITT) tolerance test (mg/dl) males Dnmt2-/-C57BL/6 (33% butter high fat diet).
-Supplementary Table 2 (Raw data of Figure 2)
Body weight (g), glucose (GTT) and insulin (ITT) tolerance test (mg/dl) Balb/c males born after microinjection with total sperm RNAs.
-Supplementary Table 3 (Raw data of Figure 3), Body weight (g), glucose (GTT) and insulin (ITT) tolerance test (mg/dl)
C57BL/6 Founder males born after total sperm RNA and miR-19b microinjection.

## Slide 4
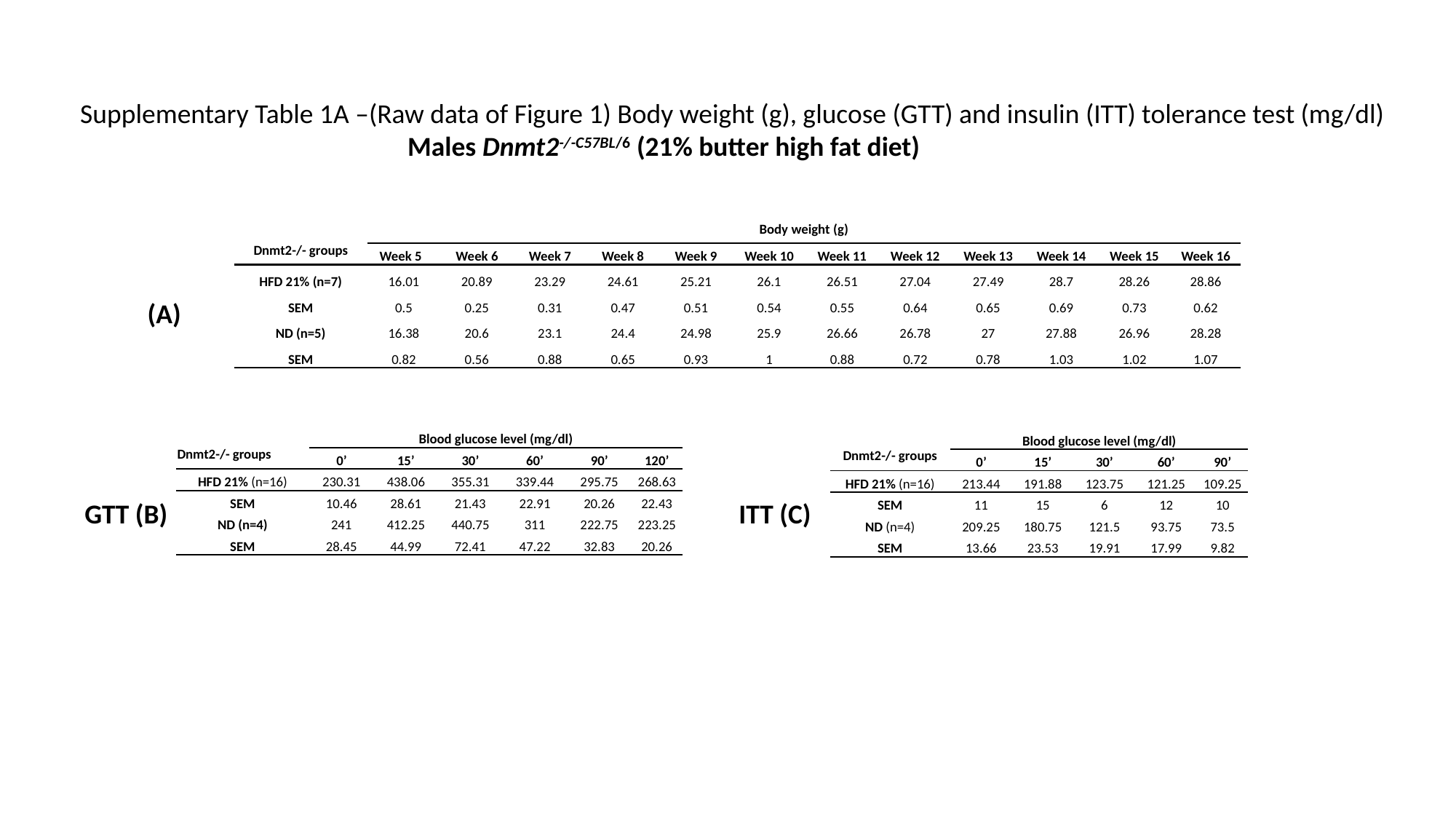

Supplementary Table 1A –(Raw data of Figure 1) Body weight (g), glucose (GTT) and insulin (ITT) tolerance test (mg/dl)
			Males Dnmt2-/-C57BL/6 (21% butter high fat diet)
| | Body weight (g) | | | | | | | | | | | |
| --- | --- | --- | --- | --- | --- | --- | --- | --- | --- | --- | --- | --- |
| Dnmt2-/- groups | Week 5 | Week 6 | Week 7 | Week 8 | Week 9 | Week 10 | Week 11 | Week 12 | Week 13 | Week 14 | Week 15 | Week 16 |
| HFD 21% (n=7) | 16.01 | 20.89 | 23.29 | 24.61 | 25.21 | 26.1 | 26.51 | 27.04 | 27.49 | 28.7 | 28.26 | 28.86 |
| SEM | 0.5 | 0.25 | 0.31 | 0.47 | 0.51 | 0.54 | 0.55 | 0.64 | 0.65 | 0.69 | 0.73 | 0.62 |
| ND (n=5) | 16.38 | 20.6 | 23.1 | 24.4 | 24.98 | 25.9 | 26.66 | 26.78 | 27 | 27.88 | 26.96 | 28.28 |
| SEM | 0.82 | 0.56 | 0.88 | 0.65 | 0.93 | 1 | 0.88 | 0.72 | 0.78 | 1.03 | 1.02 | 1.07 |
(A)
| | Blood glucose level (mg/dl) | | | | | |
| --- | --- | --- | --- | --- | --- | --- |
| Dnmt2-/- groups | 0’ | 15’ | 30’ | 60’ | 90’ | 120’ |
| HFD 21% (n=16) | 230.31 | 438.06 | 355.31 | 339.44 | 295.75 | 268.63 |
| SEM | 10.46 | 28.61 | 21.43 | 22.91 | 20.26 | 22.43 |
| ND (n=4) | 241 | 412.25 | 440.75 | 311 | 222.75 | 223.25 |
| SEM | 28.45 | 44.99 | 72.41 | 47.22 | 32.83 | 20.26 |
| | Blood glucose level (mg/dl) | | | | |
| --- | --- | --- | --- | --- | --- |
| Dnmt2-/- groups | 0’ | 15’ | 30’ | 60’ | 90’ |
| HFD 21% (n=16) | 213.44 | 191.88 | 123.75 | 121.25 | 109.25 |
| SEM | 11 | 15 | 6 | 12 | 10 |
| ND (n=4) | 209.25 | 180.75 | 121.5 | 93.75 | 73.5 |
| SEM | 13.66 | 23.53 | 19.91 | 17.99 | 9.82 |
GTT (B)
ITT (C)

## Slide 5
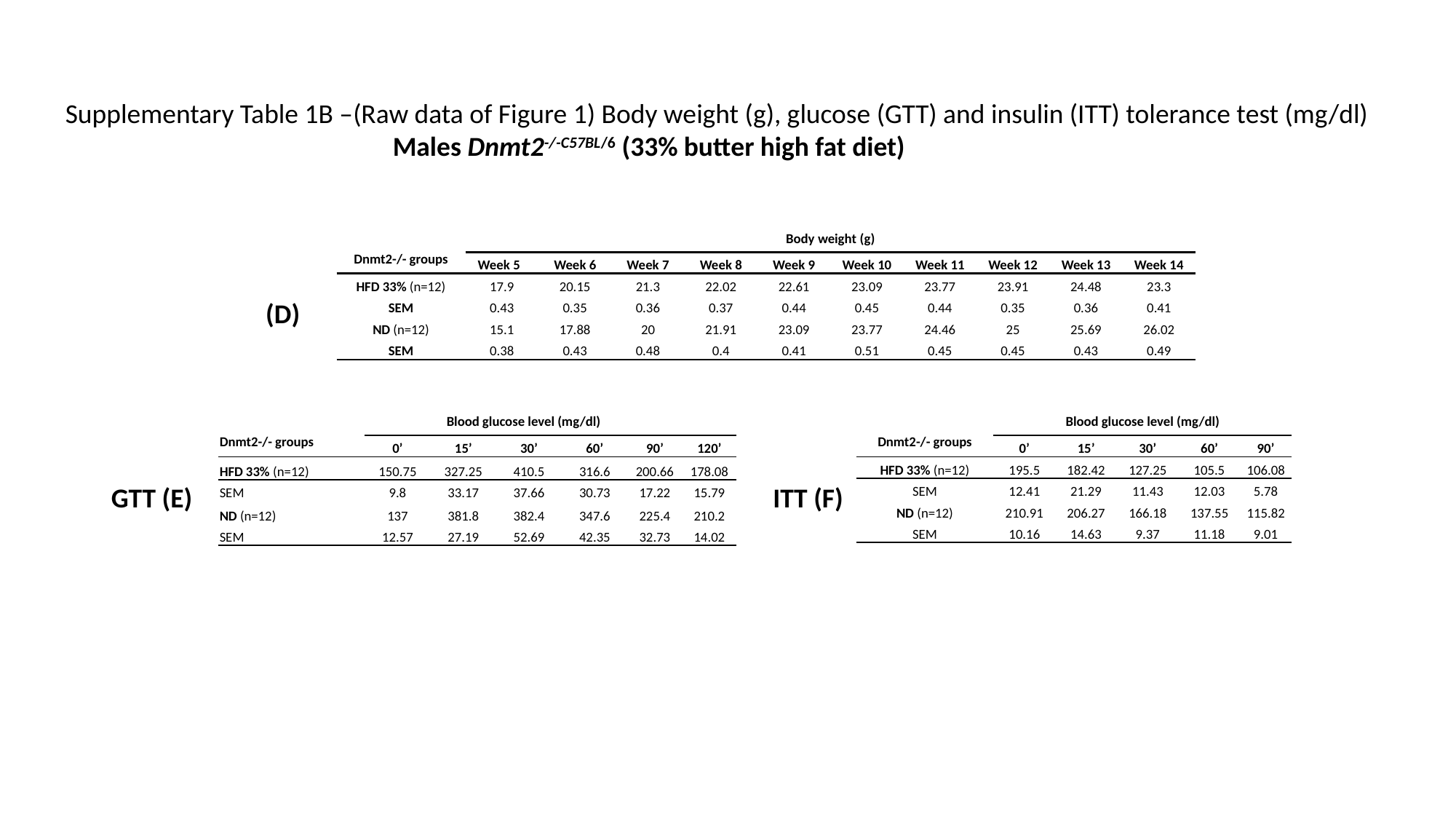

Supplementary Table 1B –(Raw data of Figure 1) Body weight (g), glucose (GTT) and insulin (ITT) tolerance test (mg/dl) 			Males Dnmt2-/-C57BL/6 (33% butter high fat diet)
| | Body weight (g) | | | | | | | | | |
| --- | --- | --- | --- | --- | --- | --- | --- | --- | --- | --- |
| Dnmt2-/- groups | Week 5 | Week 6 | Week 7 | Week 8 | Week 9 | Week 10 | Week 11 | Week 12 | Week 13 | Week 14 |
| HFD 33% (n=12) | 17.9 | 20.15 | 21.3 | 22.02 | 22.61 | 23.09 | 23.77 | 23.91 | 24.48 | 23.3 |
| SEM | 0.43 | 0.35 | 0.36 | 0.37 | 0.44 | 0.45 | 0.44 | 0.35 | 0.36 | 0.41 |
| ND (n=12) | 15.1 | 17.88 | 20 | 21.91 | 23.09 | 23.77 | 24.46 | 25 | 25.69 | 26.02 |
| SEM | 0.38 | 0.43 | 0.48 | 0.4 | 0.41 | 0.51 | 0.45 | 0.45 | 0.43 | 0.49 |
(D)
| | Blood glucose level (mg/dl) | | | | | |
| --- | --- | --- | --- | --- | --- | --- |
| Dnmt2-/- groups | 0’ | 15’ | 30’ | 60’ | 90’ | 120’ |
| HFD 33% (n=12) | 150.75 | 327.25 | 410.5 | 316.6 | 200.66 | 178.08 |
| SEM | 9.8 | 33.17 | 37.66 | 30.73 | 17.22 | 15.79 |
| ND (n=12) | 137 | 381.8 | 382.4 | 347.6 | 225.4 | 210.2 |
| SEM | 12.57 | 27.19 | 52.69 | 42.35 | 32.73 | 14.02 |
| | Blood glucose level (mg/dl) | | | | |
| --- | --- | --- | --- | --- | --- |
| Dnmt2-/- groups | 0’ | 15’ | 30’ | 60’ | 90’ |
| HFD 33% (n=12) | 195.5 | 182.42 | 127.25 | 105.5 | 106.08 |
| SEM | 12.41 | 21.29 | 11.43 | 12.03 | 5.78 |
| ND (n=12) | 210.91 | 206.27 | 166.18 | 137.55 | 115.82 |
| SEM | 10.16 | 14.63 | 9.37 | 11.18 | 9.01 |
GTT (E)
ITT (F)

## Slide 6
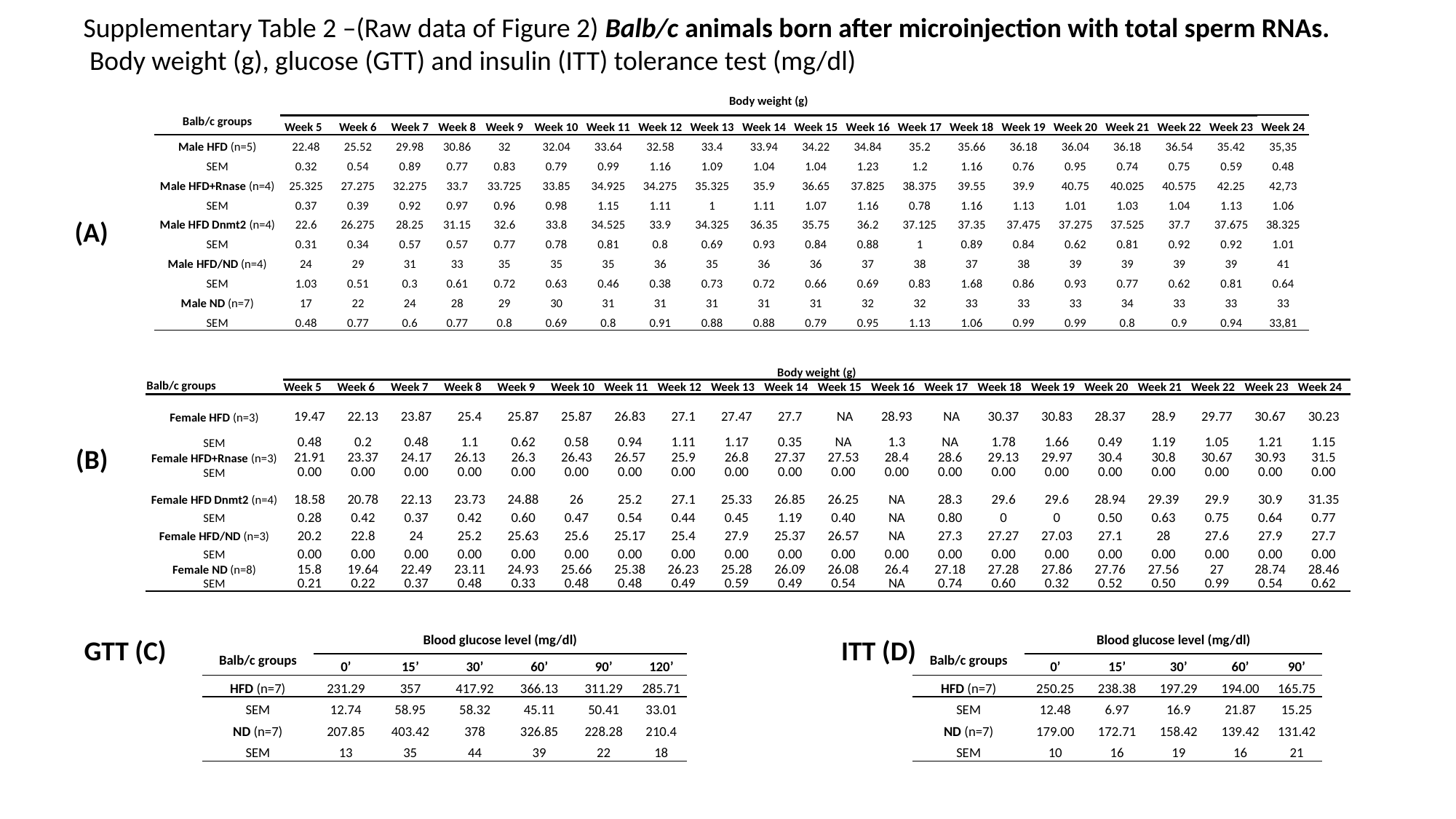

Supplementary Table 2 –(Raw data of Figure 2) Balb/c animals born after microinjection with total sperm RNAs.
 Body weight (g), glucose (GTT) and insulin (ITT) tolerance test (mg/dl)
| | Body weight (g) | | | | | | | | | | | | | | | | | | | |
| --- | --- | --- | --- | --- | --- | --- | --- | --- | --- | --- | --- | --- | --- | --- | --- | --- | --- | --- | --- | --- |
| Balb/c groups | Week 5 | Week 6 | Week 7 | Week 8 | Week 9 | Week 10 | Week 11 | Week 12 | Week 13 | Week 14 | Week 15 | Week 16 | Week 17 | Week 18 | Week 19 | Week 20 | Week 21 | Week 22 | Week 23 | Week 24 |
| Male HFD (n=5) | 22.48 | 25.52 | 29.98 | 30.86 | 32 | 32.04 | 33.64 | 32.58 | 33.4 | 33.94 | 34.22 | 34.84 | 35.2 | 35.66 | 36.18 | 36.04 | 36.18 | 36.54 | 35.42 | 35,35 |
| SEM | 0.32 | 0.54 | 0.89 | 0.77 | 0.83 | 0.79 | 0.99 | 1.16 | 1.09 | 1.04 | 1.04 | 1.23 | 1.2 | 1.16 | 0.76 | 0.95 | 0.74 | 0.75 | 0.59 | 0.48 |
| Male HFD+Rnase (n=4) | 25.325 | 27.275 | 32.275 | 33.7 | 33.725 | 33.85 | 34.925 | 34.275 | 35.325 | 35.9 | 36.65 | 37.825 | 38.375 | 39.55 | 39.9 | 40.75 | 40.025 | 40.575 | 42.25 | 42,73 |
| SEM | 0.37 | 0.39 | 0.92 | 0.97 | 0.96 | 0.98 | 1.15 | 1.11 | 1 | 1.11 | 1.07 | 1.16 | 0.78 | 1.16 | 1.13 | 1.01 | 1.03 | 1.04 | 1.13 | 1.06 |
| Male HFD Dnmt2 (n=4) | 22.6 | 26.275 | 28.25 | 31.15 | 32.6 | 33.8 | 34.525 | 33.9 | 34.325 | 36.35 | 35.75 | 36.2 | 37.125 | 37.35 | 37.475 | 37.275 | 37.525 | 37.7 | 37.675 | 38.325 |
| SEM | 0.31 | 0.34 | 0.57 | 0.57 | 0.77 | 0.78 | 0.81 | 0.8 | 0.69 | 0.93 | 0.84 | 0.88 | 1 | 0.89 | 0.84 | 0.62 | 0.81 | 0.92 | 0.92 | 1.01 |
| Male HFD/ND (n=4) | 24 | 29 | 31 | 33 | 35 | 35 | 35 | 36 | 35 | 36 | 36 | 37 | 38 | 37 | 38 | 39 | 39 | 39 | 39 | 41 |
| SEM | 1.03 | 0.51 | 0.3 | 0.61 | 0.72 | 0.63 | 0.46 | 0.38 | 0.73 | 0.72 | 0.66 | 0.69 | 0.83 | 1.68 | 0.86 | 0.93 | 0.77 | 0.62 | 0.81 | 0.64 |
| Male ND (n=7) | 17 | 22 | 24 | 28 | 29 | 30 | 31 | 31 | 31 | 31 | 31 | 32 | 32 | 33 | 33 | 33 | 34 | 33 | 33 | 33 |
| SEM | 0.48 | 0.77 | 0.6 | 0.77 | 0.8 | 0.69 | 0.8 | 0.91 | 0.88 | 0.88 | 0.79 | 0.95 | 1.13 | 1.06 | 0.99 | 0.99 | 0.8 | 0.9 | 0.94 | 33,81 |
(A)
| | Body weight (g) | | | | | | | | | | | | | | | | | | | |
| --- | --- | --- | --- | --- | --- | --- | --- | --- | --- | --- | --- | --- | --- | --- | --- | --- | --- | --- | --- | --- |
| Balb/c groups | Week 5 | Week 6 | Week 7 | Week 8 | Week 9 | Week 10 | Week 11 | Week 12 | Week 13 | Week 14 | Week 15 | Week 16 | Week 17 | Week 18 | Week 19 | Week 20 | Week 21 | Week 22 | Week 23 | Week 24 |
| Female HFD (n=3) | 19.47 | 22.13 | 23.87 | 25.4 | 25.87 | 25.87 | 26.83 | 27.1 | 27.47 | 27.7 | NA | 28.93 | NA | 30.37 | 30.83 | 28.37 | 28.9 | 29.77 | 30.67 | 30.23 |
| SEM | 0.48 | 0.2 | 0.48 | 1.1 | 0.62 | 0.58 | 0.94 | 1.11 | 1.17 | 0.35 | NA | 1.3 | NA | 1.78 | 1.66 | 0.49 | 1.19 | 1.05 | 1.21 | 1.15 |
| Female HFD+Rnase (n=3) | 21.91 | 23.37 | 24.17 | 26.13 | 26.3 | 26.43 | 26.57 | 25.9 | 26.8 | 27.37 | 27.53 | 28.4 | 28.6 | 29.13 | 29.97 | 30.4 | 30.8 | 30.67 | 30.93 | 31.5 |
| SEM | 0.00 | 0.00 | 0.00 | 0.00 | 0.00 | 0.00 | 0.00 | 0.00 | 0.00 | 0.00 | 0.00 | 0.00 | 0.00 | 0.00 | 0.00 | 0.00 | 0.00 | 0.00 | 0.00 | 0.00 |
| Female HFD Dnmt2 (n=4) | 18.58 | 20.78 | 22.13 | 23.73 | 24.88 | 26 | 25.2 | 27.1 | 25.33 | 26.85 | 26.25 | NA | 28.3 | 29.6 | 29.6 | 28.94 | 29.39 | 29.9 | 30.9 | 31.35 |
| SEM | 0.28 | 0.42 | 0.37 | 0.42 | 0.60 | 0.47 | 0.54 | 0.44 | 0.45 | 1.19 | 0.40 | NA | 0.80 | 0 | 0 | 0.50 | 0.63 | 0.75 | 0.64 | 0.77 |
| Female HFD/ND (n=3) | 20.2 | 22.8 | 24 | 25.2 | 25.63 | 25.6 | 25.17 | 25.4 | 27.9 | 25.37 | 26.57 | NA | 27.3 | 27.27 | 27.03 | 27.1 | 28 | 27.6 | 27.9 | 27.7 |
| SEM | 0.00 | 0.00 | 0.00 | 0.00 | 0.00 | 0.00 | 0.00 | 0.00 | 0.00 | 0.00 | 0.00 | 0.00 | 0.00 | 0.00 | 0.00 | 0.00 | 0.00 | 0.00 | 0.00 | 0.00 |
| Female ND (n=8) | 15.8 | 19.64 | 22.49 | 23.11 | 24.93 | 25.66 | 25.38 | 26.23 | 25.28 | 26.09 | 26.08 | 26.4 | 27.18 | 27.28 | 27.86 | 27.76 | 27.56 | 27 | 28.74 | 28.46 |
| SEM | 0.21 | 0.22 | 0.37 | 0.48 | 0.33 | 0.48 | 0.48 | 0.49 | 0.59 | 0.49 | 0.54 | NA | 0.74 | 0.60 | 0.32 | 0.52 | 0.50 | 0.99 | 0.54 | 0.62 |
(B)
GTT (C)
| | Blood glucose level (mg/dl) | | | | | |
| --- | --- | --- | --- | --- | --- | --- |
| Balb/c groups | 0’ | 15’ | 30’ | 60’ | 90’ | 120’ |
| HFD (n=7) | 231.29 | 357 | 417.92 | 366.13 | 311.29 | 285.71 |
| SEM | 12.74 | 58.95 | 58.32 | 45.11 | 50.41 | 33.01 |
| ND (n=7) | 207.85 | 403.42 | 378 | 326.85 | 228.28 | 210.4 |
| SEM | 13 | 35 | 44 | 39 | 22 | 18 |
ITT (D)
| | Blood glucose level (mg/dl) | | | | |
| --- | --- | --- | --- | --- | --- |
| Balb/c groups | 0’ | 15’ | 30’ | 60’ | 90’ |
| HFD (n=7) | 250.25 | 238.38 | 197.29 | 194.00 | 165.75 |
| SEM | 12.48 | 6.97 | 16.9 | 21.87 | 15.25 |
| ND (n=7) | 179.00 | 172.71 | 158.42 | 139.42 | 131.42 |
| SEM | 10 | 16 | 19 | 16 | 21 |

## Slide 7
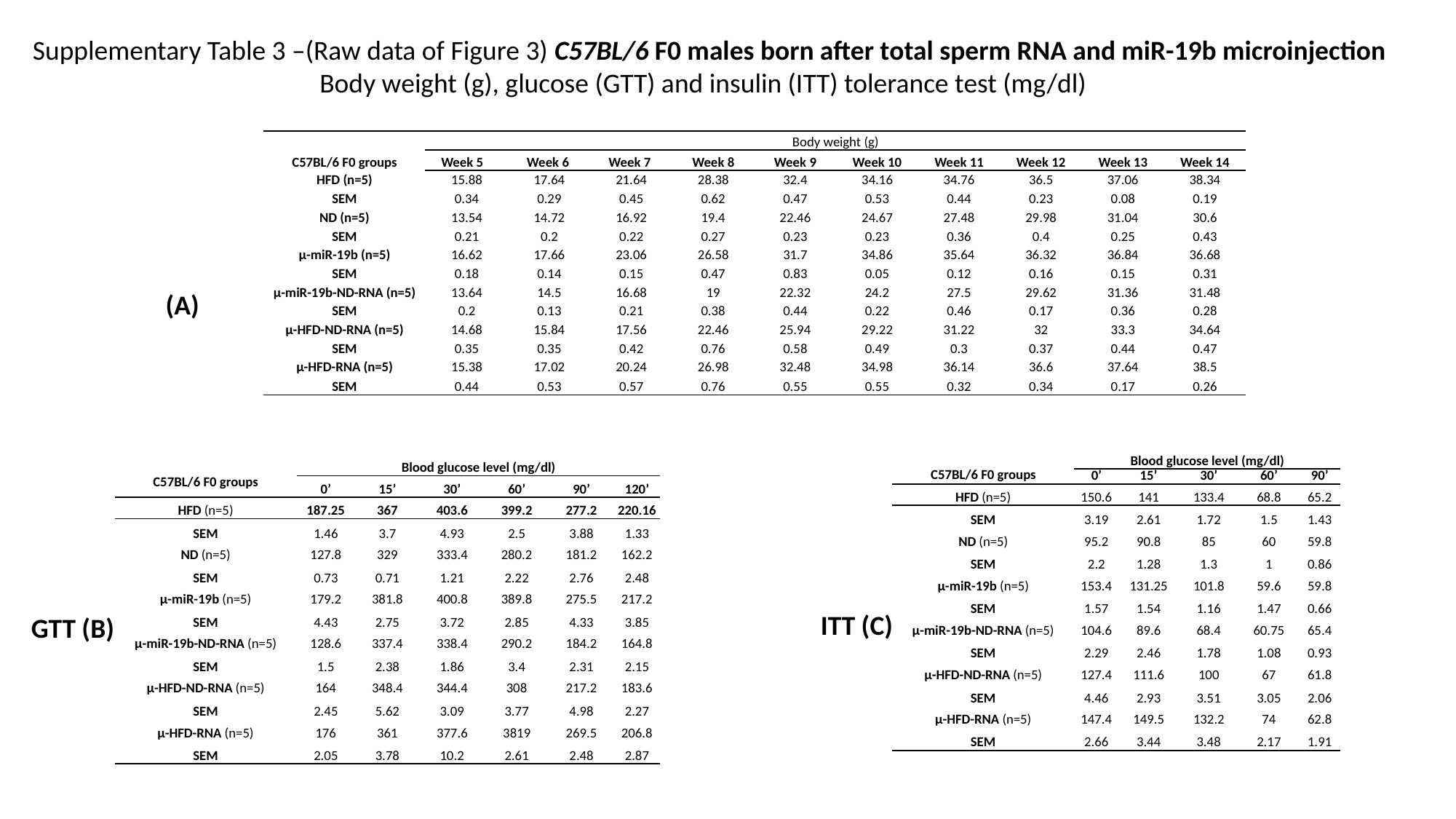

Supplementary Table 3 –(Raw data of Figure 3) C57BL/6 F0 males born after total sperm RNA and miR-19b microinjection
Body weight (g), glucose (GTT) and insulin (ITT) tolerance test (mg/dl)
| | Body weight (g) | | | | | | | | | |
| --- | --- | --- | --- | --- | --- | --- | --- | --- | --- | --- |
| C57BL/6 F0 groups | Week 5 | Week 6 | Week 7 | Week 8 | Week 9 | Week 10 | Week 11 | Week 12 | Week 13 | Week 14 |
| HFD (n=5) | 15.88 | 17.64 | 21.64 | 28.38 | 32.4 | 34.16 | 34.76 | 36.5 | 37.06 | 38.34 |
| SEM | 0.34 | 0.29 | 0.45 | 0.62 | 0.47 | 0.53 | 0.44 | 0.23 | 0.08 | 0.19 |
| ND (n=5) | 13.54 | 14.72 | 16.92 | 19.4 | 22.46 | 24.67 | 27.48 | 29.98 | 31.04 | 30.6 |
| SEM | 0.21 | 0.2 | 0.22 | 0.27 | 0.23 | 0.23 | 0.36 | 0.4 | 0.25 | 0.43 |
| µ-miR-19b (n=5) | 16.62 | 17.66 | 23.06 | 26.58 | 31.7 | 34.86 | 35.64 | 36.32 | 36.84 | 36.68 |
| SEM | 0.18 | 0.14 | 0.15 | 0.47 | 0.83 | 0.05 | 0.12 | 0.16 | 0.15 | 0.31 |
| µ-miR-19b-ND-RNA (n=5) | 13.64 | 14.5 | 16.68 | 19 | 22.32 | 24.2 | 27.5 | 29.62 | 31.36 | 31.48 |
| SEM | 0.2 | 0.13 | 0.21 | 0.38 | 0.44 | 0.22 | 0.46 | 0.17 | 0.36 | 0.28 |
| µ-HFD-ND-RNA (n=5) | 14.68 | 15.84 | 17.56 | 22.46 | 25.94 | 29.22 | 31.22 | 32 | 33.3 | 34.64 |
| SEM | 0.35 | 0.35 | 0.42 | 0.76 | 0.58 | 0.49 | 0.3 | 0.37 | 0.44 | 0.47 |
| µ-HFD-RNA (n=5) | 15.38 | 17.02 | 20.24 | 26.98 | 32.48 | 34.98 | 36.14 | 36.6 | 37.64 | 38.5 |
| SEM | 0.44 | 0.53 | 0.57 | 0.76 | 0.55 | 0.55 | 0.32 | 0.34 | 0.17 | 0.26 |
(A)
| | Blood glucose level (mg/dl) | | | | | |
| --- | --- | --- | --- | --- | --- | --- |
| C57BL/6 F0 groups | 0’ | 15’ | 30’ | 60’ | 90’ | 120’ |
| HFD (n=5) | 187.25 | 367 | 403.6 | 399.2 | 277.2 | 220.16 |
| SEM | 1.46 | 3.7 | 4.93 | 2.5 | 3.88 | 1.33 |
| ND (n=5) | 127.8 | 329 | 333.4 | 280.2 | 181.2 | 162.2 |
| SEM | 0.73 | 0.71 | 1.21 | 2.22 | 2.76 | 2.48 |
| µ-miR-19b (n=5) | 179.2 | 381.8 | 400.8 | 389.8 | 275.5 | 217.2 |
| SEM | 4.43 | 2.75 | 3.72 | 2.85 | 4.33 | 3.85 |
| µ-miR-19b-ND-RNA (n=5) | 128.6 | 337.4 | 338.4 | 290.2 | 184.2 | 164.8 |
| SEM | 1.5 | 2.38 | 1.86 | 3.4 | 2.31 | 2.15 |
| µ-HFD-ND-RNA (n=5) | 164 | 348.4 | 344.4 | 308 | 217.2 | 183.6 |
| SEM | 2.45 | 5.62 | 3.09 | 3.77 | 4.98 | 2.27 |
| µ-HFD-RNA (n=5) | 176 | 361 | 377.6 | 3819 | 269.5 | 206.8 |
| SEM | 2.05 | 3.78 | 10.2 | 2.61 | 2.48 | 2.87 |
| | Blood glucose level (mg/dl) | | | | |
| --- | --- | --- | --- | --- | --- |
| C57BL/6 F0 groups | 0’ | 15’ | 30’ | 60’ | 90’ |
| HFD (n=5) | 150.6 | 141 | 133.4 | 68.8 | 65.2 |
| SEM | 3.19 | 2.61 | 1.72 | 1.5 | 1.43 |
| ND (n=5) | 95.2 | 90.8 | 85 | 60 | 59.8 |
| SEM | 2.2 | 1.28 | 1.3 | 1 | 0.86 |
| µ-miR-19b (n=5) | 153.4 | 131.25 | 101.8 | 59.6 | 59.8 |
| SEM | 1.57 | 1.54 | 1.16 | 1.47 | 0.66 |
| µ-miR-19b-ND-RNA (n=5) | 104.6 | 89.6 | 68.4 | 60.75 | 65.4 |
| SEM | 2.29 | 2.46 | 1.78 | 1.08 | 0.93 |
| µ-HFD-ND-RNA (n=5) | 127.4 | 111.6 | 100 | 67 | 61.8 |
| SEM | 4.46 | 2.93 | 3.51 | 3.05 | 2.06 |
| µ-HFD-RNA (n=5) | 147.4 | 149.5 | 132.2 | 74 | 62.8 |
| SEM | 2.66 | 3.44 | 3.48 | 2.17 | 1.91 |
ITT (C)
GTT (B)

## Slide 8
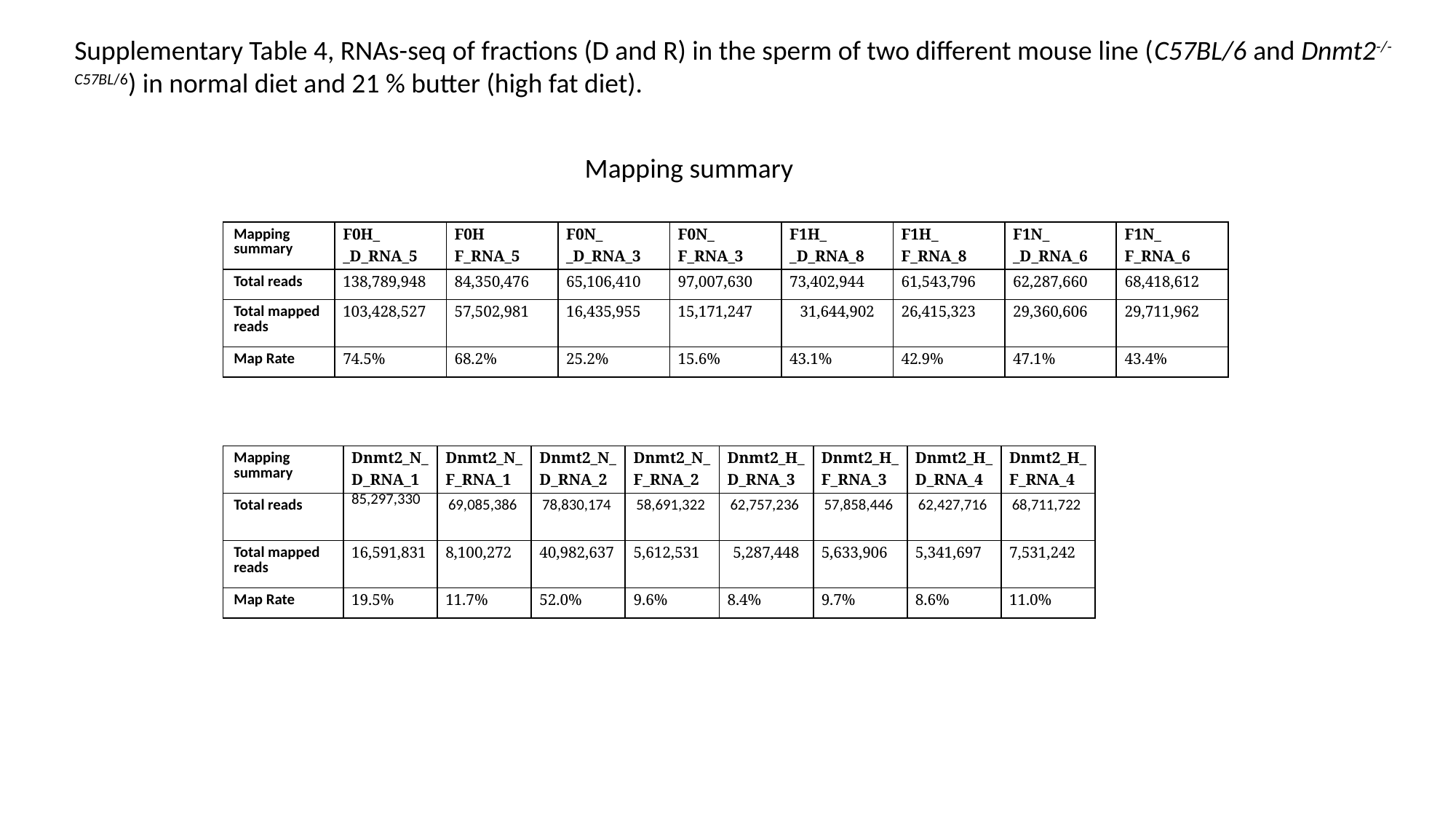

Supplementary Table 4, RNAs-seq of fractions (D and R) in the sperm of two different mouse line (C57BL/6 and Dnmt2-/-C57BL/6) in normal diet and 21 % butter (high fat diet).
Mapping summary
| Mapping summary | F0H\_ \_D\_RNA\_5 | F0H F\_RNA\_5 | F0N\_ \_D\_RNA\_3 | F0N\_ F\_RNA\_3 | F1H\_ \_D\_RNA\_8 | F1H\_ F\_RNA\_8 | F1N\_ \_D\_RNA\_6 | F1N\_ F\_RNA\_6 |
| --- | --- | --- | --- | --- | --- | --- | --- | --- |
| Total reads | 138,789,948 | 84,350,476 | 65,106,410 | 97,007,630 | 73,402,944 | 61,543,796 | 62,287,660 | 68,418,612 |
| Total mapped reads | 103,428,527 | 57,502,981 | 16,435,955 | 15,171,247 | 31,644,902 | 26,415,323 | 29,360,606 | 29,711,962 |
| Map Rate | 74.5% | 68.2% | 25.2% | 15.6% | 43.1% | 42.9% | 47.1% | 43.4% |
| Mapping summary | Dnmt2\_N\_D\_RNA\_1 | Dnmt2\_N\_F\_RNA\_1 | Dnmt2\_N\_D\_RNA\_2 | Dnmt2\_N\_F\_RNA\_2 | Dnmt2\_H\_D\_RNA\_3 | Dnmt2\_H\_F\_RNA\_3 | Dnmt2\_H\_D\_RNA\_4 | Dnmt2\_H\_F\_RNA\_4 |
| --- | --- | --- | --- | --- | --- | --- | --- | --- |
| Total reads | 85,297,330 | 69,085,386 | 78,830,174 | 58,691,322 | 62,757,236 | 57,858,446 | 62,427,716 | 68,711,722 |
| Total mapped reads | 16,591,831 | 8,100,272 | 40,982,637 | 5,612,531 | 5,287,448 | 5,633,906 | 5,341,697 | 7,531,242 |
| Map Rate | 19.5% | 11.7% | 52.0% | 9.6% | 8.4% | 9.7% | 8.6% | 11.0% |

## Slide 9
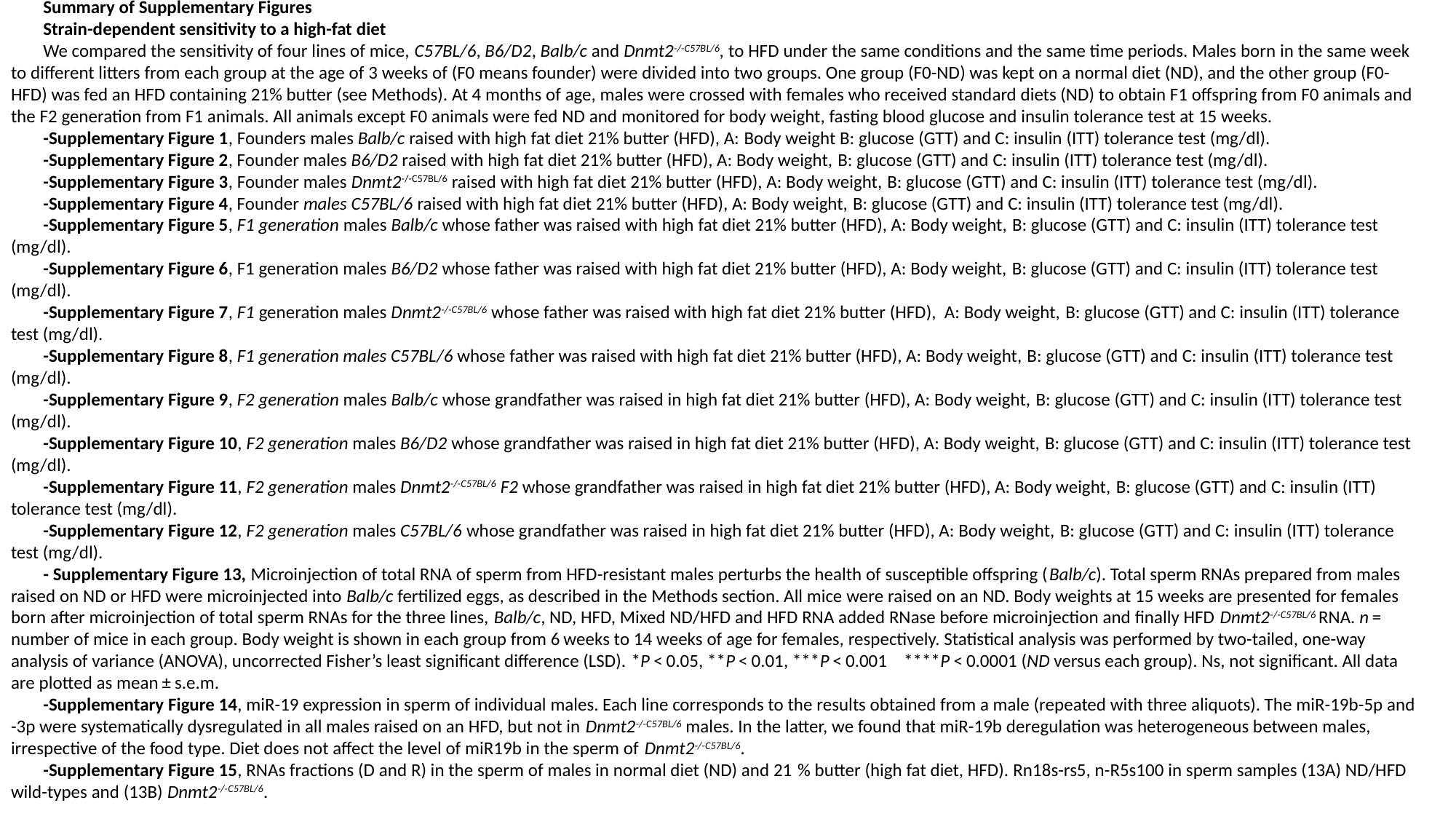

Summary of Supplementary Figures
Strain-dependent sensitivity to a high-fat diet
We compared the sensitivity of four lines of mice, C57BL/6, B6/D2, Balb/c and Dnmt2-/-C57BL/6, to HFD under the same conditions and the same time periods. Males born in the same week to different litters from each group at the age of 3 weeks of (F0 means founder) were divided into two groups. One group (F0-ND) was kept on a normal diet (ND), and the other group (F0-HFD) was fed an HFD containing 21% butter (see Methods). At 4 months of age, males were crossed with females who received standard diets (ND) to obtain F1 offspring from F0 animals and the F2 generation from F1 animals. All animals except F0 animals were fed ND and monitored for body weight, fasting blood glucose and insulin tolerance test at 15 weeks.
-Supplementary Figure 1, Founders males Balb/c raised with high fat diet 21% butter (HFD), A: Body weight B: glucose (GTT) and C: insulin (ITT) tolerance test (mg/dl).
-Supplementary Figure 2, Founder males B6/D2 raised with high fat diet 21% butter (HFD), A: Body weight, B: glucose (GTT) and C: insulin (ITT) tolerance test (mg/dl).
-Supplementary Figure 3, Founder males Dnmt2-/-C57BL/6 raised with high fat diet 21% butter (HFD), A: Body weight, B: glucose (GTT) and C: insulin (ITT) tolerance test (mg/dl).
-Supplementary Figure 4, Founder males C57BL/6 raised with high fat diet 21% butter (HFD), A: Body weight, B: glucose (GTT) and C: insulin (ITT) tolerance test (mg/dl).
-Supplementary Figure 5, F1 generation males Balb/c whose father was raised with high fat diet 21% butter (HFD), A: Body weight, B: glucose (GTT) and C: insulin (ITT) tolerance test (mg/dl).
-Supplementary Figure 6, F1 generation males B6/D2 whose father was raised with high fat diet 21% butter (HFD), A: Body weight, B: glucose (GTT) and C: insulin (ITT) tolerance test (mg/dl).
-Supplementary Figure 7, F1 generation males Dnmt2-/-C57BL/6 whose father was raised with high fat diet 21% butter (HFD), A: Body weight, B: glucose (GTT) and C: insulin (ITT) tolerance test (mg/dl).
-Supplementary Figure 8, F1 generation males C57BL/6 whose father was raised with high fat diet 21% butter (HFD), A: Body weight, B: glucose (GTT) and C: insulin (ITT) tolerance test (mg/dl).
-Supplementary Figure 9, F2 generation males Balb/c whose grandfather was raised in high fat diet 21% butter (HFD), A: Body weight, B: glucose (GTT) and C: insulin (ITT) tolerance test (mg/dl).
-Supplementary Figure 10, F2 generation males B6/D2 whose grandfather was raised in high fat diet 21% butter (HFD), A: Body weight, B: glucose (GTT) and C: insulin (ITT) tolerance test (mg/dl).
-Supplementary Figure 11, F2 generation males Dnmt2-/-C57BL/6 F2 whose grandfather was raised in high fat diet 21% butter (HFD), A: Body weight, B: glucose (GTT) and C: insulin (ITT) tolerance test (mg/dl).
-Supplementary Figure 12, F2 generation males C57BL/6 whose grandfather was raised in high fat diet 21% butter (HFD), A: Body weight, B: glucose (GTT) and C: insulin (ITT) tolerance test (mg/dl).
- Supplementary Figure 13, Microinjection of total RNA of sperm from HFD-resistant males perturbs the health of susceptible offspring (Balb/c). Total sperm RNAs prepared from males raised on ND or HFD were microinjected into Balb/c fertilized eggs, as described in the Methods section. All mice were raised on an ND. Body weights at 15 weeks are presented for females born after microinjection of total sperm RNAs for the three lines, Balb/c, ND, HFD, Mixed ND/HFD and HFD RNA added RNase before microinjection and finally HFD Dnmt2-/-C57BL/6 RNA. n = number of mice in each group. Body weight is shown in each group from 6 weeks to 14 weeks of age for females, respectively. Statistical analysis was performed by two-tailed, one-way analysis of variance (ANOVA), uncorrected Fisher’s least significant difference (LSD). *P < 0.05, **P < 0.01, ***P < 0.001 ****P < 0.0001 (ND versus each group). Ns, not significant. All data are plotted as mean ± s.e.m.
-Supplementary Figure 14, miR-19 expression in sperm of individual males. Each line corresponds to the results obtained from a male (repeated with three aliquots). The miR-19b-5p and -3p were systematically dysregulated in all males raised on an HFD, but not in Dnmt2-/-C57BL/6 males. In the latter, we found that miR-19b deregulation was heterogeneous between males, irrespective of the food type. Diet does not affect the level of miR19b in the sperm of Dnmt2-/-C57BL/6.
-Supplementary Figure 15, RNAs fractions (D and R) in the sperm of males in normal diet (ND) and 21 % butter (high fat diet, HFD). Rn18s-rs5, n-R5s100 in sperm samples (13A) ND/HFD wild-types and (13B) Dnmt2-/-C57BL/6.

## Slide 10
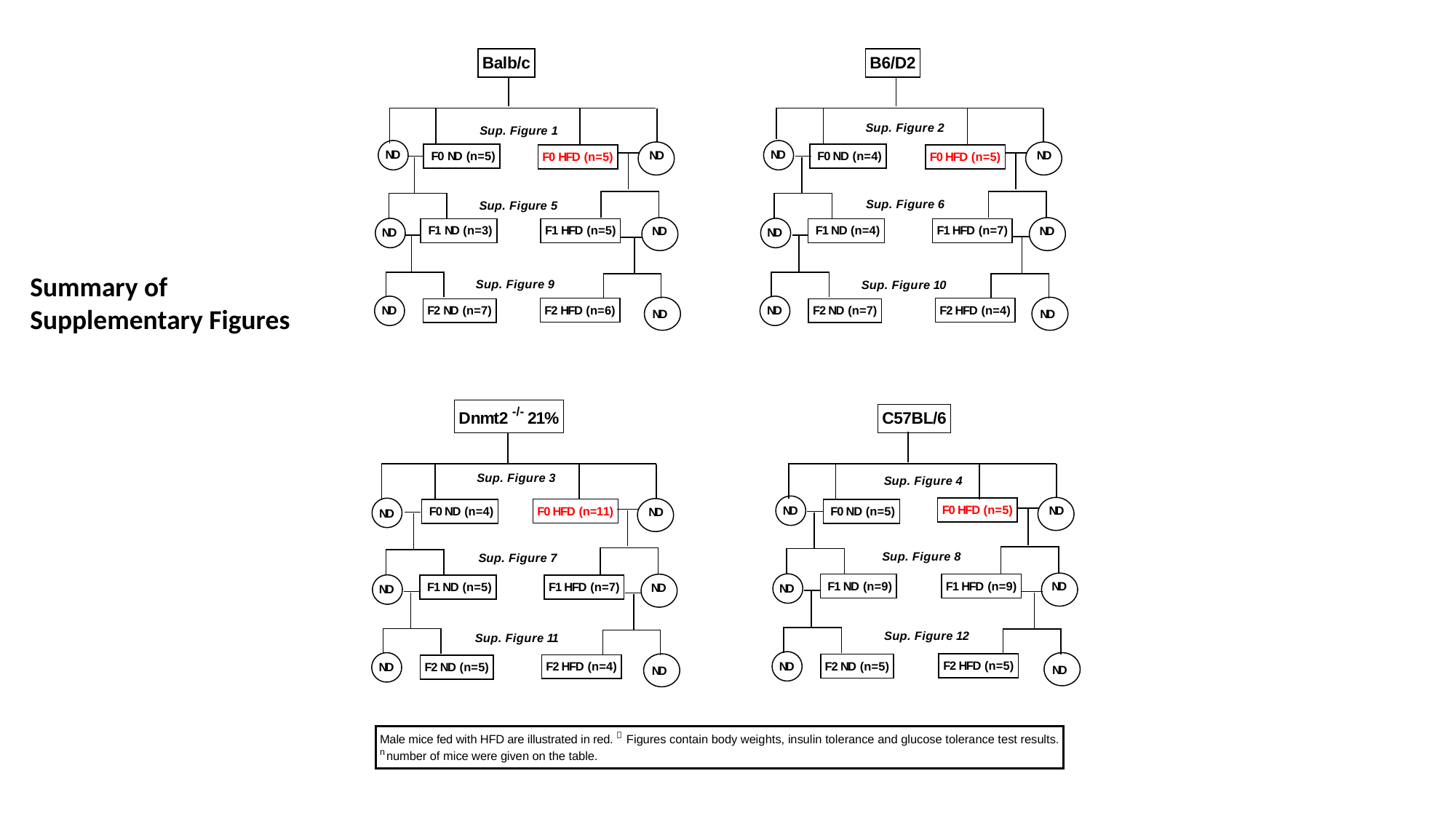

Summary of
Supplementary Figures

## Slide 11
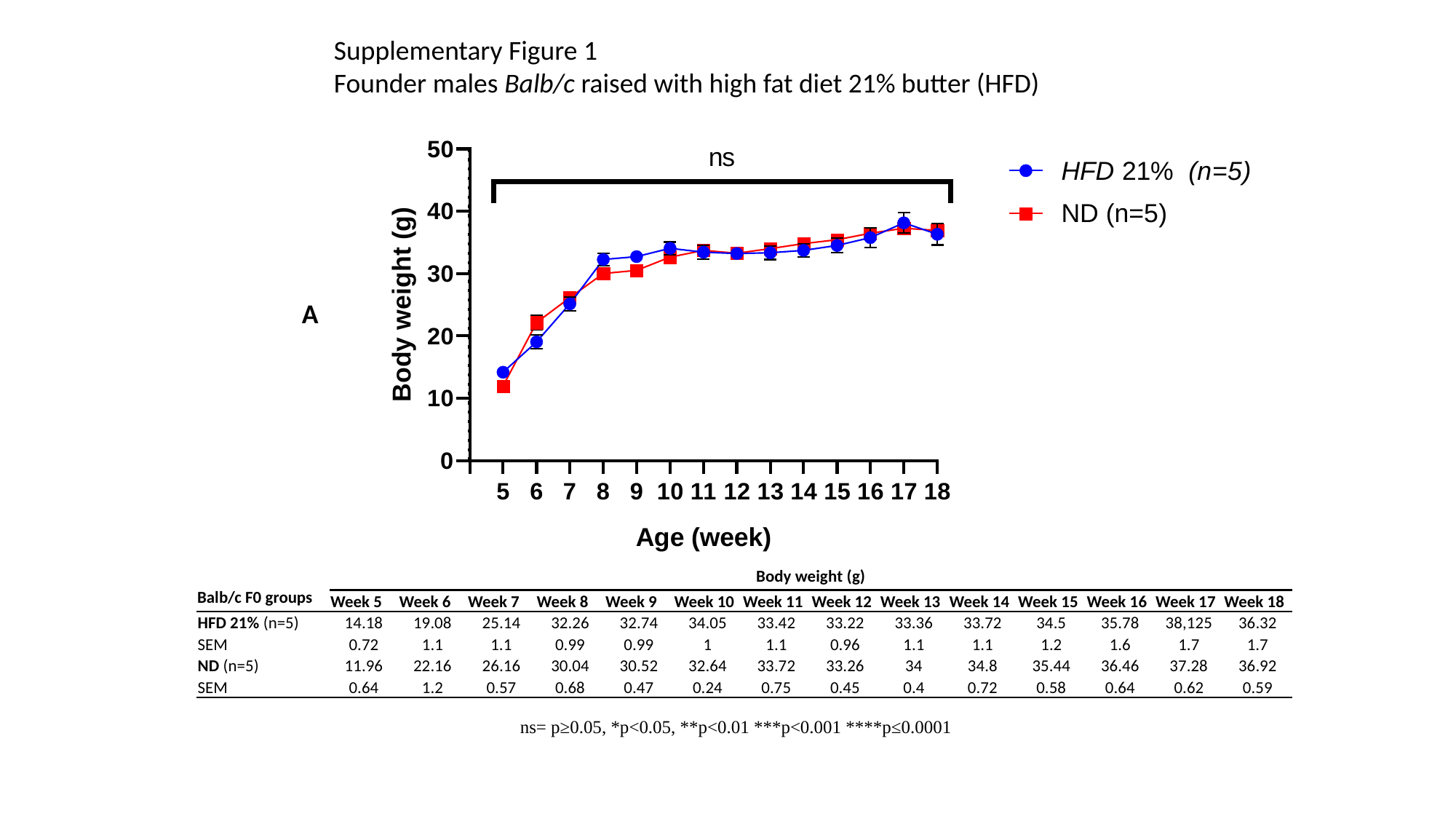

Supplementary Figure 1
Founder males Balb/c raised with high fat diet 21% butter (HFD)
A
| | Body weight (g) | | | | | | | | | | | | | |
| --- | --- | --- | --- | --- | --- | --- | --- | --- | --- | --- | --- | --- | --- | --- |
| Balb/c F0 groups | Week 5 | Week 6 | Week 7 | Week 8 | Week 9 | Week 10 | Week 11 | Week 12 | Week 13 | Week 14 | Week 15 | Week 16 | Week 17 | Week 18 |
| HFD 21% (n=5) | 14.18 | 19.08 | 25.14 | 32.26 | 32.74 | 34.05 | 33.42 | 33.22 | 33.36 | 33.72 | 34.5 | 35.78 | 38,125 | 36.32 |
| SEM | 0.72 | 1.1 | 1.1 | 0.99 | 0.99 | 1 | 1.1 | 0.96 | 1.1 | 1.1 | 1.2 | 1.6 | 1.7 | 1.7 |
| ND (n=5) | 11.96 | 22.16 | 26.16 | 30.04 | 30.52 | 32.64 | 33.72 | 33.26 | 34 | 34.8 | 35.44 | 36.46 | 37.28 | 36.92 |
| SEM | 0.64 | 1.2 | 0.57 | 0.68 | 0.47 | 0.24 | 0.75 | 0.45 | 0.4 | 0.72 | 0.58 | 0.64 | 0.62 | 0.59 |
ns= p≥0.05, *p<0.05, **p<0.01 ***p<0.001 ****p≤0.0001

## Slide 12
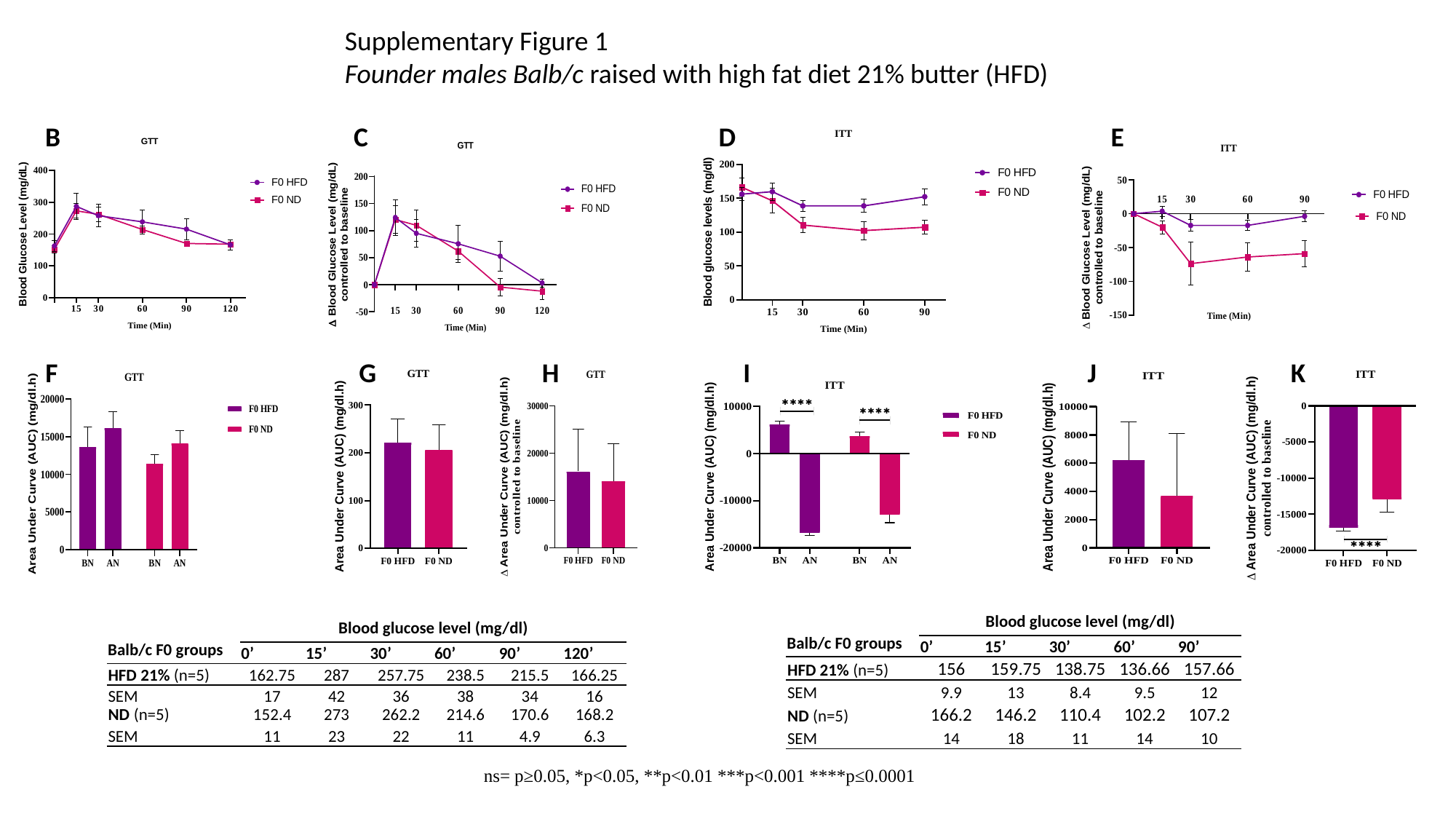

Supplementary Figure 1
Founder males Balb/c raised with high fat diet 21% butter (HFD)
B
C
D
E
F
G
H
I
J
K
| | Blood glucose level (mg/dl) | | | | |
| --- | --- | --- | --- | --- | --- |
| Balb/c F0 groups | 0’ | 15’ | 30’ | 60’ | 90’ |
| HFD 21% (n=5) | 156 | 159.75 | 138.75 | 136.66 | 157.66 |
| SEM | 9.9 | 13 | 8.4 | 9.5 | 12 |
| ND (n=5) | 166.2 | 146.2 | 110.4 | 102.2 | 107.2 |
| SEM | 14 | 18 | 11 | 14 | 10 |
| | Blood glucose level (mg/dl) | | | | | |
| --- | --- | --- | --- | --- | --- | --- |
| Balb/c F0 groups | 0’ | 15’ | 30’ | 60’ | 90’ | 120’ |
| HFD 21% (n=5) | 162.75 | 287 | 257.75 | 238.5 | 215.5 | 166.25 |
| SEM | 17 | 42 | 36 | 38 | 34 | 16 |
| ND (n=5) | 152.4 | 273 | 262.2 | 214.6 | 170.6 | 168.2 |
| SEM | 11 | 23 | 22 | 11 | 4.9 | 6.3 |
ns= p≥0.05, *p<0.05, **p<0.01 ***p<0.001 ****p≤0.0001

## Slide 13
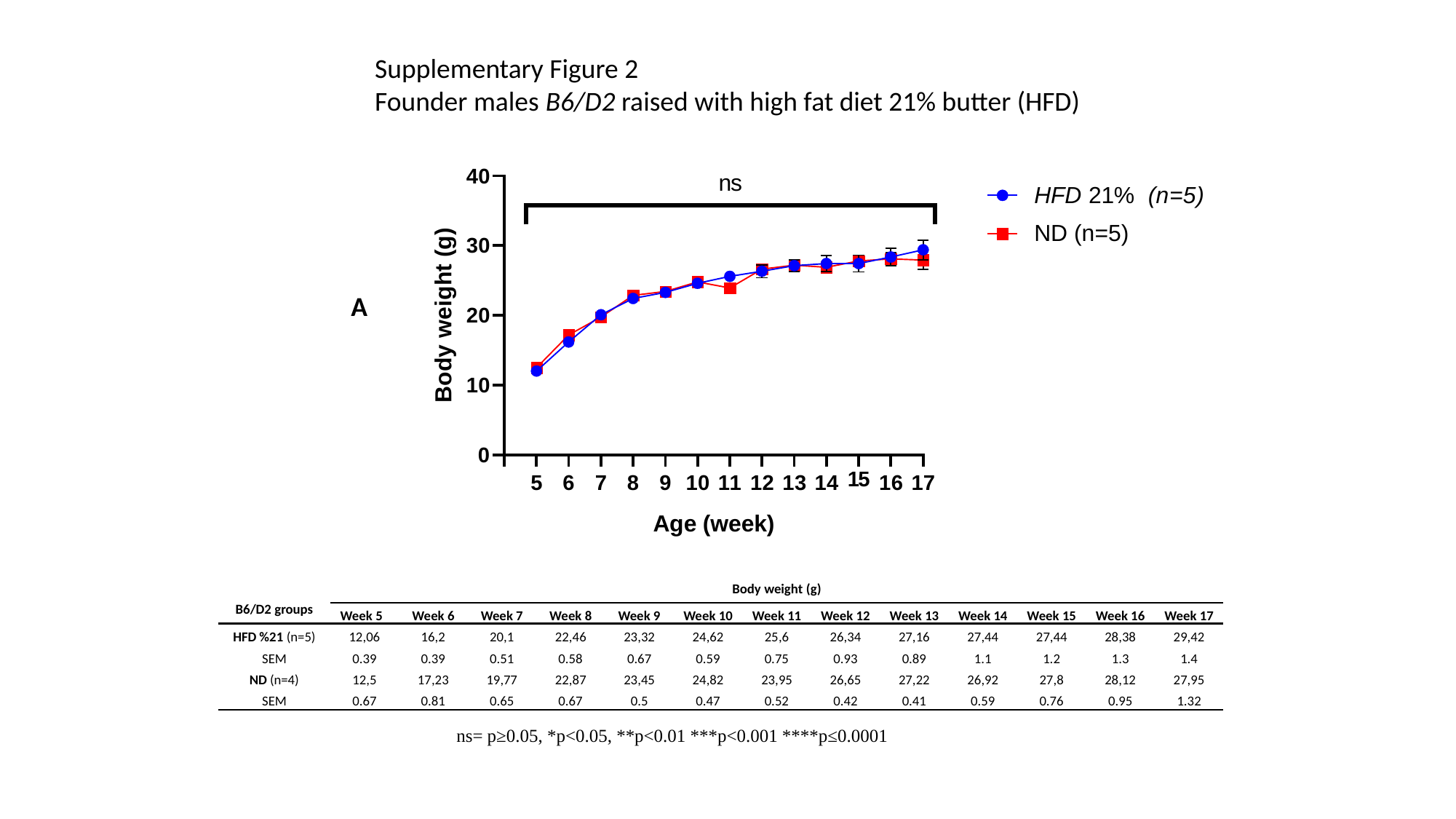

Supplementary Figure 2
Founder males B6/D2 raised with high fat diet 21% butter (HFD)
A
| | Body weight (g) | | | | | | | | | | | | |
| --- | --- | --- | --- | --- | --- | --- | --- | --- | --- | --- | --- | --- | --- |
| B6/D2 groups | Week 5 | Week 6 | Week 7 | Week 8 | Week 9 | Week 10 | Week 11 | Week 12 | Week 13 | Week 14 | Week 15 | Week 16 | Week 17 |
| HFD %21 (n=5) | 12,06 | 16,2 | 20,1 | 22,46 | 23,32 | 24,62 | 25,6 | 26,34 | 27,16 | 27,44 | 27,44 | 28,38 | 29,42 |
| SEM | 0.39 | 0.39 | 0.51 | 0.58 | 0.67 | 0.59 | 0.75 | 0.93 | 0.89 | 1.1 | 1.2 | 1.3 | 1.4 |
| ND (n=4) | 12,5 | 17,23 | 19,77 | 22,87 | 23,45 | 24,82 | 23,95 | 26,65 | 27,22 | 26,92 | 27,8 | 28,12 | 27,95 |
| SEM | 0.67 | 0.81 | 0.65 | 0.67 | 0.5 | 0.47 | 0.52 | 0.42 | 0.41 | 0.59 | 0.76 | 0.95 | 1.32 |
ns= p≥0.05, *p<0.05, **p<0.01 ***p<0.001 ****p≤0.0001

## Slide 14
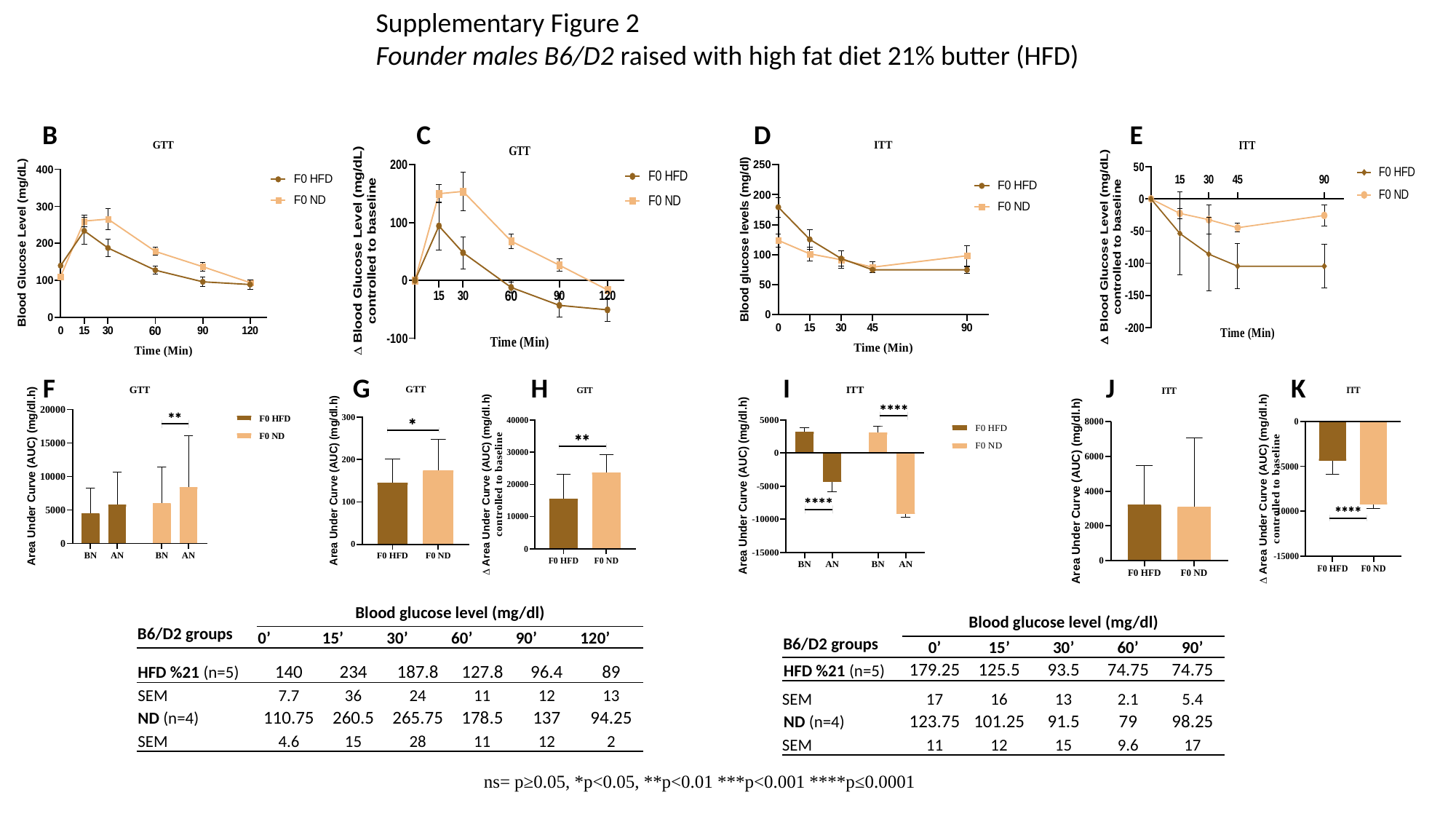

Supplementary Figure 2
Founder males B6/D2 raised with high fat diet 21% butter (HFD)
B
C
D
E
F
G
H
I
J
K
| | Blood glucose level (mg/dl) | | | | | |
| --- | --- | --- | --- | --- | --- | --- |
| B6/D2 groups | 0’ | 15’ | 30’ | 60’ | 90’ | 120’ |
| HFD %21 (n=5) | 140 | 234 | 187.8 | 127.8 | 96.4 | 89 |
| SEM | 7.7 | 36 | 24 | 11 | 12 | 13 |
| ND (n=4) | 110.75 | 260.5 | 265.75 | 178.5 | 137 | 94.25 |
| SEM | 4.6 | 15 | 28 | 11 | 12 | 2 |
| | Blood glucose level (mg/dl) | | | | |
| --- | --- | --- | --- | --- | --- |
| B6/D2 groups | 0’ | 15’ | 30’ | 60’ | 90’ |
| HFD %21 (n=5) | 179.25 | 125.5 | 93.5 | 74.75 | 74.75 |
| SEM | 17 | 16 | 13 | 2.1 | 5.4 |
| ND (n=4) | 123.75 | 101.25 | 91.5 | 79 | 98.25 |
| SEM | 11 | 12 | 15 | 9.6 | 17 |
ns= p≥0.05, *p<0.05, **p<0.01 ***p<0.001 ****p≤0.0001

## Slide 15
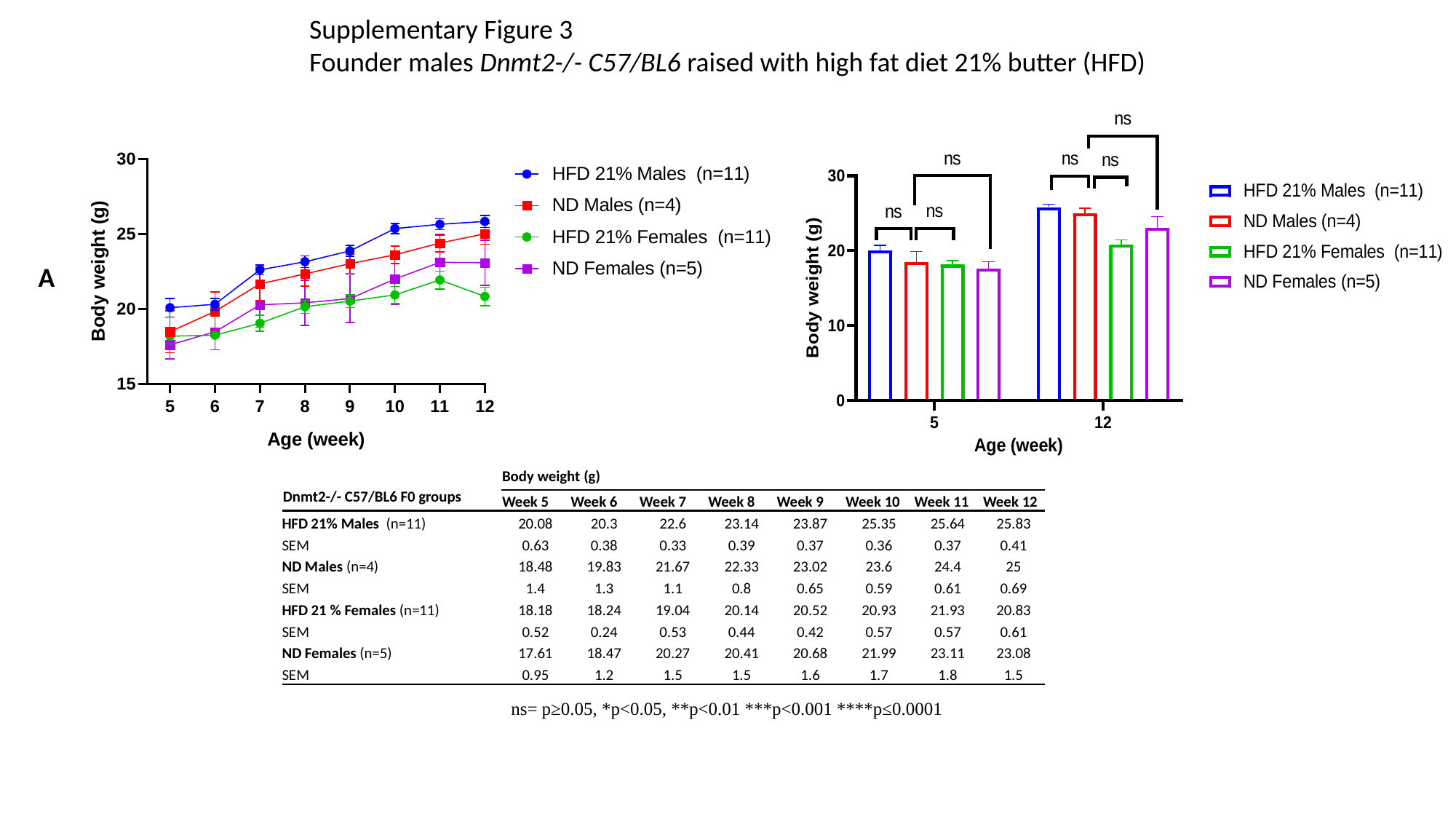

Supplementary Figure 3
Founder males Dnmt2-/- C57/BL6 raised with high fat diet 21% butter (HFD)
A
| | Body weight (g) | | | | | | | |
| --- | --- | --- | --- | --- | --- | --- | --- | --- |
| Dnmt2-/- C57/BL6 F0 groups | Week 5 | Week 6 | Week 7 | Week 8 | Week 9 | Week 10 | Week 11 | Week 12 |
| HFD 21% Males (n=11) | 20.08 | 20.3 | 22.6 | 23.14 | 23.87 | 25.35 | 25.64 | 25.83 |
| SEM | 0.63 | 0.38 | 0.33 | 0.39 | 0.37 | 0.36 | 0.37 | 0.41 |
| ND Males (n=4) | 18.48 | 19.83 | 21.67 | 22.33 | 23.02 | 23.6 | 24.4 | 25 |
| SEM | 1.4 | 1.3 | 1.1 | 0.8 | 0.65 | 0.59 | 0.61 | 0.69 |
| HFD 21 % Females (n=11) | 18.18 | 18.24 | 19.04 | 20.14 | 20.52 | 20.93 | 21.93 | 20.83 |
| SEM | 0.52 | 0.24 | 0.53 | 0.44 | 0.42 | 0.57 | 0.57 | 0.61 |
| ND Females (n=5) | 17.61 | 18.47 | 20.27 | 20.41 | 20.68 | 21.99 | 23.11 | 23.08 |
| SEM | 0.95 | 1.2 | 1.5 | 1.5 | 1.6 | 1.7 | 1.8 | 1.5 |
ns= p≥0.05, *p<0.05, **p<0.01 ***p<0.001 ****p≤0.0001

## Slide 16
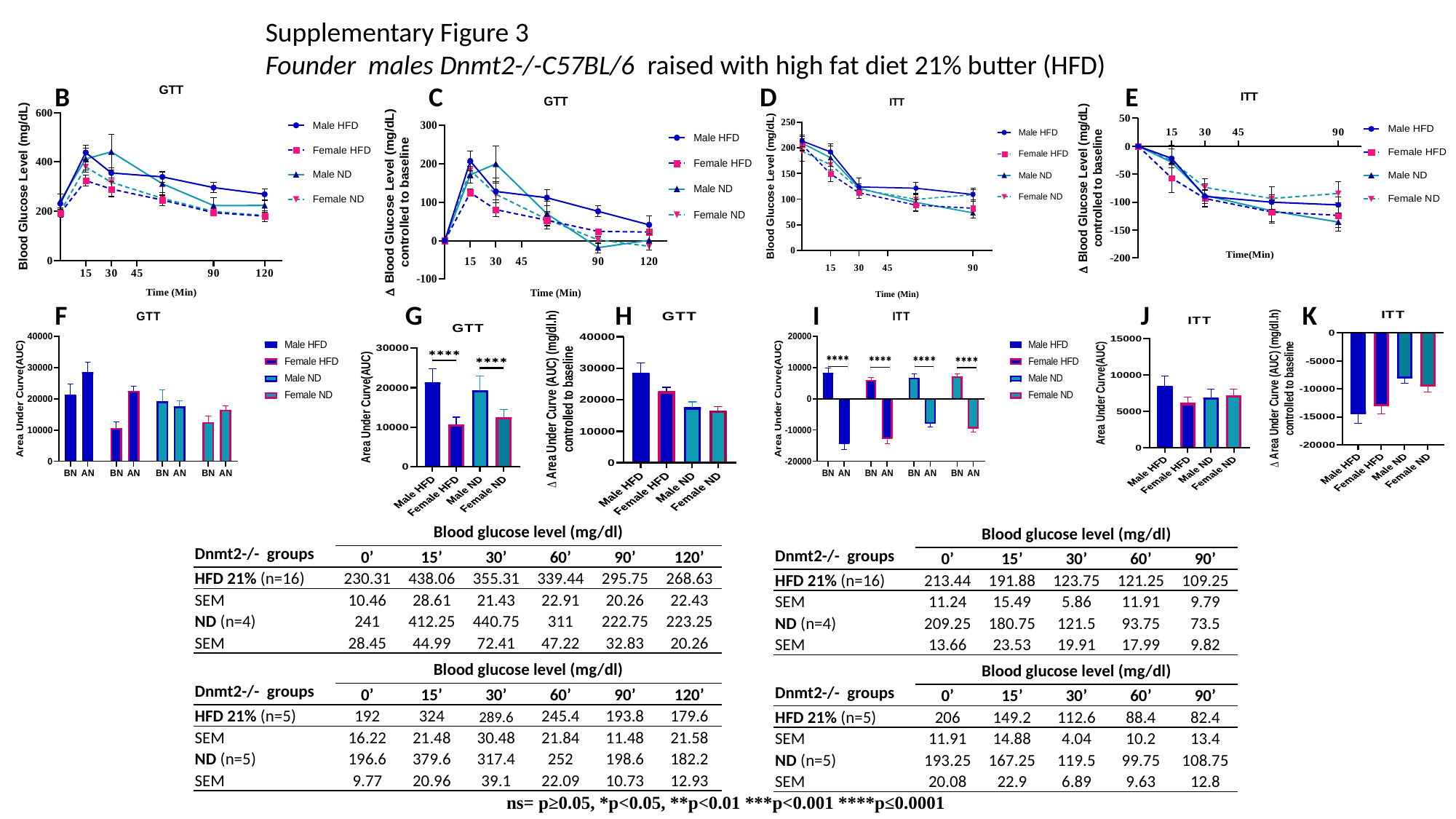

Supplementary Figure 3
Founder males Dnmt2-/-C57BL/6 raised with high fat diet 21% butter (HFD)
B
C
D
E
F
G
H
I
J
K
| | Blood glucose level (mg/dl) | | | | | |
| --- | --- | --- | --- | --- | --- | --- |
| Dnmt2-/- groups | 0’ | 15’ | 30’ | 60’ | 90’ | 120’ |
| HFD 21% (n=16) | 230.31 | 438.06 | 355.31 | 339.44 | 295.75 | 268.63 |
| SEM | 10.46 | 28.61 | 21.43 | 22.91 | 20.26 | 22.43 |
| ND (n=4) | 241 | 412.25 | 440.75 | 311 | 222.75 | 223.25 |
| SEM | 28.45 | 44.99 | 72.41 | 47.22 | 32.83 | 20.26 |
| | Blood glucose level (mg/dl) | | | | |
| --- | --- | --- | --- | --- | --- |
| Dnmt2-/- groups | 0’ | 15’ | 30’ | 60’ | 90’ |
| HFD 21% (n=16) | 213.44 | 191.88 | 123.75 | 121.25 | 109.25 |
| SEM | 11.24 | 15.49 | 5.86 | 11.91 | 9.79 |
| ND (n=4) | 209.25 | 180.75 | 121.5 | 93.75 | 73.5 |
| SEM | 13.66 | 23.53 | 19.91 | 17.99 | 9.82 |
| | Blood glucose level (mg/dl) | | | | | |
| --- | --- | --- | --- | --- | --- | --- |
| Dnmt2-/- groups | 0’ | 15’ | 30’ | 60’ | 90’ | 120’ |
| HFD 21% (n=5) | 192 | 324 | 289.6 | 245.4 | 193.8 | 179.6 |
| SEM | 16.22 | 21.48 | 30.48 | 21.84 | 11.48 | 21.58 |
| ND (n=5) | 196.6 | 379.6 | 317.4 | 252 | 198.6 | 182.2 |
| SEM | 9.77 | 20.96 | 39.1 | 22.09 | 10.73 | 12.93 |
| | Blood glucose level (mg/dl) | | | | |
| --- | --- | --- | --- | --- | --- |
| Dnmt2-/- groups | 0’ | 15’ | 30’ | 60’ | 90’ |
| HFD 21% (n=5) | 206 | 149.2 | 112.6 | 88.4 | 82.4 |
| SEM | 11.91 | 14.88 | 4.04 | 10.2 | 13.4 |
| ND (n=5) | 193.25 | 167.25 | 119.5 | 99.75 | 108.75 |
| SEM | 20.08 | 22.9 | 6.89 | 9.63 | 12.8 |
ns= p≥0.05, *p<0.05, **p<0.01 ***p<0.001 ****p≤0.0001

## Slide 17
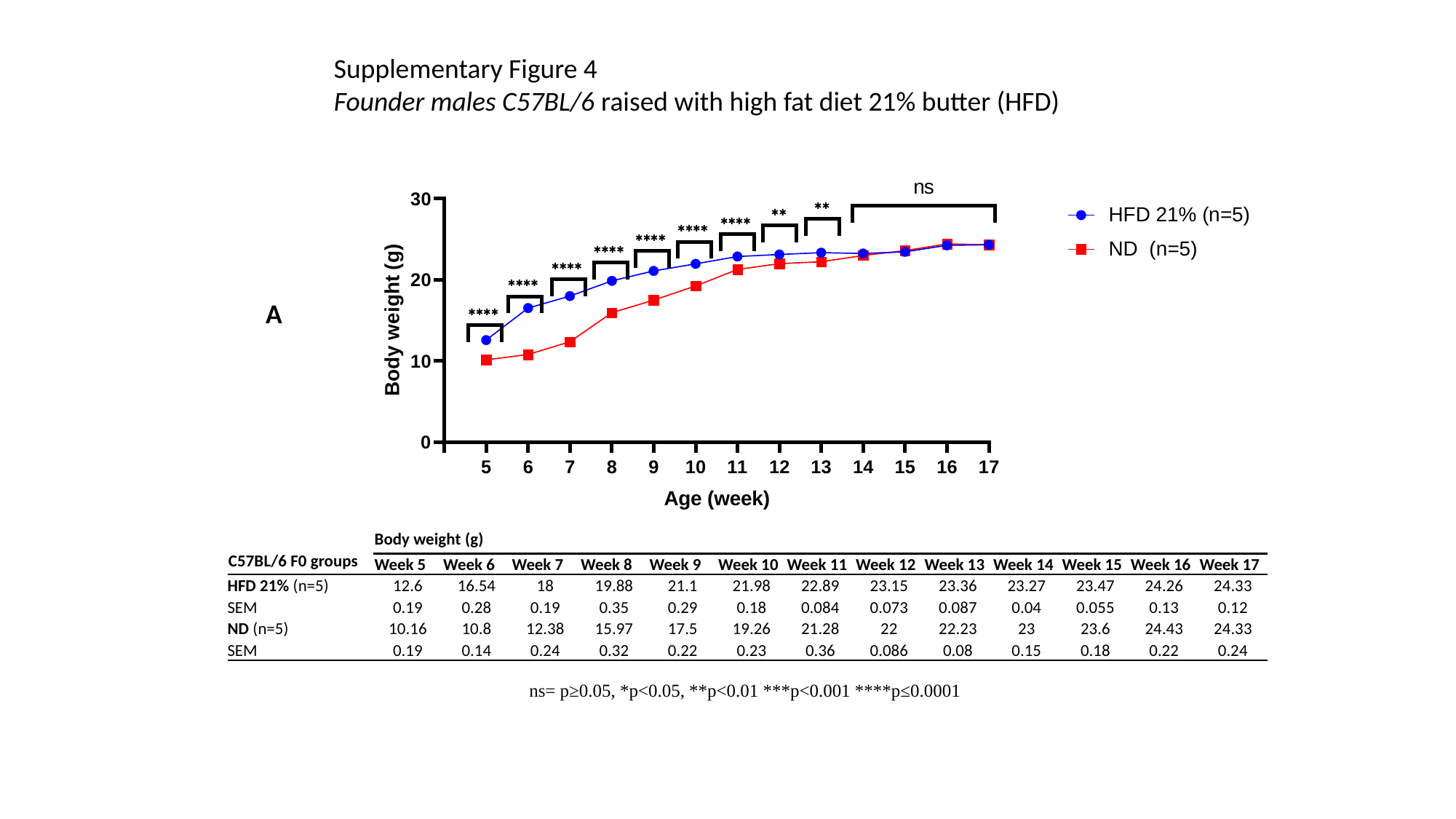

Supplementary Figure 4
Founder males C57BL/6 raised with high fat diet 21% butter (HFD)
A
| | Body weight (g) | | | | | | | | | | | | |
| --- | --- | --- | --- | --- | --- | --- | --- | --- | --- | --- | --- | --- | --- |
| C57BL/6 F0 groups | Week 5 | Week 6 | Week 7 | Week 8 | Week 9 | Week 10 | Week 11 | Week 12 | Week 13 | Week 14 | Week 15 | Week 16 | Week 17 |
| HFD 21% (n=5) | 12.6 | 16.54 | 18 | 19.88 | 21.1 | 21.98 | 22.89 | 23.15 | 23.36 | 23.27 | 23.47 | 24.26 | 24.33 |
| SEM | 0.19 | 0.28 | 0.19 | 0.35 | 0.29 | 0.18 | 0.084 | 0.073 | 0.087 | 0.04 | 0.055 | 0.13 | 0.12 |
| ND (n=5) | 10.16 | 10.8 | 12.38 | 15.97 | 17.5 | 19.26 | 21.28 | 22 | 22.23 | 23 | 23.6 | 24.43 | 24.33 |
| SEM | 0.19 | 0.14 | 0.24 | 0.32 | 0.22 | 0.23 | 0.36 | 0.086 | 0.08 | 0.15 | 0.18 | 0.22 | 0.24 |
ns= p≥0.05, *p<0.05, **p<0.01 ***p<0.001 ****p≤0.0001

## Slide 18
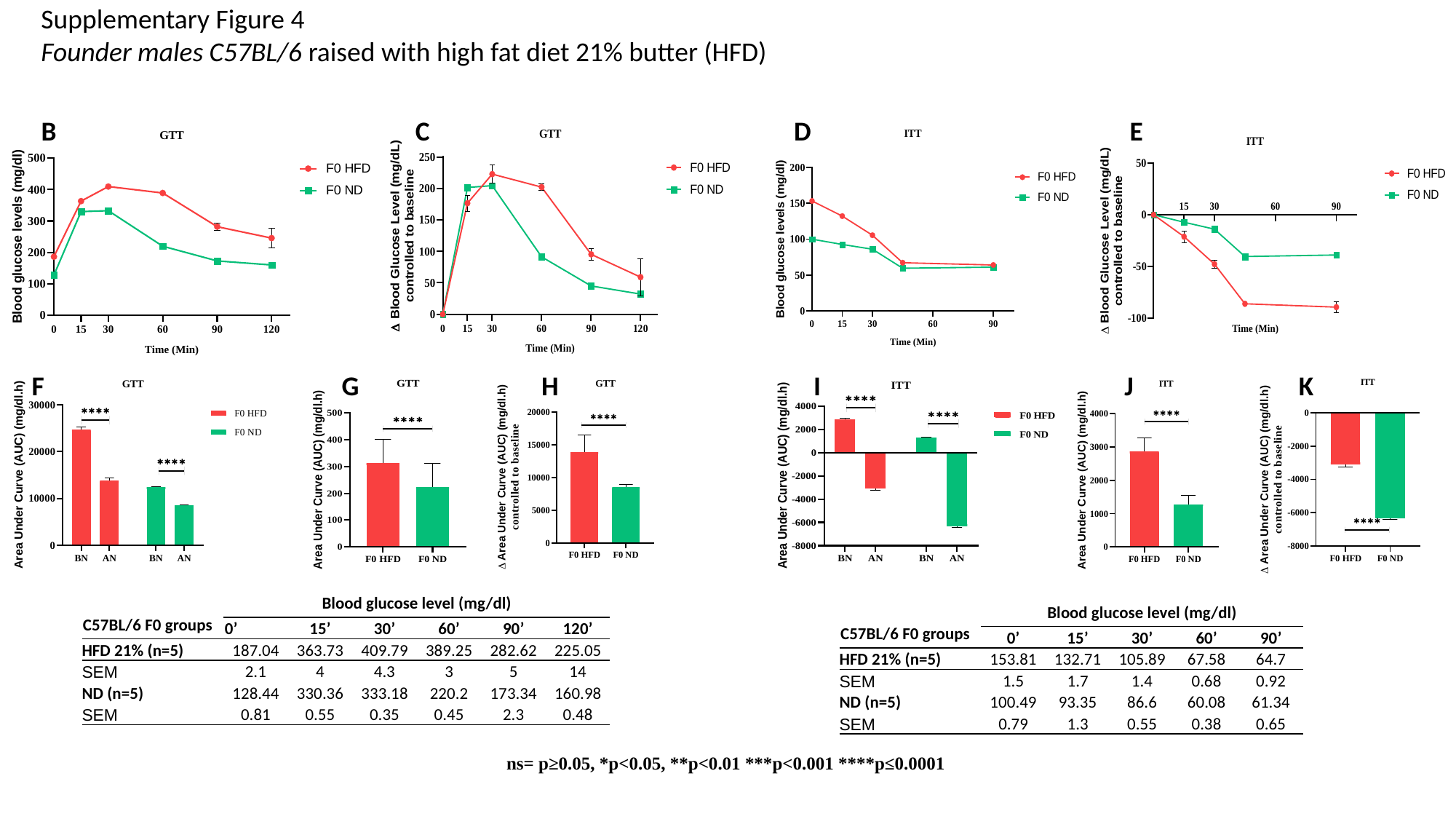

Supplementary Figure 4
Founder males C57BL/6 raised with high fat diet 21% butter (HFD)
B
C
D
E
F
G
H
I
J
K
| | Blood glucose level (mg/dl) | | | | | |
| --- | --- | --- | --- | --- | --- | --- |
| C57BL/6 F0 groups | 0’ | 15’ | 30’ | 60’ | 90’ | 120’ |
| HFD 21% (n=5) | 187.04 | 363.73 | 409.79 | 389.25 | 282.62 | 225.05 |
| SEM | 2.1 | 4 | 4.3 | 3 | 5 | 14 |
| ND (n=5) | 128.44 | 330.36 | 333.18 | 220.2 | 173.34 | 160.98 |
| SEM | 0.81 | 0.55 | 0.35 | 0.45 | 2.3 | 0.48 |
| | Blood glucose level (mg/dl) | | | | |
| --- | --- | --- | --- | --- | --- |
| C57BL/6 F0 groups | 0’ | 15’ | 30’ | 60’ | 90’ |
| HFD 21% (n=5) | 153.81 | 132.71 | 105.89 | 67.58 | 64.7 |
| SEM | 1.5 | 1.7 | 1.4 | 0.68 | 0.92 |
| ND (n=5) | 100.49 | 93.35 | 86.6 | 60.08 | 61.34 |
| SEM | 0.79 | 1.3 | 0.55 | 0.38 | 0.65 |
ns= p≥0.05, *p<0.05, **p<0.01 ***p<0.001 ****p≤0.0001

## Slide 19
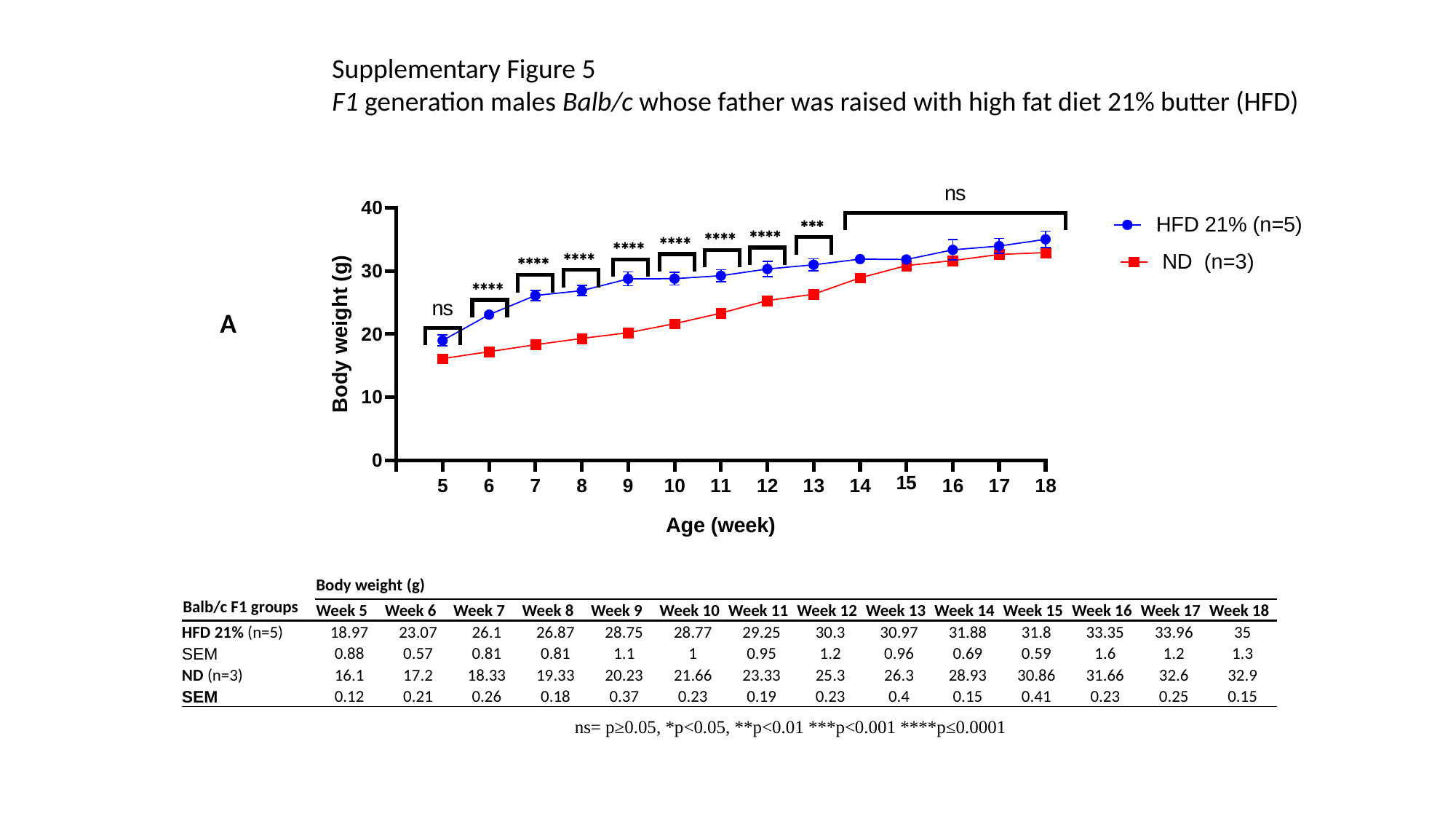

Supplementary Figure 5
F1 generation males Balb/c whose father was raised with high fat diet 21% butter (HFD)
A
| | Body weight (g) | | | | | | | | | | | | | |
| --- | --- | --- | --- | --- | --- | --- | --- | --- | --- | --- | --- | --- | --- | --- |
| Balb/c F1 groups | Week 5 | Week 6 | Week 7 | Week 8 | Week 9 | Week 10 | Week 11 | Week 12 | Week 13 | Week 14 | Week 15 | Week 16 | Week 17 | Week 18 |
| HFD 21% (n=5) | 18.97 | 23.07 | 26.1 | 26.87 | 28.75 | 28.77 | 29.25 | 30.3 | 30.97 | 31.88 | 31.8 | 33.35 | 33.96 | 35 |
| SEM | 0.88 | 0.57 | 0.81 | 0.81 | 1.1 | 1 | 0.95 | 1.2 | 0.96 | 0.69 | 0.59 | 1.6 | 1.2 | 1.3 |
| ND (n=3) | 16.1 | 17.2 | 18.33 | 19.33 | 20.23 | 21.66 | 23.33 | 25.3 | 26.3 | 28.93 | 30.86 | 31.66 | 32.6 | 32.9 |
| SEM | 0.12 | 0.21 | 0.26 | 0.18 | 0.37 | 0.23 | 0.19 | 0.23 | 0.4 | 0.15 | 0.41 | 0.23 | 0.25 | 0.15 |
ns= p≥0.05, *p<0.05, **p<0.01 ***p<0.001 ****p≤0.0001

## Slide 20
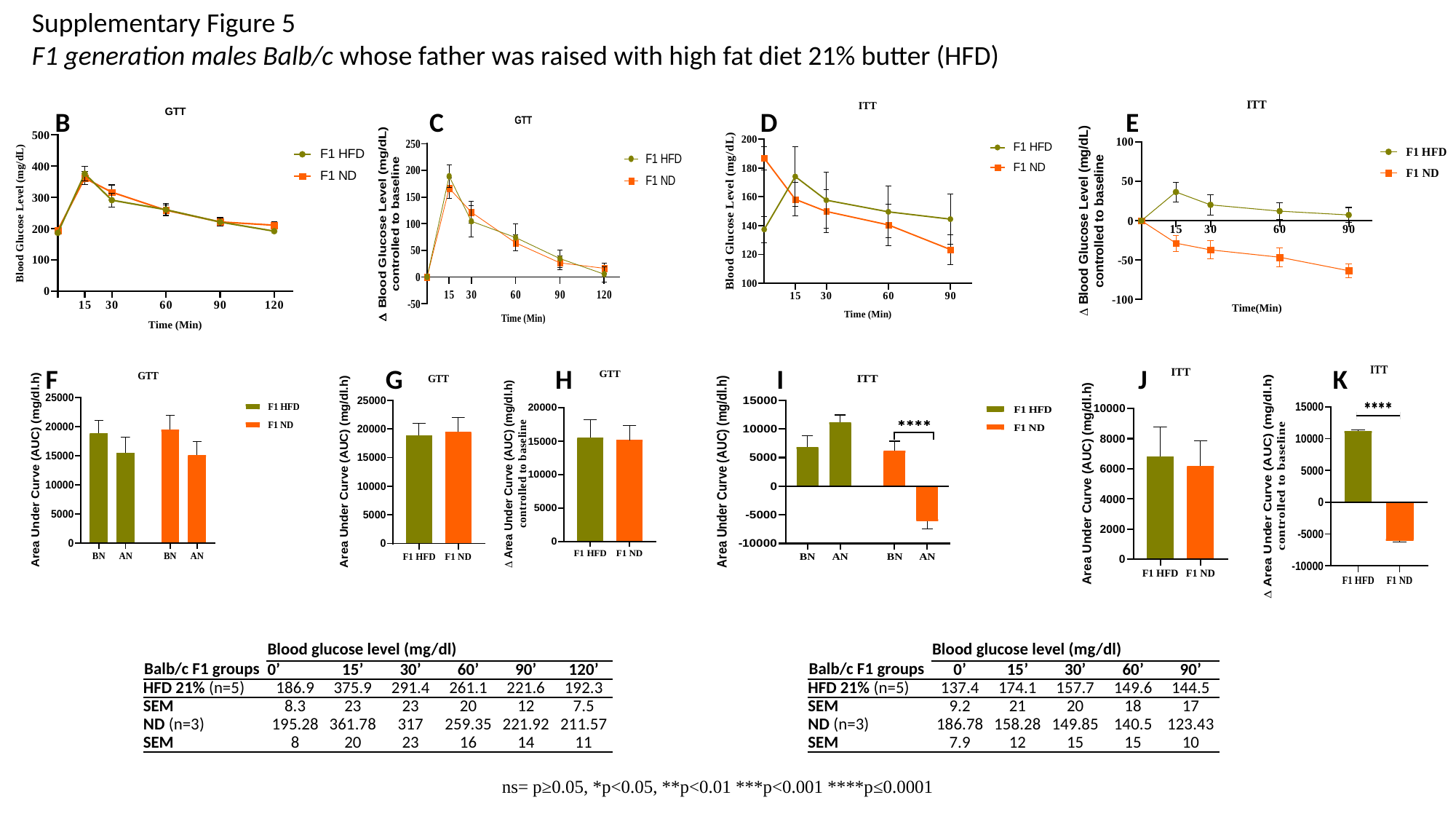

Supplementary Figure 5
F1 generation males Balb/c whose father was raised with high fat diet 21% butter (HFD)
B
C
D
E
F
G
H
I
J
K
| | Blood glucose level (mg/dl) | | | | | |
| --- | --- | --- | --- | --- | --- | --- |
| Balb/c F1 groups | 0’ | 15’ | 30’ | 60’ | 90’ | 120’ |
| HFD 21% (n=5) | 186.9 | 375.9 | 291.4 | 261.1 | 221.6 | 192.3 |
| SEM | 8.3 | 23 | 23 | 20 | 12 | 7.5 |
| ND (n=3) | 195.28 | 361.78 | 317 | 259.35 | 221.92 | 211.57 |
| SEM | 8 | 20 | 23 | 16 | 14 | 11 |
| | Blood glucose level (mg/dl) | | | | |
| --- | --- | --- | --- | --- | --- |
| Balb/c F1 groups | 0’ | 15’ | 30’ | 60’ | 90’ |
| HFD 21% (n=5) | 137.4 | 174.1 | 157.7 | 149.6 | 144.5 |
| SEM | 9.2 | 21 | 20 | 18 | 17 |
| ND (n=3) | 186.78 | 158.28 | 149.85 | 140.5 | 123.43 |
| SEM | 7.9 | 12 | 15 | 15 | 10 |
ns= p≥0.05, *p<0.05, **p<0.01 ***p<0.001 ****p≤0.0001

## Slide 21
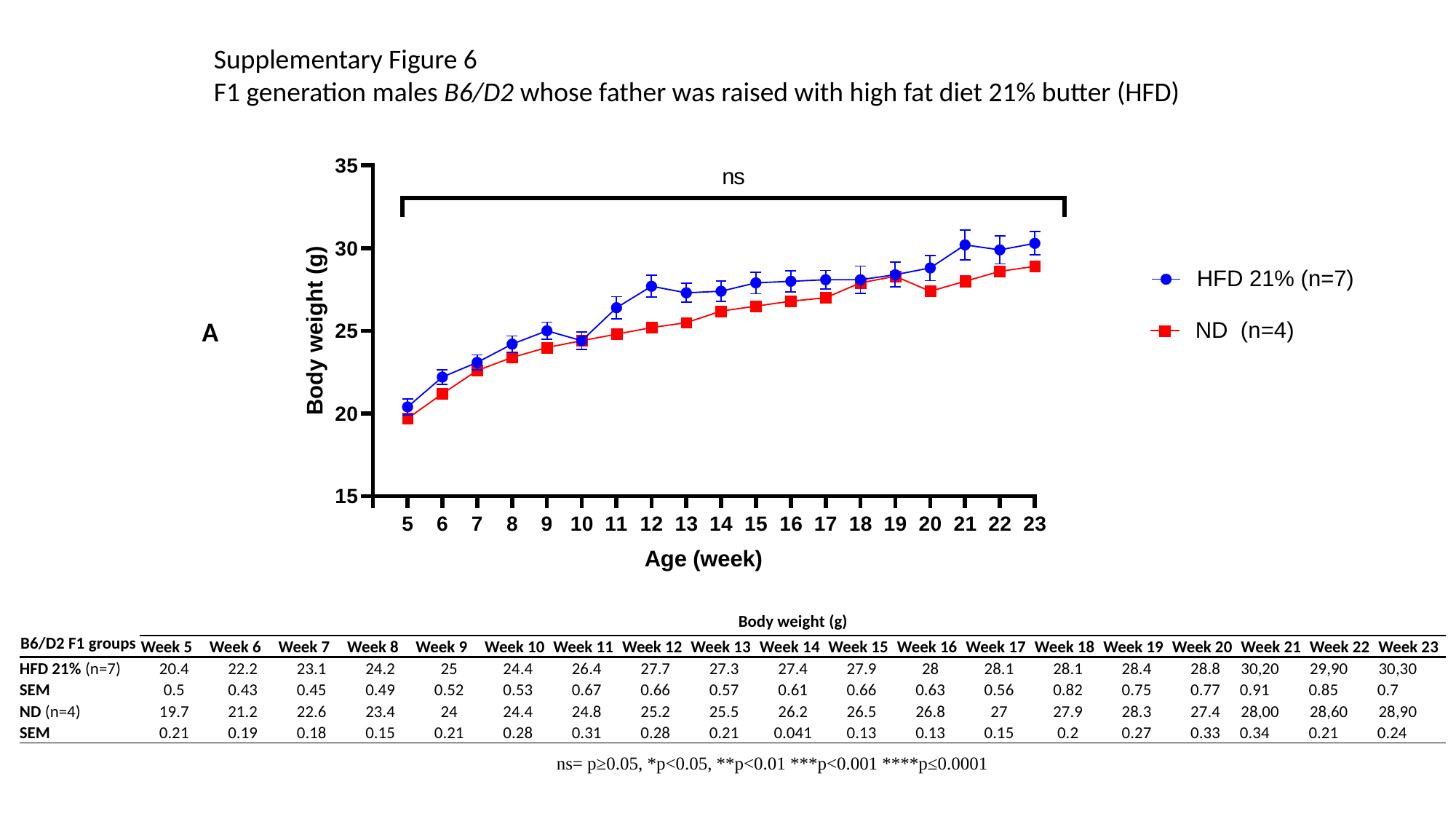

Supplementary Figure 6
F1 generation males B6/D2 whose father was raised with high fat diet 21% butter (HFD)
A
| | Body weight (g) | | | | | | | | | | | | | | | | | | |
| --- | --- | --- | --- | --- | --- | --- | --- | --- | --- | --- | --- | --- | --- | --- | --- | --- | --- | --- | --- |
| B6/D2 F1 groups | Week 5 | Week 6 | Week 7 | Week 8 | Week 9 | Week 10 | Week 11 | Week 12 | Week 13 | Week 14 | Week 15 | Week 16 | Week 17 | Week 18 | Week 19 | Week 20 | Week 21 | Week 22 | Week 23 |
| HFD 21% (n=7) | 20.4 | 22.2 | 23.1 | 24.2 | 25 | 24.4 | 26.4 | 27.7 | 27.3 | 27.4 | 27.9 | 28 | 28.1 | 28.1 | 28.4 | 28.8 | 30,20 | 29,90 | 30,30 |
| SEM | 0.5 | 0.43 | 0.45 | 0.49 | 0.52 | 0.53 | 0.67 | 0.66 | 0.57 | 0.61 | 0.66 | 0.63 | 0.56 | 0.82 | 0.75 | 0.77 | 0.91 | 0.85 | 0.7 |
| ND (n=4) | 19.7 | 21.2 | 22.6 | 23.4 | 24 | 24.4 | 24.8 | 25.2 | 25.5 | 26.2 | 26.5 | 26.8 | 27 | 27.9 | 28.3 | 27.4 | 28,00 | 28,60 | 28,90 |
| SEM | 0.21 | 0.19 | 0.18 | 0.15 | 0.21 | 0.28 | 0.31 | 0.28 | 0.21 | 0.041 | 0.13 | 0.13 | 0.15 | 0.2 | 0.27 | 0.33 | 0.34 | 0.21 | 0.24 |
ns= p≥0.05, *p<0.05, **p<0.01 ***p<0.001 ****p≤0.0001

## Slide 22
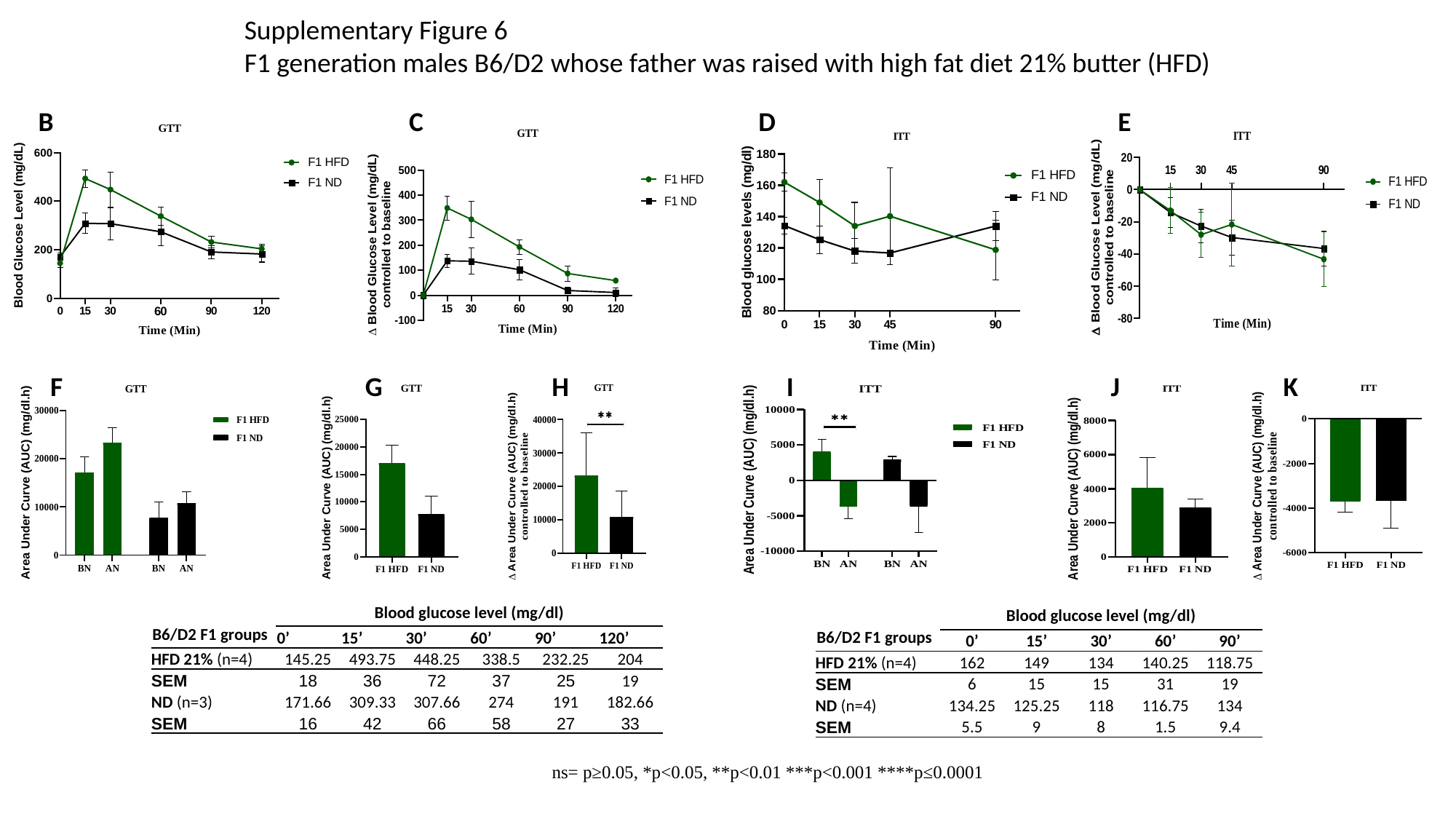

Supplementary Figure 6
F1 generation males B6/D2 whose father was raised with high fat diet 21% butter (HFD)
B
C
D
E
F
G
H
I
J
K
| | Blood glucose level (mg/dl) | | | | | |
| --- | --- | --- | --- | --- | --- | --- |
| B6/D2 F1 groups | 0’ | 15’ | 30’ | 60’ | 90’ | 120’ |
| HFD 21% (n=4) | 145.25 | 493.75 | 448.25 | 338.5 | 232.25 | 204 |
| SEM | 18 | 36 | 72 | 37 | 25 | 19 |
| ND (n=3) | 171.66 | 309.33 | 307.66 | 274 | 191 | 182.66 |
| SEM | 16 | 42 | 66 | 58 | 27 | 33 |
| | Blood glucose level (mg/dl) | | | | |
| --- | --- | --- | --- | --- | --- |
| B6/D2 F1 groups | 0’ | 15’ | 30’ | 60’ | 90’ |
| HFD 21% (n=4) | 162 | 149 | 134 | 140.25 | 118.75 |
| SEM | 6 | 15 | 15 | 31 | 19 |
| ND (n=4) | 134.25 | 125.25 | 118 | 116.75 | 134 |
| SEM | 5.5 | 9 | 8 | 1.5 | 9.4 |
ns= p≥0.05, *p<0.05, **p<0.01 ***p<0.001 ****p≤0.0001

## Slide 23
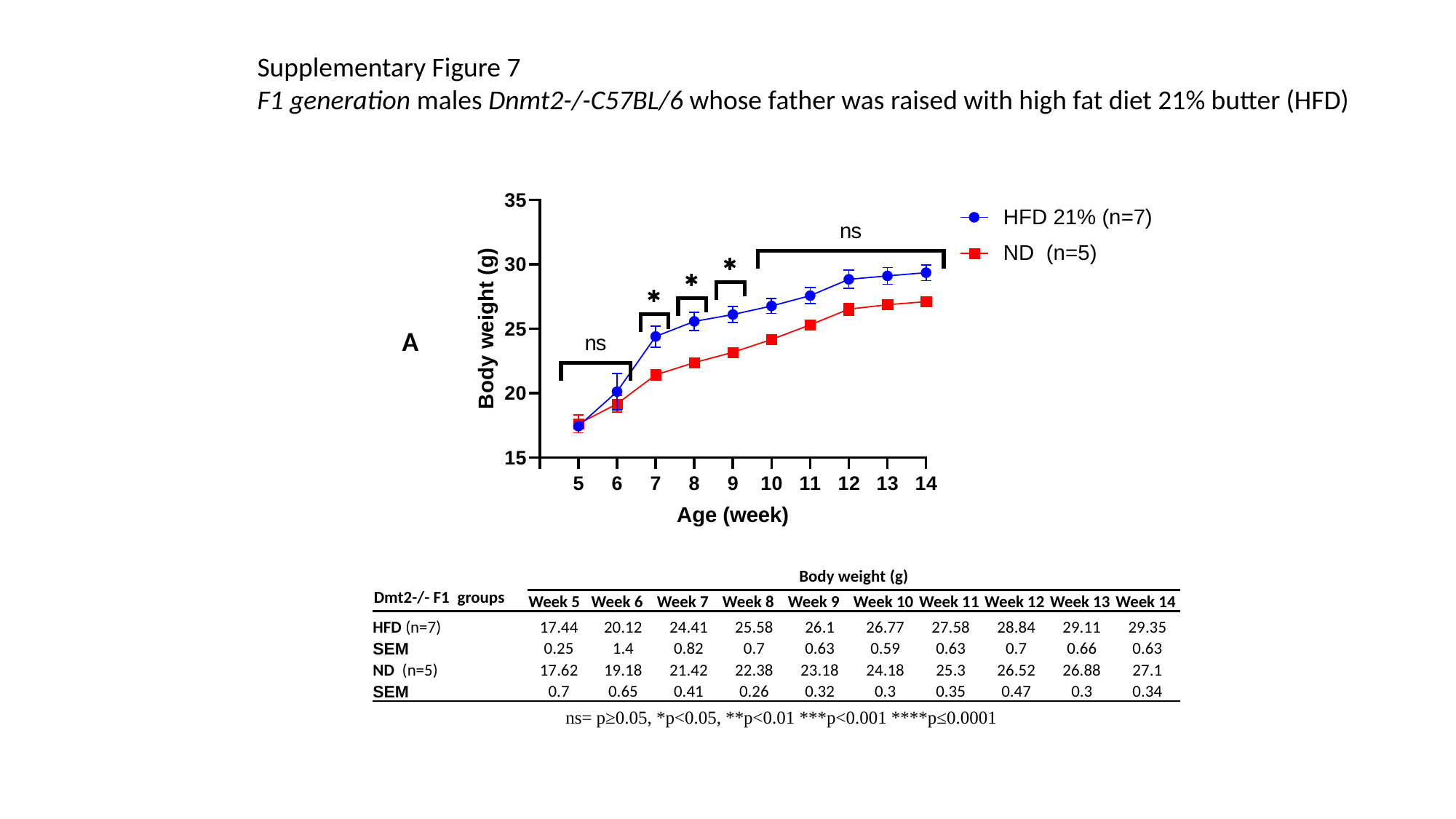

Supplementary Figure 7
F1 generation males Dnmt2-/-C57BL/6 whose father was raised with high fat diet 21% butter (HFD)
A
| | | Body weight (g) | | | | | | | | | |
| --- | --- | --- | --- | --- | --- | --- | --- | --- | --- | --- | --- |
| Dmt2-/- F1 groups | | Week 5 | Week 6 | Week 7 | Week 8 | Week 9 | Week 10 | Week 11 | Week 12 | Week 13 | Week 14 |
| HFD (n=7) | | 17.44 | 20.12 | 24.41 | 25.58 | 26.1 | 26.77 | 27.58 | 28.84 | 29.11 | 29.35 |
| SEM | | 0.25 | 1.4 | 0.82 | 0.7 | 0.63 | 0.59 | 0.63 | 0.7 | 0.66 | 0.63 |
| ND (n=5) | | 17.62 | 19.18 | 21.42 | 22.38 | 23.18 | 24.18 | 25.3 | 26.52 | 26.88 | 27.1 |
| SEM | | 0.7 | 0.65 | 0.41 | 0.26 | 0.32 | 0.3 | 0.35 | 0.47 | 0.3 | 0.34 |
ns= p≥0.05, *p<0.05, **p<0.01 ***p<0.001 ****p≤0.0001

## Slide 24
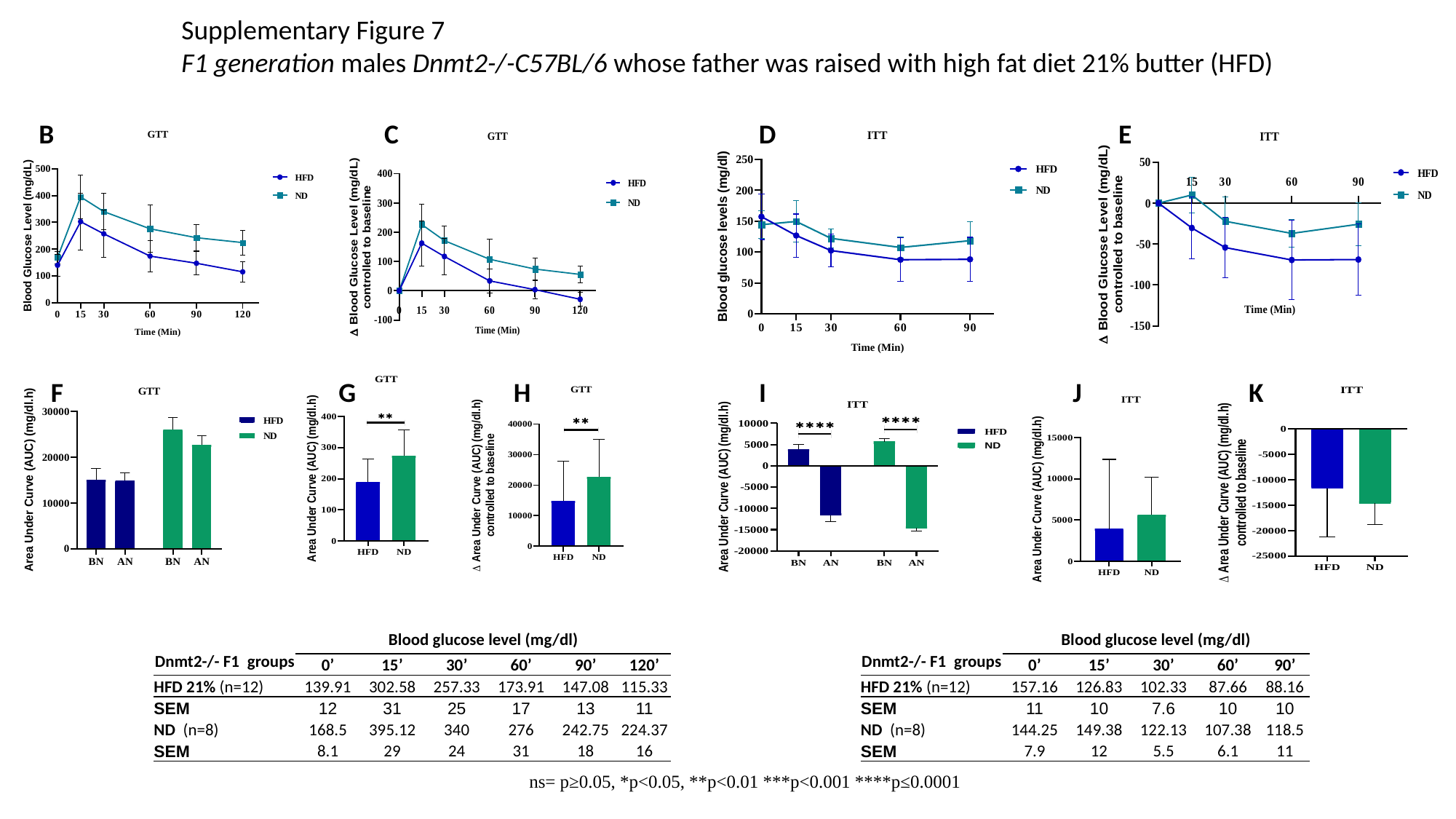

Supplementary Figure 7
F1 generation males Dnmt2-/-C57BL/6 whose father was raised with high fat diet 21% butter (HFD)
B
C
D
E
F
G
H
I
J
K
| | Blood glucose level (mg/dl) | | | | | |
| --- | --- | --- | --- | --- | --- | --- |
| Dnmt2-/- F1 groups | 0’ | 15’ | 30’ | 60’ | 90’ | 120’ |
| HFD 21% (n=12) | 139.91 | 302.58 | 257.33 | 173.91 | 147.08 | 115.33 |
| SEM | 12 | 31 | 25 | 17 | 13 | 11 |
| ND (n=8) | 168.5 | 395.12 | 340 | 276 | 242.75 | 224.37 |
| SEM | 8.1 | 29 | 24 | 31 | 18 | 16 |
| | Blood glucose level (mg/dl) | | | | |
| --- | --- | --- | --- | --- | --- |
| Dnmt2-/- F1 groups | 0’ | 15’ | 30’ | 60’ | 90’ |
| HFD 21% (n=12) | 157.16 | 126.83 | 102.33 | 87.66 | 88.16 |
| SEM | 11 | 10 | 7.6 | 10 | 10 |
| ND (n=8) | 144.25 | 149.38 | 122.13 | 107.38 | 118.5 |
| SEM | 7.9 | 12 | 5.5 | 6.1 | 11 |
ns= p≥0.05, *p<0.05, **p<0.01 ***p<0.001 ****p≤0.0001

## Slide 25
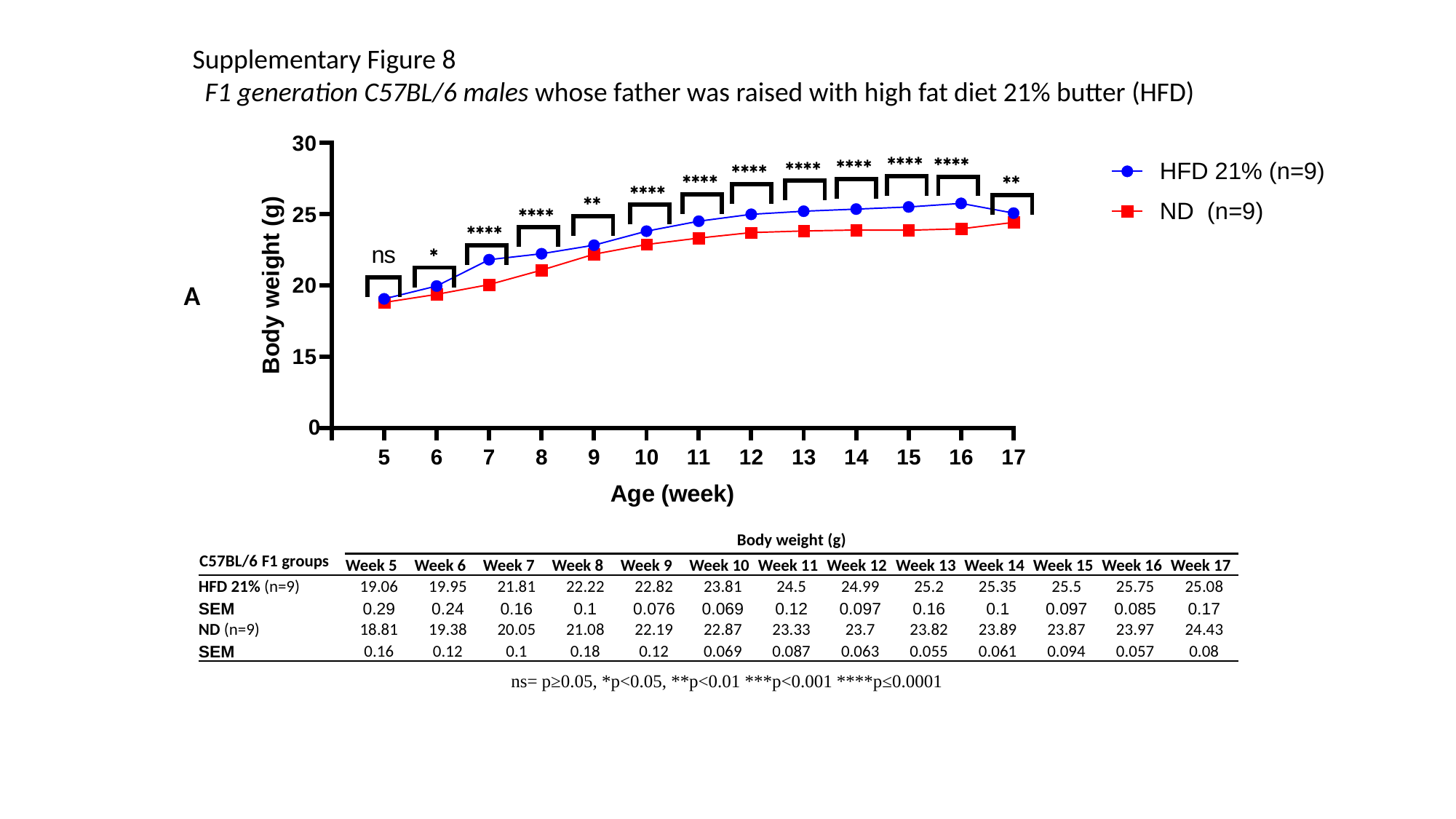

Supplementary Figure 8
 F1 generation C57BL/6 males whose father was raised with high fat diet 21% butter (HFD)
A
| | Body weight (g) | | | | | | | | | | | | |
| --- | --- | --- | --- | --- | --- | --- | --- | --- | --- | --- | --- | --- | --- |
| C57BL/6 F1 groups | Week 5 | Week 6 | Week 7 | Week 8 | Week 9 | Week 10 | Week 11 | Week 12 | Week 13 | Week 14 | Week 15 | Week 16 | Week 17 |
| HFD 21% (n=9) | 19.06 | 19.95 | 21.81 | 22.22 | 22.82 | 23.81 | 24.5 | 24.99 | 25.2 | 25.35 | 25.5 | 25.75 | 25.08 |
| SEM | 0.29 | 0.24 | 0.16 | 0.1 | 0.076 | 0.069 | 0.12 | 0.097 | 0.16 | 0.1 | 0.097 | 0.085 | 0.17 |
| ND (n=9) | 18.81 | 19.38 | 20.05 | 21.08 | 22.19 | 22.87 | 23.33 | 23.7 | 23.82 | 23.89 | 23.87 | 23.97 | 24.43 |
| SEM | 0.16 | 0.12 | 0.1 | 0.18 | 0.12 | 0.069 | 0.087 | 0.063 | 0.055 | 0.061 | 0.094 | 0.057 | 0.08 |
ns= p≥0.05, *p<0.05, **p<0.01 ***p<0.001 ****p≤0.0001

## Slide 26
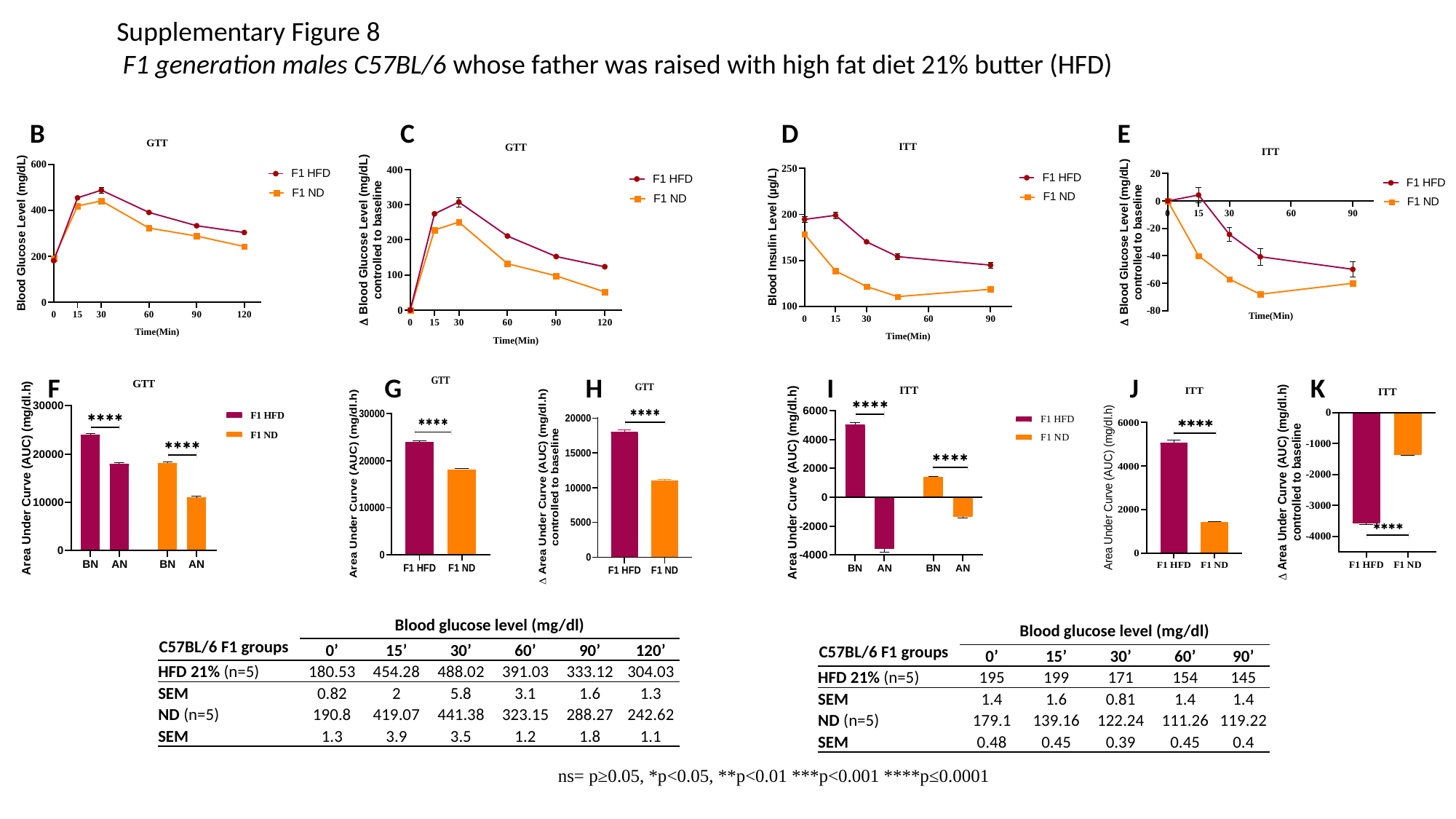

Supplementary Figure 8
 F1 generation males C57BL/6 whose father was raised with high fat diet 21% butter (HFD)
B
C
D
E
F
G
H
I
J
K
| | Blood glucose level (mg/dl) | | | | | |
| --- | --- | --- | --- | --- | --- | --- |
| C57BL/6 F1 groups | 0’ | 15’ | 30’ | 60’ | 90’ | 120’ |
| HFD 21% (n=5) | 180.53 | 454.28 | 488.02 | 391.03 | 333.12 | 304.03 |
| SEM | 0.82 | 2 | 5.8 | 3.1 | 1.6 | 1.3 |
| ND (n=5) | 190.8 | 419.07 | 441.38 | 323.15 | 288.27 | 242.62 |
| SEM | 1.3 | 3.9 | 3.5 | 1.2 | 1.8 | 1.1 |
| | Blood glucose level (mg/dl) | | | | |
| --- | --- | --- | --- | --- | --- |
| C57BL/6 F1 groups | 0’ | 15’ | 30’ | 60’ | 90’ |
| HFD 21% (n=5) | 195 | 199 | 171 | 154 | 145 |
| SEM | 1.4 | 1.6 | 0.81 | 1.4 | 1.4 |
| ND (n=5) | 179.1 | 139.16 | 122.24 | 111.26 | 119.22 |
| SEM | 0.48 | 0.45 | 0.39 | 0.45 | 0.4 |
ns= p≥0.05, *p<0.05, **p<0.01 ***p<0.001 ****p≤0.0001

## Slide 27
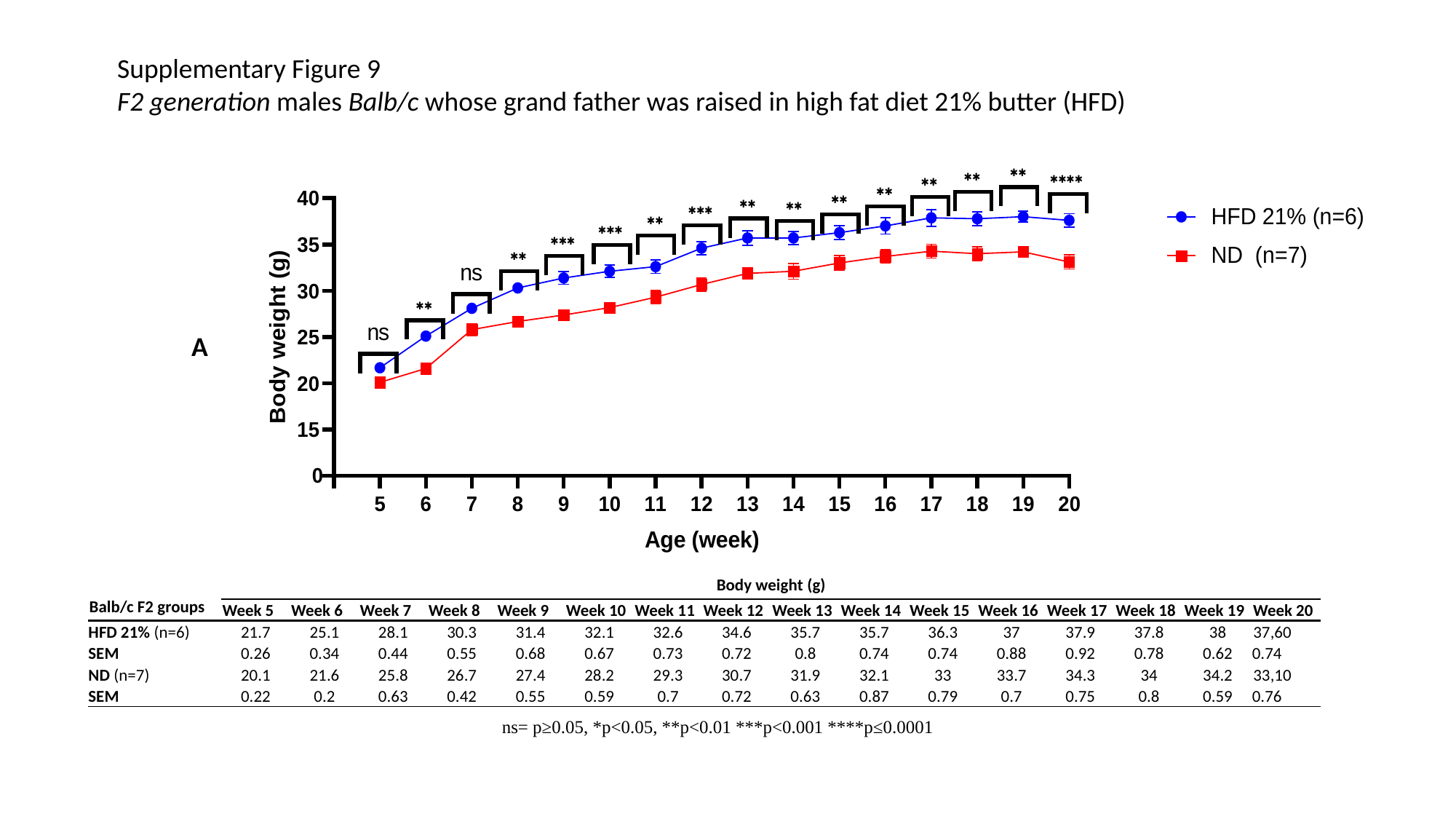

Supplementary Figure 9
F2 generation males Balb/c whose grand father was raised in high fat diet 21% butter (HFD)
A
| | Body weight (g) | | | | | | | | | | | | | | | |
| --- | --- | --- | --- | --- | --- | --- | --- | --- | --- | --- | --- | --- | --- | --- | --- | --- |
| Balb/c F2 groups | Week 5 | Week 6 | Week 7 | Week 8 | Week 9 | Week 10 | Week 11 | Week 12 | Week 13 | Week 14 | Week 15 | Week 16 | Week 17 | Week 18 | Week 19 | Week 20 |
| HFD 21% (n=6) | 21.7 | 25.1 | 28.1 | 30.3 | 31.4 | 32.1 | 32.6 | 34.6 | 35.7 | 35.7 | 36.3 | 37 | 37.9 | 37.8 | 38 | 37,60 |
| SEM | 0.26 | 0.34 | 0.44 | 0.55 | 0.68 | 0.67 | 0.73 | 0.72 | 0.8 | 0.74 | 0.74 | 0.88 | 0.92 | 0.78 | 0.62 | 0.74 |
| ND (n=7) | 20.1 | 21.6 | 25.8 | 26.7 | 27.4 | 28.2 | 29.3 | 30.7 | 31.9 | 32.1 | 33 | 33.7 | 34.3 | 34 | 34.2 | 33,10 |
| SEM | 0.22 | 0.2 | 0.63 | 0.42 | 0.55 | 0.59 | 0.7 | 0.72 | 0.63 | 0.87 | 0.79 | 0.7 | 0.75 | 0.8 | 0.59 | 0.76 |
ns= p≥0.05, *p<0.05, **p<0.01 ***p<0.001 ****p≤0.0001

## Slide 28
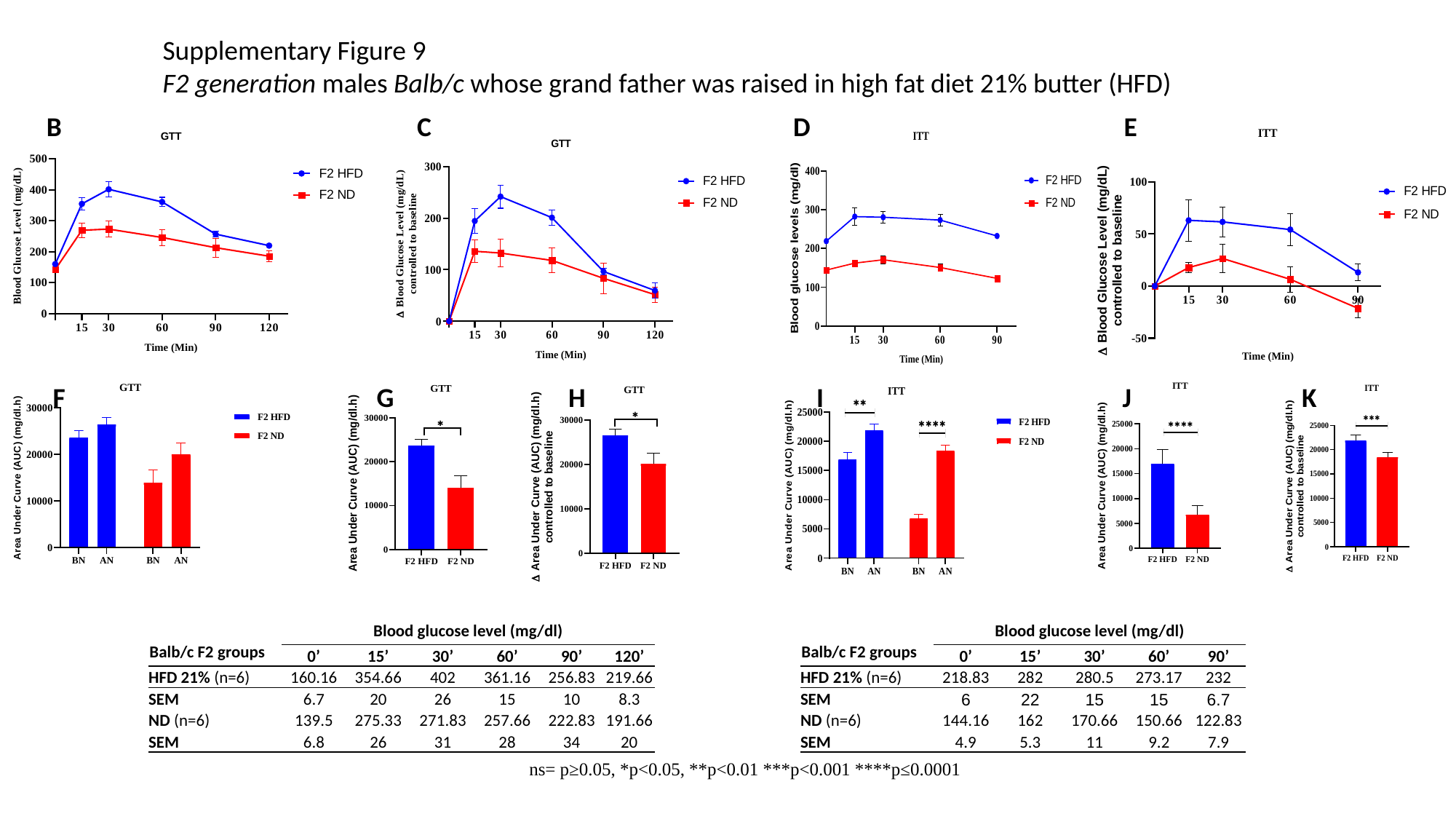

Supplementary Figure 9
F2 generation males Balb/c whose grand father was raised in high fat diet 21% butter (HFD)
B
C
D
E
F
G
H
I
J
K
| | Blood glucose level (mg/dl) | | | | | |
| --- | --- | --- | --- | --- | --- | --- |
| Balb/c F2 groups | 0’ | 15’ | 30’ | 60’ | 90’ | 120’ |
| HFD 21% (n=6) | 160.16 | 354.66 | 402 | 361.16 | 256.83 | 219.66 |
| SEM | 6.7 | 20 | 26 | 15 | 10 | 8.3 |
| ND (n=6) | 139.5 | 275.33 | 271.83 | 257.66 | 222.83 | 191.66 |
| SEM | 6.8 | 26 | 31 | 28 | 34 | 20 |
| | Blood glucose level (mg/dl) | | | | |
| --- | --- | --- | --- | --- | --- |
| Balb/c F2 groups | 0’ | 15’ | 30’ | 60’ | 90’ |
| HFD 21% (n=6) | 218.83 | 282 | 280.5 | 273.17 | 232 |
| SEM | 6 | 22 | 15 | 15 | 6.7 |
| ND (n=6) | 144.16 | 162 | 170.66 | 150.66 | 122.83 |
| SEM | 4.9 | 5.3 | 11 | 9.2 | 7.9 |
ns= p≥0.05, *p<0.05, **p<0.01 ***p<0.001 ****p≤0.0001

## Slide 29
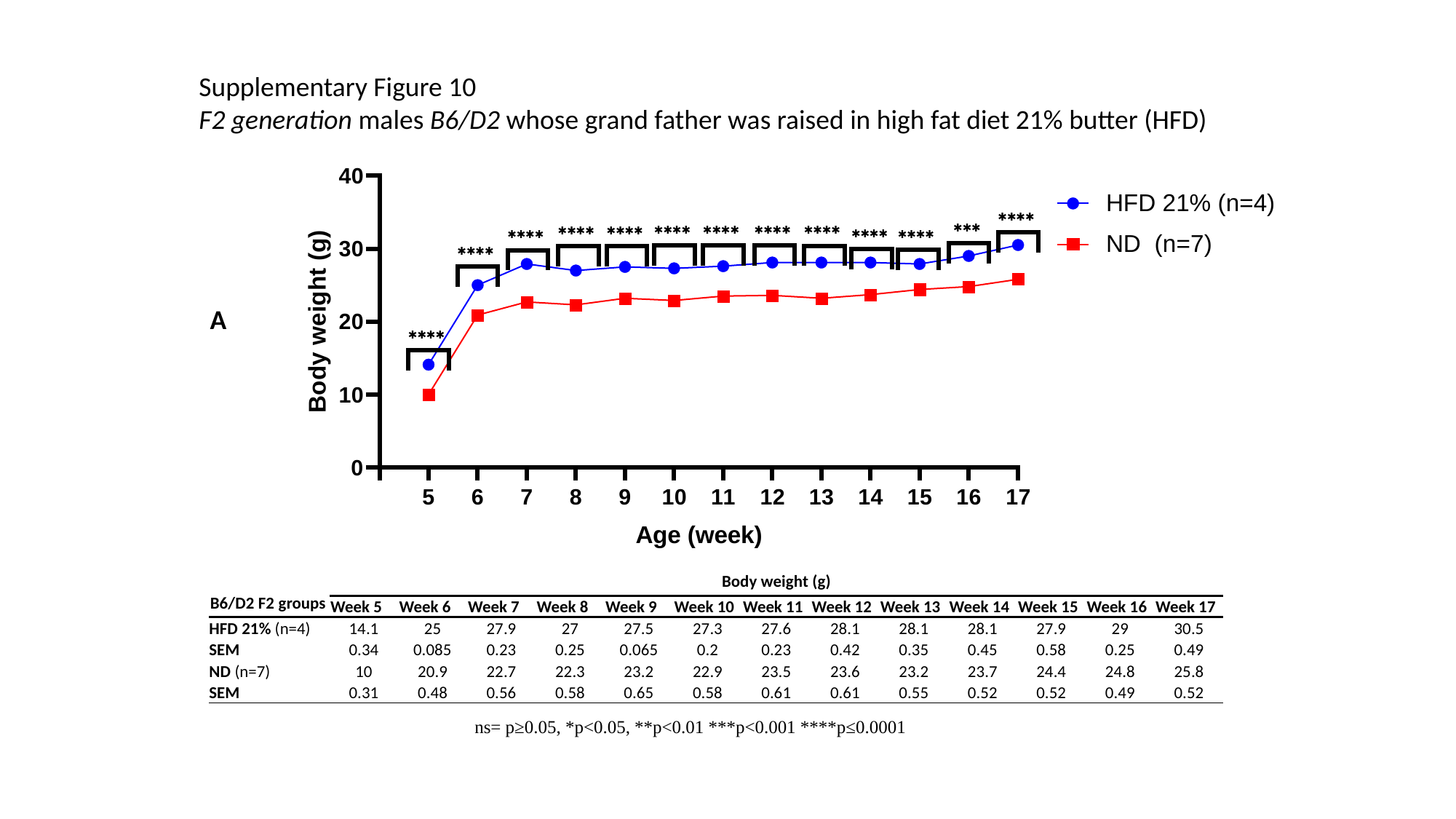

Supplementary Figure 10
F2 generation males B6/D2 whose grand father was raised in high fat diet 21% butter (HFD)
A
| | Body weight (g) | | | | | | | | | | | | |
| --- | --- | --- | --- | --- | --- | --- | --- | --- | --- | --- | --- | --- | --- |
| B6/D2 F2 groups | Week 5 | Week 6 | Week 7 | Week 8 | Week 9 | Week 10 | Week 11 | Week 12 | Week 13 | Week 14 | Week 15 | Week 16 | Week 17 |
| HFD 21% (n=4) | 14.1 | 25 | 27.9 | 27 | 27.5 | 27.3 | 27.6 | 28.1 | 28.1 | 28.1 | 27.9 | 29 | 30.5 |
| SEM | 0.34 | 0.085 | 0.23 | 0.25 | 0.065 | 0.2 | 0.23 | 0.42 | 0.35 | 0.45 | 0.58 | 0.25 | 0.49 |
| ND (n=7) | 10 | 20.9 | 22.7 | 22.3 | 23.2 | 22.9 | 23.5 | 23.6 | 23.2 | 23.7 | 24.4 | 24.8 | 25.8 |
| SEM | 0.31 | 0.48 | 0.56 | 0.58 | 0.65 | 0.58 | 0.61 | 0.61 | 0.55 | 0.52 | 0.52 | 0.49 | 0.52 |
ns= p≥0.05, *p<0.05, **p<0.01 ***p<0.001 ****p≤0.0001

## Slide 30
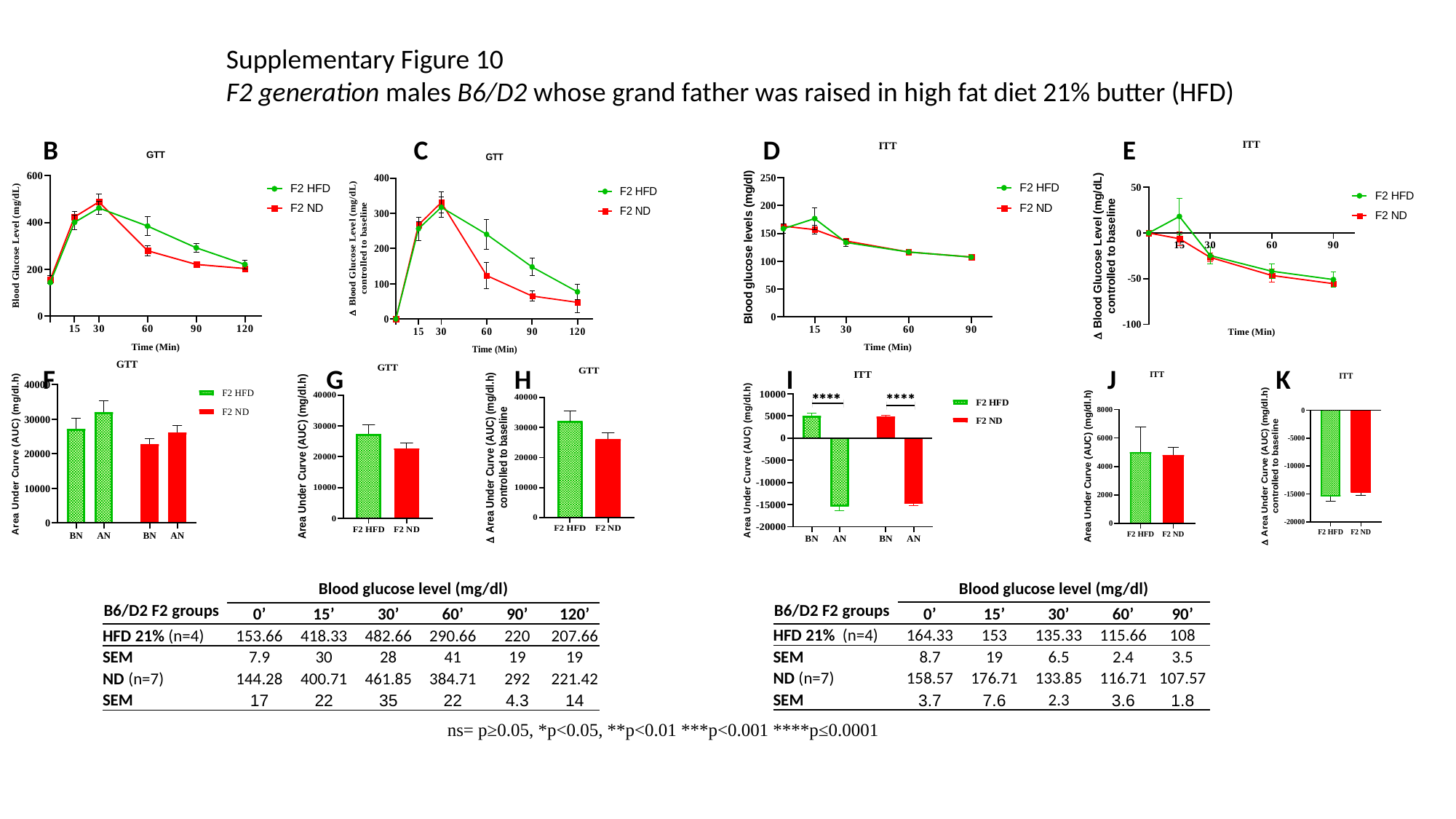

Supplementary Figure 10
F2 generation males B6/D2 whose grand father was raised in high fat diet 21% butter (HFD)
B
C
D
E
F
G
H
I
J
K
| | Blood glucose level (mg/dl) | | | | |
| --- | --- | --- | --- | --- | --- |
| B6/D2 F2 groups | 0’ | 15’ | 30’ | 60’ | 90’ |
| HFD 21% (n=4) | 164.33 | 153 | 135.33 | 115.66 | 108 |
| SEM | 8.7 | 19 | 6.5 | 2.4 | 3.5 |
| ND (n=7) | 158.57 | 176.71 | 133.85 | 116.71 | 107.57 |
| SEM | 3.7 | 7.6 | 2.3 | 3.6 | 1.8 |
| | Blood glucose level (mg/dl) | | | | | |
| --- | --- | --- | --- | --- | --- | --- |
| B6/D2 F2 groups | 0’ | 15’ | 30’ | 60’ | 90’ | 120’ |
| HFD 21% (n=4) | 153.66 | 418.33 | 482.66 | 290.66 | 220 | 207.66 |
| SEM | 7.9 | 30 | 28 | 41 | 19 | 19 |
| ND (n=7) | 144.28 | 400.71 | 461.85 | 384.71 | 292 | 221.42 |
| SEM | 17 | 22 | 35 | 22 | 4.3 | 14 |
ns= p≥0.05, *p<0.05, **p<0.01 ***p<0.001 ****p≤0.0001

## Slide 31
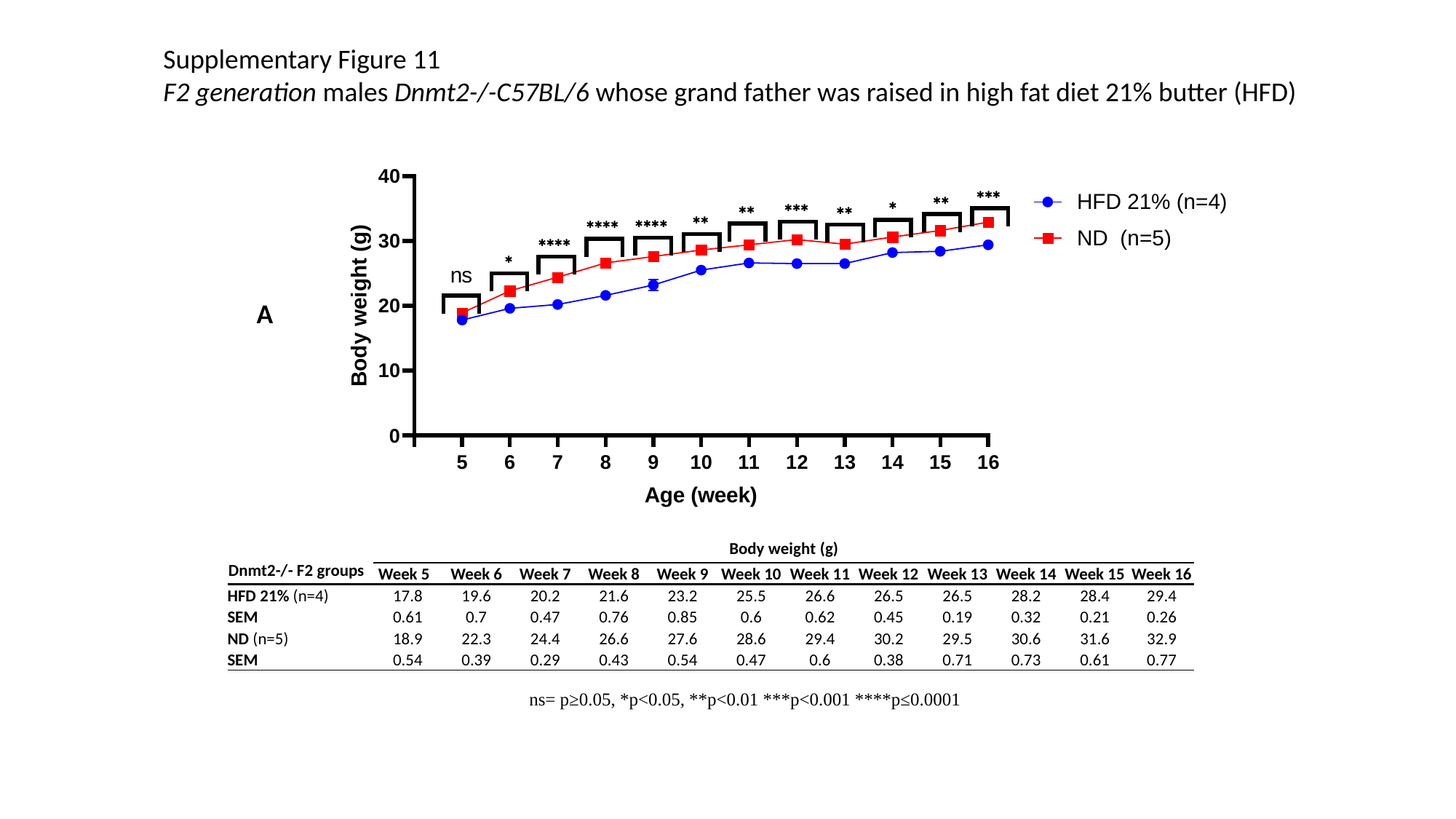

Supplementary Figure 11
F2 generation males Dnmt2-/-C57BL/6 whose grand father was raised in high fat diet 21% butter (HFD)
A
| | Body weight (g) | | | | | | | | | | | |
| --- | --- | --- | --- | --- | --- | --- | --- | --- | --- | --- | --- | --- |
| Dnmt2-/- F2 groups | Week 5 | Week 6 | Week 7 | Week 8 | Week 9 | Week 10 | Week 11 | Week 12 | Week 13 | Week 14 | Week 15 | Week 16 |
| HFD 21% (n=4) | 17.8 | 19.6 | 20.2 | 21.6 | 23.2 | 25.5 | 26.6 | 26.5 | 26.5 | 28.2 | 28.4 | 29.4 |
| SEM | 0.61 | 0.7 | 0.47 | 0.76 | 0.85 | 0.6 | 0.62 | 0.45 | 0.19 | 0.32 | 0.21 | 0.26 |
| ND (n=5) | 18.9 | 22.3 | 24.4 | 26.6 | 27.6 | 28.6 | 29.4 | 30.2 | 29.5 | 30.6 | 31.6 | 32.9 |
| SEM | 0.54 | 0.39 | 0.29 | 0.43 | 0.54 | 0.47 | 0.6 | 0.38 | 0.71 | 0.73 | 0.61 | 0.77 |
ns= p≥0.05, *p<0.05, **p<0.01 ***p<0.001 ****p≤0.0001

## Slide 32
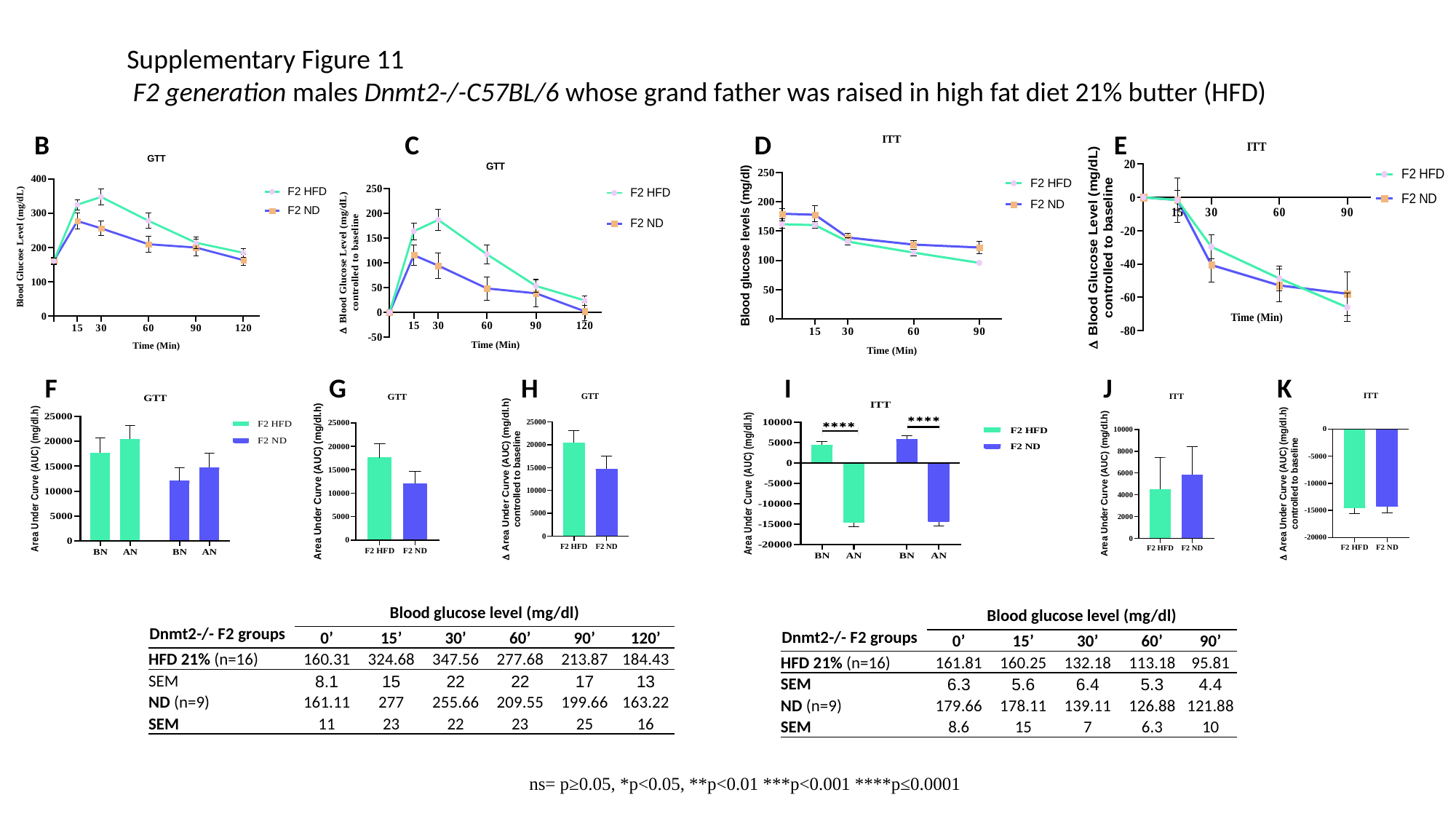

Supplementary Figure 11
 F2 generation males Dnmt2-/-C57BL/6 whose grand father was raised in high fat diet 21% butter (HFD)
B
C
D
E
F
G
H
I
J
K
| | Blood glucose level (mg/dl) | | | | | |
| --- | --- | --- | --- | --- | --- | --- |
| Dnmt2-/- F2 groups | 0’ | 15’ | 30’ | 60’ | 90’ | 120’ |
| HFD 21% (n=16) | 160.31 | 324.68 | 347.56 | 277.68 | 213.87 | 184.43 |
| SEM | 8.1 | 15 | 22 | 22 | 17 | 13 |
| ND (n=9) | 161.11 | 277 | 255.66 | 209.55 | 199.66 | 163.22 |
| SEM | 11 | 23 | 22 | 23 | 25 | 16 |
| | Blood glucose level (mg/dl) | | | | |
| --- | --- | --- | --- | --- | --- |
| Dnmt2-/- F2 groups | 0’ | 15’ | 30’ | 60’ | 90’ |
| HFD 21% (n=16) | 161.81 | 160.25 | 132.18 | 113.18 | 95.81 |
| SEM | 6.3 | 5.6 | 6.4 | 5.3 | 4.4 |
| ND (n=9) | 179.66 | 178.11 | 139.11 | 126.88 | 121.88 |
| SEM | 8.6 | 15 | 7 | 6.3 | 10 |
ns= p≥0.05, *p<0.05, **p<0.01 ***p<0.001 ****p≤0.0001

## Slide 33
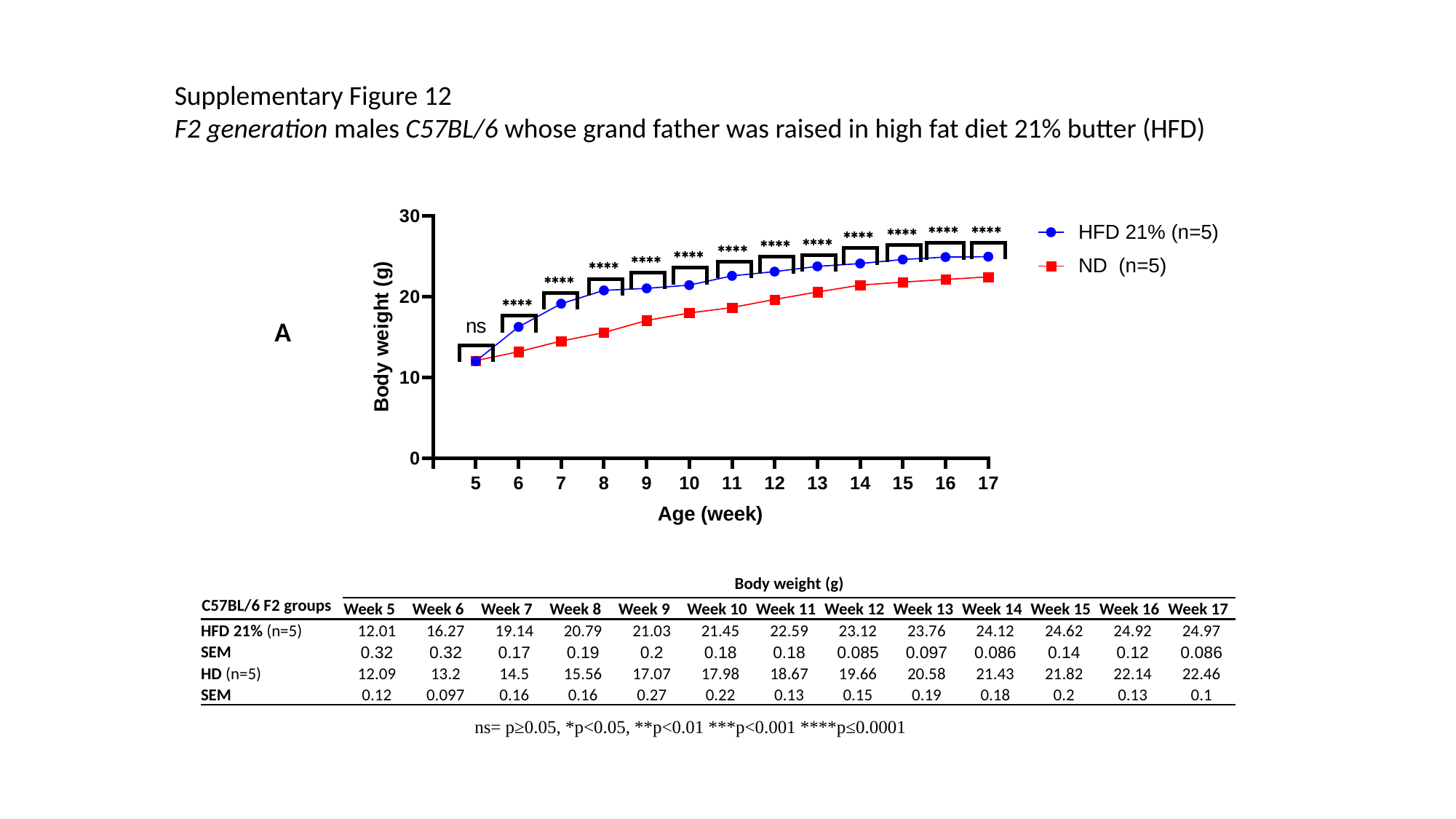

Supplementary Figure 12
F2 generation males C57BL/6 whose grand father was raised in high fat diet 21% butter (HFD)
A
| | Body weight (g) | | | | | | | | | | | | |
| --- | --- | --- | --- | --- | --- | --- | --- | --- | --- | --- | --- | --- | --- |
| C57BL/6 F2 groups | Week 5 | Week 6 | Week 7 | Week 8 | Week 9 | Week 10 | Week 11 | Week 12 | Week 13 | Week 14 | Week 15 | Week 16 | Week 17 |
| HFD 21% (n=5) | 12.01 | 16.27 | 19.14 | 20.79 | 21.03 | 21.45 | 22.59 | 23.12 | 23.76 | 24.12 | 24.62 | 24.92 | 24.97 |
| SEM | 0.32 | 0.32 | 0.17 | 0.19 | 0.2 | 0.18 | 0.18 | 0.085 | 0.097 | 0.086 | 0.14 | 0.12 | 0.086 |
| HD (n=5) | 12.09 | 13.2 | 14.5 | 15.56 | 17.07 | 17.98 | 18.67 | 19.66 | 20.58 | 21.43 | 21.82 | 22.14 | 22.46 |
| SEM | 0.12 | 0.097 | 0.16 | 0.16 | 0.27 | 0.22 | 0.13 | 0.15 | 0.19 | 0.18 | 0.2 | 0.13 | 0.1 |
ns= p≥0.05, *p<0.05, **p<0.01 ***p<0.001 ****p≤0.0001

## Slide 34
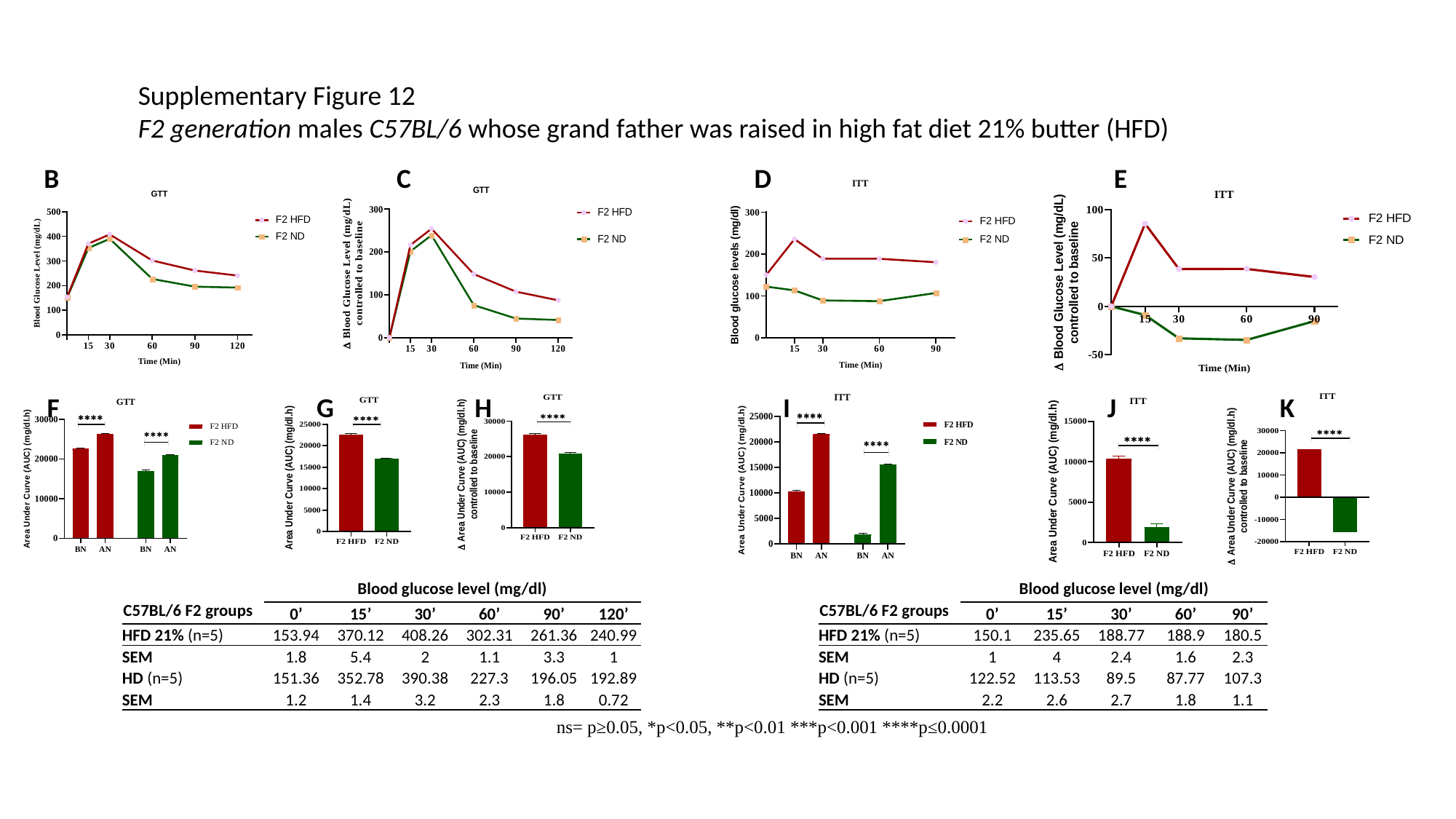

Supplementary Figure 12
F2 generation males C57BL/6 whose grand father was raised in high fat diet 21% butter (HFD)
B
C
D
E
F
G
H
I
J
K
| | Blood glucose level (mg/dl) | | | | | |
| --- | --- | --- | --- | --- | --- | --- |
| C57BL/6 F2 groups | 0’ | 15’ | 30’ | 60’ | 90’ | 120’ |
| HFD 21% (n=5) | 153.94 | 370.12 | 408.26 | 302.31 | 261.36 | 240.99 |
| SEM | 1.8 | 5.4 | 2 | 1.1 | 3.3 | 1 |
| HD (n=5) | 151.36 | 352.78 | 390.38 | 227.3 | 196.05 | 192.89 |
| SEM | 1.2 | 1.4 | 3.2 | 2.3 | 1.8 | 0.72 |
| | Blood glucose level (mg/dl) | | | | |
| --- | --- | --- | --- | --- | --- |
| C57BL/6 F2 groups | 0’ | 15’ | 30’ | 60’ | 90’ |
| HFD 21% (n=5) | 150.1 | 235.65 | 188.77 | 188.9 | 180.5 |
| SEM | 1 | 4 | 2.4 | 1.6 | 2.3 |
| HD (n=5) | 122.52 | 113.53 | 89.5 | 87.77 | 107.3 |
| SEM | 2.2 | 2.6 | 2.7 | 1.8 | 1.1 |
ns= p≥0.05, *p<0.05, **p<0.01 ***p<0.001 ****p≤0.0001

## Slide 35
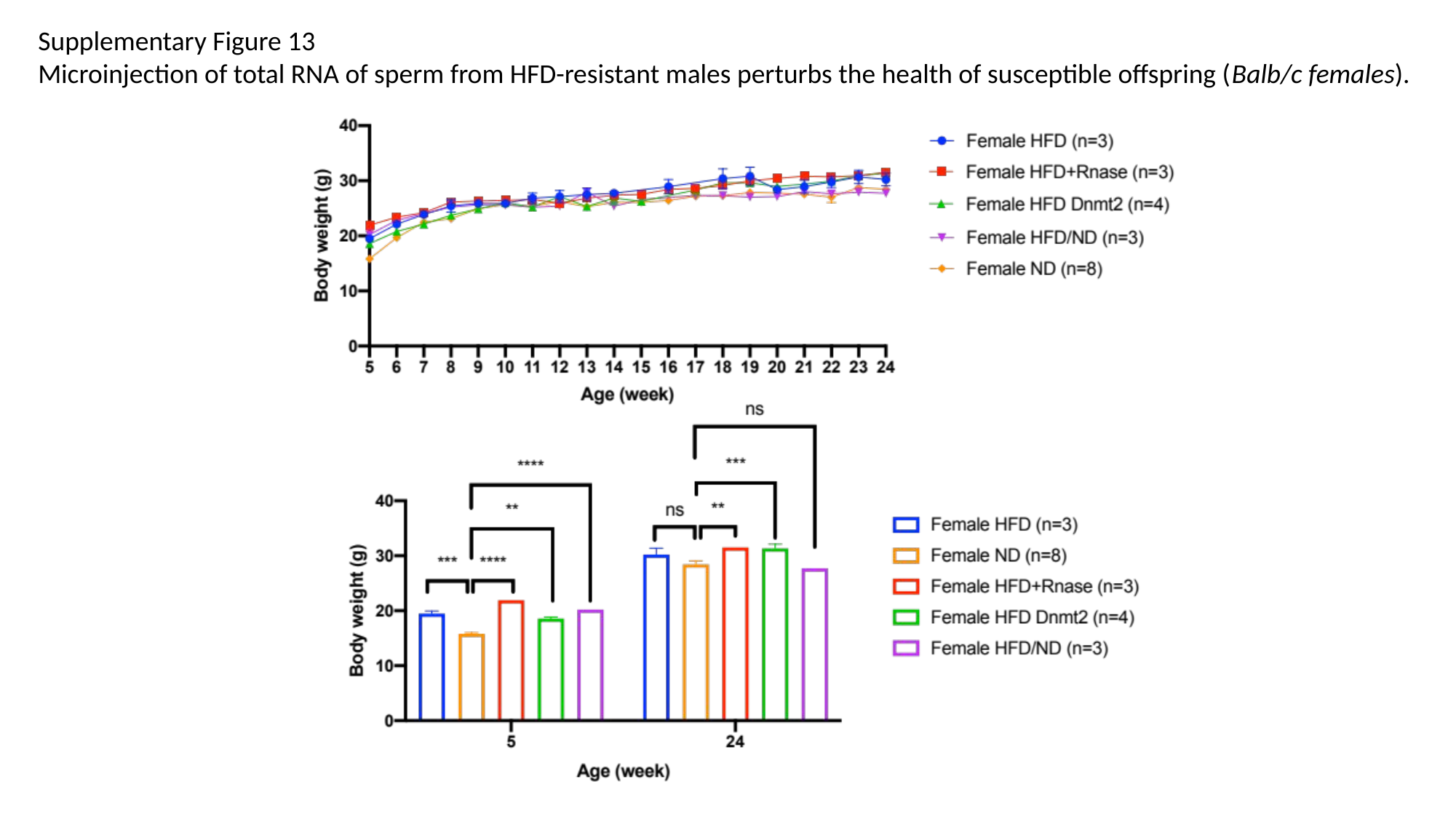

Supplementary Figure 13
Microinjection of total RNA of sperm from HFD-resistant males perturbs the health of susceptible offspring (Balb/c females).

## Slide 36
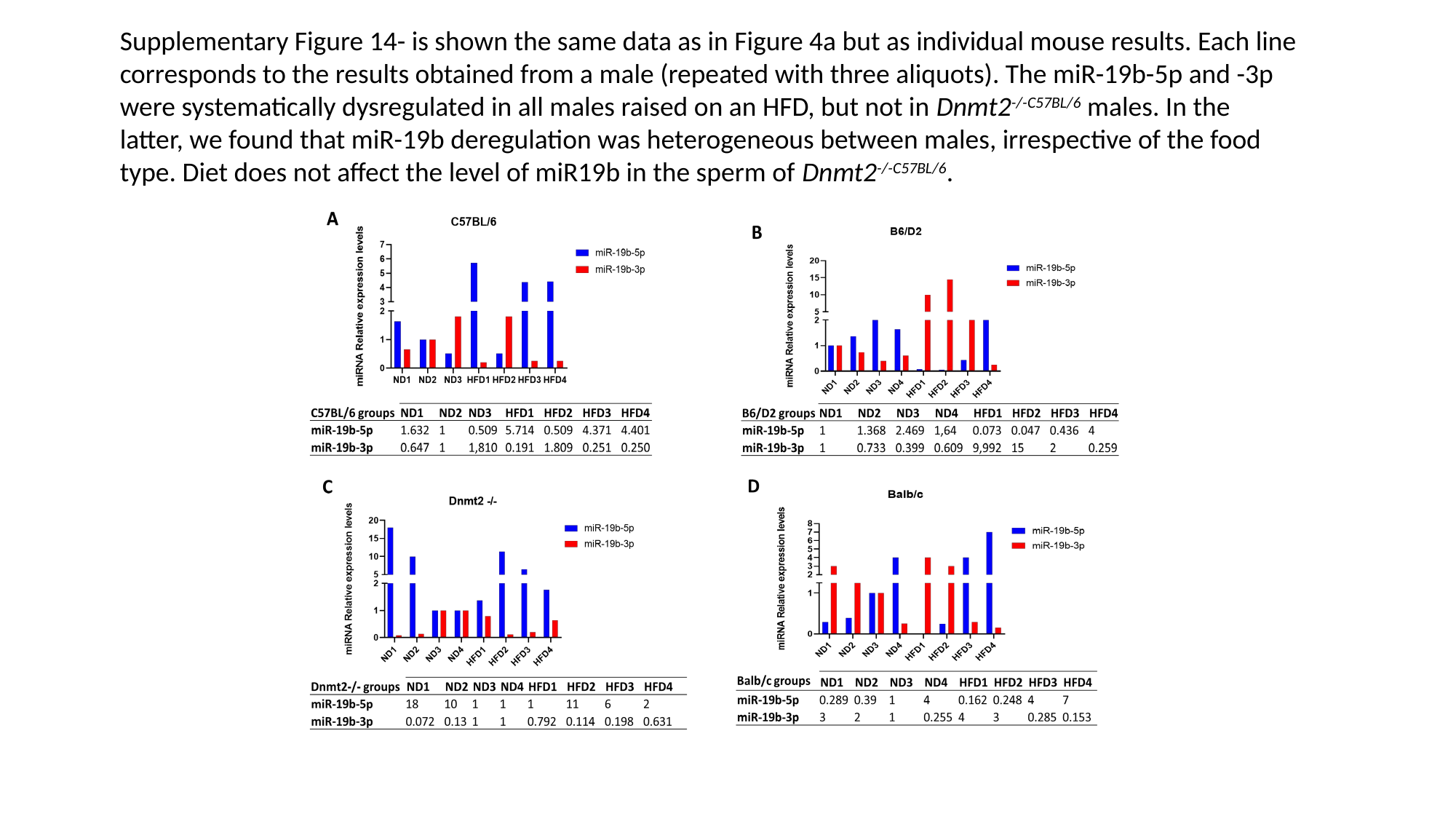

Supplementary Figure 14- is shown the same data as in Figure 4a but as individual mouse results. Each line corresponds to the results obtained from a male (repeated with three aliquots). The miR-19b-5p and -3p were systematically dysregulated in all males raised on an HFD, but not in Dnmt2-/-C57BL/6 males. In the latter, we found that miR-19b deregulation was heterogeneous between males, irrespective of the food type. Diet does not affect the level of miR19b in the sperm of Dnmt2-/-C57BL/6.

## Slide 37
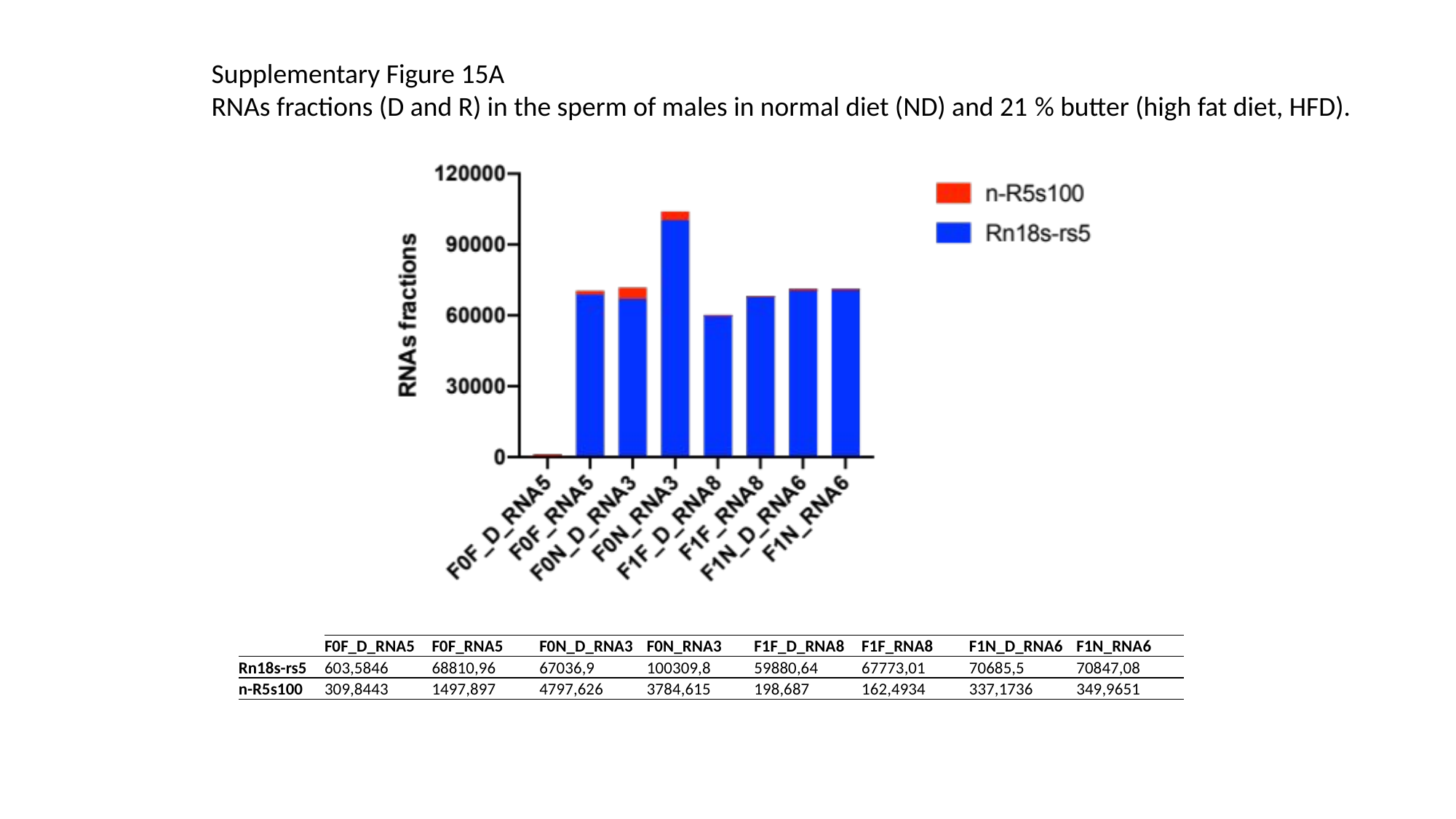

Supplementary Figure 15A
RNAs fractions (D and R) in the sperm of males in normal diet (ND) and 21 % butter (high fat diet, HFD).
| | F0F\_D\_RNA5 | F0F\_RNA5 | F0N\_D\_RNA3 | F0N\_RNA3 | F1F\_D\_RNA8 | F1F\_RNA8 | F1N\_D\_RNA6 | F1N\_RNA6 |
| --- | --- | --- | --- | --- | --- | --- | --- | --- |
| Rn18s-rs5 | 603,5846 | 68810,96 | 67036,9 | 100309,8 | 59880,64 | 67773,01 | 70685,5 | 70847,08 |
| n-R5s100 | 309,8443 | 1497,897 | 4797,626 | 3784,615 | 198,687 | 162,4934 | 337,1736 | 349,9651 |

## Slide 38
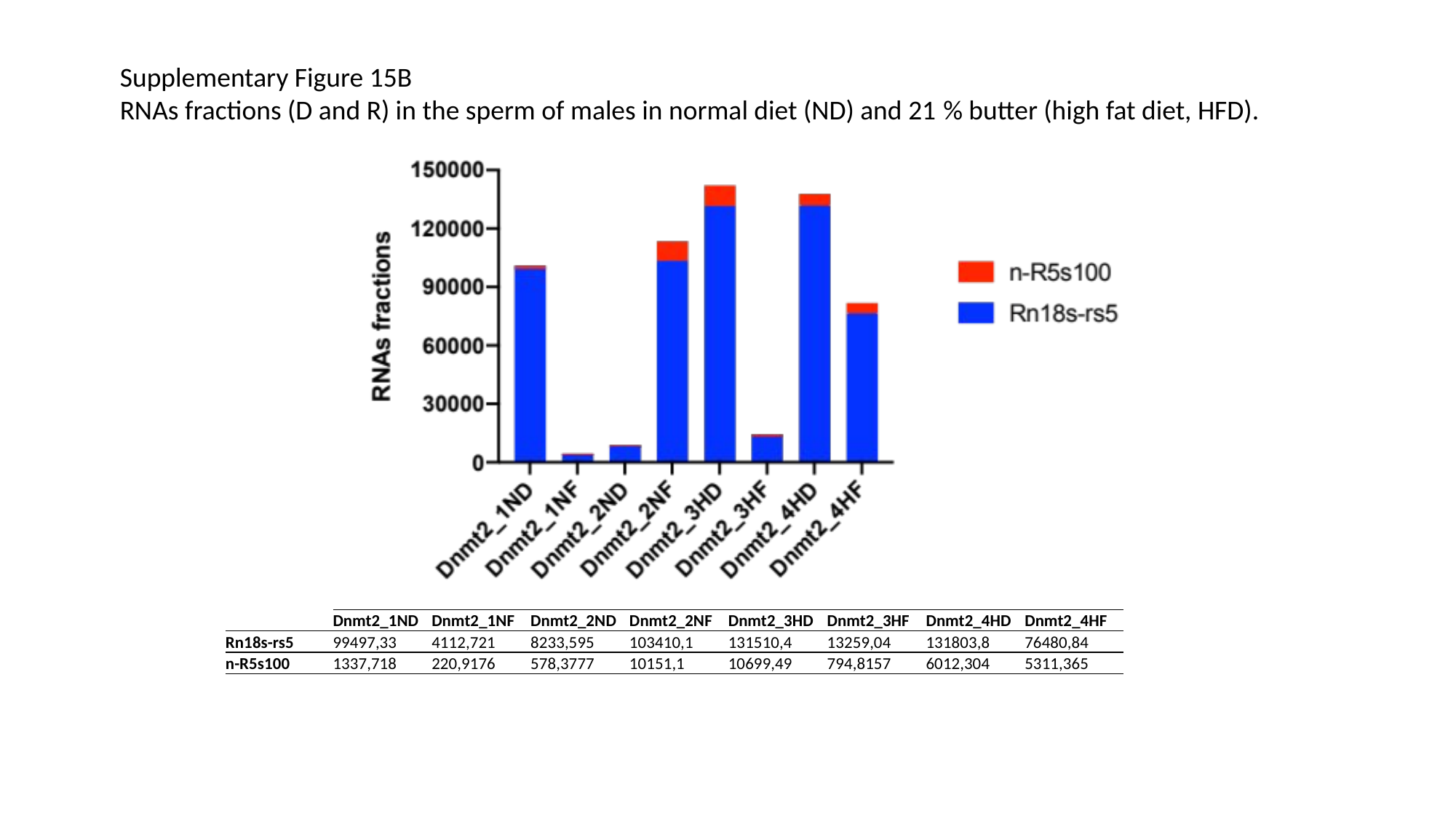

Supplementary Figure 15B
RNAs fractions (D and R) in the sperm of males in normal diet (ND) and 21 % butter (high fat diet, HFD).
| | Dnmt2\_1ND | Dnmt2\_1NF | Dnmt2\_2ND | Dnmt2\_2NF | Dnmt2\_3HD | Dnmt2\_3HF | Dnmt2\_4HD | Dnmt2\_4HF |
| --- | --- | --- | --- | --- | --- | --- | --- | --- |
| Rn18s-rs5 | 99497,33 | 4112,721 | 8233,595 | 103410,1 | 131510,4 | 13259,04 | 131803,8 | 76480,84 |
| n-R5s100 | 1337,718 | 220,9176 | 578,3777 | 10151,1 | 10699,49 | 794,8157 | 6012,304 | 5311,365 |

## Slide 39
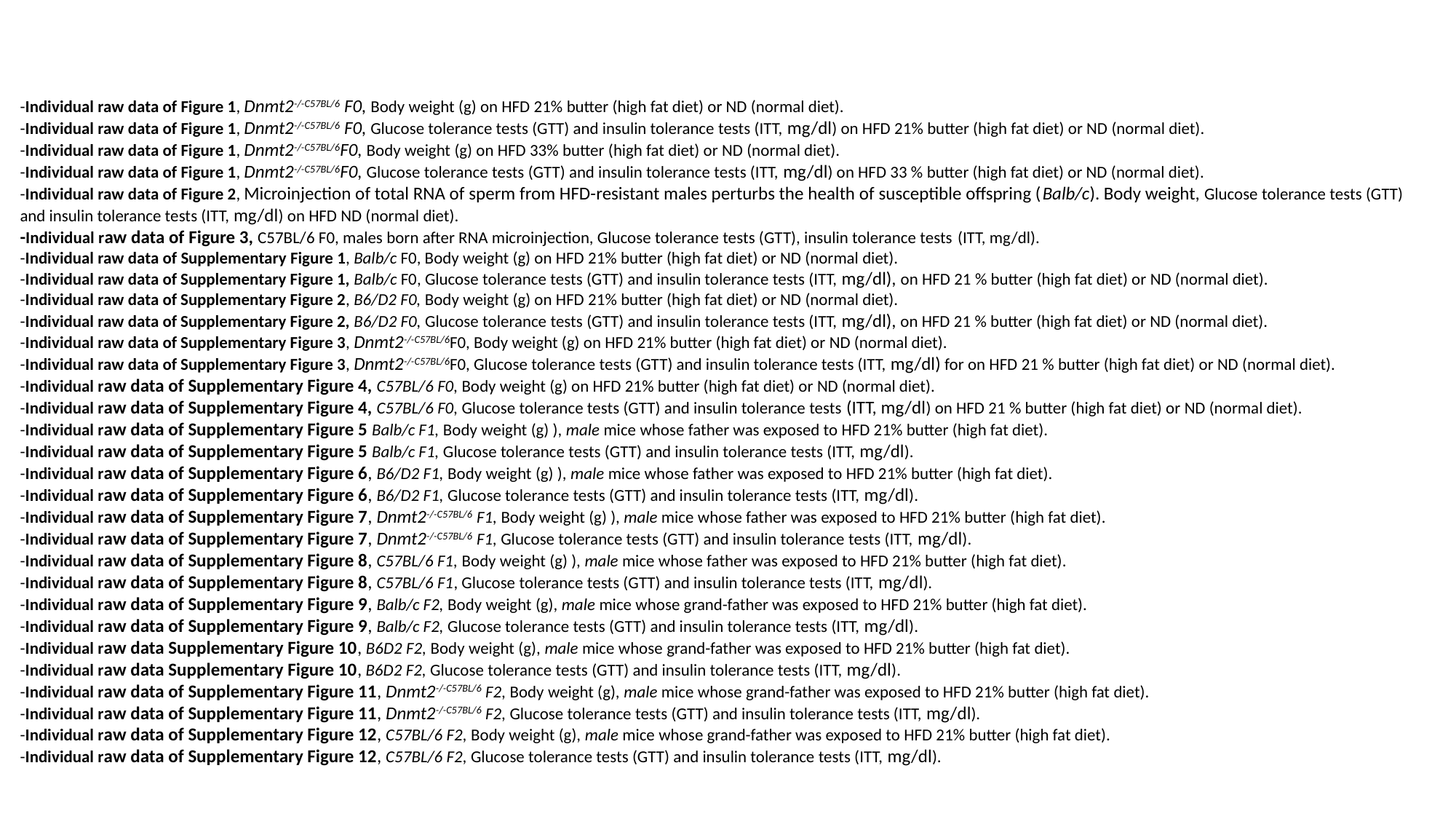

-Individual raw data of Figure 1, Dnmt2-/-C57BL/6 F0, Body weight (g) on HFD 21% butter (high fat diet) or ND (normal diet).
-Individual raw data of Figure 1, Dnmt2-/-C57BL/6 F0, Glucose tolerance tests (GTT) and insulin tolerance tests (ITT, mg/dl) on HFD 21% butter (high fat diet) or ND (normal diet).
-Individual raw data of Figure 1, Dnmt2-/-C57BL/6F0, Body weight (g) on HFD 33% butter (high fat diet) or ND (normal diet).
-Individual raw data of Figure 1, Dnmt2-/-C57BL/6F0, Glucose tolerance tests (GTT) and insulin tolerance tests (ITT, mg/dl) on HFD 33 % butter (high fat diet) or ND (normal diet).
-Individual raw data of Figure 2, Microinjection of total RNA of sperm from HFD-resistant males perturbs the health of susceptible offspring (Balb/c). Body weight, Glucose tolerance tests (GTT) and insulin tolerance tests (ITT, mg/dl) on HFD ND (normal diet).
-Individual raw data of Figure 3, C57BL/6 F0, males born after RNA microinjection, Glucose tolerance tests (GTT), insulin tolerance tests (ITT, mg/dl).
-Individual raw data of Supplementary Figure 1, Balb/c F0, Body weight (g) on HFD 21% butter (high fat diet) or ND (normal diet).
-Individual raw data of Supplementary Figure 1, Balb/c F0, Glucose tolerance tests (GTT) and insulin tolerance tests (ITT, mg/dl), on HFD 21 % butter (high fat diet) or ND (normal diet).
-Individual raw data of Supplementary Figure 2, B6/D2 F0, Body weight (g) on HFD 21% butter (high fat diet) or ND (normal diet).
-Individual raw data of Supplementary Figure 2, B6/D2 F0, Glucose tolerance tests (GTT) and insulin tolerance tests (ITT, mg/dl), on HFD 21 % butter (high fat diet) or ND (normal diet).
-Individual raw data of Supplementary Figure 3, Dnmt2-/-C57BL/6F0, Body weight (g) on HFD 21% butter (high fat diet) or ND (normal diet).
-Individual raw data of Supplementary Figure 3, Dnmt2-/-C57BL/6F0, Glucose tolerance tests (GTT) and insulin tolerance tests (ITT, mg/dl) for on HFD 21 % butter (high fat diet) or ND (normal diet).
-Individual raw data of Supplementary Figure 4, C57BL/6 F0, Body weight (g) on HFD 21% butter (high fat diet) or ND (normal diet).
-Individual raw data of Supplementary Figure 4, C57BL/6 F0, Glucose tolerance tests (GTT) and insulin tolerance tests (ITT, mg/dl) on HFD 21 % butter (high fat diet) or ND (normal diet).
-Individual raw data of Supplementary Figure 5 Balb/c F1, Body weight (g) ), male mice whose father was exposed to HFD 21% butter (high fat diet).
-Individual raw data of Supplementary Figure 5 Balb/c F1, Glucose tolerance tests (GTT) and insulin tolerance tests (ITT, mg/dl).
-Individual raw data of Supplementary Figure 6, B6/D2 F1, Body weight (g) ), male mice whose father was exposed to HFD 21% butter (high fat diet).
-Individual raw data of Supplementary Figure 6, B6/D2 F1, Glucose tolerance tests (GTT) and insulin tolerance tests (ITT, mg/dl).
-Individual raw data of Supplementary Figure 7, Dnmt2-/-C57BL/6 F1, Body weight (g) ), male mice whose father was exposed to HFD 21% butter (high fat diet).
-Individual raw data of Supplementary Figure 7, Dnmt2-/-C57BL/6 F1, Glucose tolerance tests (GTT) and insulin tolerance tests (ITT, mg/dl).
-Individual raw data of Supplementary Figure 8, C57BL/6 F1, Body weight (g) ), male mice whose father was exposed to HFD 21% butter (high fat diet).
-Individual raw data of Supplementary Figure 8, C57BL/6 F1, Glucose tolerance tests (GTT) and insulin tolerance tests (ITT, mg/dl).
-Individual raw data of Supplementary Figure 9, Balb/c F2, Body weight (g), male mice whose grand-father was exposed to HFD 21% butter (high fat diet).
-Individual raw data of Supplementary Figure 9, Balb/c F2, Glucose tolerance tests (GTT) and insulin tolerance tests (ITT, mg/dl).
-Individual raw data Supplementary Figure 10, B6D2 F2, Body weight (g), male mice whose grand-father was exposed to HFD 21% butter (high fat diet).
-Individual raw data Supplementary Figure 10, B6D2 F2, Glucose tolerance tests (GTT) and insulin tolerance tests (ITT, mg/dl).
-Individual raw data of Supplementary Figure 11, Dnmt2-/-C57BL/6 F2, Body weight (g), male mice whose grand-father was exposed to HFD 21% butter (high fat diet).
-Individual raw data of Supplementary Figure 11, Dnmt2-/-C57BL/6 F2, Glucose tolerance tests (GTT) and insulin tolerance tests (ITT, mg/dl).
-Individual raw data of Supplementary Figure 12, C57BL/6 F2, Body weight (g), male mice whose grand-father was exposed to HFD 21% butter (high fat diet).
-Individual raw data of Supplementary Figure 12, C57BL/6 F2, Glucose tolerance tests (GTT) and insulin tolerance tests (ITT, mg/dl).

## Slide 40
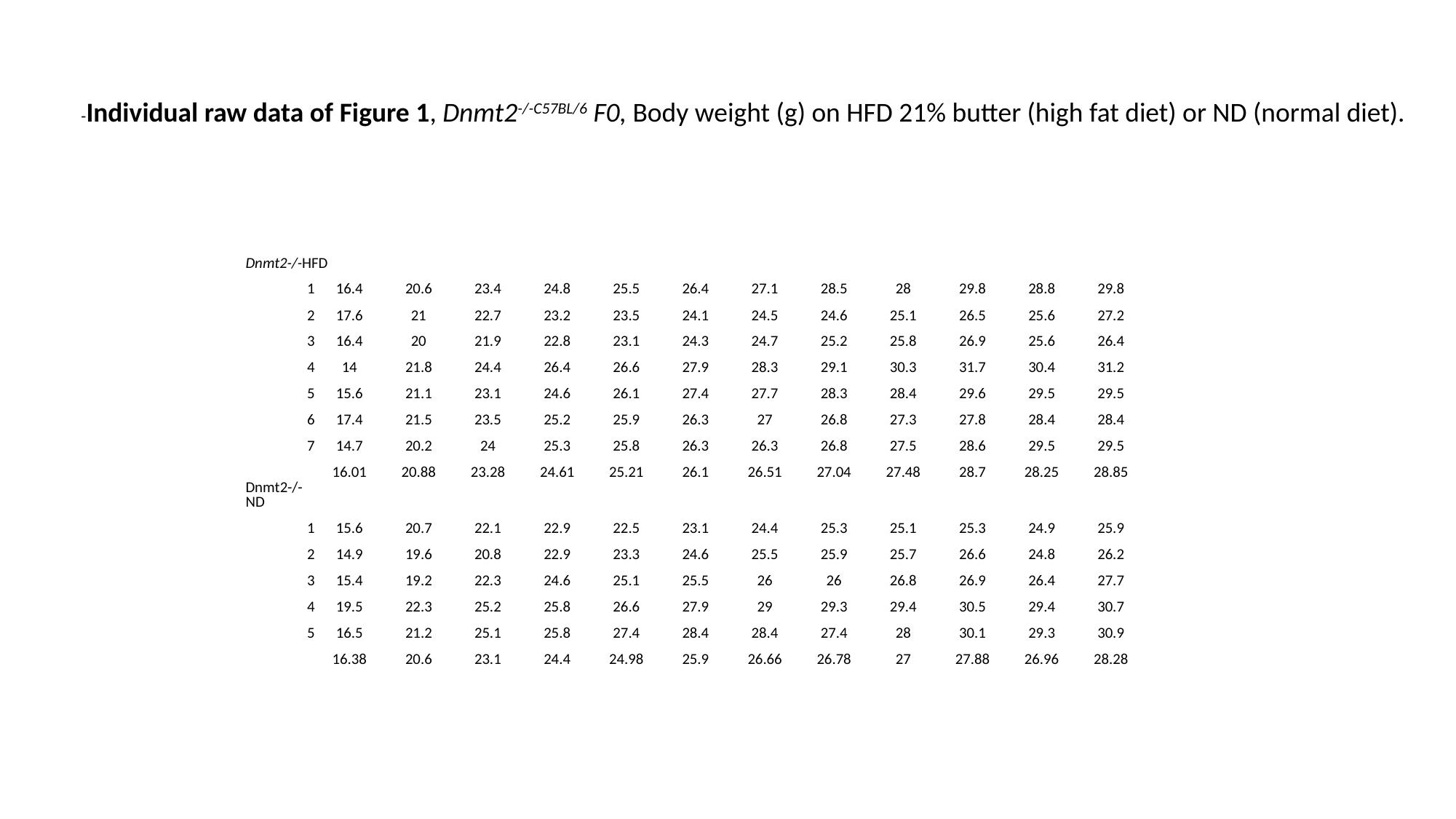

-Individual raw data of Figure 1, Dnmt2-/-C57BL/6 F0, Body weight (g) on HFD 21% butter (high fat diet) or ND (normal diet).
| Dnmt2-/-HFD | | | | | | | | | | | | |
| --- | --- | --- | --- | --- | --- | --- | --- | --- | --- | --- | --- | --- |
| 1 | 16.4 | 20.6 | 23.4 | 24.8 | 25.5 | 26.4 | 27.1 | 28.5 | 28 | 29.8 | 28.8 | 29.8 |
| 2 | 17.6 | 21 | 22.7 | 23.2 | 23.5 | 24.1 | 24.5 | 24.6 | 25.1 | 26.5 | 25.6 | 27.2 |
| 3 | 16.4 | 20 | 21.9 | 22.8 | 23.1 | 24.3 | 24.7 | 25.2 | 25.8 | 26.9 | 25.6 | 26.4 |
| 4 | 14 | 21.8 | 24.4 | 26.4 | 26.6 | 27.9 | 28.3 | 29.1 | 30.3 | 31.7 | 30.4 | 31.2 |
| 5 | 15.6 | 21.1 | 23.1 | 24.6 | 26.1 | 27.4 | 27.7 | 28.3 | 28.4 | 29.6 | 29.5 | 29.5 |
| 6 | 17.4 | 21.5 | 23.5 | 25.2 | 25.9 | 26.3 | 27 | 26.8 | 27.3 | 27.8 | 28.4 | 28.4 |
| 7 | 14.7 | 20.2 | 24 | 25.3 | 25.8 | 26.3 | 26.3 | 26.8 | 27.5 | 28.6 | 29.5 | 29.5 |
| | 16.01 | 20.88 | 23.28 | 24.61 | 25.21 | 26.1 | 26.51 | 27.04 | 27.48 | 28.7 | 28.25 | 28.85 |
| Dnmt2-/-ND | | | | | | | | | | | | |
| 1 | 15.6 | 20.7 | 22.1 | 22.9 | 22.5 | 23.1 | 24.4 | 25.3 | 25.1 | 25.3 | 24.9 | 25.9 |
| 2 | 14.9 | 19.6 | 20.8 | 22.9 | 23.3 | 24.6 | 25.5 | 25.9 | 25.7 | 26.6 | 24.8 | 26.2 |
| 3 | 15.4 | 19.2 | 22.3 | 24.6 | 25.1 | 25.5 | 26 | 26 | 26.8 | 26.9 | 26.4 | 27.7 |
| 4 | 19.5 | 22.3 | 25.2 | 25.8 | 26.6 | 27.9 | 29 | 29.3 | 29.4 | 30.5 | 29.4 | 30.7 |
| 5 | 16.5 | 21.2 | 25.1 | 25.8 | 27.4 | 28.4 | 28.4 | 27.4 | 28 | 30.1 | 29.3 | 30.9 |
| | 16.38 | 20.6 | 23.1 | 24.4 | 24.98 | 25.9 | 26.66 | 26.78 | 27 | 27.88 | 26.96 | 28.28 |

## Slide 41
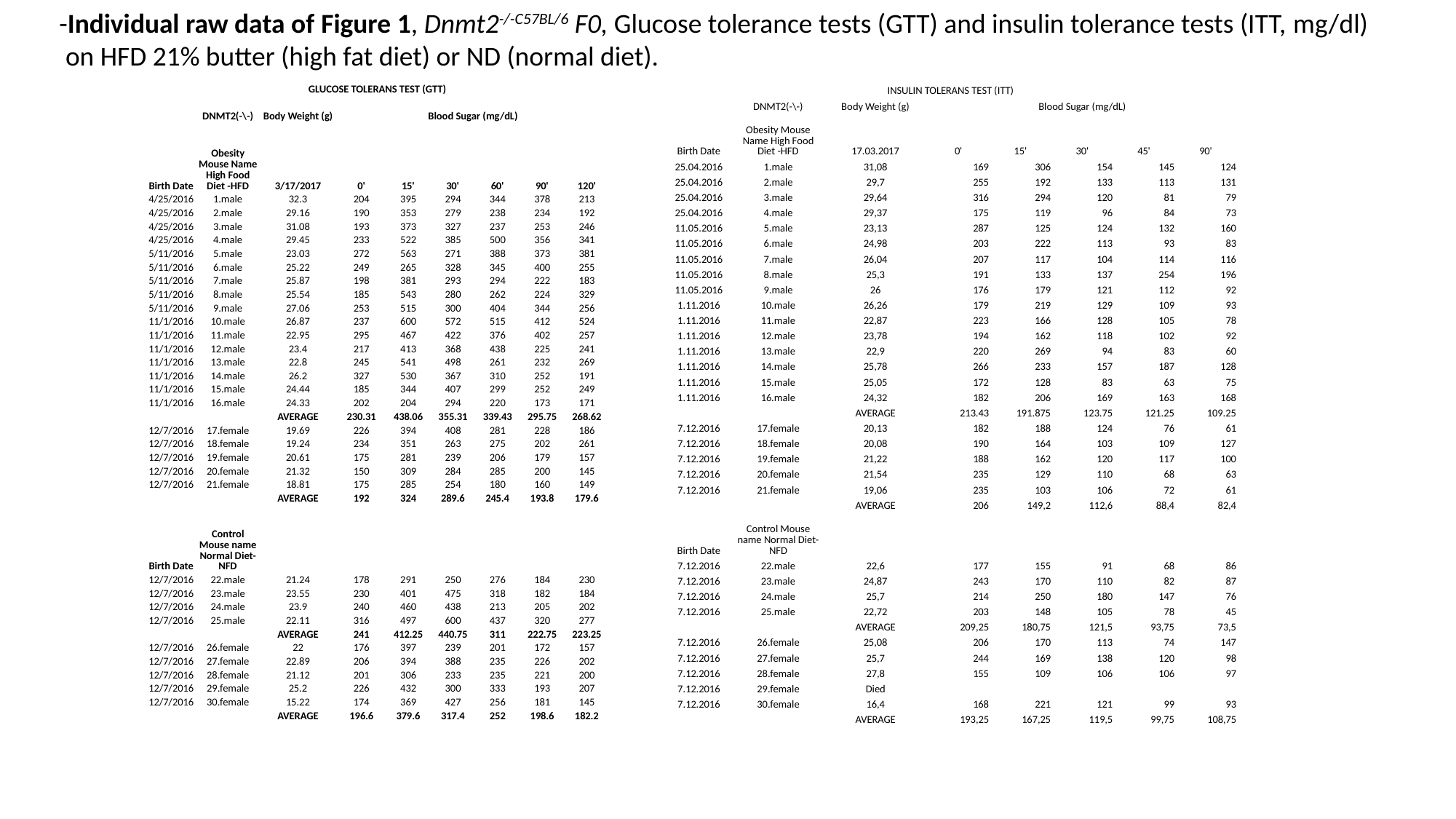

-Individual raw data of Figure 1, Dnmt2-/-C57BL/6 F0, Glucose tolerance tests (GTT) and insulin tolerance tests (ITT, mg/dl)
 on HFD 21% butter (high fat diet) or ND (normal diet).
| GLUCOSE TOLERANS TEST (GTT) | | | | | | | | |
| --- | --- | --- | --- | --- | --- | --- | --- | --- |
| | DNMT2(-\-) | Body Weight (g) | Blood Sugar (mg/dL) | | | | | |
| Birth Date | Obesity Mouse Name High Food Diet -HFD | 3/17/2017 | 0' | 15' | 30' | 60' | 90' | 120' |
| 4/25/2016 | 1.male | 32.3 | 204 | 395 | 294 | 344 | 378 | 213 |
| 4/25/2016 | 2.male | 29.16 | 190 | 353 | 279 | 238 | 234 | 192 |
| 4/25/2016 | 3.male | 31.08 | 193 | 373 | 327 | 237 | 253 | 246 |
| 4/25/2016 | 4.male | 29.45 | 233 | 522 | 385 | 500 | 356 | 341 |
| 5/11/2016 | 5.male | 23.03 | 272 | 563 | 271 | 388 | 373 | 381 |
| 5/11/2016 | 6.male | 25.22 | 249 | 265 | 328 | 345 | 400 | 255 |
| 5/11/2016 | 7.male | 25.87 | 198 | 381 | 293 | 294 | 222 | 183 |
| 5/11/2016 | 8.male | 25.54 | 185 | 543 | 280 | 262 | 224 | 329 |
| 5/11/2016 | 9.male | 27.06 | 253 | 515 | 300 | 404 | 344 | 256 |
| 11/1/2016 | 10.male | 26.87 | 237 | 600 | 572 | 515 | 412 | 524 |
| 11/1/2016 | 11.male | 22.95 | 295 | 467 | 422 | 376 | 402 | 257 |
| 11/1/2016 | 12.male | 23.4 | 217 | 413 | 368 | 438 | 225 | 241 |
| 11/1/2016 | 13.male | 22.8 | 245 | 541 | 498 | 261 | 232 | 269 |
| 11/1/2016 | 14.male | 26.2 | 327 | 530 | 367 | 310 | 252 | 191 |
| 11/1/2016 | 15.male | 24.44 | 185 | 344 | 407 | 299 | 252 | 249 |
| 11/1/2016 | 16.male | 24.33 | 202 | 204 | 294 | 220 | 173 | 171 |
| | | AVERAGE | 230.31 | 438.06 | 355.31 | 339.43 | 295.75 | 268.62 |
| 12/7/2016 | 17.female | 19.69 | 226 | 394 | 408 | 281 | 228 | 186 |
| 12/7/2016 | 18.female | 19.24 | 234 | 351 | 263 | 275 | 202 | 261 |
| 12/7/2016 | 19.female | 20.61 | 175 | 281 | 239 | 206 | 179 | 157 |
| 12/7/2016 | 20.female | 21.32 | 150 | 309 | 284 | 285 | 200 | 145 |
| 12/7/2016 | 21.female | 18.81 | 175 | 285 | 254 | 180 | 160 | 149 |
| | | AVERAGE | 192 | 324 | 289.6 | 245.4 | 193.8 | 179.6 |
| Birth Date | Control Mouse name Normal Diet- NFD | | | | | | | |
| 12/7/2016 | 22.male | 21.24 | 178 | 291 | 250 | 276 | 184 | 230 |
| 12/7/2016 | 23.male | 23.55 | 230 | 401 | 475 | 318 | 182 | 184 |
| 12/7/2016 | 24.male | 23.9 | 240 | 460 | 438 | 213 | 205 | 202 |
| 12/7/2016 | 25.male | 22.11 | 316 | 497 | 600 | 437 | 320 | 277 |
| | | AVERAGE | 241 | 412.25 | 440.75 | 311 | 222.75 | 223.25 |
| 12/7/2016 | 26.female | 22 | 176 | 397 | 239 | 201 | 172 | 157 |
| 12/7/2016 | 27.female | 22.89 | 206 | 394 | 388 | 235 | 226 | 202 |
| 12/7/2016 | 28.female | 21.12 | 201 | 306 | 233 | 235 | 221 | 200 |
| 12/7/2016 | 29.female | 25.2 | 226 | 432 | 300 | 333 | 193 | 207 |
| 12/7/2016 | 30.female | 15.22 | 174 | 369 | 427 | 256 | 181 | 145 |
| | | AVERAGE | 196.6 | 379.6 | 317.4 | 252 | 198.6 | 182.2 |
| | | | | | | | | |
| INSULIN TOLERANS TEST (ITT) | | | | | | | |
| --- | --- | --- | --- | --- | --- | --- | --- |
| | DNMT2(-\-) | Body Weight (g) | Blood Sugar (mg/dL) | | | | |
| Birth Date | Obesity Mouse Name High Food Diet -HFD | 17.03.2017 | 0' | 15' | 30' | 45' | 90' |
| 25.04.2016 | 1.male | 31,08 | 169 | 306 | 154 | 145 | 124 |
| 25.04.2016 | 2.male | 29,7 | 255 | 192 | 133 | 113 | 131 |
| 25.04.2016 | 3.male | 29,64 | 316 | 294 | 120 | 81 | 79 |
| 25.04.2016 | 4.male | 29,37 | 175 | 119 | 96 | 84 | 73 |
| 11.05.2016 | 5.male | 23,13 | 287 | 125 | 124 | 132 | 160 |
| 11.05.2016 | 6.male | 24,98 | 203 | 222 | 113 | 93 | 83 |
| 11.05.2016 | 7.male | 26,04 | 207 | 117 | 104 | 114 | 116 |
| 11.05.2016 | 8.male | 25,3 | 191 | 133 | 137 | 254 | 196 |
| 11.05.2016 | 9.male | 26 | 176 | 179 | 121 | 112 | 92 |
| 1.11.2016 | 10.male | 26,26 | 179 | 219 | 129 | 109 | 93 |
| 1.11.2016 | 11.male | 22,87 | 223 | 166 | 128 | 105 | 78 |
| 1.11.2016 | 12.male | 23,78 | 194 | 162 | 118 | 102 | 92 |
| 1.11.2016 | 13.male | 22,9 | 220 | 269 | 94 | 83 | 60 |
| 1.11.2016 | 14.male | 25,78 | 266 | 233 | 157 | 187 | 128 |
| 1.11.2016 | 15.male | 25,05 | 172 | 128 | 83 | 63 | 75 |
| 1.11.2016 | 16.male | 24,32 | 182 | 206 | 169 | 163 | 168 |
| | | AVERAGE | 213.43 | 191.875 | 123.75 | 121.25 | 109.25 |
| 7.12.2016 | 17.female | 20,13 | 182 | 188 | 124 | 76 | 61 |
| 7.12.2016 | 18.female | 20,08 | 190 | 164 | 103 | 109 | 127 |
| 7.12.2016 | 19.female | 21,22 | 188 | 162 | 120 | 117 | 100 |
| 7.12.2016 | 20.female | 21,54 | 235 | 129 | 110 | 68 | 63 |
| 7.12.2016 | 21.female | 19,06 | 235 | 103 | 106 | 72 | 61 |
| | | AVERAGE | 206 | 149,2 | 112,6 | 88,4 | 82,4 |
| Birth Date | Control Mouse name Normal Diet- NFD | | | | | | |
| 7.12.2016 | 22.male | 22,6 | 177 | 155 | 91 | 68 | 86 |
| 7.12.2016 | 23.male | 24,87 | 243 | 170 | 110 | 82 | 87 |
| 7.12.2016 | 24.male | 25,7 | 214 | 250 | 180 | 147 | 76 |
| 7.12.2016 | 25.male | 22,72 | 203 | 148 | 105 | 78 | 45 |
| | | AVERAGE | 209,25 | 180,75 | 121,5 | 93,75 | 73,5 |
| 7.12.2016 | 26.female | 25,08 | 206 | 170 | 113 | 74 | 147 |
| 7.12.2016 | 27.female | 25,7 | 244 | 169 | 138 | 120 | 98 |
| 7.12.2016 | 28.female | 27,8 | 155 | 109 | 106 | 106 | 97 |
| 7.12.2016 | 29.female | Died | | | | | |
| 7.12.2016 | 30.female | 16,4 | 168 | 221 | 121 | 99 | 93 |
| | | AVERAGE | 193,25 | 167,25 | 119,5 | 99,75 | 108,75 |

## Slide 42
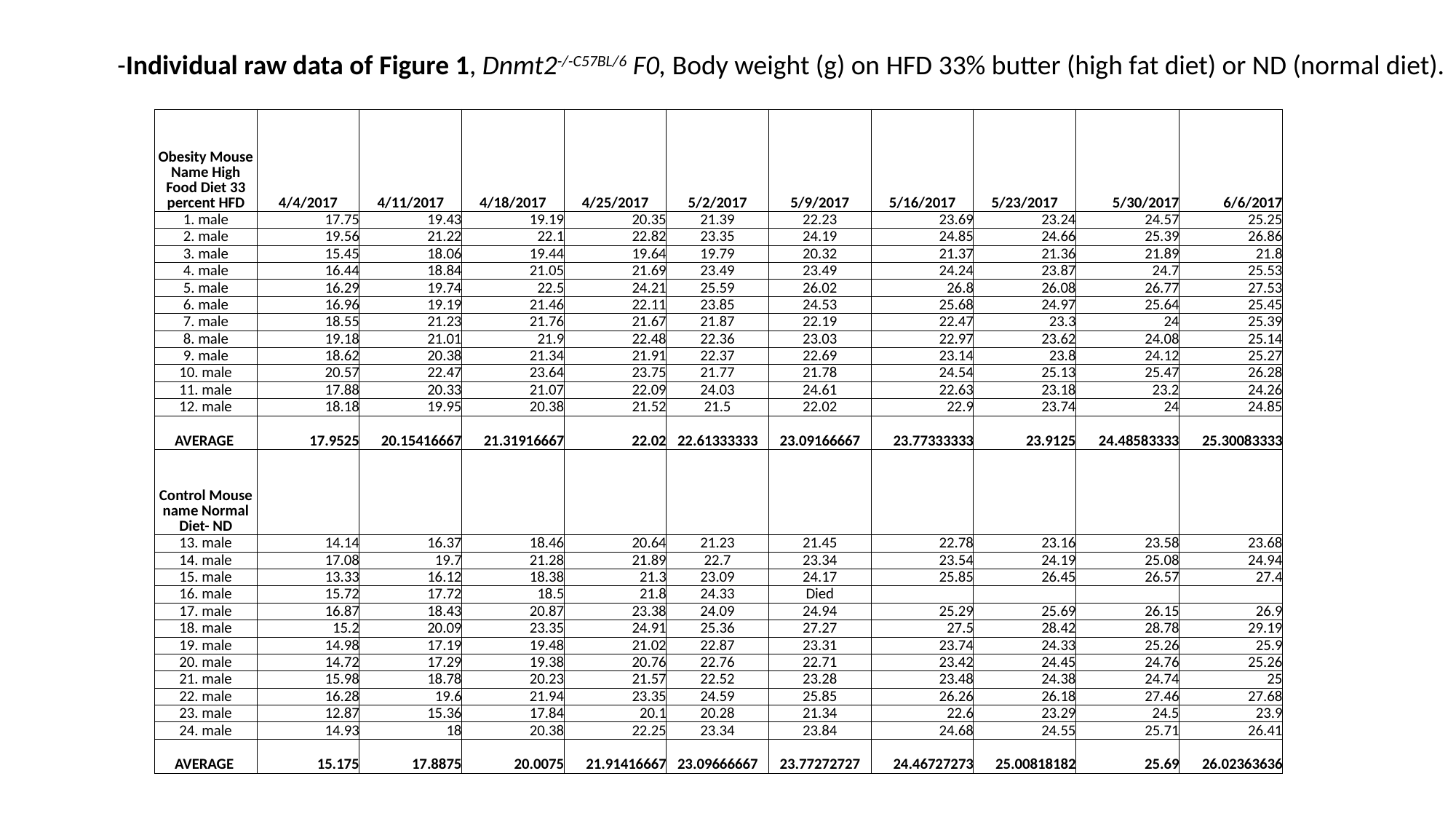

-Individual raw data of Figure 1, Dnmt2-/-C57BL/6 F0, Body weight (g) on HFD 33% butter (high fat diet) or ND (normal diet).
| Obesity Mouse Name High Food Diet 33 percent HFD | 4/4/2017 | 4/11/2017 | 4/18/2017 | 4/25/2017 | 5/2/2017 | 5/9/2017 | 5/16/2017 | 5/23/2017 | 5/30/2017 | 6/6/2017 |
| --- | --- | --- | --- | --- | --- | --- | --- | --- | --- | --- |
| 1. male | 17.75 | 19.43 | 19.19 | 20.35 | 21.39 | 22.23 | 23.69 | 23.24 | 24.57 | 25.25 |
| 2. male | 19.56 | 21.22 | 22.1 | 22.82 | 23.35 | 24.19 | 24.85 | 24.66 | 25.39 | 26.86 |
| 3. male | 15.45 | 18.06 | 19.44 | 19.64 | 19.79 | 20.32 | 21.37 | 21.36 | 21.89 | 21.8 |
| 4. male | 16.44 | 18.84 | 21.05 | 21.69 | 23.49 | 23.49 | 24.24 | 23.87 | 24.7 | 25.53 |
| 5. male | 16.29 | 19.74 | 22.5 | 24.21 | 25.59 | 26.02 | 26.8 | 26.08 | 26.77 | 27.53 |
| 6. male | 16.96 | 19.19 | 21.46 | 22.11 | 23.85 | 24.53 | 25.68 | 24.97 | 25.64 | 25.45 |
| 7. male | 18.55 | 21.23 | 21.76 | 21.67 | 21.87 | 22.19 | 22.47 | 23.3 | 24 | 25.39 |
| 8. male | 19.18 | 21.01 | 21.9 | 22.48 | 22.36 | 23.03 | 22.97 | 23.62 | 24.08 | 25.14 |
| 9. male | 18.62 | 20.38 | 21.34 | 21.91 | 22.37 | 22.69 | 23.14 | 23.8 | 24.12 | 25.27 |
| 10. male | 20.57 | 22.47 | 23.64 | 23.75 | 21.77 | 21.78 | 24.54 | 25.13 | 25.47 | 26.28 |
| 11. male | 17.88 | 20.33 | 21.07 | 22.09 | 24.03 | 24.61 | 22.63 | 23.18 | 23.2 | 24.26 |
| 12. male | 18.18 | 19.95 | 20.38 | 21.52 | 21.5 | 22.02 | 22.9 | 23.74 | 24 | 24.85 |
| AVERAGE | 17.9525 | 20.15416667 | 21.31916667 | 22.02 | 22.61333333 | 23.09166667 | 23.77333333 | 23.9125 | 24.48583333 | 25.30083333 |
| Control Mouse name Normal Diet- ND | | | | | | | | | | |
| 13. male | 14.14 | 16.37 | 18.46 | 20.64 | 21.23 | 21.45 | 22.78 | 23.16 | 23.58 | 23.68 |
| 14. male | 17.08 | 19.7 | 21.28 | 21.89 | 22.7 | 23.34 | 23.54 | 24.19 | 25.08 | 24.94 |
| 15. male | 13.33 | 16.12 | 18.38 | 21.3 | 23.09 | 24.17 | 25.85 | 26.45 | 26.57 | 27.4 |
| 16. male | 15.72 | 17.72 | 18.5 | 21.8 | 24.33 | Died | | | | |
| 17. male | 16.87 | 18.43 | 20.87 | 23.38 | 24.09 | 24.94 | 25.29 | 25.69 | 26.15 | 26.9 |
| 18. male | 15.2 | 20.09 | 23.35 | 24.91 | 25.36 | 27.27 | 27.5 | 28.42 | 28.78 | 29.19 |
| 19. male | 14.98 | 17.19 | 19.48 | 21.02 | 22.87 | 23.31 | 23.74 | 24.33 | 25.26 | 25.9 |
| 20. male | 14.72 | 17.29 | 19.38 | 20.76 | 22.76 | 22.71 | 23.42 | 24.45 | 24.76 | 25.26 |
| 21. male | 15.98 | 18.78 | 20.23 | 21.57 | 22.52 | 23.28 | 23.48 | 24.38 | 24.74 | 25 |
| 22. male | 16.28 | 19.6 | 21.94 | 23.35 | 24.59 | 25.85 | 26.26 | 26.18 | 27.46 | 27.68 |
| 23. male | 12.87 | 15.36 | 17.84 | 20.1 | 20.28 | 21.34 | 22.6 | 23.29 | 24.5 | 23.9 |
| 24. male | 14.93 | 18 | 20.38 | 22.25 | 23.34 | 23.84 | 24.68 | 24.55 | 25.71 | 26.41 |
| AVERAGE | 15.175 | 17.8875 | 20.0075 | 21.91416667 | 23.09666667 | 23.77272727 | 24.46727273 | 25.00818182 | 25.69 | 26.02363636 |

## Slide 43
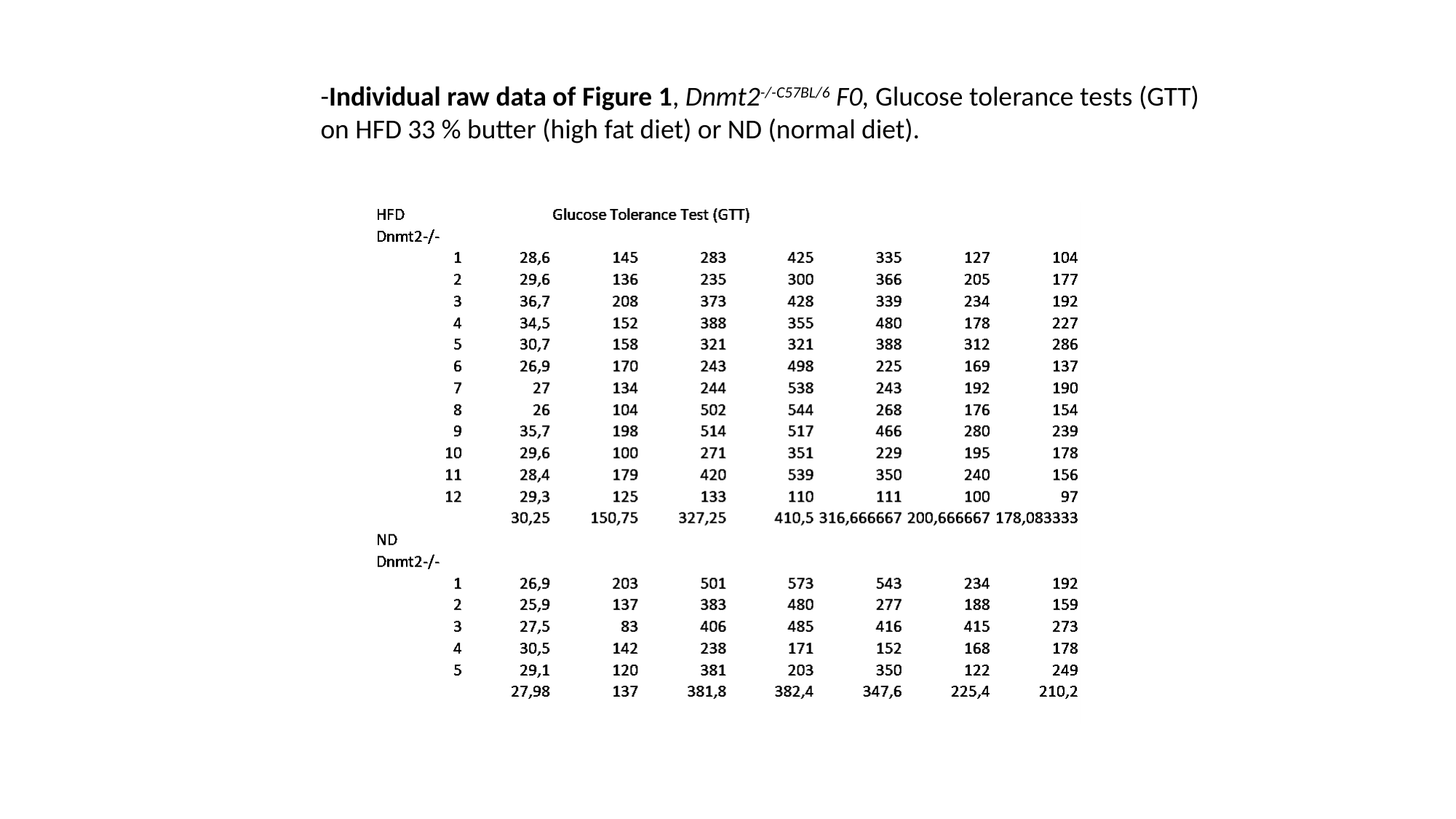

-Individual raw data of Figure 1, Dnmt2-/-C57BL/6 F0, Glucose tolerance tests (GTT)
on HFD 33 % butter (high fat diet) or ND (normal diet).

## Slide 44
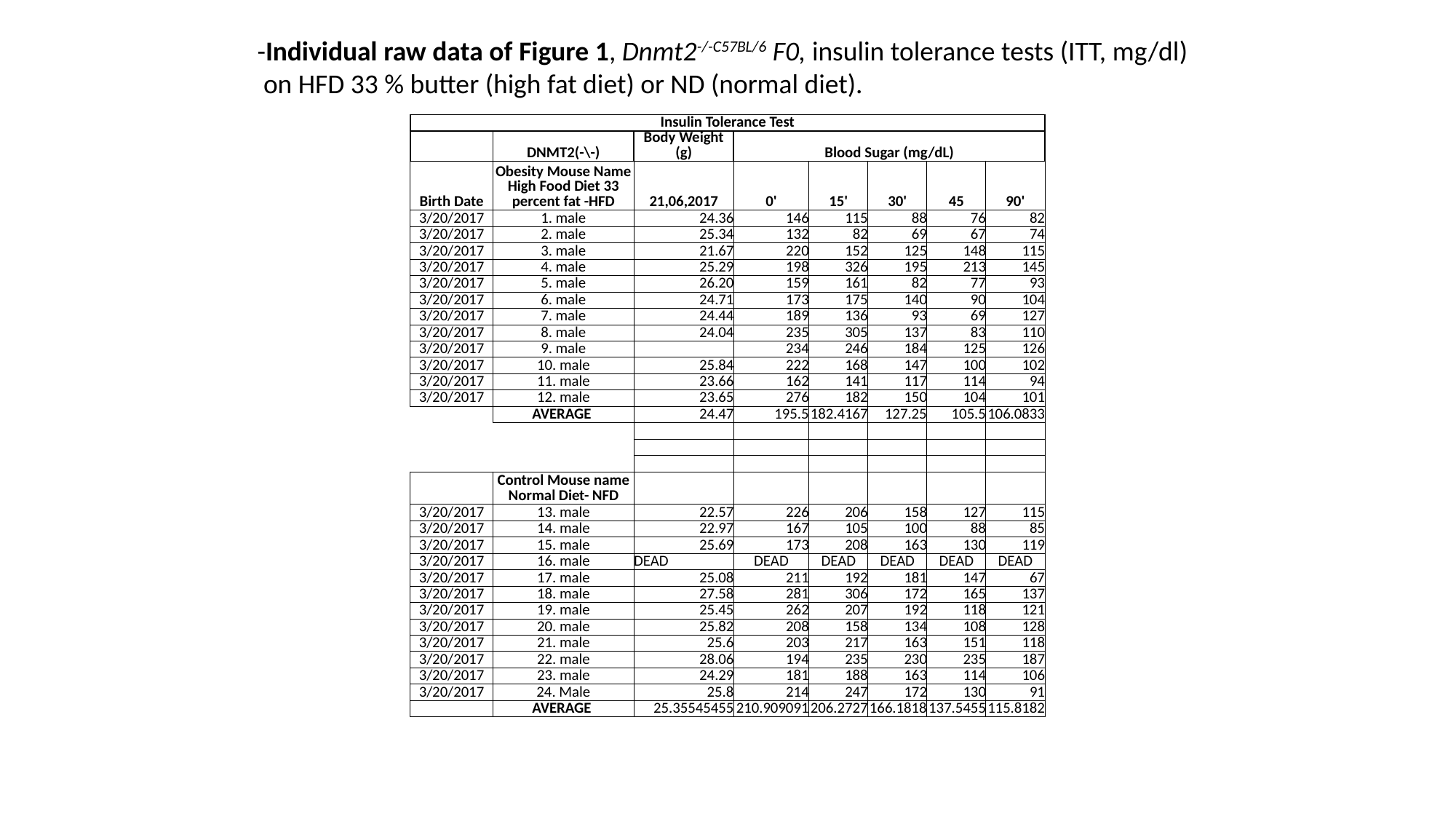

-Individual raw data of Figure 1, Dnmt2-/-C57BL/6 F0, insulin tolerance tests (ITT, mg/dl)
 on HFD 33 % butter (high fat diet) or ND (normal diet).
| Insulin Tolerance Test | | | | | | | |
| --- | --- | --- | --- | --- | --- | --- | --- |
| | DNMT2(-\-) | Body Weight (g) | Blood Sugar (mg/dL) | | | | |
| Birth Date | Obesity Mouse Name High Food Diet 33 percent fat -HFD | 21,06,2017 | 0' | 15' | 30' | 45 | 90' |
| 3/20/2017 | 1. male | 24.36 | 146 | 115 | 88 | 76 | 82 |
| 3/20/2017 | 2. male | 25.34 | 132 | 82 | 69 | 67 | 74 |
| 3/20/2017 | 3. male | 21.67 | 220 | 152 | 125 | 148 | 115 |
| 3/20/2017 | 4. male | 25.29 | 198 | 326 | 195 | 213 | 145 |
| 3/20/2017 | 5. male | 26.20 | 159 | 161 | 82 | 77 | 93 |
| 3/20/2017 | 6. male | 24.71 | 173 | 175 | 140 | 90 | 104 |
| 3/20/2017 | 7. male | 24.44 | 189 | 136 | 93 | 69 | 127 |
| 3/20/2017 | 8. male | 24.04 | 235 | 305 | 137 | 83 | 110 |
| 3/20/2017 | 9. male | | 234 | 246 | 184 | 125 | 126 |
| 3/20/2017 | 10. male | 25.84 | 222 | 168 | 147 | 100 | 102 |
| 3/20/2017 | 11. male | 23.66 | 162 | 141 | 117 | 114 | 94 |
| 3/20/2017 | 12. male | 23.65 | 276 | 182 | 150 | 104 | 101 |
| | AVERAGE | 24.47 | 195.5 | 182.4167 | 127.25 | 105.5 | 106.0833 |
| | | | | | | | |
| | | | | | | | |
| | | | | | | | |
| | Control Mouse name Normal Diet- NFD | | | | | | |
| 3/20/2017 | 13. male | 22.57 | 226 | 206 | 158 | 127 | 115 |
| 3/20/2017 | 14. male | 22.97 | 167 | 105 | 100 | 88 | 85 |
| 3/20/2017 | 15. male | 25.69 | 173 | 208 | 163 | 130 | 119 |
| 3/20/2017 | 16. male | DEAD | DEAD | DEAD | DEAD | DEAD | DEAD |
| 3/20/2017 | 17. male | 25.08 | 211 | 192 | 181 | 147 | 67 |
| 3/20/2017 | 18. male | 27.58 | 281 | 306 | 172 | 165 | 137 |
| 3/20/2017 | 19. male | 25.45 | 262 | 207 | 192 | 118 | 121 |
| 3/20/2017 | 20. male | 25.82 | 208 | 158 | 134 | 108 | 128 |
| 3/20/2017 | 21. male | 25.6 | 203 | 217 | 163 | 151 | 118 |
| 3/20/2017 | 22. male | 28.06 | 194 | 235 | 230 | 235 | 187 |
| 3/20/2017 | 23. male | 24.29 | 181 | 188 | 163 | 114 | 106 |
| 3/20/2017 | 24. Male | 25.8 | 214 | 247 | 172 | 130 | 91 |
| | AVERAGE | 25.35545455 | 210.909091 | 206.2727 | 166.1818 | 137.5455 | 115.8182 |

## Slide 45
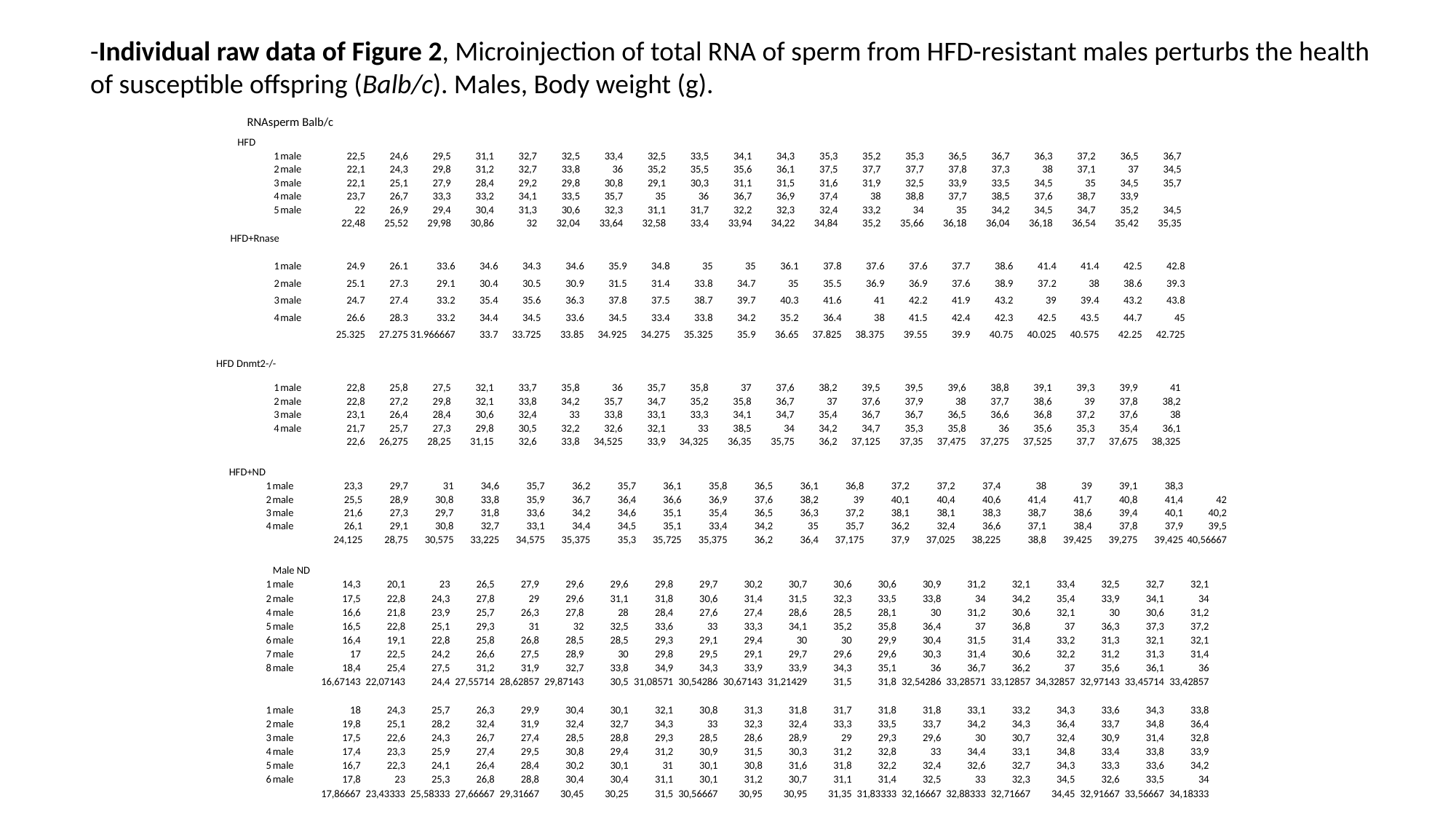

-Individual raw data of Figure 2, Microinjection of total RNA of sperm from HFD-resistant males perturbs the health
of susceptible offspring (Balb/c). Males, Body weight (g).
RNAsperm Balb/c
| HFD | | | | | | | | | | | | | | | | | | | | | |
| --- | --- | --- | --- | --- | --- | --- | --- | --- | --- | --- | --- | --- | --- | --- | --- | --- | --- | --- | --- | --- | --- |
| 1 | male | 22,5 | 24,6 | 29,5 | 31,1 | 32,7 | 32,5 | 33,4 | 32,5 | 33,5 | 34,1 | 34,3 | 35,3 | 35,2 | 35,3 | 36,5 | 36,7 | 36,3 | 37,2 | 36,5 | 36,7 |
| 2 | male | 22,1 | 24,3 | 29,8 | 31,2 | 32,7 | 33,8 | 36 | 35,2 | 35,5 | 35,6 | 36,1 | 37,5 | 37,7 | 37,7 | 37,8 | 37,3 | 38 | 37,1 | 37 | 34,5 |
| 3 | male | 22,1 | 25,1 | 27,9 | 28,4 | 29,2 | 29,8 | 30,8 | 29,1 | 30,3 | 31,1 | 31,5 | 31,6 | 31,9 | 32,5 | 33,9 | 33,5 | 34,5 | 35 | 34,5 | 35,7 |
| 4 | male | 23,7 | 26,7 | 33,3 | 33,2 | 34,1 | 33,5 | 35,7 | 35 | 36 | 36,7 | 36,9 | 37,4 | 38 | 38,8 | 37,7 | 38,5 | 37,6 | 38,7 | 33,9 | |
| 5 | male | 22 | 26,9 | 29,4 | 30,4 | 31,3 | 30,6 | 32,3 | 31,1 | 31,7 | 32,2 | 32,3 | 32,4 | 33,2 | 34 | 35 | 34,2 | 34,5 | 34,7 | 35,2 | 34,5 |
| | | 22,48 | 25,52 | 29,98 | 30,86 | 32 | 32,04 | 33,64 | 32,58 | 33,4 | 33,94 | 34,22 | 34,84 | 35,2 | 35,66 | 36,18 | 36,04 | 36,18 | 36,54 | 35,42 | 35,35 |
| HFD+Rnase | | | | | | | | | | | | | | | | | | | | | |
| --- | --- | --- | --- | --- | --- | --- | --- | --- | --- | --- | --- | --- | --- | --- | --- | --- | --- | --- | --- | --- | --- |
| 1 | male | 24.9 | 26.1 | 33.6 | 34.6 | 34.3 | 34.6 | 35.9 | 34.8 | 35 | 35 | 36.1 | 37.8 | 37.6 | 37.6 | 37.7 | 38.6 | 41.4 | 41.4 | 42.5 | 42.8 |
| 2 | male | 25.1 | 27.3 | 29.1 | 30.4 | 30.5 | 30.9 | 31.5 | 31.4 | 33.8 | 34.7 | 35 | 35.5 | 36.9 | 36.9 | 37.6 | 38.9 | 37.2 | 38 | 38.6 | 39.3 |
| 3 | male | 24.7 | 27.4 | 33.2 | 35.4 | 35.6 | 36.3 | 37.8 | 37.5 | 38.7 | 39.7 | 40.3 | 41.6 | 41 | 42.2 | 41.9 | 43.2 | 39 | 39.4 | 43.2 | 43.8 |
| 4 | male | 26.6 | 28.3 | 33.2 | 34.4 | 34.5 | 33.6 | 34.5 | 33.4 | 33.8 | 34.2 | 35.2 | 36.4 | 38 | 41.5 | 42.4 | 42.3 | 42.5 | 43.5 | 44.7 | 45 |
| | | 25.325 | 27.275 | 31.966667 | 33.7 | 33.725 | 33.85 | 34.925 | 34.275 | 35.325 | 35.9 | 36.65 | 37.825 | 38.375 | 39.55 | 39.9 | 40.75 | 40.025 | 40.575 | 42.25 | 42.725 |
| HFD Dnmt2-/- | | | | | | | | | | | | | | | | | | | | | |
| --- | --- | --- | --- | --- | --- | --- | --- | --- | --- | --- | --- | --- | --- | --- | --- | --- | --- | --- | --- | --- | --- |
| 1 | male | 22,8 | 25,8 | 27,5 | 32,1 | 33,7 | 35,8 | 36 | 35,7 | 35,8 | 37 | 37,6 | 38,2 | 39,5 | 39,5 | 39,6 | 38,8 | 39,1 | 39,3 | 39,9 | 41 |
| 2 | male | 22,8 | 27,2 | 29,8 | 32,1 | 33,8 | 34,2 | 35,7 | 34,7 | 35,2 | 35,8 | 36,7 | 37 | 37,6 | 37,9 | 38 | 37,7 | 38,6 | 39 | 37,8 | 38,2 |
| 3 | male | 23,1 | 26,4 | 28,4 | 30,6 | 32,4 | 33 | 33,8 | 33,1 | 33,3 | 34,1 | 34,7 | 35,4 | 36,7 | 36,7 | 36,5 | 36,6 | 36,8 | 37,2 | 37,6 | 38 |
| 4 | male | 21,7 | 25,7 | 27,3 | 29,8 | 30,5 | 32,2 | 32,6 | 32,1 | 33 | 38,5 | 34 | 34,2 | 34,7 | 35,3 | 35,8 | 36 | 35,6 | 35,3 | 35,4 | 36,1 |
| | | 22,6 | 26,275 | 28,25 | 31,15 | 32,6 | 33,8 | 34,525 | 33,9 | 34,325 | 36,35 | 35,75 | 36,2 | 37,125 | 37,35 | 37,475 | 37,275 | 37,525 | 37,7 | 37,675 | 38,325 |
| HFD+ND | | | | | | | | | | | | | | | | | | | | | |
| --- | --- | --- | --- | --- | --- | --- | --- | --- | --- | --- | --- | --- | --- | --- | --- | --- | --- | --- | --- | --- | --- |
| 1 | male | 23,3 | 29,7 | 31 | 34,6 | 35,7 | 36,2 | 35,7 | 36,1 | 35,8 | 36,5 | 36,1 | 36,8 | 37,2 | 37,2 | 37,4 | 38 | 39 | 39,1 | 38,3 | |
| 2 | male | 25,5 | 28,9 | 30,8 | 33,8 | 35,9 | 36,7 | 36,4 | 36,6 | 36,9 | 37,6 | 38,2 | 39 | 40,1 | 40,4 | 40,6 | 41,4 | 41,7 | 40,8 | 41,4 | 42 |
| 3 | male | 21,6 | 27,3 | 29,7 | 31,8 | 33,6 | 34,2 | 34,6 | 35,1 | 35,4 | 36,5 | 36,3 | 37,2 | 38,1 | 38,1 | 38,3 | 38,7 | 38,6 | 39,4 | 40,1 | 40,2 |
| 4 | male | 26,1 | 29,1 | 30,8 | 32,7 | 33,1 | 34,4 | 34,5 | 35,1 | 33,4 | 34,2 | 35 | 35,7 | 36,2 | 32,4 | 36,6 | 37,1 | 38,4 | 37,8 | 37,9 | 39,5 |
| | | 24,125 | 28,75 | 30,575 | 33,225 | 34,575 | 35,375 | 35,3 | 35,725 | 35,375 | 36,2 | 36,4 | 37,175 | 37,9 | 37,025 | 38,225 | 38,8 | 39,425 | 39,275 | 39,425 | 40,56667 |
| | | | | | | | | | | | | | | | | | | | | | |
| --- | --- | --- | --- | --- | --- | --- | --- | --- | --- | --- | --- | --- | --- | --- | --- | --- | --- | --- | --- | --- | --- |
| | Male ND | | | | | | | | | | | | | | | | | | | | |
| 1 | male | 14,3 | 20,1 | 23 | 26,5 | 27,9 | 29,6 | 29,6 | 29,8 | 29,7 | 30,2 | 30,7 | 30,6 | 30,6 | 30,9 | 31,2 | 32,1 | 33,4 | 32,5 | 32,7 | 32,1 |
| 2 | male | 17,5 | 22,8 | 24,3 | 27,8 | 29 | 29,6 | 31,1 | 31,8 | 30,6 | 31,4 | 31,5 | 32,3 | 33,5 | 33,8 | 34 | 34,2 | 35,4 | 33,9 | 34,1 | 34 |
| 4 | male | 16,6 | 21,8 | 23,9 | 25,7 | 26,3 | 27,8 | 28 | 28,4 | 27,6 | 27,4 | 28,6 | 28,5 | 28,1 | 30 | 31,2 | 30,6 | 32,1 | 30 | 30,6 | 31,2 |
| 5 | male | 16,5 | 22,8 | 25,1 | 29,3 | 31 | 32 | 32,5 | 33,6 | 33 | 33,3 | 34,1 | 35,2 | 35,8 | 36,4 | 37 | 36,8 | 37 | 36,3 | 37,3 | 37,2 |
| 6 | male | 16,4 | 19,1 | 22,8 | 25,8 | 26,8 | 28,5 | 28,5 | 29,3 | 29,1 | 29,4 | 30 | 30 | 29,9 | 30,4 | 31,5 | 31,4 | 33,2 | 31,3 | 32,1 | 32,1 |
| 7 | male | 17 | 22,5 | 24,2 | 26,6 | 27,5 | 28,9 | 30 | 29,8 | 29,5 | 29,1 | 29,7 | 29,6 | 29,6 | 30,3 | 31,4 | 30,6 | 32,2 | 31,2 | 31,3 | 31,4 |
| 8 | male | 18,4 | 25,4 | 27,5 | 31,2 | 31,9 | 32,7 | 33,8 | 34,9 | 34,3 | 33,9 | 33,9 | 34,3 | 35,1 | 36 | 36,7 | 36,2 | 37 | 35,6 | 36,1 | 36 |
| | | 16,67143 | 22,07143 | 24,4 | 27,55714 | 28,62857 | 29,87143 | 30,5 | 31,08571 | 30,54286 | 30,67143 | 31,21429 | 31,5 | 31,8 | 32,54286 | 33,28571 | 33,12857 | 34,32857 | 32,97143 | 33,45714 | 33,42857 |
| | | | | | | | | | | | | | | | | | | | | | |
| 1 | male | 18 | 24,3 | 25,7 | 26,3 | 29,9 | 30,4 | 30,1 | 32,1 | 30,8 | 31,3 | 31,8 | 31,7 | 31,8 | 31,8 | 33,1 | 33,2 | 34,3 | 33,6 | 34,3 | 33,8 |
| 2 | male | 19,8 | 25,1 | 28,2 | 32,4 | 31,9 | 32,4 | 32,7 | 34,3 | 33 | 32,3 | 32,4 | 33,3 | 33,5 | 33,7 | 34,2 | 34,3 | 36,4 | 33,7 | 34,8 | 36,4 |
| 3 | male | 17,5 | 22,6 | 24,3 | 26,7 | 27,4 | 28,5 | 28,8 | 29,3 | 28,5 | 28,6 | 28,9 | 29 | 29,3 | 29,6 | 30 | 30,7 | 32,4 | 30,9 | 31,4 | 32,8 |
| 4 | male | 17,4 | 23,3 | 25,9 | 27,4 | 29,5 | 30,8 | 29,4 | 31,2 | 30,9 | 31,5 | 30,3 | 31,2 | 32,8 | 33 | 34,4 | 33,1 | 34,8 | 33,4 | 33,8 | 33,9 |
| 5 | male | 16,7 | 22,3 | 24,1 | 26,4 | 28,4 | 30,2 | 30,1 | 31 | 30,1 | 30,8 | 31,6 | 31,8 | 32,2 | 32,4 | 32,6 | 32,7 | 34,3 | 33,3 | 33,6 | 34,2 |
| 6 | male | 17,8 | 23 | 25,3 | 26,8 | 28,8 | 30,4 | 30,4 | 31,1 | 30,1 | 31,2 | 30,7 | 31,1 | 31,4 | 32,5 | 33 | 32,3 | 34,5 | 32,6 | 33,5 | 34 |
| | | 17,86667 | 23,43333 | 25,58333 | 27,66667 | 29,31667 | 30,45 | 30,25 | 31,5 | 30,56667 | 30,95 | 30,95 | 31,35 | 31,83333 | 32,16667 | 32,88333 | 32,71667 | 34,45 | 32,91667 | 33,56667 | 34,18333 |

## Slide 46
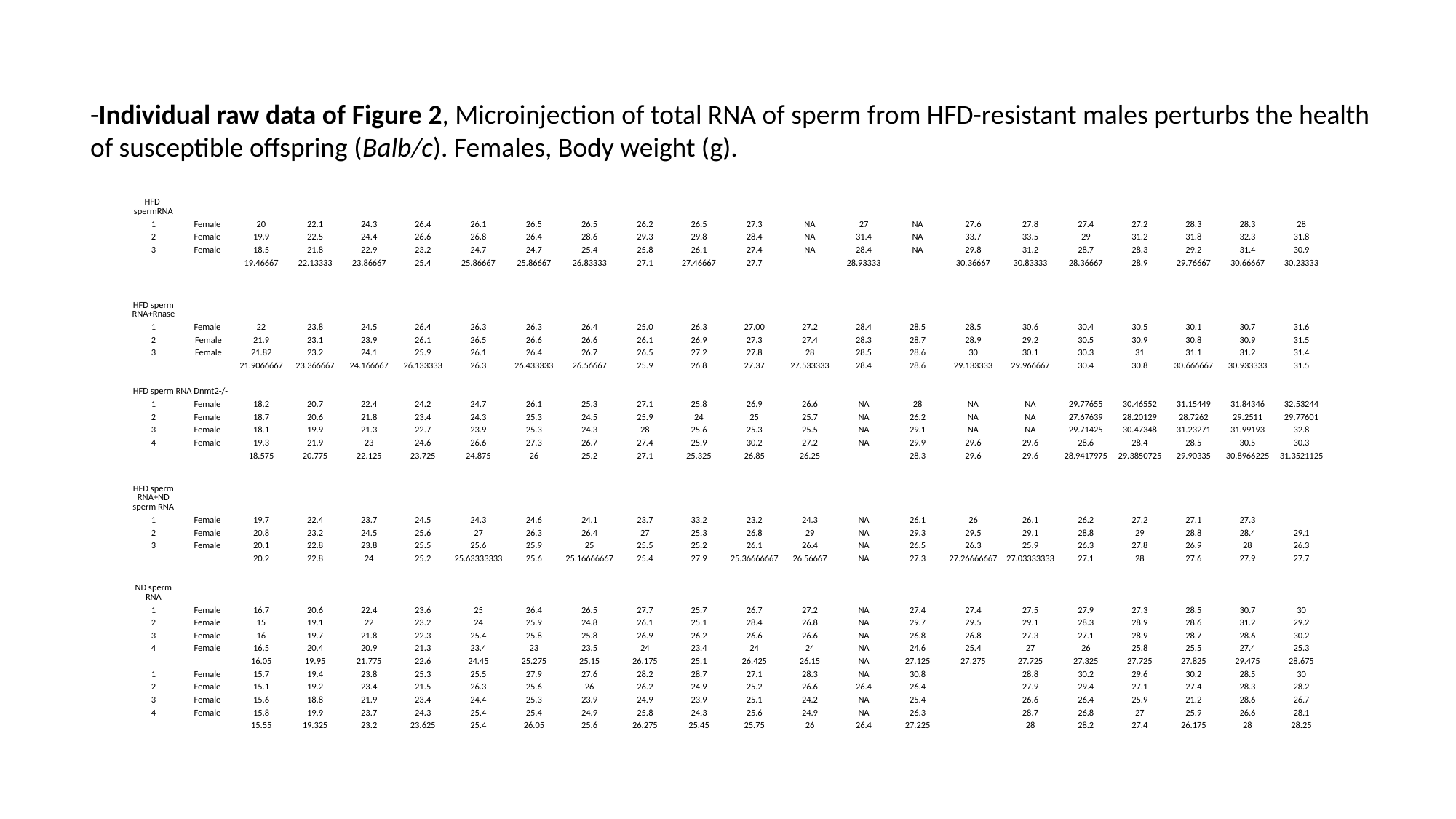

-Individual raw data of Figure 2, Microinjection of total RNA of sperm from HFD-resistant males perturbs the health
of susceptible offspring (Balb/c). Females, Body weight (g).
| HFD-spermRNA | | | | | | | | | | | | | | | | | | | | | |
| --- | --- | --- | --- | --- | --- | --- | --- | --- | --- | --- | --- | --- | --- | --- | --- | --- | --- | --- | --- | --- | --- |
| 1 | Female | 20 | 22.1 | 24.3 | 26.4 | 26.1 | 26.5 | 26.5 | 26.2 | 26.5 | 27.3 | NA | 27 | NA | 27.6 | 27.8 | 27.4 | 27.2 | 28.3 | 28.3 | 28 |
| 2 | Female | 19.9 | 22.5 | 24.4 | 26.6 | 26.8 | 26.4 | 28.6 | 29.3 | 29.8 | 28.4 | NA | 31.4 | NA | 33.7 | 33.5 | 29 | 31.2 | 31.8 | 32.3 | 31.8 |
| 3 | Female | 18.5 | 21.8 | 22.9 | 23.2 | 24.7 | 24.7 | 25.4 | 25.8 | 26.1 | 27.4 | NA | 28.4 | NA | 29.8 | 31.2 | 28.7 | 28.3 | 29.2 | 31.4 | 30.9 |
| | | 19.46667 | 22.13333 | 23.86667 | 25.4 | 25.86667 | 25.86667 | 26.83333 | 27.1 | 27.46667 | 27.7 | | 28.93333 | | 30.36667 | 30.83333 | 28.36667 | 28.9 | 29.76667 | 30.66667 | 30.23333 |
| | | | | | | | | | | | | | | | | | | | | | |
| | | | | | | | | | | | | | | | | | | | | | |
| HFD sperm RNA+Rnase | | | | | | | | | | | | | | | | | | | | | |
| 1 | Female | 22 | 23.8 | 24.5 | 26.4 | 26.3 | 26.3 | 26.4 | 25.0 | 26.3 | 27.00 | 27.2 | 28.4 | 28.5 | 28.5 | 30.6 | 30.4 | 30.5 | 30.1 | 30.7 | 31.6 |
| 2 | Female | 21.9 | 23.1 | 23.9 | 26.1 | 26.5 | 26.6 | 26.6 | 26.1 | 26.9 | 27.3 | 27.4 | 28.3 | 28.7 | 28.9 | 29.2 | 30.5 | 30.9 | 30.8 | 30.9 | 31.5 |
| 3 | Female | 21.82 | 23.2 | 24.1 | 25.9 | 26.1 | 26.4 | 26.7 | 26.5 | 27.2 | 27.8 | 28 | 28.5 | 28.6 | 30 | 30.1 | 30.3 | 31 | 31.1 | 31.2 | 31.4 |
| | | 21.9066667 | 23.366667 | 24.166667 | 26.133333 | 26.3 | 26.433333 | 26.56667 | 25.9 | 26.8 | 27.37 | 27.533333 | 28.4 | 28.6 | 29.133333 | 29.966667 | 30.4 | 30.8 | 30.666667 | 30.933333 | 31.5 |
| | | | | | | | | | | | | | | | | | | | | | |
| HFD sperm RNA Dnmt2-/- | | | | | | | | | | | | | | | | | | | | | |
| 1 | Female | 18.2 | 20.7 | 22.4 | 24.2 | 24.7 | 26.1 | 25.3 | 27.1 | 25.8 | 26.9 | 26.6 | NA | 28 | NA | NA | 29.77655 | 30.46552 | 31.15449 | 31.84346 | 32.53244 |
| 2 | Female | 18.7 | 20.6 | 21.8 | 23.4 | 24.3 | 25.3 | 24.5 | 25.9 | 24 | 25 | 25.7 | NA | 26.2 | NA | NA | 27.67639 | 28.20129 | 28.7262 | 29.2511 | 29.77601 |
| 3 | Female | 18.1 | 19.9 | 21.3 | 22.7 | 23.9 | 25.3 | 24.3 | 28 | 25.6 | 25.3 | 25.5 | NA | 29.1 | NA | NA | 29.71425 | 30.47348 | 31.23271 | 31.99193 | 32.8 |
| 4 | Female | 19.3 | 21.9 | 23 | 24.6 | 26.6 | 27.3 | 26.7 | 27.4 | 25.9 | 30.2 | 27.2 | NA | 29.9 | 29.6 | 29.6 | 28.6 | 28.4 | 28.5 | 30.5 | 30.3 |
| | | 18.575 | 20.775 | 22.125 | 23.725 | 24.875 | 26 | 25.2 | 27.1 | 25.325 | 26.85 | 26.25 | | 28.3 | 29.6 | 29.6 | 28.9417975 | 29.3850725 | 29.90335 | 30.8966225 | 31.3521125 |
| | | | | | | | | | | | | | | | | | | | | | |
| HFD sperm RNA+ND sperm RNA | | | | | | | | | | | | | | | | | | | | | |
| 1 | Female | 19.7 | 22.4 | 23.7 | 24.5 | 24.3 | 24.6 | 24.1 | 23.7 | 33.2 | 23.2 | 24.3 | NA | 26.1 | 26 | 26.1 | 26.2 | 27.2 | 27.1 | 27.3 | |
| 2 | Female | 20.8 | 23.2 | 24.5 | 25.6 | 27 | 26.3 | 26.4 | 27 | 25.3 | 26.8 | 29 | NA | 29.3 | 29.5 | 29.1 | 28.8 | 29 | 28.8 | 28.4 | 29.1 |
| 3 | Female | 20.1 | 22.8 | 23.8 | 25.5 | 25.6 | 25.9 | 25 | 25.5 | 25.2 | 26.1 | 26.4 | NA | 26.5 | 26.3 | 25.9 | 26.3 | 27.8 | 26.9 | 28 | 26.3 |
| | | 20.2 | 22.8 | 24 | 25.2 | 25.63333333 | 25.6 | 25.16666667 | 25.4 | 27.9 | 25.36666667 | 26.56667 | NA | 27.3 | 27.26666667 | 27.03333333 | 27.1 | 28 | 27.6 | 27.9 | 27.7 |
| | | | | | | | | | | | | | | | | | | | | | |
| ND sperm RNA | | | | | | | | | | | | | | | | | | | | | |
| 1 | Female | 16.7 | 20.6 | 22.4 | 23.6 | 25 | 26.4 | 26.5 | 27.7 | 25.7 | 26.7 | 27.2 | NA | 27.4 | 27.4 | 27.5 | 27.9 | 27.3 | 28.5 | 30.7 | 30 |
| 2 | Female | 15 | 19.1 | 22 | 23.2 | 24 | 25.9 | 24.8 | 26.1 | 25.1 | 28.4 | 26.8 | NA | 29.7 | 29.5 | 29.1 | 28.3 | 28.9 | 28.6 | 31.2 | 29.2 |
| 3 | Female | 16 | 19.7 | 21.8 | 22.3 | 25.4 | 25.8 | 25.8 | 26.9 | 26.2 | 26.6 | 26.6 | NA | 26.8 | 26.8 | 27.3 | 27.1 | 28.9 | 28.7 | 28.6 | 30.2 |
| 4 | Female | 16.5 | 20.4 | 20.9 | 21.3 | 23.4 | 23 | 23.5 | 24 | 23.4 | 24 | 24 | NA | 24.6 | 25.4 | 27 | 26 | 25.8 | 25.5 | 27.4 | 25.3 |
| | | 16.05 | 19.95 | 21.775 | 22.6 | 24.45 | 25.275 | 25.15 | 26.175 | 25.1 | 26.425 | 26.15 | NA | 27.125 | 27.275 | 27.725 | 27.325 | 27.725 | 27.825 | 29.475 | 28.675 |
| 1 | Female | 15.7 | 19.4 | 23.8 | 25.3 | 25.5 | 27.9 | 27.6 | 28.2 | 28.7 | 27.1 | 28.3 | NA | 30.8 | | 28.8 | 30.2 | 29.6 | 30.2 | 28.5 | 30 |
| 2 | Female | 15.1 | 19.2 | 23.4 | 21.5 | 26.3 | 25.6 | 26 | 26.2 | 24.9 | 25.2 | 26.6 | 26.4 | 26.4 | | 27.9 | 29.4 | 27.1 | 27.4 | 28.3 | 28.2 |
| 3 | Female | 15.6 | 18.8 | 21.9 | 23.4 | 24.4 | 25.3 | 23.9 | 24.9 | 23.9 | 25.1 | 24.2 | NA | 25.4 | | 26.6 | 26.4 | 25.9 | 21.2 | 28.6 | 26.7 |
| 4 | Female | 15.8 | 19.9 | 23.7 | 24.3 | 25.4 | 25.4 | 24.9 | 25.8 | 24.3 | 25.6 | 24.9 | NA | 26.3 | | 28.7 | 26.8 | 27 | 25.9 | 26.6 | 28.1 |
| | | 15.55 | 19.325 | 23.2 | 23.625 | 25.4 | 26.05 | 25.6 | 26.275 | 25.45 | 25.75 | 26 | 26.4 | 27.225 | | 28 | 28.2 | 27.4 | 26.175 | 28 | 28.25 |

## Slide 47
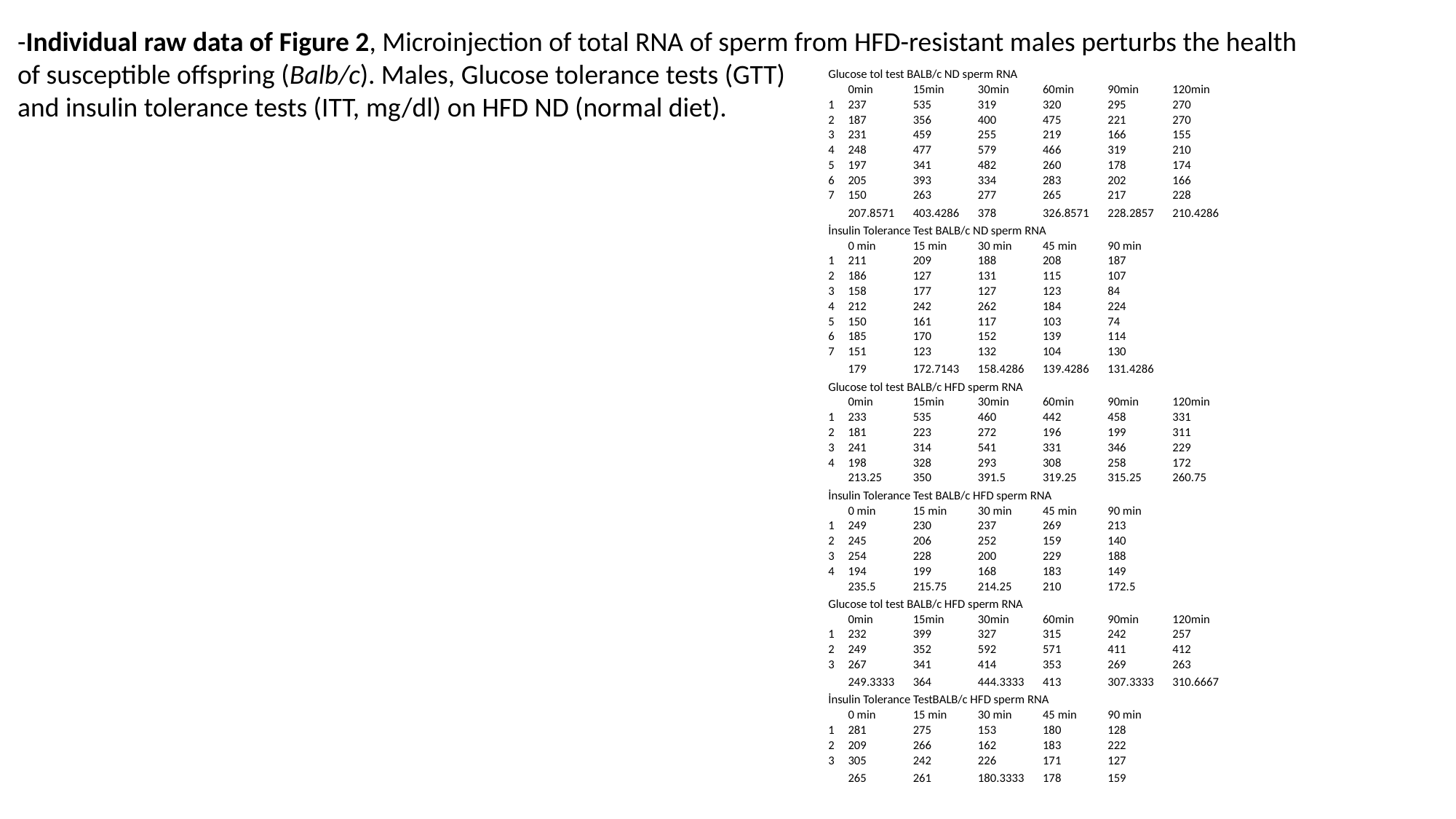

-Individual raw data of Figure 2, Microinjection of total RNA of sperm from HFD-resistant males perturbs the health
of susceptible offspring (Balb/c). Males, Glucose tolerance tests (GTT)
and insulin tolerance tests (ITT, mg/dl) on HFD ND (normal diet).
| Glucose tol test BALB/c ND sperm RNA | | | | | | |
| --- | --- | --- | --- | --- | --- | --- |
| | 0min | 15min | 30min | 60min | 90min | 120min |
| 1 | 237 | 535 | 319 | 320 | 295 | 270 |
| 2 | 187 | 356 | 400 | 475 | 221 | 270 |
| 3 | 231 | 459 | 255 | 219 | 166 | 155 |
| 4 | 248 | 477 | 579 | 466 | 319 | 210 |
| 5 | 197 | 341 | 482 | 260 | 178 | 174 |
| 6 | 205 | 393 | 334 | 283 | 202 | 166 |
| 7 | 150 | 263 | 277 | 265 | 217 | 228 |
| | 207.8571 | 403.4286 | 378 | 326.8571 | 228.2857 | 210.4286 |
| İnsulin Tolerance Test BALB/c ND sperm RNA | | | | | | |
| | 0 min | 15 min | 30 min | 45 min | 90 min | |
| 1 | 211 | 209 | 188 | 208 | 187 | |
| 2 | 186 | 127 | 131 | 115 | 107 | |
| 3 | 158 | 177 | 127 | 123 | 84 | |
| 4 | 212 | 242 | 262 | 184 | 224 | |
| 5 | 150 | 161 | 117 | 103 | 74 | |
| 6 | 185 | 170 | 152 | 139 | 114 | |
| 7 | 151 | 123 | 132 | 104 | 130 | |
| | 179 | 172.7143 | 158.4286 | 139.4286 | 131.4286 | |
| Glucose tol test BALB/c HFD sperm RNA | | | | | | |
| | 0min | 15min | 30min | 60min | 90min | 120min |
| 1 | 233 | 535 | 460 | 442 | 458 | 331 |
| 2 | 181 | 223 | 272 | 196 | 199 | 311 |
| 3 | 241 | 314 | 541 | 331 | 346 | 229 |
| 4 | 198 | 328 | 293 | 308 | 258 | 172 |
| | 213.25 | 350 | 391.5 | 319.25 | 315.25 | 260.75 |
| İnsulin Tolerance Test BALB/c HFD sperm RNA | | | | | | |
| | 0 min | 15 min | 30 min | 45 min | 90 min | |
| 1 | 249 | 230 | 237 | 269 | 213 | |
| 2 | 245 | 206 | 252 | 159 | 140 | |
| 3 | 254 | 228 | 200 | 229 | 188 | |
| 4 | 194 | 199 | 168 | 183 | 149 | |
| | 235.5 | 215.75 | 214.25 | 210 | 172.5 | |
| Glucose tol test BALB/c HFD sperm RNA | | | | | | |
| | 0min | 15min | 30min | 60min | 90min | 120min |
| 1 | 232 | 399 | 327 | 315 | 242 | 257 |
| 2 | 249 | 352 | 592 | 571 | 411 | 412 |
| 3 | 267 | 341 | 414 | 353 | 269 | 263 |
| | 249.3333 | 364 | 444.3333 | 413 | 307.3333 | 310.6667 |
| İnsulin Tolerance TestBALB/c HFD sperm RNA | | | | | | |
| | 0 min | 15 min | 30 min | 45 min | 90 min | |
| 1 | 281 | 275 | 153 | 180 | 128 | |
| 2 | 209 | 266 | 162 | 183 | 222 | |
| 3 | 305 | 242 | 226 | 171 | 127 | |
| | 265 | 261 | 180.3333 | 178 | 159 | |

## Slide 48
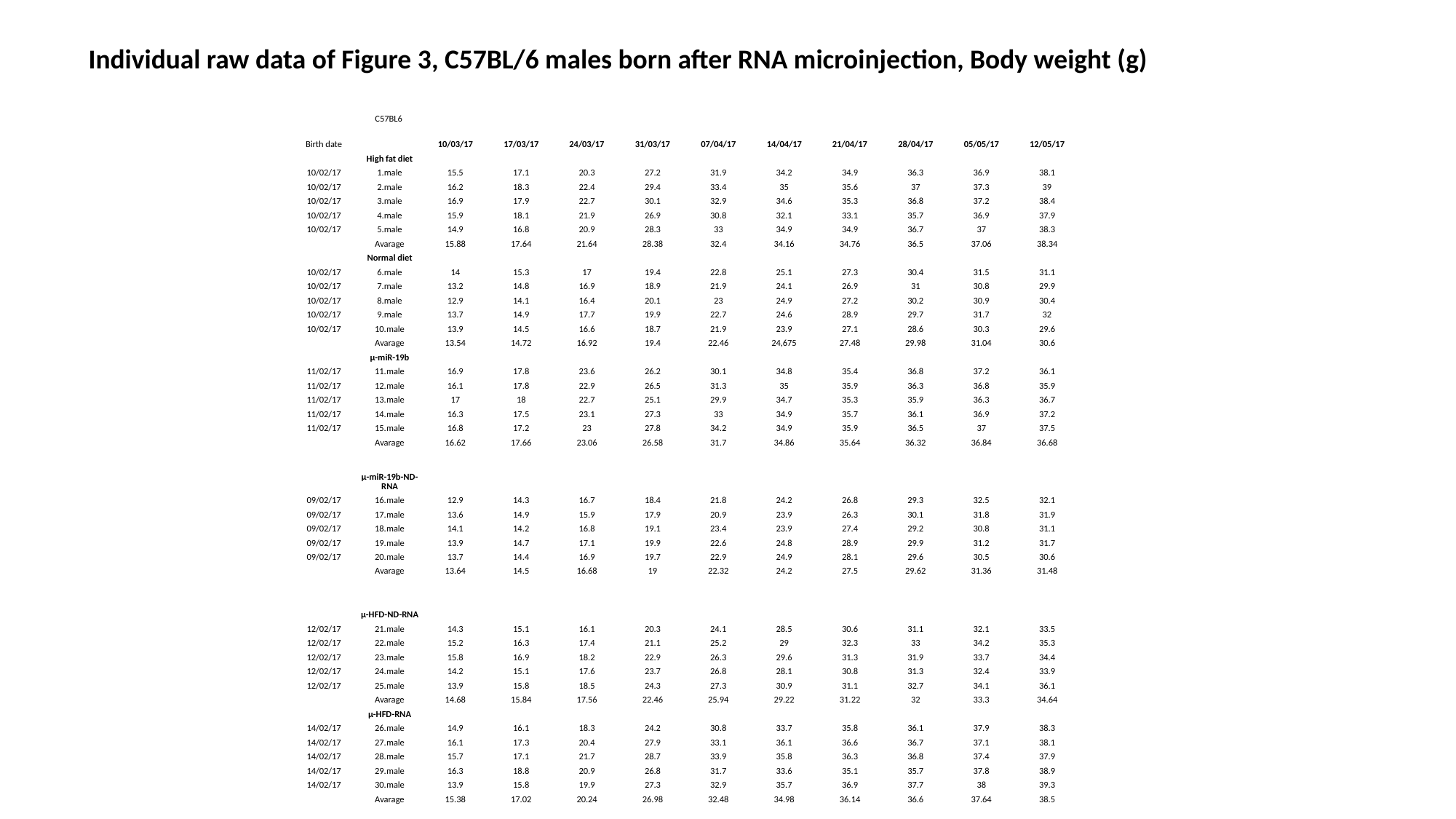

Individual raw data of Figure 3, C57BL/6 males born after RNA microinjection, Body weight (g)
| | C57BL6 | | | | | | | | | | |
| --- | --- | --- | --- | --- | --- | --- | --- | --- | --- | --- | --- |
| Birth date | | 10/03/17 | 17/03/17 | 24/03/17 | 31/03/17 | 07/04/17 | 14/04/17 | 21/04/17 | 28/04/17 | 05/05/17 | 12/05/17 |
| | High fat diet | | | | | | | | | | |
| 10/02/17 | 1.male | 15.5 | 17.1 | 20.3 | 27.2 | 31.9 | 34.2 | 34.9 | 36.3 | 36.9 | 38.1 |
| 10/02/17 | 2.male | 16.2 | 18.3 | 22.4 | 29.4 | 33.4 | 35 | 35.6 | 37 | 37.3 | 39 |
| 10/02/17 | 3.male | 16.9 | 17.9 | 22.7 | 30.1 | 32.9 | 34.6 | 35.3 | 36.8 | 37.2 | 38.4 |
| 10/02/17 | 4.male | 15.9 | 18.1 | 21.9 | 26.9 | 30.8 | 32.1 | 33.1 | 35.7 | 36.9 | 37.9 |
| 10/02/17 | 5.male | 14.9 | 16.8 | 20.9 | 28.3 | 33 | 34.9 | 34.9 | 36.7 | 37 | 38.3 |
| | Avarage | 15.88 | 17.64 | 21.64 | 28.38 | 32.4 | 34.16 | 34.76 | 36.5 | 37.06 | 38.34 |
| | Normal diet | | | | | | | | | | |
| 10/02/17 | 6.male | 14 | 15.3 | 17 | 19.4 | 22.8 | 25.1 | 27.3 | 30.4 | 31.5 | 31.1 |
| 10/02/17 | 7.male | 13.2 | 14.8 | 16.9 | 18.9 | 21.9 | 24.1 | 26.9 | 31 | 30.8 | 29.9 |
| 10/02/17 | 8.male | 12.9 | 14.1 | 16.4 | 20.1 | 23 | 24.9 | 27.2 | 30.2 | 30.9 | 30.4 |
| 10/02/17 | 9.male | 13.7 | 14.9 | 17.7 | 19.9 | 22.7 | 24.6 | 28.9 | 29.7 | 31.7 | 32 |
| 10/02/17 | 10.male | 13.9 | 14.5 | 16.6 | 18.7 | 21.9 | 23.9 | 27.1 | 28.6 | 30.3 | 29.6 |
| | Avarage | 13.54 | 14.72 | 16.92 | 19.4 | 22.46 | 24,675 | 27.48 | 29.98 | 31.04 | 30.6 |
| | µ-miR-19b | | | | | | | | | | |
| 11/02/17 | 11.male | 16.9 | 17.8 | 23.6 | 26.2 | 30.1 | 34.8 | 35.4 | 36.8 | 37.2 | 36.1 |
| 11/02/17 | 12.male | 16.1 | 17.8 | 22.9 | 26.5 | 31.3 | 35 | 35.9 | 36.3 | 36.8 | 35.9 |
| 11/02/17 | 13.male | 17 | 18 | 22.7 | 25.1 | 29.9 | 34.7 | 35.3 | 35.9 | 36.3 | 36.7 |
| 11/02/17 | 14.male | 16.3 | 17.5 | 23.1 | 27.3 | 33 | 34.9 | 35.7 | 36.1 | 36.9 | 37.2 |
| 11/02/17 | 15.male | 16.8 | 17.2 | 23 | 27.8 | 34.2 | 34.9 | 35.9 | 36.5 | 37 | 37.5 |
| | Avarage | 16.62 | 17.66 | 23.06 | 26.58 | 31.7 | 34.86 | 35.64 | 36.32 | 36.84 | 36.68 |
| | µ-miR-19b-ND-RNA | | | | | | | | | | |
| 09/02/17 | 16.male | 12.9 | 14.3 | 16.7 | 18.4 | 21.8 | 24.2 | 26.8 | 29.3 | 32.5 | 32.1 |
| 09/02/17 | 17.male | 13.6 | 14.9 | 15.9 | 17.9 | 20.9 | 23.9 | 26.3 | 30.1 | 31.8 | 31.9 |
| 09/02/17 | 18.male | 14.1 | 14.2 | 16.8 | 19.1 | 23.4 | 23.9 | 27.4 | 29.2 | 30.8 | 31.1 |
| 09/02/17 | 19.male | 13.9 | 14.7 | 17.1 | 19.9 | 22.6 | 24.8 | 28.9 | 29.9 | 31.2 | 31.7 |
| 09/02/17 | 20.male | 13.7 | 14.4 | 16.9 | 19.7 | 22.9 | 24.9 | 28.1 | 29.6 | 30.5 | 30.6 |
| | Avarage | 13.64 | 14.5 | 16.68 | 19 | 22.32 | 24.2 | 27.5 | 29.62 | 31.36 | 31.48 |
| | µ-HFD-ND-RNA | | | | | | | | | | |
| 12/02/17 | 21.male | 14.3 | 15.1 | 16.1 | 20.3 | 24.1 | 28.5 | 30.6 | 31.1 | 32.1 | 33.5 |
| 12/02/17 | 22.male | 15.2 | 16.3 | 17.4 | 21.1 | 25.2 | 29 | 32.3 | 33 | 34.2 | 35.3 |
| 12/02/17 | 23.male | 15.8 | 16.9 | 18.2 | 22.9 | 26.3 | 29.6 | 31.3 | 31.9 | 33.7 | 34.4 |
| 12/02/17 | 24.male | 14.2 | 15.1 | 17.6 | 23.7 | 26.8 | 28.1 | 30.8 | 31.3 | 32.4 | 33.9 |
| 12/02/17 | 25.male | 13.9 | 15.8 | 18.5 | 24.3 | 27.3 | 30.9 | 31.1 | 32.7 | 34.1 | 36.1 |
| | Avarage | 14.68 | 15.84 | 17.56 | 22.46 | 25.94 | 29.22 | 31.22 | 32 | 33.3 | 34.64 |
| | µ-HFD-RNA | | | | | | | | | | |
| 14/02/17 | 26.male | 14.9 | 16.1 | 18.3 | 24.2 | 30.8 | 33.7 | 35.8 | 36.1 | 37.9 | 38.3 |
| 14/02/17 | 27.male | 16.1 | 17.3 | 20.4 | 27.9 | 33.1 | 36.1 | 36.6 | 36.7 | 37.1 | 38.1 |
| 14/02/17 | 28.male | 15.7 | 17.1 | 21.7 | 28.7 | 33.9 | 35.8 | 36.3 | 36.8 | 37.4 | 37.9 |
| 14/02/17 | 29.male | 16.3 | 18.8 | 20.9 | 26.8 | 31.7 | 33.6 | 35.1 | 35.7 | 37.8 | 38.9 |
| 14/02/17 | 30.male | 13.9 | 15.8 | 19.9 | 27.3 | 32.9 | 35.7 | 36.9 | 37.7 | 38 | 39.3 |
| | Avarage | 15.38 | 17.02 | 20.24 | 26.98 | 32.48 | 34.98 | 36.14 | 36.6 | 37.64 | 38.5 |

## Slide 49
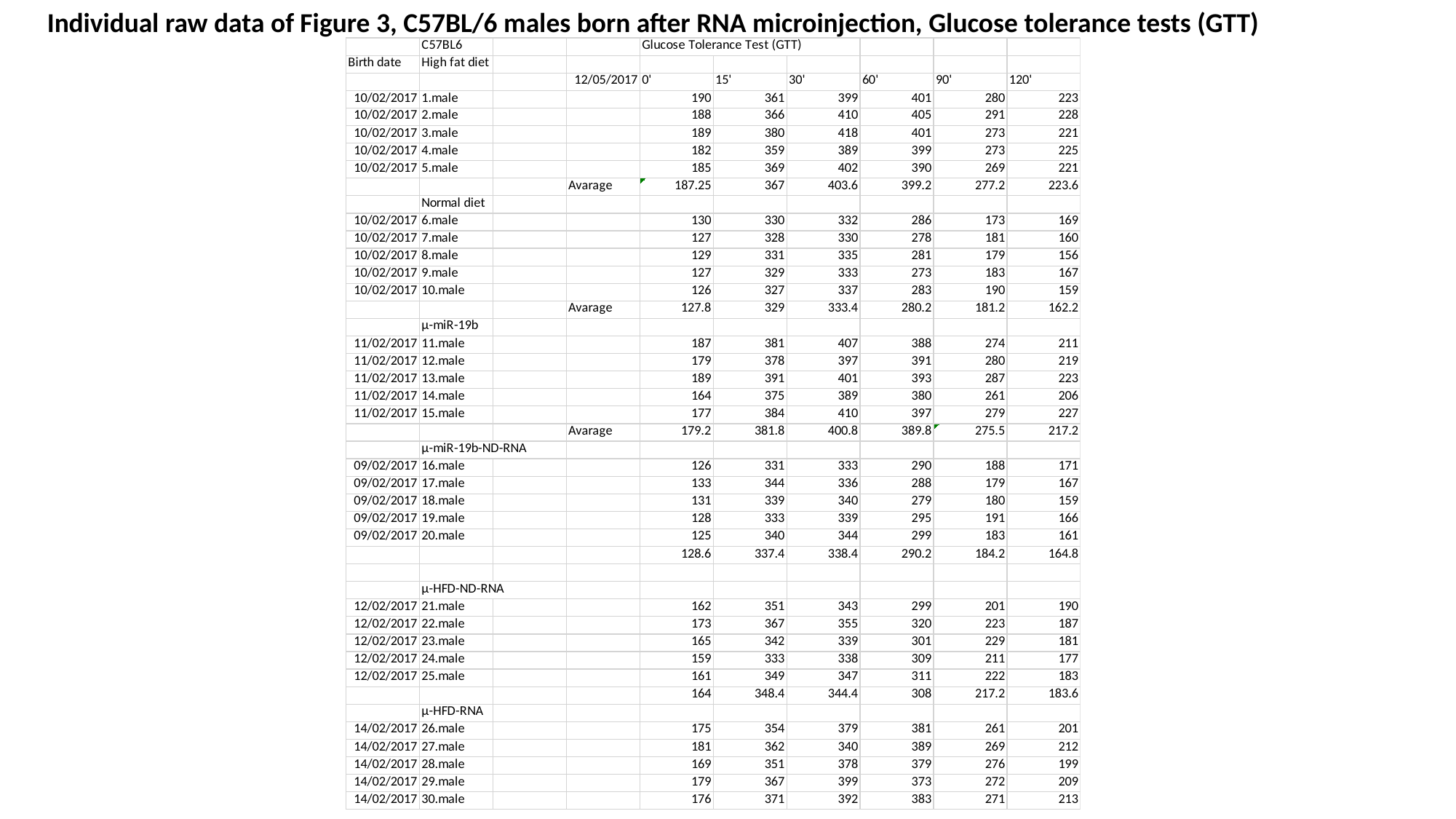

Individual raw data of Figure 3, C57BL/6 males born after RNA microinjection, Glucose tolerance tests (GTT)

## Slide 50
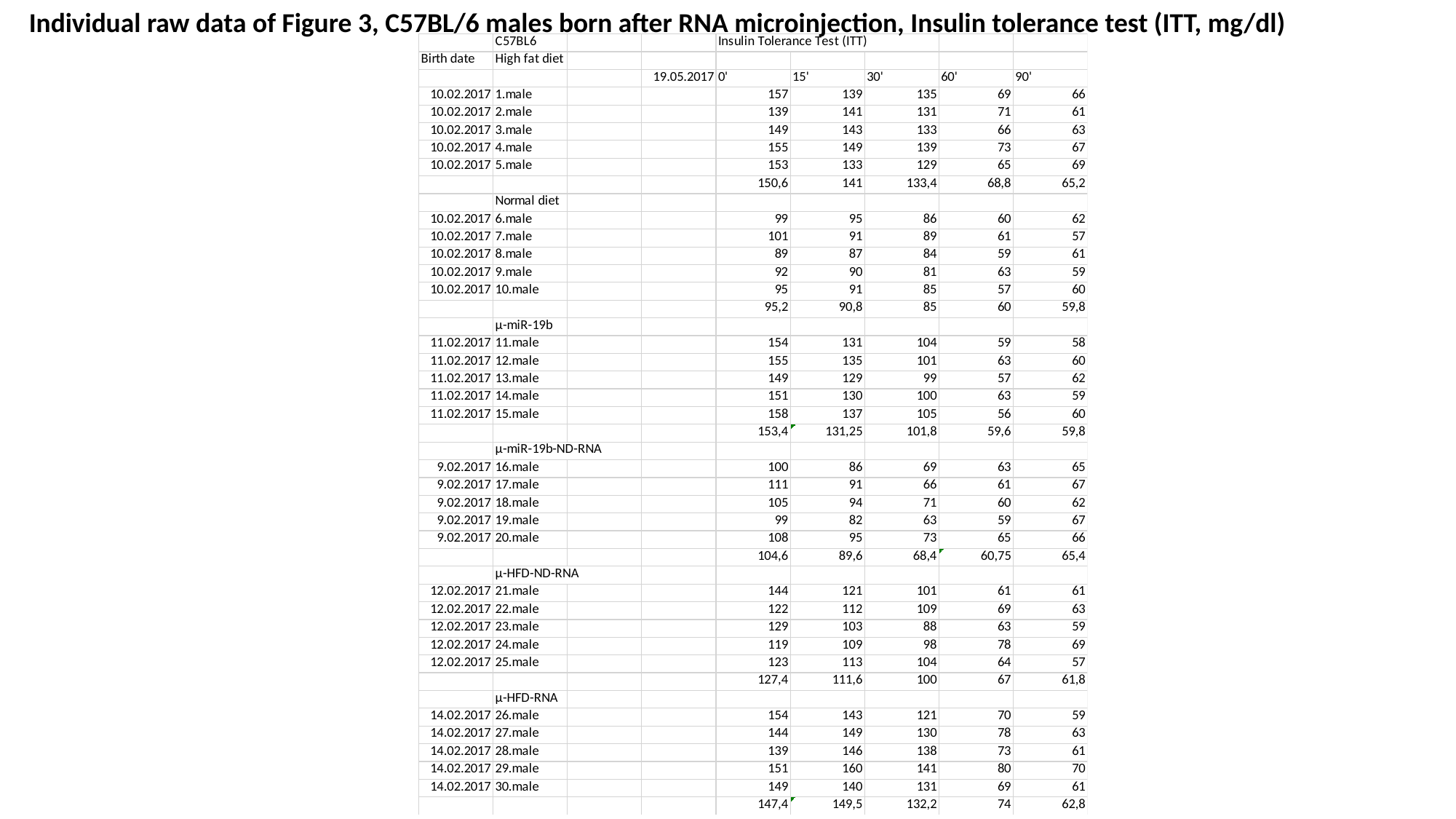

Individual raw data of Figure 3, C57BL/6 males born after RNA microinjection, Insulin tolerance test (ITT, mg/dl)

## Slide 51
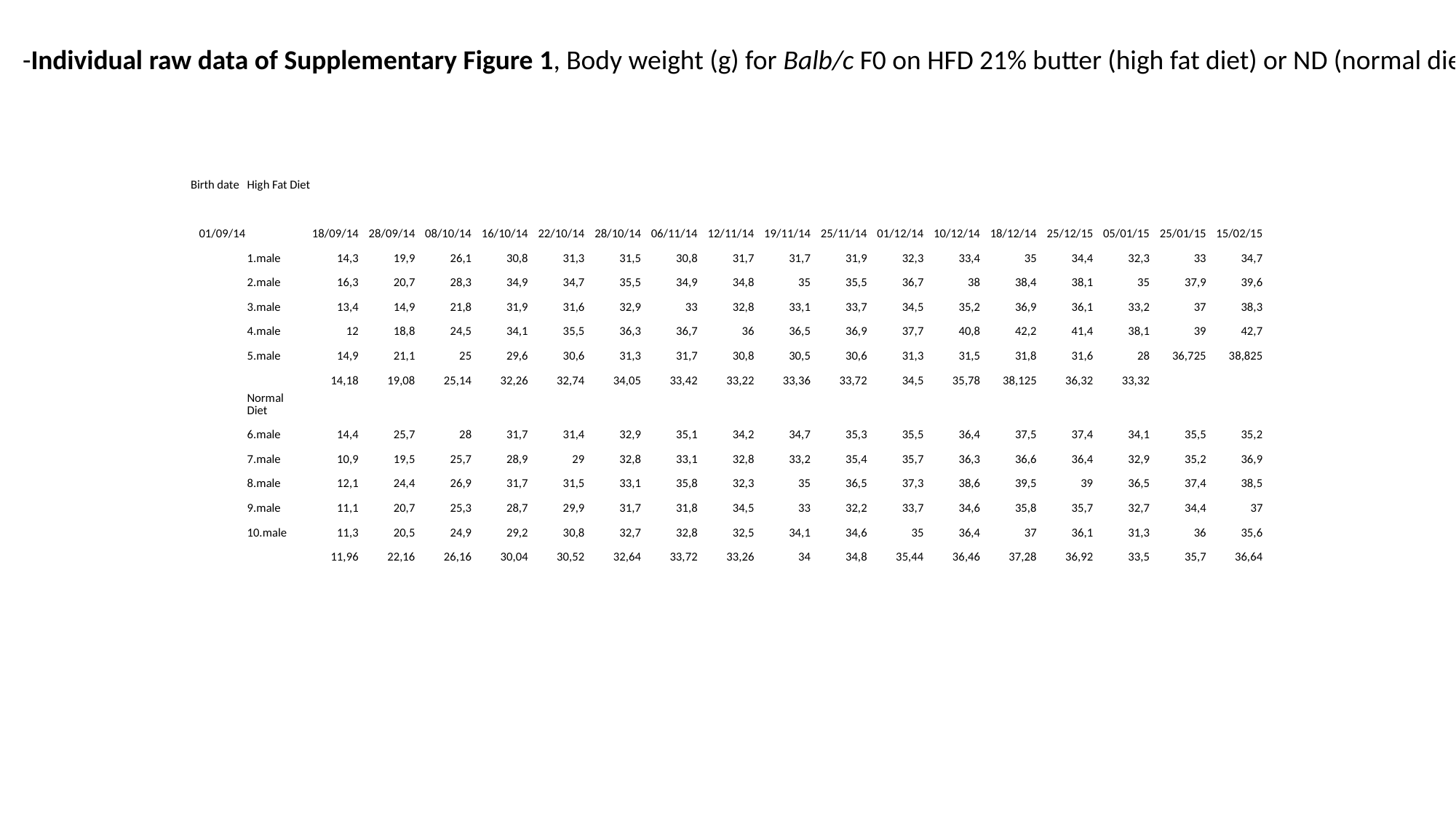

-Individual raw data of Supplementary Figure 1, Body weight (g) for Balb/c F0 on HFD 21% butter (high fat diet) or ND (normal diet).
| Birth date | High Fat Diet | | | | | | | | | | | | | | | | | |
| --- | --- | --- | --- | --- | --- | --- | --- | --- | --- | --- | --- | --- | --- | --- | --- | --- | --- | --- |
| | | | | | | | | | | | | | | | | | | |
| 01/09/14 | | 18/09/14 | 28/09/14 | 08/10/14 | 16/10/14 | 22/10/14 | 28/10/14 | 06/11/14 | 12/11/14 | 19/11/14 | 25/11/14 | 01/12/14 | 10/12/14 | 18/12/14 | 25/12/15 | 05/01/15 | 25/01/15 | 15/02/15 |
| | 1.male | 14,3 | 19,9 | 26,1 | 30,8 | 31,3 | 31,5 | 30,8 | 31,7 | 31,7 | 31,9 | 32,3 | 33,4 | 35 | 34,4 | 32,3 | 33 | 34,7 |
| | 2.male | 16,3 | 20,7 | 28,3 | 34,9 | 34,7 | 35,5 | 34,9 | 34,8 | 35 | 35,5 | 36,7 | 38 | 38,4 | 38,1 | 35 | 37,9 | 39,6 |
| | 3.male | 13,4 | 14,9 | 21,8 | 31,9 | 31,6 | 32,9 | 33 | 32,8 | 33,1 | 33,7 | 34,5 | 35,2 | 36,9 | 36,1 | 33,2 | 37 | 38,3 |
| | 4.male | 12 | 18,8 | 24,5 | 34,1 | 35,5 | 36,3 | 36,7 | 36 | 36,5 | 36,9 | 37,7 | 40,8 | 42,2 | 41,4 | 38,1 | 39 | 42,7 |
| | 5.male | 14,9 | 21,1 | 25 | 29,6 | 30,6 | 31,3 | 31,7 | 30,8 | 30,5 | 30,6 | 31,3 | 31,5 | 31,8 | 31,6 | 28 | 36,725 | 38,825 |
| | | 14,18 | 19,08 | 25,14 | 32,26 | 32,74 | 34,05 | 33,42 | 33,22 | 33,36 | 33,72 | 34,5 | 35,78 | 38,125 | 36,32 | 33,32 | | |
| | Normal Diet | | | | | | | | | | | | | | | | | |
| | 6.male | 14,4 | 25,7 | 28 | 31,7 | 31,4 | 32,9 | 35,1 | 34,2 | 34,7 | 35,3 | 35,5 | 36,4 | 37,5 | 37,4 | 34,1 | 35,5 | 35,2 |
| | 7.male | 10,9 | 19,5 | 25,7 | 28,9 | 29 | 32,8 | 33,1 | 32,8 | 33,2 | 35,4 | 35,7 | 36,3 | 36,6 | 36,4 | 32,9 | 35,2 | 36,9 |
| | 8.male | 12,1 | 24,4 | 26,9 | 31,7 | 31,5 | 33,1 | 35,8 | 32,3 | 35 | 36,5 | 37,3 | 38,6 | 39,5 | 39 | 36,5 | 37,4 | 38,5 |
| | 9.male | 11,1 | 20,7 | 25,3 | 28,7 | 29,9 | 31,7 | 31,8 | 34,5 | 33 | 32,2 | 33,7 | 34,6 | 35,8 | 35,7 | 32,7 | 34,4 | 37 |
| | 10.male | 11,3 | 20,5 | 24,9 | 29,2 | 30,8 | 32,7 | 32,8 | 32,5 | 34,1 | 34,6 | 35 | 36,4 | 37 | 36,1 | 31,3 | 36 | 35,6 |
| | | 11,96 | 22,16 | 26,16 | 30,04 | 30,52 | 32,64 | 33,72 | 33,26 | 34 | 34,8 | 35,44 | 36,46 | 37,28 | 36,92 | 33,5 | 35,7 | 36,64 |

## Slide 52
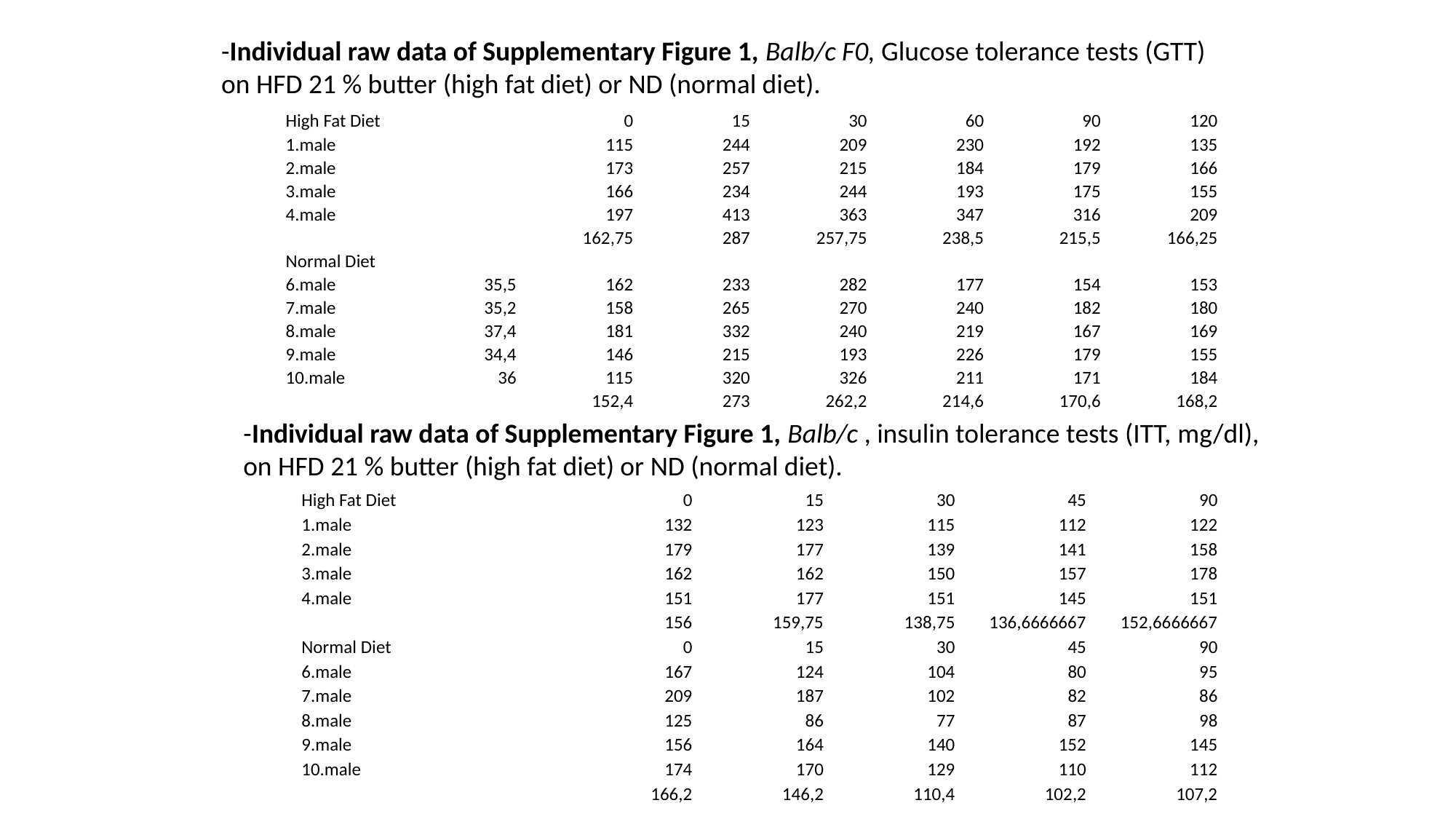

-Individual raw data of Supplementary Figure 1, Balb/c F0, Glucose tolerance tests (GTT)
on HFD 21 % butter (high fat diet) or ND (normal diet).
| High Fat Diet | | 0 | 15 | 30 | 60 | 90 | 120 |
| --- | --- | --- | --- | --- | --- | --- | --- |
| 1.male | | 115 | 244 | 209 | 230 | 192 | 135 |
| 2.male | | 173 | 257 | 215 | 184 | 179 | 166 |
| 3.male | | 166 | 234 | 244 | 193 | 175 | 155 |
| 4.male | | 197 | 413 | 363 | 347 | 316 | 209 |
| | | 162,75 | 287 | 257,75 | 238,5 | 215,5 | 166,25 |
| Normal Diet | | | | | | | |
| 6.male | 35,5 | 162 | 233 | 282 | 177 | 154 | 153 |
| 7.male | 35,2 | 158 | 265 | 270 | 240 | 182 | 180 |
| 8.male | 37,4 | 181 | 332 | 240 | 219 | 167 | 169 |
| 9.male | 34,4 | 146 | 215 | 193 | 226 | 179 | 155 |
| 10.male | 36 | 115 | 320 | 326 | 211 | 171 | 184 |
| | | 152,4 | 273 | 262,2 | 214,6 | 170,6 | 168,2 |
-Individual raw data of Supplementary Figure 1, Balb/c , insulin tolerance tests (ITT, mg/dl),
on HFD 21 % butter (high fat diet) or ND (normal diet).
| High Fat Diet | | 0 | 15 | 30 | 45 | 90 |
| --- | --- | --- | --- | --- | --- | --- |
| 1.male | | 132 | 123 | 115 | 112 | 122 |
| 2.male | | 179 | 177 | 139 | 141 | 158 |
| 3.male | | 162 | 162 | 150 | 157 | 178 |
| 4.male | | 151 | 177 | 151 | 145 | 151 |
| | | 156 | 159,75 | 138,75 | 136,6666667 | 152,6666667 |
| Normal Diet | | 0 | 15 | 30 | 45 | 90 |
| 6.male | | 167 | 124 | 104 | 80 | 95 |
| 7.male | | 209 | 187 | 102 | 82 | 86 |
| 8.male | | 125 | 86 | 77 | 87 | 98 |
| 9.male | | 156 | 164 | 140 | 152 | 145 |
| 10.male | | 174 | 170 | 129 | 110 | 112 |
| | | 166,2 | 146,2 | 110,4 | 102,2 | 107,2 |

## Slide 53
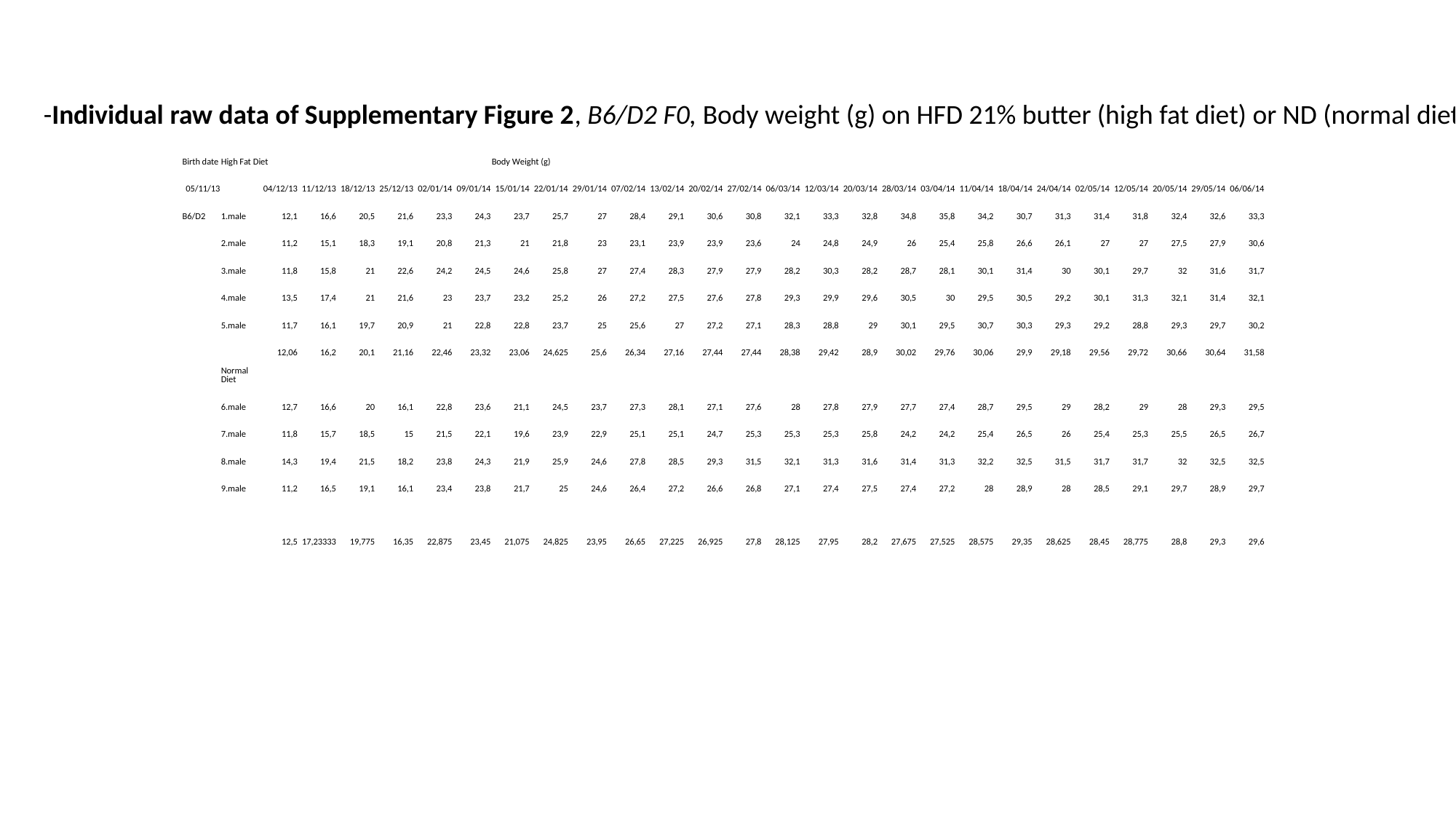

-Individual raw data of Supplementary Figure 2, B6/D2 F0, Body weight (g) on HFD 21% butter (high fat diet) or ND (normal diet).
| Birth date | High Fat Diet | | | | | | | Body Weight (g) | | | | | | | | | | | | | | | | | | | |
| --- | --- | --- | --- | --- | --- | --- | --- | --- | --- | --- | --- | --- | --- | --- | --- | --- | --- | --- | --- | --- | --- | --- | --- | --- | --- | --- | --- |
| 05/11/13 | | 04/12/13 | 11/12/13 | 18/12/13 | 25/12/13 | 02/01/14 | 09/01/14 | 15/01/14 | 22/01/14 | 29/01/14 | 07/02/14 | 13/02/14 | 20/02/14 | 27/02/14 | 06/03/14 | 12/03/14 | 20/03/14 | 28/03/14 | 03/04/14 | 11/04/14 | 18/04/14 | 24/04/14 | 02/05/14 | 12/05/14 | 20/05/14 | 29/05/14 | 06/06/14 |
| B6/D2 | 1.male | 12,1 | 16,6 | 20,5 | 21,6 | 23,3 | 24,3 | 23,7 | 25,7 | 27 | 28,4 | 29,1 | 30,6 | 30,8 | 32,1 | 33,3 | 32,8 | 34,8 | 35,8 | 34,2 | 30,7 | 31,3 | 31,4 | 31,8 | 32,4 | 32,6 | 33,3 |
| | 2.male | 11,2 | 15,1 | 18,3 | 19,1 | 20,8 | 21,3 | 21 | 21,8 | 23 | 23,1 | 23,9 | 23,9 | 23,6 | 24 | 24,8 | 24,9 | 26 | 25,4 | 25,8 | 26,6 | 26,1 | 27 | 27 | 27,5 | 27,9 | 30,6 |
| | 3.male | 11,8 | 15,8 | 21 | 22,6 | 24,2 | 24,5 | 24,6 | 25,8 | 27 | 27,4 | 28,3 | 27,9 | 27,9 | 28,2 | 30,3 | 28,2 | 28,7 | 28,1 | 30,1 | 31,4 | 30 | 30,1 | 29,7 | 32 | 31,6 | 31,7 |
| | 4.male | 13,5 | 17,4 | 21 | 21,6 | 23 | 23,7 | 23,2 | 25,2 | 26 | 27,2 | 27,5 | 27,6 | 27,8 | 29,3 | 29,9 | 29,6 | 30,5 | 30 | 29,5 | 30,5 | 29,2 | 30,1 | 31,3 | 32,1 | 31,4 | 32,1 |
| | 5.male | 11,7 | 16,1 | 19,7 | 20,9 | 21 | 22,8 | 22,8 | 23,7 | 25 | 25,6 | 27 | 27,2 | 27,1 | 28,3 | 28,8 | 29 | 30,1 | 29,5 | 30,7 | 30,3 | 29,3 | 29,2 | 28,8 | 29,3 | 29,7 | 30,2 |
| | | 12,06 | 16,2 | 20,1 | 21,16 | 22,46 | 23,32 | 23,06 | 24,625 | 25,6 | 26,34 | 27,16 | 27,44 | 27,44 | 28,38 | 29,42 | 28,9 | 30,02 | 29,76 | 30,06 | 29,9 | 29,18 | 29,56 | 29,72 | 30,66 | 30,64 | 31,58 |
| | Normal Diet | | | | | | | | | | | | | | | | | | | | | | | | | | |
| | 6.male | 12,7 | 16,6 | 20 | 16,1 | 22,8 | 23,6 | 21,1 | 24,5 | 23,7 | 27,3 | 28,1 | 27,1 | 27,6 | 28 | 27,8 | 27,9 | 27,7 | 27,4 | 28,7 | 29,5 | 29 | 28,2 | 29 | 28 | 29,3 | 29,5 |
| | 7.male | 11,8 | 15,7 | 18,5 | 15 | 21,5 | 22,1 | 19,6 | 23,9 | 22,9 | 25,1 | 25,1 | 24,7 | 25,3 | 25,3 | 25,3 | 25,8 | 24,2 | 24,2 | 25,4 | 26,5 | 26 | 25,4 | 25,3 | 25,5 | 26,5 | 26,7 |
| | 8.male | 14,3 | 19,4 | 21,5 | 18,2 | 23,8 | 24,3 | 21,9 | 25,9 | 24,6 | 27,8 | 28,5 | 29,3 | 31,5 | 32,1 | 31,3 | 31,6 | 31,4 | 31,3 | 32,2 | 32,5 | 31,5 | 31,7 | 31,7 | 32 | 32,5 | 32,5 |
| | 9.male | 11,2 | 16,5 | 19,1 | 16,1 | 23,4 | 23,8 | 21,7 | 25 | 24,6 | 26,4 | 27,2 | 26,6 | 26,8 | 27,1 | 27,4 | 27,5 | 27,4 | 27,2 | 28 | 28,9 | 28 | 28,5 | 29,1 | 29,7 | 28,9 | 29,7 |
| | | 12,5 | 17,23333 | 19,775 | 16,35 | 22,875 | 23,45 | 21,075 | 24,825 | 23,95 | 26,65 | 27,225 | 26,925 | 27,8 | 28,125 | 27,95 | 28,2 | 27,675 | 27,525 | 28,575 | 29,35 | 28,625 | 28,45 | 28,775 | 28,8 | 29,3 | 29,6 |

## Slide 54
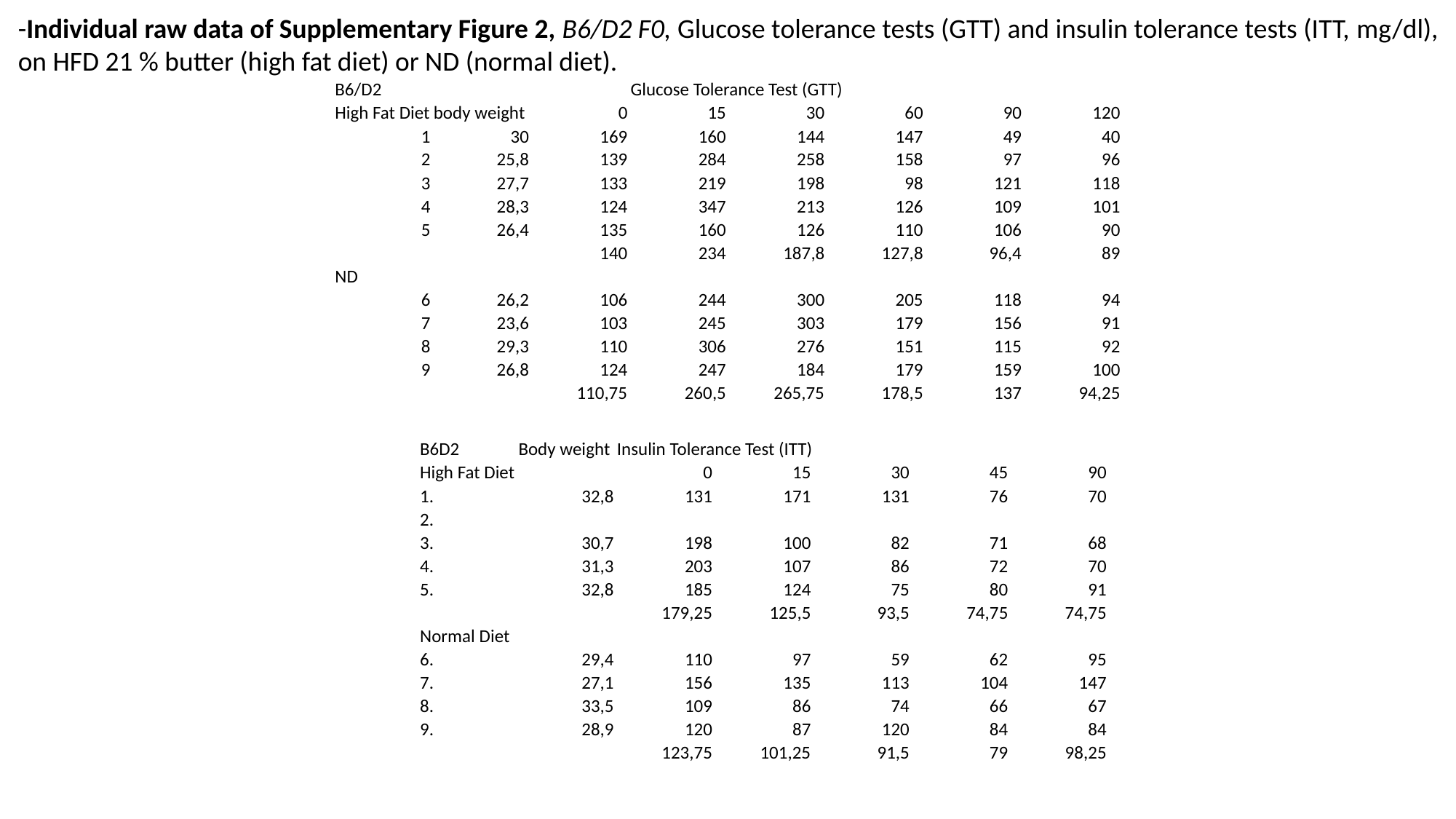

-Individual raw data of Supplementary Figure 2, B6/D2 F0, Glucose tolerance tests (GTT) and insulin tolerance tests (ITT, mg/dl),
on HFD 21 % butter (high fat diet) or ND (normal diet).
| B6/D2 | | | Glucose Tolerance Test (GTT) | | | | |
| --- | --- | --- | --- | --- | --- | --- | --- |
| High Fat Diet | body weight | 0 | 15 | 30 | 60 | 90 | 120 |
| 1 | 30 | 169 | 160 | 144 | 147 | 49 | 40 |
| 2 | 25,8 | 139 | 284 | 258 | 158 | 97 | 96 |
| 3 | 27,7 | 133 | 219 | 198 | 98 | 121 | 118 |
| 4 | 28,3 | 124 | 347 | 213 | 126 | 109 | 101 |
| 5 | 26,4 | 135 | 160 | 126 | 110 | 106 | 90 |
| | | 140 | 234 | 187,8 | 127,8 | 96,4 | 89 |
| ND | | | | | | | |
| 6 | 26,2 | 106 | 244 | 300 | 205 | 118 | 94 |
| 7 | 23,6 | 103 | 245 | 303 | 179 | 156 | 91 |
| 8 | 29,3 | 110 | 306 | 276 | 151 | 115 | 92 |
| 9 | 26,8 | 124 | 247 | 184 | 179 | 159 | 100 |
| | | 110,75 | 260,5 | 265,75 | 178,5 | 137 | 94,25 |
| B6D2 | Body weight | Insulin Tolerance Test (ITT) | | | | |
| --- | --- | --- | --- | --- | --- | --- |
| High Fat Diet | | 0 | 15 | 30 | 45 | 90 |
| 1. | 32,8 | 131 | 171 | 131 | 76 | 70 |
| 2. | | | | | | |
| 3. | 30,7 | 198 | 100 | 82 | 71 | 68 |
| 4. | 31,3 | 203 | 107 | 86 | 72 | 70 |
| 5. | 32,8 | 185 | 124 | 75 | 80 | 91 |
| | | 179,25 | 125,5 | 93,5 | 74,75 | 74,75 |
| Normal Diet | | | | | | |
| 6. | 29,4 | 110 | 97 | 59 | 62 | 95 |
| 7. | 27,1 | 156 | 135 | 113 | 104 | 147 |
| 8. | 33,5 | 109 | 86 | 74 | 66 | 67 |
| 9. | 28,9 | 120 | 87 | 120 | 84 | 84 |
| | | 123,75 | 101,25 | 91,5 | 79 | 98,25 |

## Slide 55
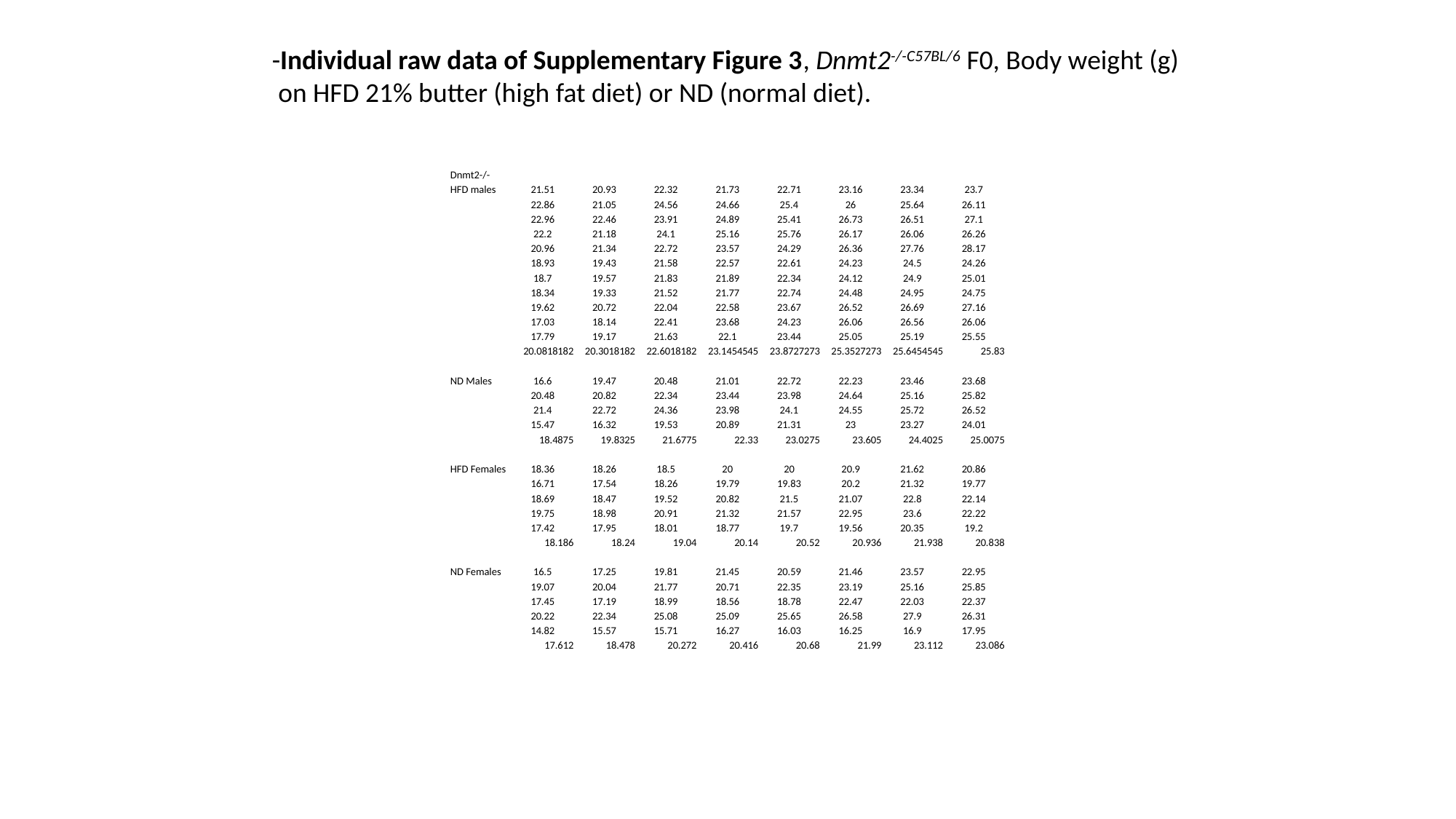

-Individual raw data of Supplementary Figure 3, Dnmt2-/-C57BL/6 F0, Body weight (g)
 on HFD 21% butter (high fat diet) or ND (normal diet).
| Dnmt2-/- | | | | | | | | |
| --- | --- | --- | --- | --- | --- | --- | --- | --- |
| HFD males | 21.51 | 20.93 | 22.32 | 21.73 | 22.71 | 23.16 | 23.34 | 23.7 |
| | 22.86 | 21.05 | 24.56 | 24.66 | 25.4 | 26 | 25.64 | 26.11 |
| | 22.96 | 22.46 | 23.91 | 24.89 | 25.41 | 26.73 | 26.51 | 27.1 |
| | 22.2 | 21.18 | 24.1 | 25.16 | 25.76 | 26.17 | 26.06 | 26.26 |
| | 20.96 | 21.34 | 22.72 | 23.57 | 24.29 | 26.36 | 27.76 | 28.17 |
| | 18.93 | 19.43 | 21.58 | 22.57 | 22.61 | 24.23 | 24.5 | 24.26 |
| | 18.7 | 19.57 | 21.83 | 21.89 | 22.34 | 24.12 | 24.9 | 25.01 |
| | 18.34 | 19.33 | 21.52 | 21.77 | 22.74 | 24.48 | 24.95 | 24.75 |
| | 19.62 | 20.72 | 22.04 | 22.58 | 23.67 | 26.52 | 26.69 | 27.16 |
| | 17.03 | 18.14 | 22.41 | 23.68 | 24.23 | 26.06 | 26.56 | 26.06 |
| | 17.79 | 19.17 | 21.63 | 22.1 | 23.44 | 25.05 | 25.19 | 25.55 |
| | 20.0818182 | 20.3018182 | 22.6018182 | 23.1454545 | 23.8727273 | 25.3527273 | 25.6454545 | 25.83 |
| | | | | | | | | |
| ND Males | 16.6 | 19.47 | 20.48 | 21.01 | 22.72 | 22.23 | 23.46 | 23.68 |
| | 20.48 | 20.82 | 22.34 | 23.44 | 23.98 | 24.64 | 25.16 | 25.82 |
| | 21.4 | 22.72 | 24.36 | 23.98 | 24.1 | 24.55 | 25.72 | 26.52 |
| | 15.47 | 16.32 | 19.53 | 20.89 | 21.31 | 23 | 23.27 | 24.01 |
| | 18.4875 | 19.8325 | 21.6775 | 22.33 | 23.0275 | 23.605 | 24.4025 | 25.0075 |
| | | | | | | | | |
| HFD Females | 18.36 | 18.26 | 18.5 | 20 | 20 | 20.9 | 21.62 | 20.86 |
| | 16.71 | 17.54 | 18.26 | 19.79 | 19.83 | 20.2 | 21.32 | 19.77 |
| | 18.69 | 18.47 | 19.52 | 20.82 | 21.5 | 21.07 | 22.8 | 22.14 |
| | 19.75 | 18.98 | 20.91 | 21.32 | 21.57 | 22.95 | 23.6 | 22.22 |
| | 17.42 | 17.95 | 18.01 | 18.77 | 19.7 | 19.56 | 20.35 | 19.2 |
| | 18.186 | 18.24 | 19.04 | 20.14 | 20.52 | 20.936 | 21.938 | 20.838 |
| | | | | | | | | |
| ND Females | 16.5 | 17.25 | 19.81 | 21.45 | 20.59 | 21.46 | 23.57 | 22.95 |
| | 19.07 | 20.04 | 21.77 | 20.71 | 22.35 | 23.19 | 25.16 | 25.85 |
| | 17.45 | 17.19 | 18.99 | 18.56 | 18.78 | 22.47 | 22.03 | 22.37 |
| | 20.22 | 22.34 | 25.08 | 25.09 | 25.65 | 26.58 | 27.9 | 26.31 |
| | 14.82 | 15.57 | 15.71 | 16.27 | 16.03 | 16.25 | 16.9 | 17.95 |
| | 17.612 | 18.478 | 20.272 | 20.416 | 20.68 | 21.99 | 23.112 | 23.086 |

## Slide 56
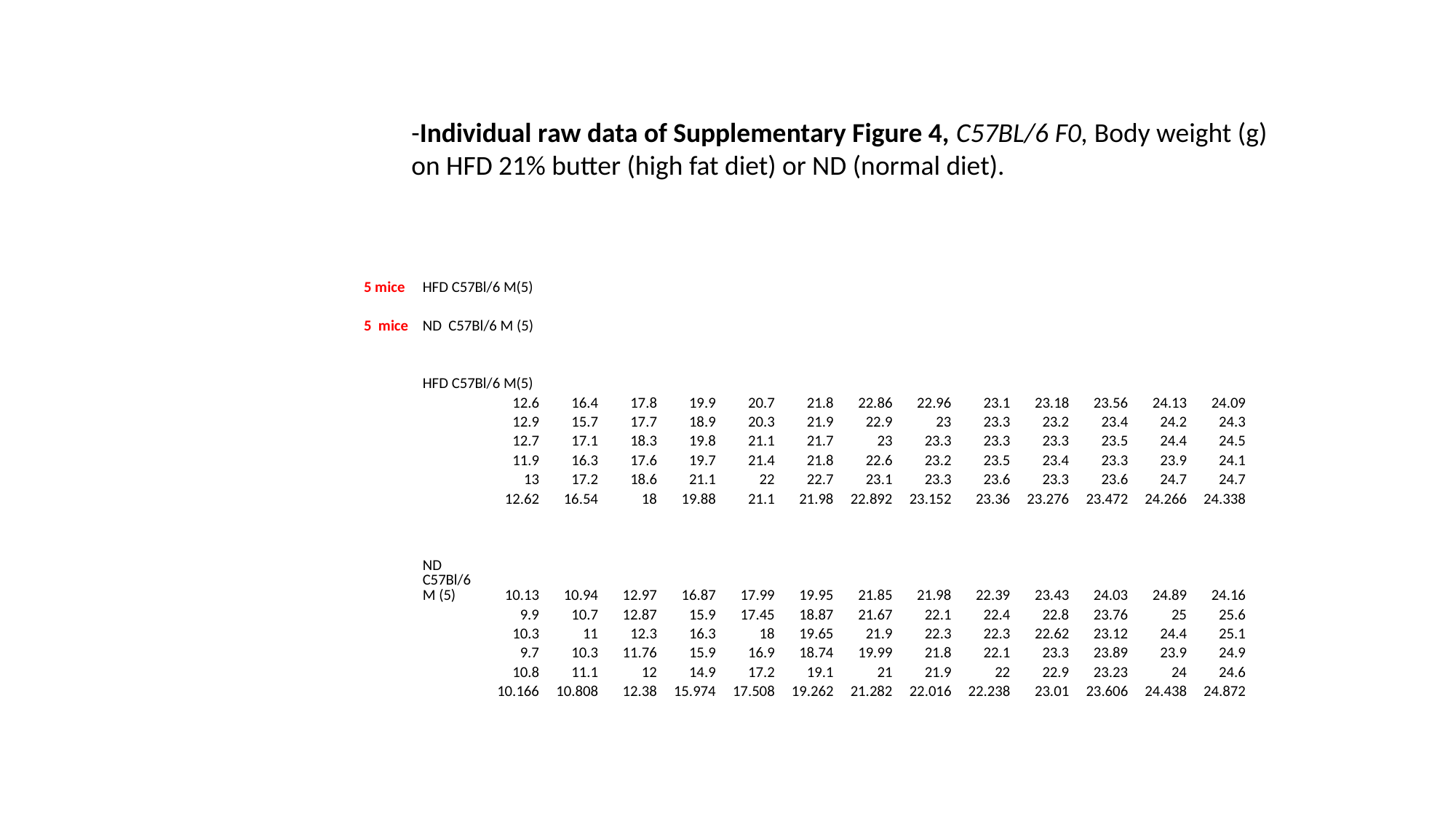

-Individual raw data of Supplementary Figure 4, C57BL/6 F0, Body weight (g)
on HFD 21% butter (high fat diet) or ND (normal diet).
| 5 mice | HFD C57Bl/6 M(5) | | | | | | | | | | | | | |
| --- | --- | --- | --- | --- | --- | --- | --- | --- | --- | --- | --- | --- | --- | --- |
| 5 mice | ND C57Bl/6 M (5) | | | | | | | | | | | | | |
| | | | | | | | | | | | | | | |
| | HFD C57Bl/6 M(5) | | | | | | | | | | | | | |
| | | 12.6 | 16.4 | 17.8 | 19.9 | 20.7 | 21.8 | 22.86 | 22.96 | 23.1 | 23.18 | 23.56 | 24.13 | 24.09 |
| | | 12.9 | 15.7 | 17.7 | 18.9 | 20.3 | 21.9 | 22.9 | 23 | 23.3 | 23.2 | 23.4 | 24.2 | 24.3 |
| | | 12.7 | 17.1 | 18.3 | 19.8 | 21.1 | 21.7 | 23 | 23.3 | 23.3 | 23.3 | 23.5 | 24.4 | 24.5 |
| | | 11.9 | 16.3 | 17.6 | 19.7 | 21.4 | 21.8 | 22.6 | 23.2 | 23.5 | 23.4 | 23.3 | 23.9 | 24.1 |
| | | 13 | 17.2 | 18.6 | 21.1 | 22 | 22.7 | 23.1 | 23.3 | 23.6 | 23.3 | 23.6 | 24.7 | 24.7 |
| | | 12.62 | 16.54 | 18 | 19.88 | 21.1 | 21.98 | 22.892 | 23.152 | 23.36 | 23.276 | 23.472 | 24.266 | 24.338 |
| | | | | | | | | | | | | | | |
| | | | | | | | | | | | | | | |
| | ND C57Bl/6 M (5) | 10.13 | 10.94 | 12.97 | 16.87 | 17.99 | 19.95 | 21.85 | 21.98 | 22.39 | 23.43 | 24.03 | 24.89 | 24.16 |
| | | 9.9 | 10.7 | 12.87 | 15.9 | 17.45 | 18.87 | 21.67 | 22.1 | 22.4 | 22.8 | 23.76 | 25 | 25.6 |
| | | 10.3 | 11 | 12.3 | 16.3 | 18 | 19.65 | 21.9 | 22.3 | 22.3 | 22.62 | 23.12 | 24.4 | 25.1 |
| | | 9.7 | 10.3 | 11.76 | 15.9 | 16.9 | 18.74 | 19.99 | 21.8 | 22.1 | 23.3 | 23.89 | 23.9 | 24.9 |
| | | 10.8 | 11.1 | 12 | 14.9 | 17.2 | 19.1 | 21 | 21.9 | 22 | 22.9 | 23.23 | 24 | 24.6 |
| | | 10.166 | 10.808 | 12.38 | 15.974 | 17.508 | 19.262 | 21.282 | 22.016 | 22.238 | 23.01 | 23.606 | 24.438 | 24.872 |

## Slide 57
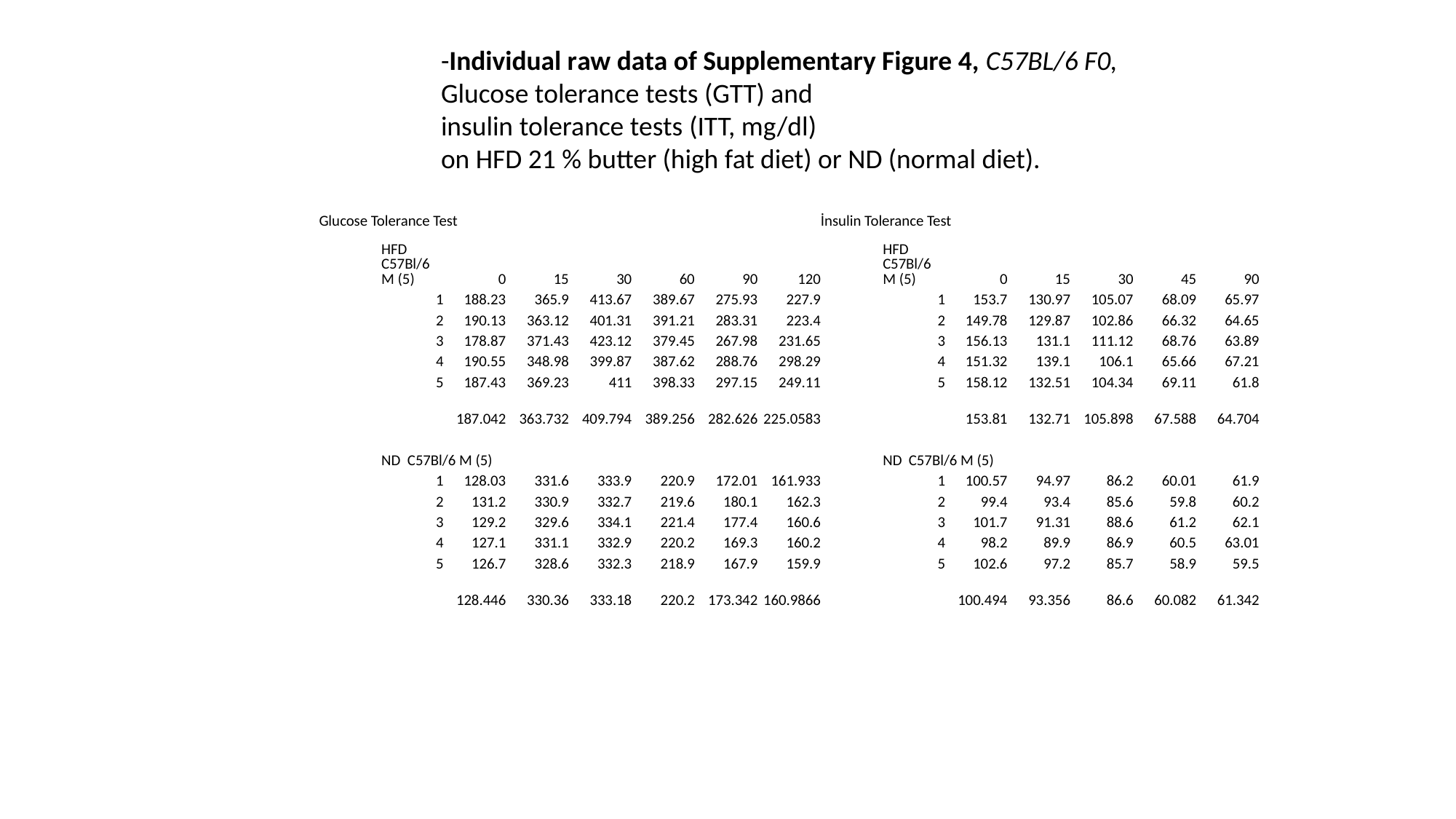

-Individual raw data of Supplementary Figure 4, C57BL/6 F0,
Glucose tolerance tests (GTT) and
insulin tolerance tests (ITT, mg/dl)
on HFD 21 % butter (high fat diet) or ND (normal diet).
| Glucose Tolerance Test | | | | | | | | İnsulin Tolerance Test | | | | | | |
| --- | --- | --- | --- | --- | --- | --- | --- | --- | --- | --- | --- | --- | --- | --- |
| | HFD C57Bl/6 M (5) | 0 | 15 | 30 | 60 | 90 | 120 | | HFD C57Bl/6 M (5) | 0 | 15 | 30 | 45 | 90 |
| | 1 | 188.23 | 365.9 | 413.67 | 389.67 | 275.93 | 227.9 | | 1 | 153.7 | 130.97 | 105.07 | 68.09 | 65.97 |
| | 2 | 190.13 | 363.12 | 401.31 | 391.21 | 283.31 | 223.4 | | 2 | 149.78 | 129.87 | 102.86 | 66.32 | 64.65 |
| | 3 | 178.87 | 371.43 | 423.12 | 379.45 | 267.98 | 231.65 | | 3 | 156.13 | 131.1 | 111.12 | 68.76 | 63.89 |
| | 4 | 190.55 | 348.98 | 399.87 | 387.62 | 288.76 | 298.29 | | 4 | 151.32 | 139.1 | 106.1 | 65.66 | 67.21 |
| | 5 | 187.43 | 369.23 | 411 | 398.33 | 297.15 | 249.11 | | 5 | 158.12 | 132.51 | 104.34 | 69.11 | 61.8 |
| | | 187.042 | 363.732 | 409.794 | 389.256 | 282.626 | 225.0583 | | | 153.81 | 132.71 | 105.898 | 67.588 | 64.704 |
| | | | | | | | | | | | | | | |
| | ND C57Bl/6 M (5) | | | | | | | | ND C57Bl/6 M (5) | | | | | |
| | 1 | 128.03 | 331.6 | 333.9 | 220.9 | 172.01 | 161.933 | | 1 | 100.57 | 94.97 | 86.2 | 60.01 | 61.9 |
| | 2 | 131.2 | 330.9 | 332.7 | 219.6 | 180.1 | 162.3 | | 2 | 99.4 | 93.4 | 85.6 | 59.8 | 60.2 |
| | 3 | 129.2 | 329.6 | 334.1 | 221.4 | 177.4 | 160.6 | | 3 | 101.7 | 91.31 | 88.6 | 61.2 | 62.1 |
| | 4 | 127.1 | 331.1 | 332.9 | 220.2 | 169.3 | 160.2 | | 4 | 98.2 | 89.9 | 86.9 | 60.5 | 63.01 |
| | 5 | 126.7 | 328.6 | 332.3 | 218.9 | 167.9 | 159.9 | | 5 | 102.6 | 97.2 | 85.7 | 58.9 | 59.5 |
| | | 128.446 | 330.36 | 333.18 | 220.2 | 173.342 | 160.9866 | | | 100.494 | 93.356 | 86.6 | 60.082 | 61.342 |

## Slide 58
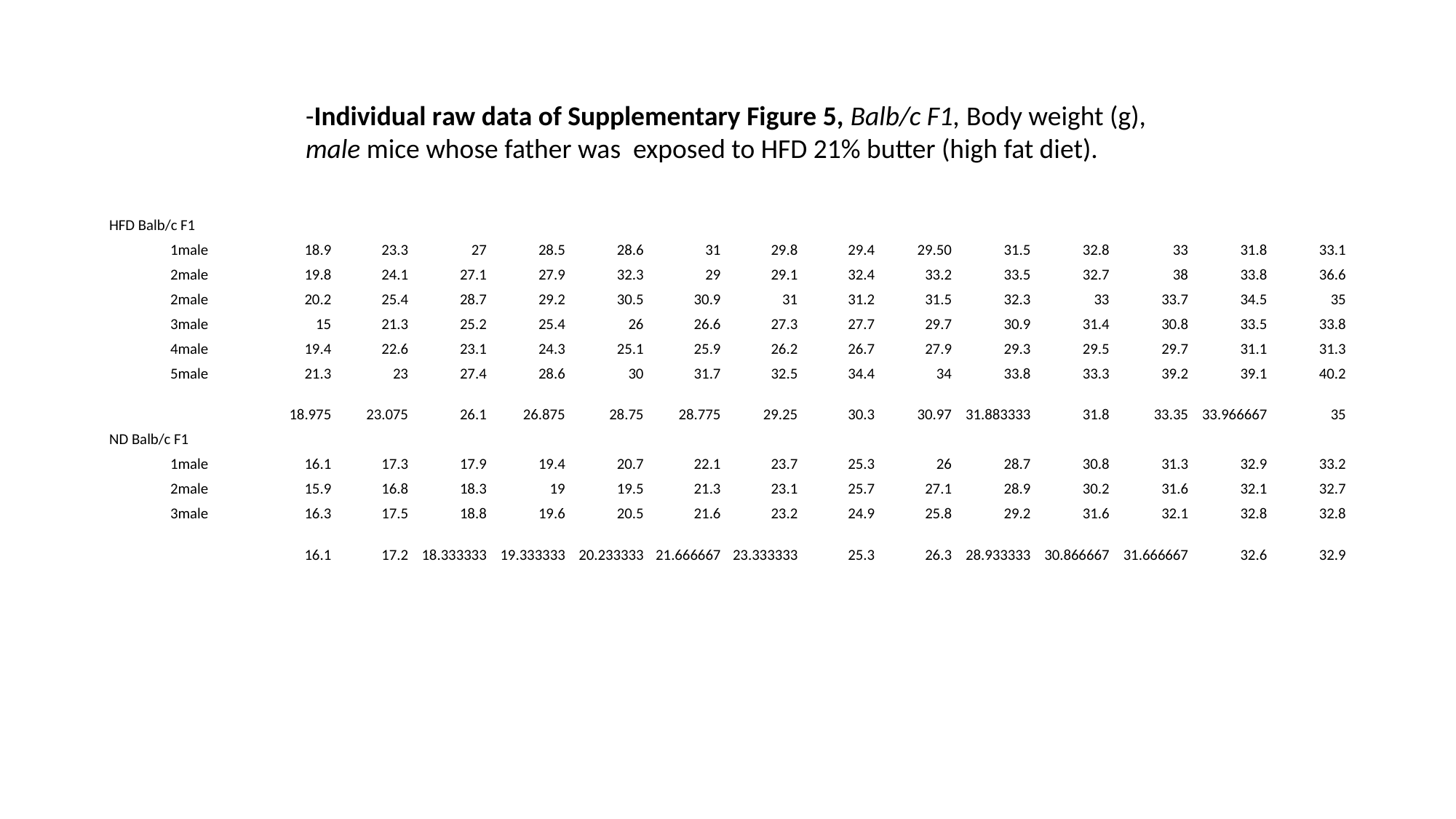

-Individual raw data of Supplementary Figure 5, Balb/c F1, Body weight (g),
male mice whose father was exposed to HFD 21% butter (high fat diet).
| HFD Balb/c F1 | | | | | | | | | | | | | | | |
| --- | --- | --- | --- | --- | --- | --- | --- | --- | --- | --- | --- | --- | --- | --- | --- |
| 1 | male | 18.9 | 23.3 | 27 | 28.5 | 28.6 | 31 | 29.8 | 29.4 | 29.50 | 31.5 | 32.8 | 33 | 31.8 | 33.1 |
| 2 | male | 19.8 | 24.1 | 27.1 | 27.9 | 32.3 | 29 | 29.1 | 32.4 | 33.2 | 33.5 | 32.7 | 38 | 33.8 | 36.6 |
| 2 | male | 20.2 | 25.4 | 28.7 | 29.2 | 30.5 | 30.9 | 31 | 31.2 | 31.5 | 32.3 | 33 | 33.7 | 34.5 | 35 |
| 3 | male | 15 | 21.3 | 25.2 | 25.4 | 26 | 26.6 | 27.3 | 27.7 | 29.7 | 30.9 | 31.4 | 30.8 | 33.5 | 33.8 |
| 4 | male | 19.4 | 22.6 | 23.1 | 24.3 | 25.1 | 25.9 | 26.2 | 26.7 | 27.9 | 29.3 | 29.5 | 29.7 | 31.1 | 31.3 |
| 5 | male | 21.3 | 23 | 27.4 | 28.6 | 30 | 31.7 | 32.5 | 34.4 | 34 | 33.8 | 33.3 | 39.2 | 39.1 | 40.2 |
| | | 18.975 | 23.075 | 26.1 | 26.875 | 28.75 | 28.775 | 29.25 | 30.3 | 30.97 | 31.883333 | 31.8 | 33.35 | 33.966667 | 35 |
| ND Balb/c F1 | | | | | | | | | | | | | | | |
| 1 | male | 16.1 | 17.3 | 17.9 | 19.4 | 20.7 | 22.1 | 23.7 | 25.3 | 26 | 28.7 | 30.8 | 31.3 | 32.9 | 33.2 |
| 2 | male | 15.9 | 16.8 | 18.3 | 19 | 19.5 | 21.3 | 23.1 | 25.7 | 27.1 | 28.9 | 30.2 | 31.6 | 32.1 | 32.7 |
| 3 | male | 16.3 | 17.5 | 18.8 | 19.6 | 20.5 | 21.6 | 23.2 | 24.9 | 25.8 | 29.2 | 31.6 | 32.1 | 32.8 | 32.8 |
| | | 16.1 | 17.2 | 18.333333 | 19.333333 | 20.233333 | 21.666667 | 23.333333 | 25.3 | 26.3 | 28.933333 | 30.866667 | 31.666667 | 32.6 | 32.9 |

## Slide 59
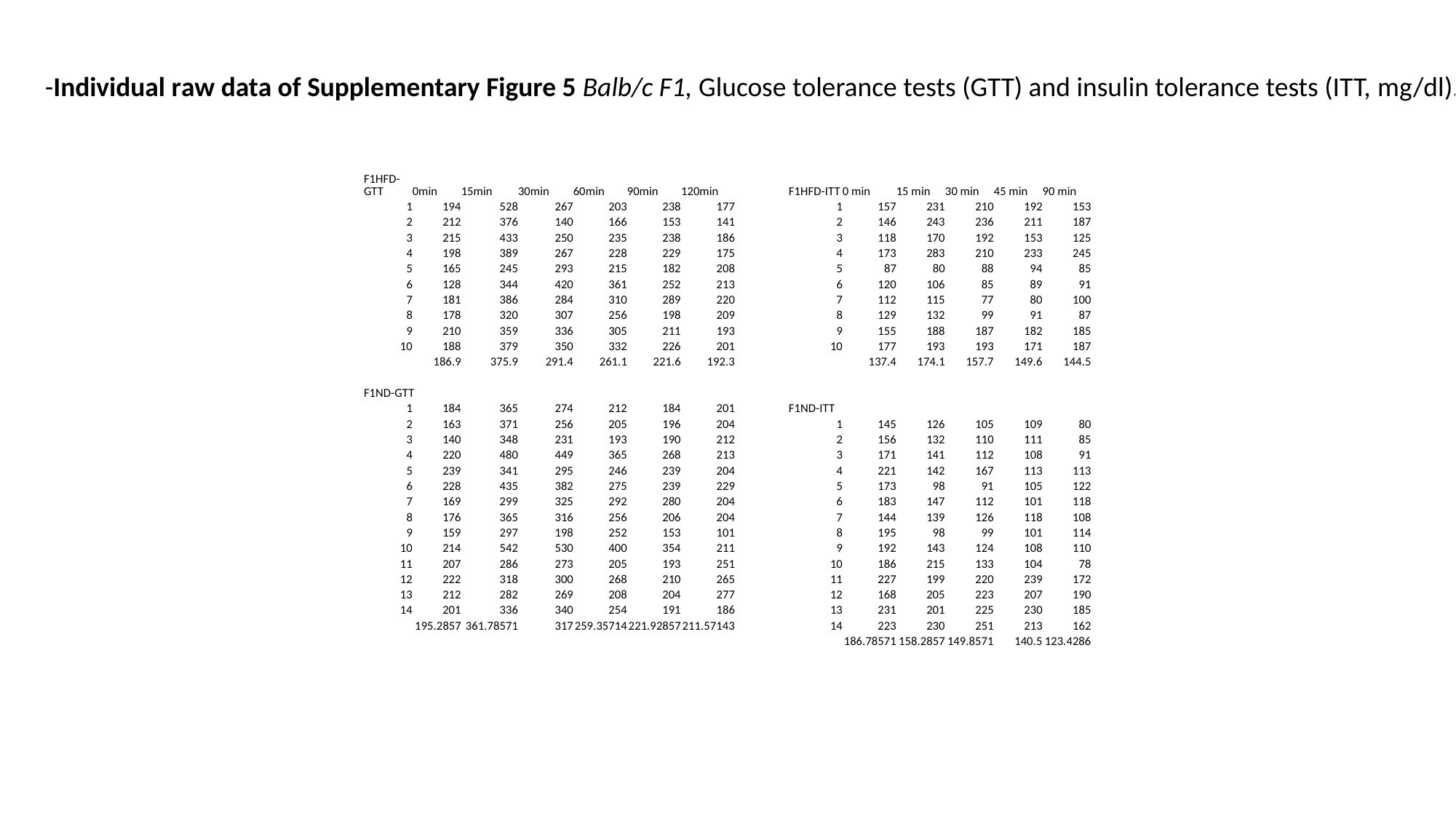

-Individual raw data of Supplementary Figure 5 Balb/c F1, Glucose tolerance tests (GTT) and insulin tolerance tests (ITT, mg/dl).
| F1HFD-GTT | 0min | 15min | 30min | 60min | 90min | 120min | | F1HFD-ITT | 0 min | 15 min | 30 min | 45 min | 90 min |
| --- | --- | --- | --- | --- | --- | --- | --- | --- | --- | --- | --- | --- | --- |
| 1 | 194 | 528 | 267 | 203 | 238 | 177 | | 1 | 157 | 231 | 210 | 192 | 153 |
| 2 | 212 | 376 | 140 | 166 | 153 | 141 | | 2 | 146 | 243 | 236 | 211 | 187 |
| 3 | 215 | 433 | 250 | 235 | 238 | 186 | | 3 | 118 | 170 | 192 | 153 | 125 |
| 4 | 198 | 389 | 267 | 228 | 229 | 175 | | 4 | 173 | 283 | 210 | 233 | 245 |
| 5 | 165 | 245 | 293 | 215 | 182 | 208 | | 5 | 87 | 80 | 88 | 94 | 85 |
| 6 | 128 | 344 | 420 | 361 | 252 | 213 | | 6 | 120 | 106 | 85 | 89 | 91 |
| 7 | 181 | 386 | 284 | 310 | 289 | 220 | | 7 | 112 | 115 | 77 | 80 | 100 |
| 8 | 178 | 320 | 307 | 256 | 198 | 209 | | 8 | 129 | 132 | 99 | 91 | 87 |
| 9 | 210 | 359 | 336 | 305 | 211 | 193 | | 9 | 155 | 188 | 187 | 182 | 185 |
| 10 | 188 | 379 | 350 | 332 | 226 | 201 | | 10 | 177 | 193 | 193 | 171 | 187 |
| | 186.9 | 375.9 | 291.4 | 261.1 | 221.6 | 192.3 | | | 137.4 | 174.1 | 157.7 | 149.6 | 144.5 |
| | | | | | | | | | | | | | |
| F1ND-GTT | | | | | | | | | | | | | |
| 1 | 184 | 365 | 274 | 212 | 184 | 201 | | F1ND-ITT | | | | | |
| 2 | 163 | 371 | 256 | 205 | 196 | 204 | | 1 | 145 | 126 | 105 | 109 | 80 |
| 3 | 140 | 348 | 231 | 193 | 190 | 212 | | 2 | 156 | 132 | 110 | 111 | 85 |
| 4 | 220 | 480 | 449 | 365 | 268 | 213 | | 3 | 171 | 141 | 112 | 108 | 91 |
| 5 | 239 | 341 | 295 | 246 | 239 | 204 | | 4 | 221 | 142 | 167 | 113 | 113 |
| 6 | 228 | 435 | 382 | 275 | 239 | 229 | | 5 | 173 | 98 | 91 | 105 | 122 |
| 7 | 169 | 299 | 325 | 292 | 280 | 204 | | 6 | 183 | 147 | 112 | 101 | 118 |
| 8 | 176 | 365 | 316 | 256 | 206 | 204 | | 7 | 144 | 139 | 126 | 118 | 108 |
| 9 | 159 | 297 | 198 | 252 | 153 | 101 | | 8 | 195 | 98 | 99 | 101 | 114 |
| 10 | 214 | 542 | 530 | 400 | 354 | 211 | | 9 | 192 | 143 | 124 | 108 | 110 |
| 11 | 207 | 286 | 273 | 205 | 193 | 251 | | 10 | 186 | 215 | 133 | 104 | 78 |
| 12 | 222 | 318 | 300 | 268 | 210 | 265 | | 11 | 227 | 199 | 220 | 239 | 172 |
| 13 | 212 | 282 | 269 | 208 | 204 | 277 | | 12 | 168 | 205 | 223 | 207 | 190 |
| 14 | 201 | 336 | 340 | 254 | 191 | 186 | | 13 | 231 | 201 | 225 | 230 | 185 |
| | 195.2857 | 361.78571 | 317 | 259.35714 | 221.92857 | 211.57143 | | 14 | 223 | 230 | 251 | 213 | 162 |
| | | | | | | | | | 186.78571 | 158.2857 | 149.8571 | 140.5 | 123.4286 |

## Slide 60
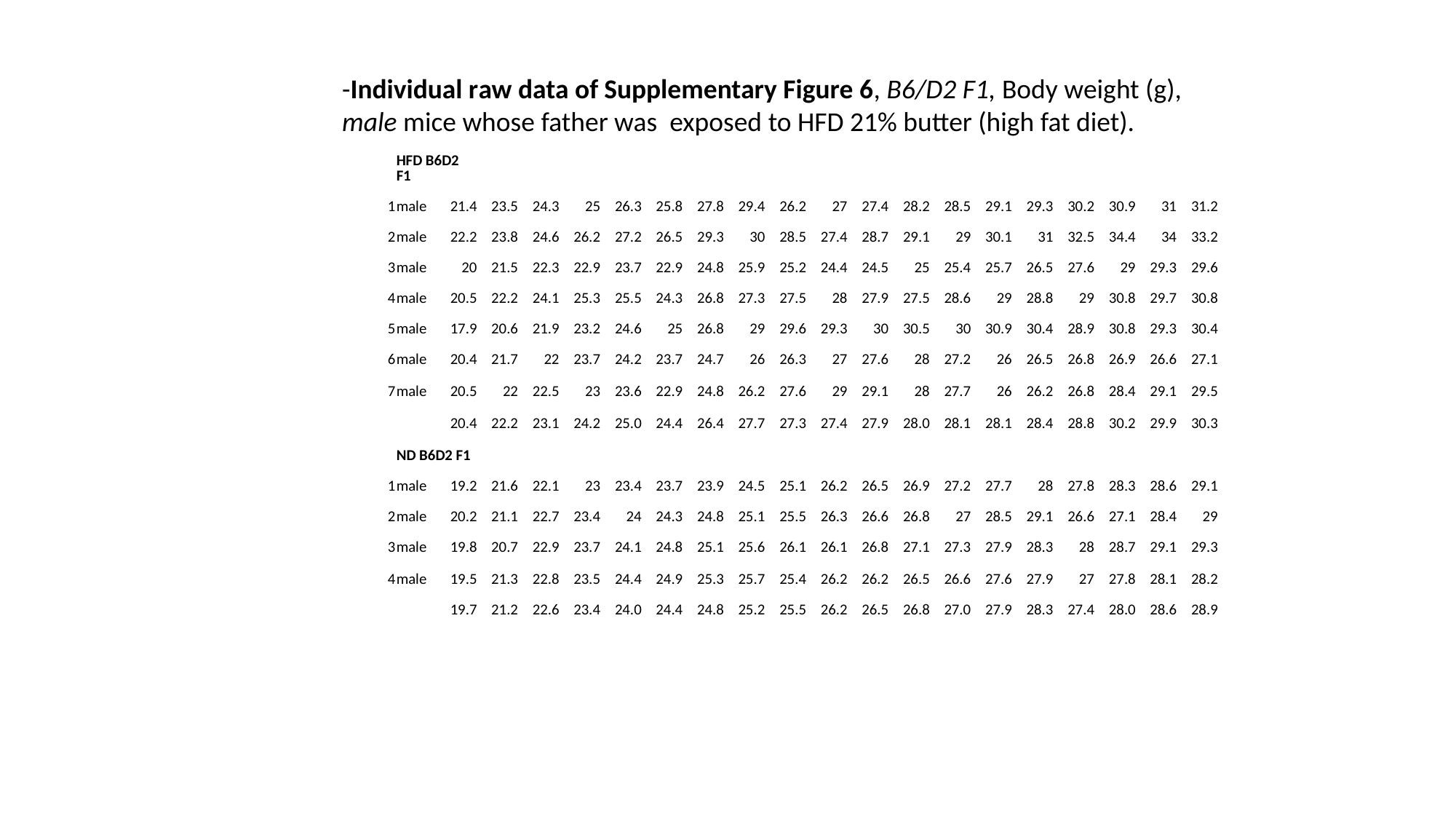

-Individual raw data of Supplementary Figure 6, B6/D2 F1, Body weight (g),
male mice whose father was exposed to HFD 21% butter (high fat diet).
| | HFD B6D2 F1 | | | | | | | | | | | | | | | | | | | |
| --- | --- | --- | --- | --- | --- | --- | --- | --- | --- | --- | --- | --- | --- | --- | --- | --- | --- | --- | --- | --- |
| 1 | male | 21.4 | 23.5 | 24.3 | 25 | 26.3 | 25.8 | 27.8 | 29.4 | 26.2 | 27 | 27.4 | 28.2 | 28.5 | 29.1 | 29.3 | 30.2 | 30.9 | 31 | 31.2 |
| 2 | male | 22.2 | 23.8 | 24.6 | 26.2 | 27.2 | 26.5 | 29.3 | 30 | 28.5 | 27.4 | 28.7 | 29.1 | 29 | 30.1 | 31 | 32.5 | 34.4 | 34 | 33.2 |
| 3 | male | 20 | 21.5 | 22.3 | 22.9 | 23.7 | 22.9 | 24.8 | 25.9 | 25.2 | 24.4 | 24.5 | 25 | 25.4 | 25.7 | 26.5 | 27.6 | 29 | 29.3 | 29.6 |
| 4 | male | 20.5 | 22.2 | 24.1 | 25.3 | 25.5 | 24.3 | 26.8 | 27.3 | 27.5 | 28 | 27.9 | 27.5 | 28.6 | 29 | 28.8 | 29 | 30.8 | 29.7 | 30.8 |
| 5 | male | 17.9 | 20.6 | 21.9 | 23.2 | 24.6 | 25 | 26.8 | 29 | 29.6 | 29.3 | 30 | 30.5 | 30 | 30.9 | 30.4 | 28.9 | 30.8 | 29.3 | 30.4 |
| 6 | male | 20.4 | 21.7 | 22 | 23.7 | 24.2 | 23.7 | 24.7 | 26 | 26.3 | 27 | 27.6 | 28 | 27.2 | 26 | 26.5 | 26.8 | 26.9 | 26.6 | 27.1 |
| 7 | male | 20.5 | 22 | 22.5 | 23 | 23.6 | 22.9 | 24.8 | 26.2 | 27.6 | 29 | 29.1 | 28 | 27.7 | 26 | 26.2 | 26.8 | 28.4 | 29.1 | 29.5 |
| | | 20.4 | 22.2 | 23.1 | 24.2 | 25.0 | 24.4 | 26.4 | 27.7 | 27.3 | 27.4 | 27.9 | 28.0 | 28.1 | 28.1 | 28.4 | 28.8 | 30.2 | 29.9 | 30.3 |
| | ND B6D2 F1 | | | | | | | | | | | | | | | | | | | |
| 1 | male | 19.2 | 21.6 | 22.1 | 23 | 23.4 | 23.7 | 23.9 | 24.5 | 25.1 | 26.2 | 26.5 | 26.9 | 27.2 | 27.7 | 28 | 27.8 | 28.3 | 28.6 | 29.1 |
| 2 | male | 20.2 | 21.1 | 22.7 | 23.4 | 24 | 24.3 | 24.8 | 25.1 | 25.5 | 26.3 | 26.6 | 26.8 | 27 | 28.5 | 29.1 | 26.6 | 27.1 | 28.4 | 29 |
| 3 | male | 19.8 | 20.7 | 22.9 | 23.7 | 24.1 | 24.8 | 25.1 | 25.6 | 26.1 | 26.1 | 26.8 | 27.1 | 27.3 | 27.9 | 28.3 | 28 | 28.7 | 29.1 | 29.3 |
| 4 | male | 19.5 | 21.3 | 22.8 | 23.5 | 24.4 | 24.9 | 25.3 | 25.7 | 25.4 | 26.2 | 26.2 | 26.5 | 26.6 | 27.6 | 27.9 | 27 | 27.8 | 28.1 | 28.2 |
| | | 19.7 | 21.2 | 22.6 | 23.4 | 24.0 | 24.4 | 24.8 | 25.2 | 25.5 | 26.2 | 26.5 | 26.8 | 27.0 | 27.9 | 28.3 | 27.4 | 28.0 | 28.6 | 28.9 |

## Slide 61
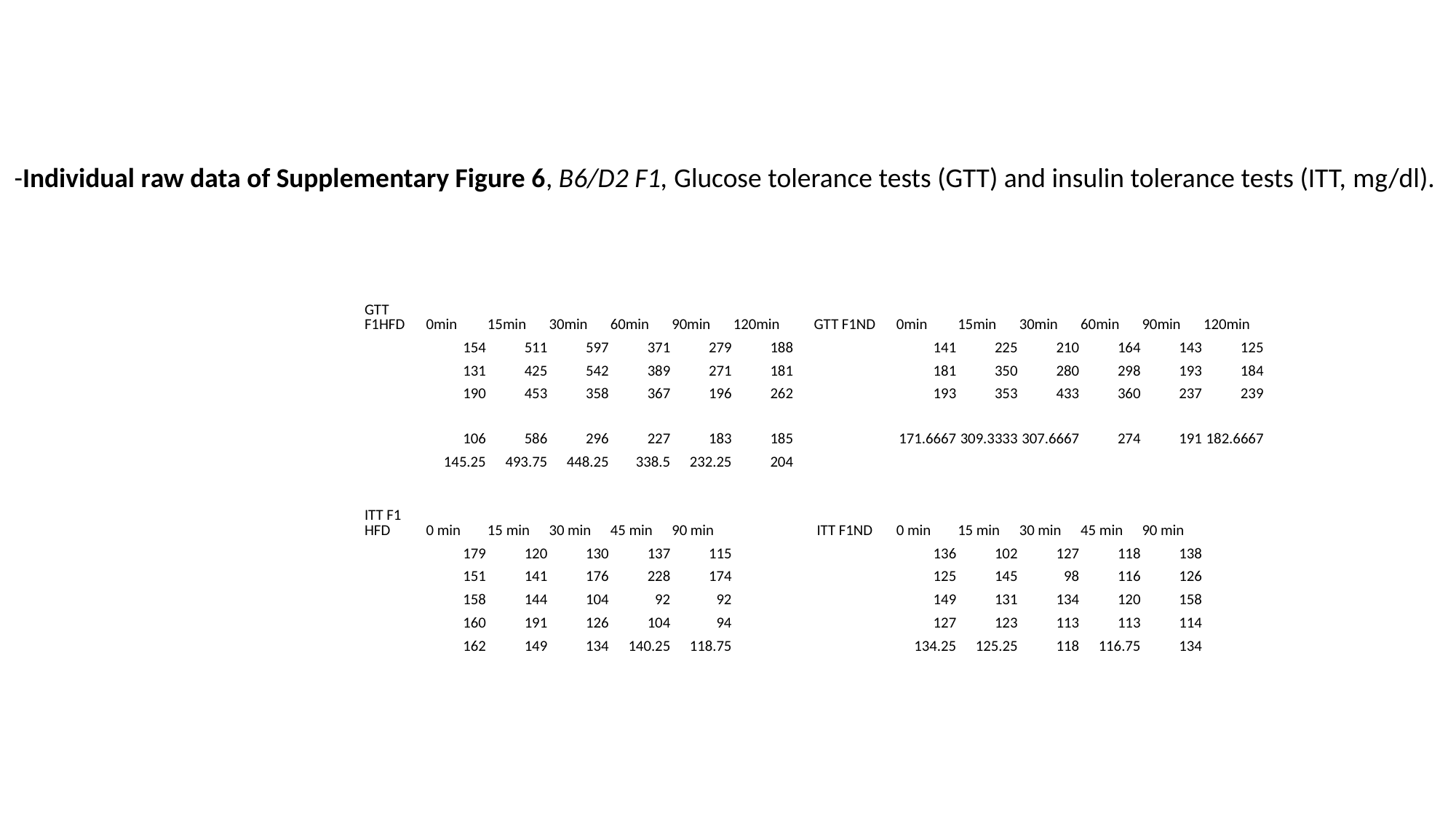

-Individual raw data of Supplementary Figure 6, B6/D2 F1, Glucose tolerance tests (GTT) and insulin tolerance tests (ITT, mg/dl).
| GTT F1HFD | 0min | 15min | 30min | 60min | 90min | 120min | GTT F1ND | 0min | 15min | 30min | 60min | 90min | 120min |
| --- | --- | --- | --- | --- | --- | --- | --- | --- | --- | --- | --- | --- | --- |
| | 154 | 511 | 597 | 371 | 279 | 188 | | 141 | 225 | 210 | 164 | 143 | 125 |
| | 131 | 425 | 542 | 389 | 271 | 181 | | 181 | 350 | 280 | 298 | 193 | 184 |
| | 190 | 453 | 358 | 367 | 196 | 262 | | 193 | 353 | 433 | 360 | 237 | 239 |
| | 106 | 586 | 296 | 227 | 183 | 185 | | 171.6667 | 309.3333 | 307.6667 | 274 | 191 | 182.6667 |
| | 145.25 | 493.75 | 448.25 | 338.5 | 232.25 | 204 | | | | | | | |
| | | | | | | | | | | | | | |
| ITT F1 HFD | 0 min | 15 min | 30 min | 45 min | 90 min | | ITT F1ND | 0 min | 15 min | 30 min | 45 min | 90 min | |
| | 179 | 120 | 130 | 137 | 115 | | | 136 | 102 | 127 | 118 | 138 | |
| | 151 | 141 | 176 | 228 | 174 | | | 125 | 145 | 98 | 116 | 126 | |
| | 158 | 144 | 104 | 92 | 92 | | | 149 | 131 | 134 | 120 | 158 | |
| | 160 | 191 | 126 | 104 | 94 | | | 127 | 123 | 113 | 113 | 114 | |
| | 162 | 149 | 134 | 140.25 | 118.75 | | | 134.25 | 125.25 | 118 | 116.75 | 134 | |

## Slide 62
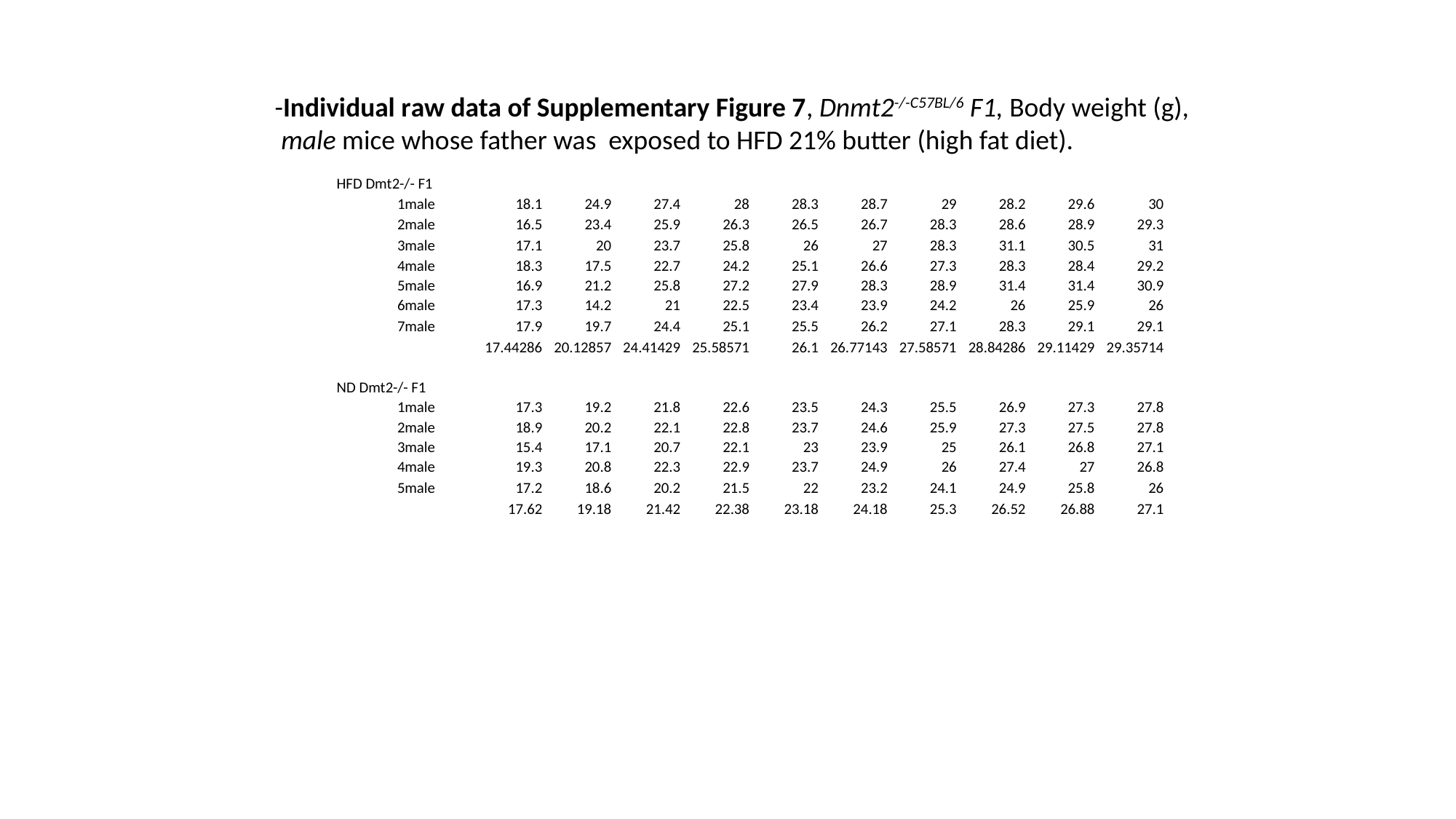

-Individual raw data of Supplementary Figure 7, Dnmt2-/-C57BL/6 F1, Body weight (g),
 male mice whose father was exposed to HFD 21% butter (high fat diet).
| HFD Dmt2-/- F1 | | | | | | | | | | | |
| --- | --- | --- | --- | --- | --- | --- | --- | --- | --- | --- | --- |
| 1 | male | 18.1 | 24.9 | 27.4 | 28 | 28.3 | 28.7 | 29 | 28.2 | 29.6 | 30 |
| 2 | male | 16.5 | 23.4 | 25.9 | 26.3 | 26.5 | 26.7 | 28.3 | 28.6 | 28.9 | 29.3 |
| 3 | male | 17.1 | 20 | 23.7 | 25.8 | 26 | 27 | 28.3 | 31.1 | 30.5 | 31 |
| 4 | male | 18.3 | 17.5 | 22.7 | 24.2 | 25.1 | 26.6 | 27.3 | 28.3 | 28.4 | 29.2 |
| 5 | male | 16.9 | 21.2 | 25.8 | 27.2 | 27.9 | 28.3 | 28.9 | 31.4 | 31.4 | 30.9 |
| 6 | male | 17.3 | 14.2 | 21 | 22.5 | 23.4 | 23.9 | 24.2 | 26 | 25.9 | 26 |
| 7 | male | 17.9 | 19.7 | 24.4 | 25.1 | 25.5 | 26.2 | 27.1 | 28.3 | 29.1 | 29.1 |
| | | 17.44286 | 20.12857 | 24.41429 | 25.58571 | 26.1 | 26.77143 | 27.58571 | 28.84286 | 29.11429 | 29.35714 |
| | | | | | | | | | | | |
| ND Dmt2-/- F1 | | | | | | | | | | | |
| 1 | male | 17.3 | 19.2 | 21.8 | 22.6 | 23.5 | 24.3 | 25.5 | 26.9 | 27.3 | 27.8 |
| 2 | male | 18.9 | 20.2 | 22.1 | 22.8 | 23.7 | 24.6 | 25.9 | 27.3 | 27.5 | 27.8 |
| 3 | male | 15.4 | 17.1 | 20.7 | 22.1 | 23 | 23.9 | 25 | 26.1 | 26.8 | 27.1 |
| 4 | male | 19.3 | 20.8 | 22.3 | 22.9 | 23.7 | 24.9 | 26 | 27.4 | 27 | 26.8 |
| 5 | male | 17.2 | 18.6 | 20.2 | 21.5 | 22 | 23.2 | 24.1 | 24.9 | 25.8 | 26 |
| | | 17.62 | 19.18 | 21.42 | 22.38 | 23.18 | 24.18 | 25.3 | 26.52 | 26.88 | 27.1 |

## Slide 63
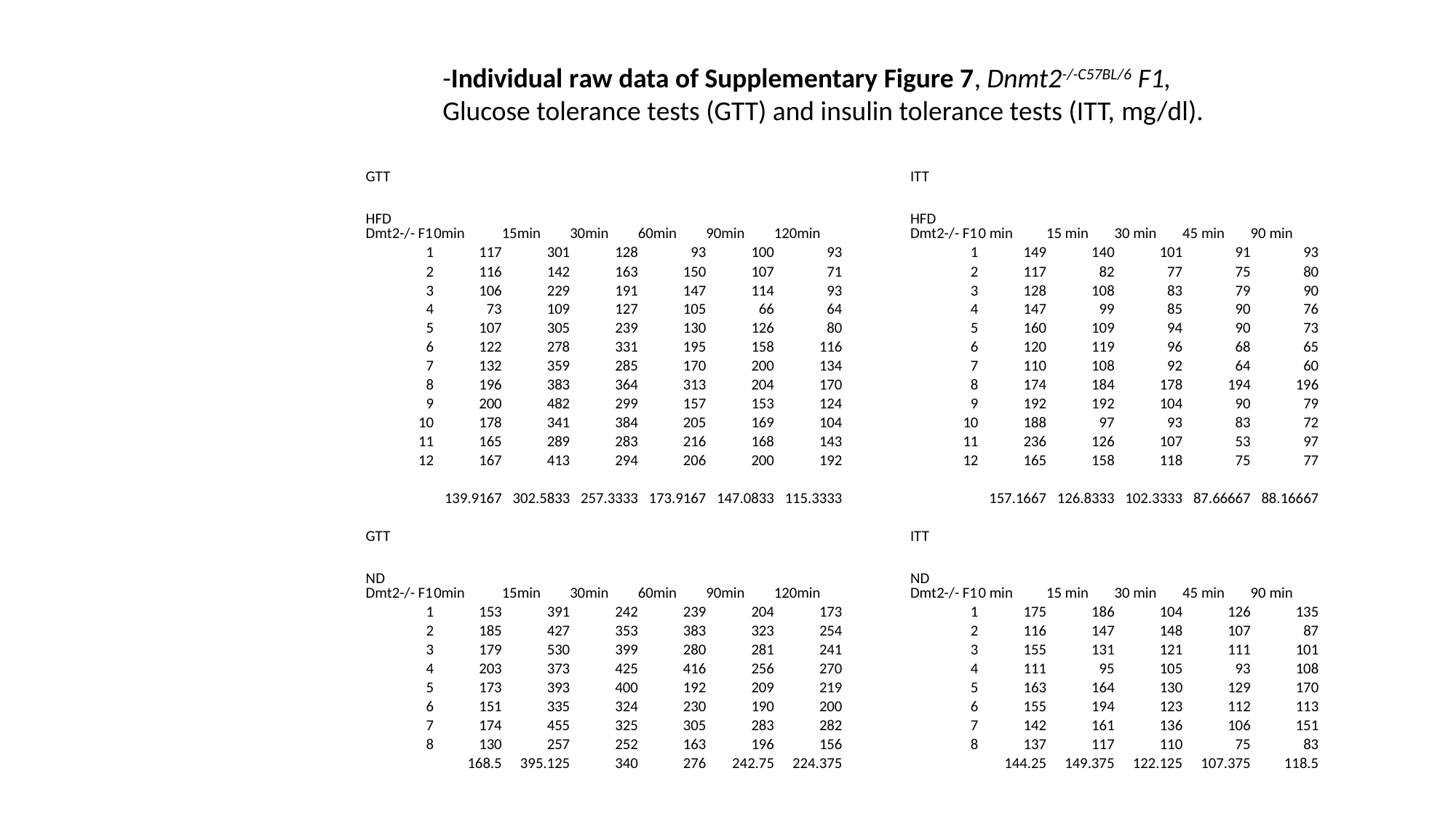

-Individual raw data of Supplementary Figure 7, Dnmt2-/-C57BL/6 F1,
Glucose tolerance tests (GTT) and insulin tolerance tests (ITT, mg/dl).
| GTT | | | | | | | | ITT | | | | | |
| --- | --- | --- | --- | --- | --- | --- | --- | --- | --- | --- | --- | --- | --- |
| HFD Dmt2-/- F1 | 0min | 15min | 30min | 60min | 90min | 120min | | HFD Dmt2-/- F1 | 0 min | 15 min | 30 min | 45 min | 90 min |
| 1 | 117 | 301 | 128 | 93 | 100 | 93 | | 1 | 149 | 140 | 101 | 91 | 93 |
| 2 | 116 | 142 | 163 | 150 | 107 | 71 | | 2 | 117 | 82 | 77 | 75 | 80 |
| 3 | 106 | 229 | 191 | 147 | 114 | 93 | | 3 | 128 | 108 | 83 | 79 | 90 |
| 4 | 73 | 109 | 127 | 105 | 66 | 64 | | 4 | 147 | 99 | 85 | 90 | 76 |
| 5 | 107 | 305 | 239 | 130 | 126 | 80 | | 5 | 160 | 109 | 94 | 90 | 73 |
| 6 | 122 | 278 | 331 | 195 | 158 | 116 | | 6 | 120 | 119 | 96 | 68 | 65 |
| 7 | 132 | 359 | 285 | 170 | 200 | 134 | | 7 | 110 | 108 | 92 | 64 | 60 |
| 8 | 196 | 383 | 364 | 313 | 204 | 170 | | 8 | 174 | 184 | 178 | 194 | 196 |
| 9 | 200 | 482 | 299 | 157 | 153 | 124 | | 9 | 192 | 192 | 104 | 90 | 79 |
| 10 | 178 | 341 | 384 | 205 | 169 | 104 | | 10 | 188 | 97 | 93 | 83 | 72 |
| 11 | 165 | 289 | 283 | 216 | 168 | 143 | | 11 | 236 | 126 | 107 | 53 | 97 |
| 12 | 167 | 413 | 294 | 206 | 200 | 192 | | 12 | 165 | 158 | 118 | 75 | 77 |
| | 139.9167 | 302.5833 | 257.3333 | 173.9167 | 147.0833 | 115.3333 | | | 157.1667 | 126.8333 | 102.3333 | 87.66667 | 88.16667 |
| | | | | | | | | | | | | | |
| GTT | | | | | | | | ITT | | | | | |
| ND Dmt2-/- F1 | 0min | 15min | 30min | 60min | 90min | 120min | | ND Dmt2-/- F1 | 0 min | 15 min | 30 min | 45 min | 90 min |
| 1 | 153 | 391 | 242 | 239 | 204 | 173 | | 1 | 175 | 186 | 104 | 126 | 135 |
| 2 | 185 | 427 | 353 | 383 | 323 | 254 | | 2 | 116 | 147 | 148 | 107 | 87 |
| 3 | 179 | 530 | 399 | 280 | 281 | 241 | | 3 | 155 | 131 | 121 | 111 | 101 |
| 4 | 203 | 373 | 425 | 416 | 256 | 270 | | 4 | 111 | 95 | 105 | 93 | 108 |
| 5 | 173 | 393 | 400 | 192 | 209 | 219 | | 5 | 163 | 164 | 130 | 129 | 170 |
| 6 | 151 | 335 | 324 | 230 | 190 | 200 | | 6 | 155 | 194 | 123 | 112 | 113 |
| 7 | 174 | 455 | 325 | 305 | 283 | 282 | | 7 | 142 | 161 | 136 | 106 | 151 |
| 8 | 130 | 257 | 252 | 163 | 196 | 156 | | 8 | 137 | 117 | 110 | 75 | 83 |
| | 168.5 | 395.125 | 340 | 276 | 242.75 | 224.375 | | | 144.25 | 149.375 | 122.125 | 107.375 | 118.5 |

## Slide 64
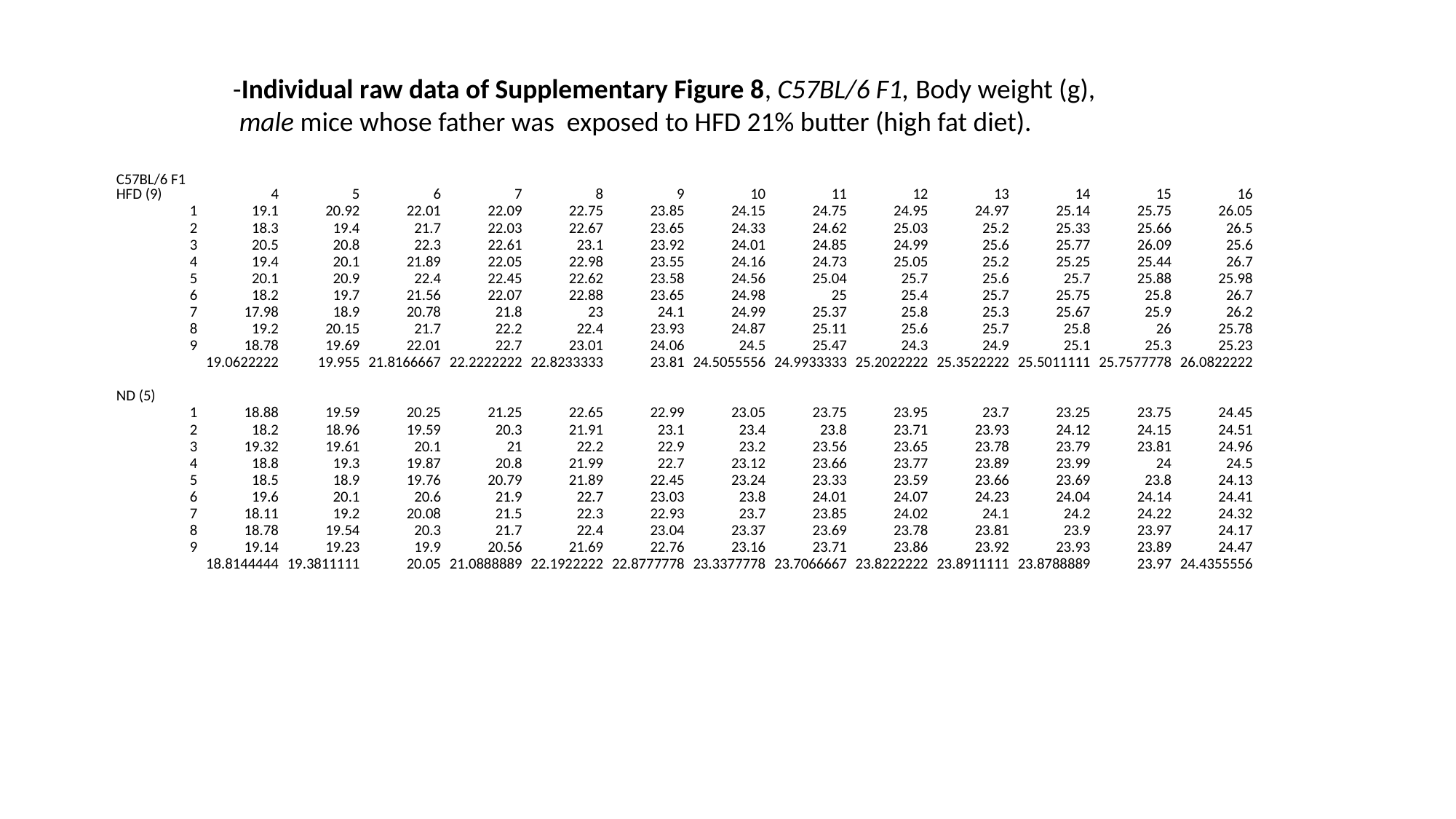

-Individual raw data of Supplementary Figure 8, C57BL/6 F1, Body weight (g),
 male mice whose father was exposed to HFD 21% butter (high fat diet).
| C57BL/6 F1 HFD (9) | 4 | 5 | 6 | 7 | 8 | 9 | 10 | 11 | 12 | 13 | 14 | 15 | 16 |
| --- | --- | --- | --- | --- | --- | --- | --- | --- | --- | --- | --- | --- | --- |
| 1 | 19.1 | 20.92 | 22.01 | 22.09 | 22.75 | 23.85 | 24.15 | 24.75 | 24.95 | 24.97 | 25.14 | 25.75 | 26.05 |
| 2 | 18.3 | 19.4 | 21.7 | 22.03 | 22.67 | 23.65 | 24.33 | 24.62 | 25.03 | 25.2 | 25.33 | 25.66 | 26.5 |
| 3 | 20.5 | 20.8 | 22.3 | 22.61 | 23.1 | 23.92 | 24.01 | 24.85 | 24.99 | 25.6 | 25.77 | 26.09 | 25.6 |
| 4 | 19.4 | 20.1 | 21.89 | 22.05 | 22.98 | 23.55 | 24.16 | 24.73 | 25.05 | 25.2 | 25.25 | 25.44 | 26.7 |
| 5 | 20.1 | 20.9 | 22.4 | 22.45 | 22.62 | 23.58 | 24.56 | 25.04 | 25.7 | 25.6 | 25.7 | 25.88 | 25.98 |
| 6 | 18.2 | 19.7 | 21.56 | 22.07 | 22.88 | 23.65 | 24.98 | 25 | 25.4 | 25.7 | 25.75 | 25.8 | 26.7 |
| 7 | 17.98 | 18.9 | 20.78 | 21.8 | 23 | 24.1 | 24.99 | 25.37 | 25.8 | 25.3 | 25.67 | 25.9 | 26.2 |
| 8 | 19.2 | 20.15 | 21.7 | 22.2 | 22.4 | 23.93 | 24.87 | 25.11 | 25.6 | 25.7 | 25.8 | 26 | 25.78 |
| 9 | 18.78 | 19.69 | 22.01 | 22.7 | 23.01 | 24.06 | 24.5 | 25.47 | 24.3 | 24.9 | 25.1 | 25.3 | 25.23 |
| | 19.0622222 | 19.955 | 21.8166667 | 22.2222222 | 22.8233333 | 23.81 | 24.5055556 | 24.9933333 | 25.2022222 | 25.3522222 | 25.5011111 | 25.7577778 | 26.0822222 |
| | | | | | | | | | | | | | |
| ND (5) | | | | | | | | | | | | | |
| 1 | 18.88 | 19.59 | 20.25 | 21.25 | 22.65 | 22.99 | 23.05 | 23.75 | 23.95 | 23.7 | 23.25 | 23.75 | 24.45 |
| 2 | 18.2 | 18.96 | 19.59 | 20.3 | 21.91 | 23.1 | 23.4 | 23.8 | 23.71 | 23.93 | 24.12 | 24.15 | 24.51 |
| 3 | 19.32 | 19.61 | 20.1 | 21 | 22.2 | 22.9 | 23.2 | 23.56 | 23.65 | 23.78 | 23.79 | 23.81 | 24.96 |
| 4 | 18.8 | 19.3 | 19.87 | 20.8 | 21.99 | 22.7 | 23.12 | 23.66 | 23.77 | 23.89 | 23.99 | 24 | 24.5 |
| 5 | 18.5 | 18.9 | 19.76 | 20.79 | 21.89 | 22.45 | 23.24 | 23.33 | 23.59 | 23.66 | 23.69 | 23.8 | 24.13 |
| 6 | 19.6 | 20.1 | 20.6 | 21.9 | 22.7 | 23.03 | 23.8 | 24.01 | 24.07 | 24.23 | 24.04 | 24.14 | 24.41 |
| 7 | 18.11 | 19.2 | 20.08 | 21.5 | 22.3 | 22.93 | 23.7 | 23.85 | 24.02 | 24.1 | 24.2 | 24.22 | 24.32 |
| 8 | 18.78 | 19.54 | 20.3 | 21.7 | 22.4 | 23.04 | 23.37 | 23.69 | 23.78 | 23.81 | 23.9 | 23.97 | 24.17 |
| 9 | 19.14 | 19.23 | 19.9 | 20.56 | 21.69 | 22.76 | 23.16 | 23.71 | 23.86 | 23.92 | 23.93 | 23.89 | 24.47 |
| | 18.8144444 | 19.3811111 | 20.05 | 21.0888889 | 22.1922222 | 22.8777778 | 23.3377778 | 23.7066667 | 23.8222222 | 23.8911111 | 23.8788889 | 23.97 | 24.4355556 |

## Slide 65
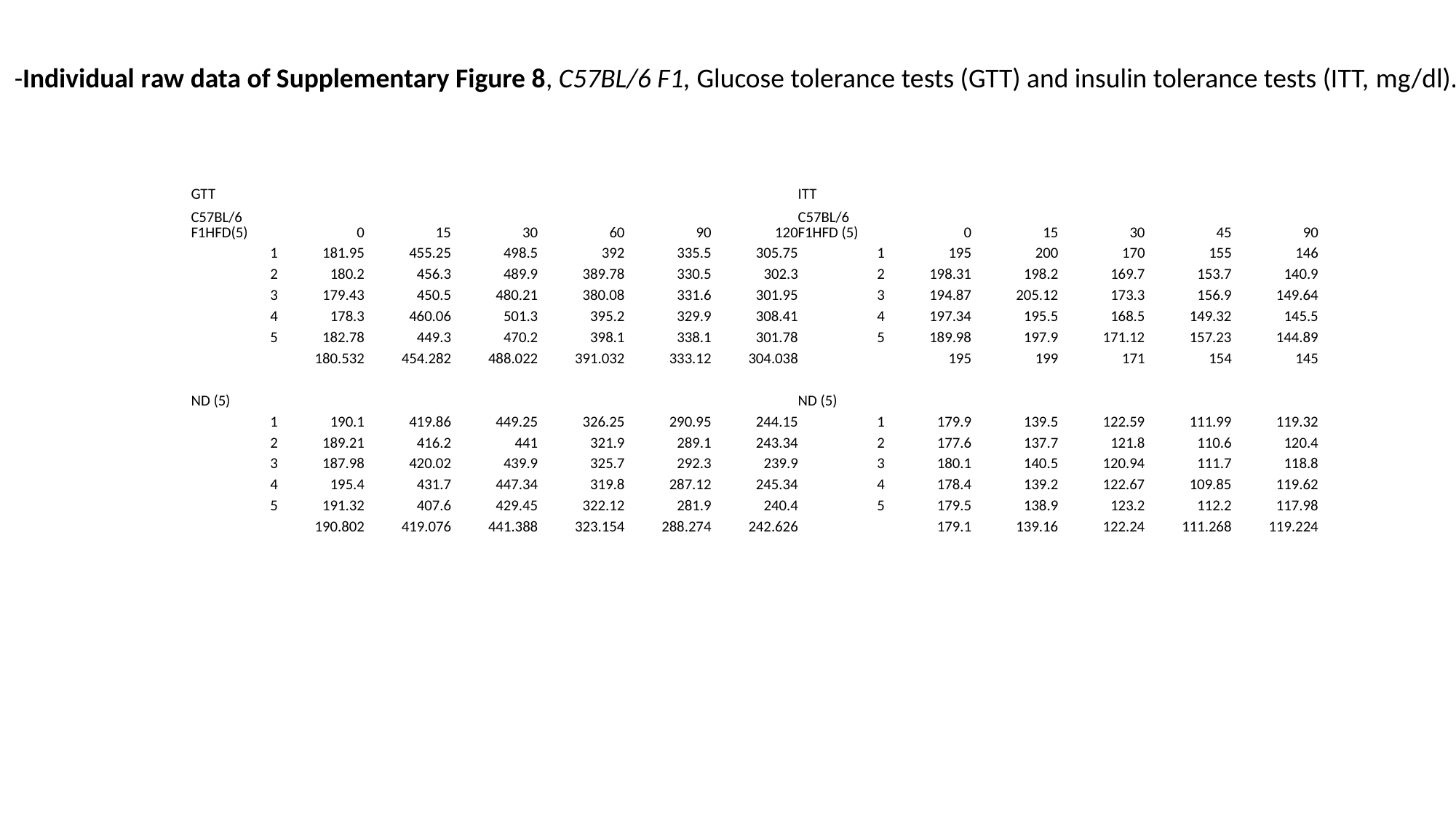

-Individual raw data of Supplementary Figure 8, C57BL/6 F1, Glucose tolerance tests (GTT) and insulin tolerance tests (ITT, mg/dl).
| GTT | | | | | | | ITT | | | | | |
| --- | --- | --- | --- | --- | --- | --- | --- | --- | --- | --- | --- | --- |
| C57BL/6 F1HFD(5) | 0 | 15 | 30 | 60 | 90 | 120 | C57BL/6 F1HFD (5) | 0 | 15 | 30 | 45 | 90 |
| 1 | 181.95 | 455.25 | 498.5 | 392 | 335.5 | 305.75 | 1 | 195 | 200 | 170 | 155 | 146 |
| 2 | 180.2 | 456.3 | 489.9 | 389.78 | 330.5 | 302.3 | 2 | 198.31 | 198.2 | 169.7 | 153.7 | 140.9 |
| 3 | 179.43 | 450.5 | 480.21 | 380.08 | 331.6 | 301.95 | 3 | 194.87 | 205.12 | 173.3 | 156.9 | 149.64 |
| 4 | 178.3 | 460.06 | 501.3 | 395.2 | 329.9 | 308.41 | 4 | 197.34 | 195.5 | 168.5 | 149.32 | 145.5 |
| 5 | 182.78 | 449.3 | 470.2 | 398.1 | 338.1 | 301.78 | 5 | 189.98 | 197.9 | 171.12 | 157.23 | 144.89 |
| | 180.532 | 454.282 | 488.022 | 391.032 | 333.12 | 304.038 | | 195 | 199 | 171 | 154 | 145 |
| | | | | | | | | | | | | |
| ND (5) | | | | | | | ND (5) | | | | | |
| 1 | 190.1 | 419.86 | 449.25 | 326.25 | 290.95 | 244.15 | 1 | 179.9 | 139.5 | 122.59 | 111.99 | 119.32 |
| 2 | 189.21 | 416.2 | 441 | 321.9 | 289.1 | 243.34 | 2 | 177.6 | 137.7 | 121.8 | 110.6 | 120.4 |
| 3 | 187.98 | 420.02 | 439.9 | 325.7 | 292.3 | 239.9 | 3 | 180.1 | 140.5 | 120.94 | 111.7 | 118.8 |
| 4 | 195.4 | 431.7 | 447.34 | 319.8 | 287.12 | 245.34 | 4 | 178.4 | 139.2 | 122.67 | 109.85 | 119.62 |
| 5 | 191.32 | 407.6 | 429.45 | 322.12 | 281.9 | 240.4 | 5 | 179.5 | 138.9 | 123.2 | 112.2 | 117.98 |
| | 190.802 | 419.076 | 441.388 | 323.154 | 288.274 | 242.626 | | 179.1 | 139.16 | 122.24 | 111.268 | 119.224 |

## Slide 66
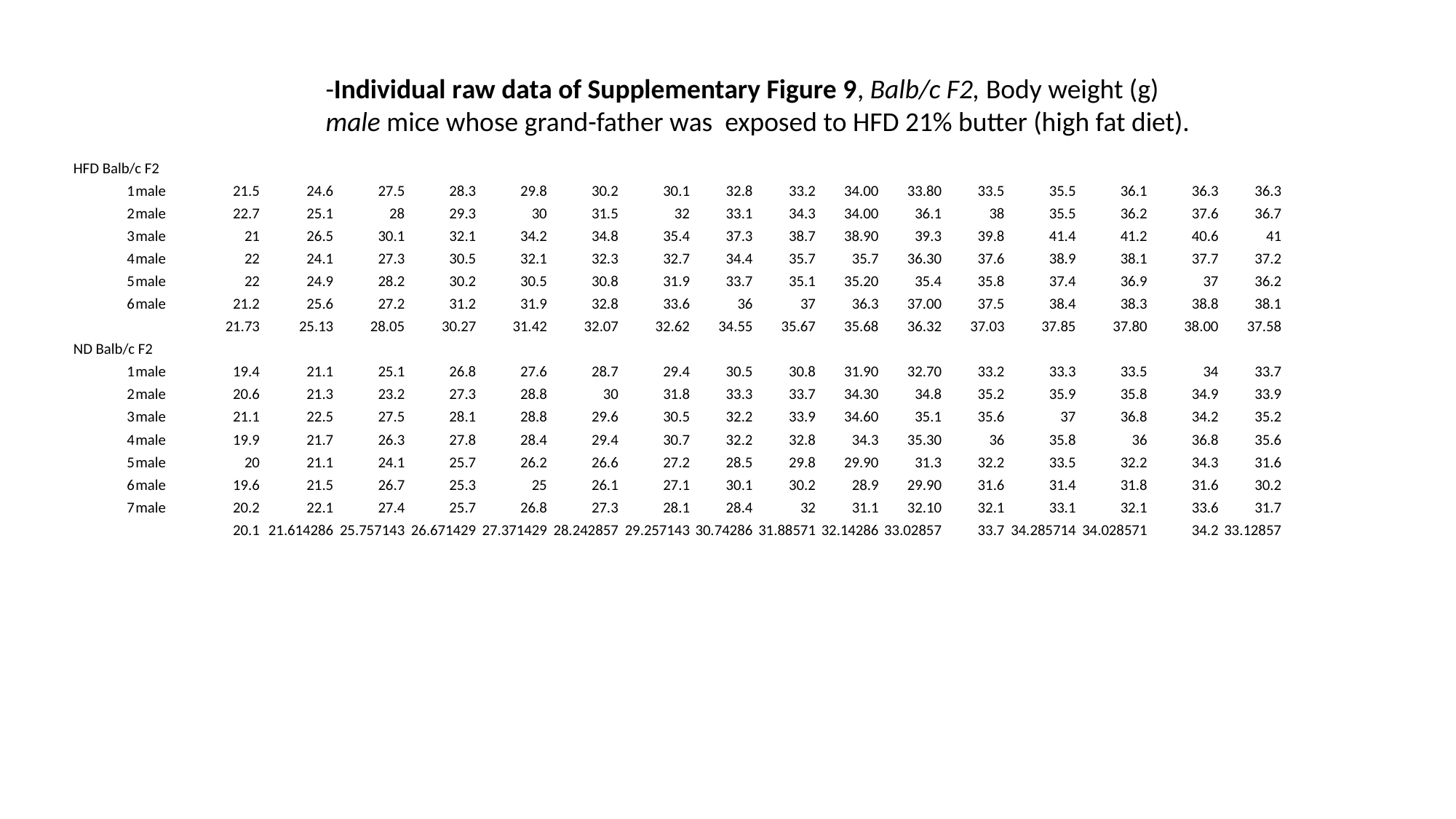

-Individual raw data of Supplementary Figure 9, Balb/c F2, Body weight (g)
male mice whose grand-father was exposed to HFD 21% butter (high fat diet).
| HFD Balb/c F2 | | | | | | | | | | | | | | | | | |
| --- | --- | --- | --- | --- | --- | --- | --- | --- | --- | --- | --- | --- | --- | --- | --- | --- | --- |
| 1 | male | 21.5 | 24.6 | 27.5 | 28.3 | 29.8 | 30.2 | 30.1 | 32.8 | 33.2 | 34.00 | 33.80 | 33.5 | 35.5 | 36.1 | 36.3 | 36.3 |
| 2 | male | 22.7 | 25.1 | 28 | 29.3 | 30 | 31.5 | 32 | 33.1 | 34.3 | 34.00 | 36.1 | 38 | 35.5 | 36.2 | 37.6 | 36.7 |
| 3 | male | 21 | 26.5 | 30.1 | 32.1 | 34.2 | 34.8 | 35.4 | 37.3 | 38.7 | 38.90 | 39.3 | 39.8 | 41.4 | 41.2 | 40.6 | 41 |
| 4 | male | 22 | 24.1 | 27.3 | 30.5 | 32.1 | 32.3 | 32.7 | 34.4 | 35.7 | 35.7 | 36.30 | 37.6 | 38.9 | 38.1 | 37.7 | 37.2 |
| 5 | male | 22 | 24.9 | 28.2 | 30.2 | 30.5 | 30.8 | 31.9 | 33.7 | 35.1 | 35.20 | 35.4 | 35.8 | 37.4 | 36.9 | 37 | 36.2 |
| 6 | male | 21.2 | 25.6 | 27.2 | 31.2 | 31.9 | 32.8 | 33.6 | 36 | 37 | 36.3 | 37.00 | 37.5 | 38.4 | 38.3 | 38.8 | 38.1 |
| | | 21.73 | 25.13 | 28.05 | 30.27 | 31.42 | 32.07 | 32.62 | 34.55 | 35.67 | 35.68 | 36.32 | 37.03 | 37.85 | 37.80 | 38.00 | 37.58 |
| ND Balb/c F2 | | | | | | | | | | | | | | | | | |
| 1 | male | 19.4 | 21.1 | 25.1 | 26.8 | 27.6 | 28.7 | 29.4 | 30.5 | 30.8 | 31.90 | 32.70 | 33.2 | 33.3 | 33.5 | 34 | 33.7 |
| 2 | male | 20.6 | 21.3 | 23.2 | 27.3 | 28.8 | 30 | 31.8 | 33.3 | 33.7 | 34.30 | 34.8 | 35.2 | 35.9 | 35.8 | 34.9 | 33.9 |
| 3 | male | 21.1 | 22.5 | 27.5 | 28.1 | 28.8 | 29.6 | 30.5 | 32.2 | 33.9 | 34.60 | 35.1 | 35.6 | 37 | 36.8 | 34.2 | 35.2 |
| 4 | male | 19.9 | 21.7 | 26.3 | 27.8 | 28.4 | 29.4 | 30.7 | 32.2 | 32.8 | 34.3 | 35.30 | 36 | 35.8 | 36 | 36.8 | 35.6 |
| 5 | male | 20 | 21.1 | 24.1 | 25.7 | 26.2 | 26.6 | 27.2 | 28.5 | 29.8 | 29.90 | 31.3 | 32.2 | 33.5 | 32.2 | 34.3 | 31.6 |
| 6 | male | 19.6 | 21.5 | 26.7 | 25.3 | 25 | 26.1 | 27.1 | 30.1 | 30.2 | 28.9 | 29.90 | 31.6 | 31.4 | 31.8 | 31.6 | 30.2 |
| 7 | male | 20.2 | 22.1 | 27.4 | 25.7 | 26.8 | 27.3 | 28.1 | 28.4 | 32 | 31.1 | 32.10 | 32.1 | 33.1 | 32.1 | 33.6 | 31.7 |
| | | 20.1 | 21.614286 | 25.757143 | 26.671429 | 27.371429 | 28.242857 | 29.257143 | 30.74286 | 31.88571 | 32.14286 | 33.02857 | 33.7 | 34.285714 | 34.028571 | 34.2 | 33.12857 |

## Slide 67
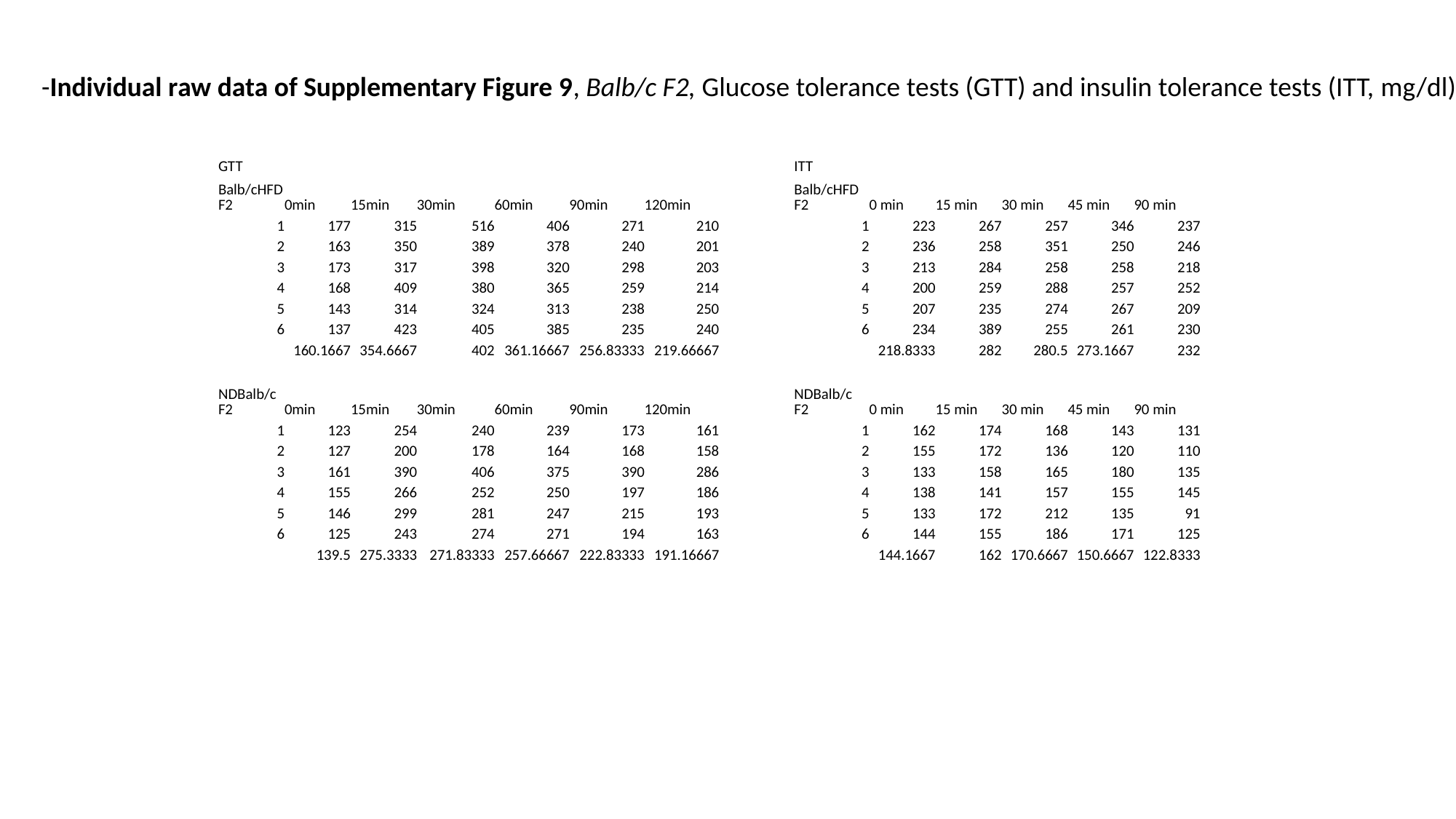

-Individual raw data of Supplementary Figure 9, Balb/c F2, Glucose tolerance tests (GTT) and insulin tolerance tests (ITT, mg/dl).
| GTT | | | | | | | | ITT | | | | | |
| --- | --- | --- | --- | --- | --- | --- | --- | --- | --- | --- | --- | --- | --- |
| Balb/cHFD F2 | 0min | 15min | 30min | 60min | 90min | 120min | | Balb/cHFD F2 | 0 min | 15 min | 30 min | 45 min | 90 min |
| 1 | 177 | 315 | 516 | 406 | 271 | 210 | | 1 | 223 | 267 | 257 | 346 | 237 |
| 2 | 163 | 350 | 389 | 378 | 240 | 201 | | 2 | 236 | 258 | 351 | 250 | 246 |
| 3 | 173 | 317 | 398 | 320 | 298 | 203 | | 3 | 213 | 284 | 258 | 258 | 218 |
| 4 | 168 | 409 | 380 | 365 | 259 | 214 | | 4 | 200 | 259 | 288 | 257 | 252 |
| 5 | 143 | 314 | 324 | 313 | 238 | 250 | | 5 | 207 | 235 | 274 | 267 | 209 |
| 6 | 137 | 423 | 405 | 385 | 235 | 240 | | 6 | 234 | 389 | 255 | 261 | 230 |
| | 160.1667 | 354.6667 | 402 | 361.16667 | 256.83333 | 219.66667 | | | 218.8333 | 282 | 280.5 | 273.1667 | 232 |
| | | | | | | | | | | | | | |
| NDBalb/c F2 | 0min | 15min | 30min | 60min | 90min | 120min | | NDBalb/c F2 | 0 min | 15 min | 30 min | 45 min | 90 min |
| 1 | 123 | 254 | 240 | 239 | 173 | 161 | | 1 | 162 | 174 | 168 | 143 | 131 |
| 2 | 127 | 200 | 178 | 164 | 168 | 158 | | 2 | 155 | 172 | 136 | 120 | 110 |
| 3 | 161 | 390 | 406 | 375 | 390 | 286 | | 3 | 133 | 158 | 165 | 180 | 135 |
| 4 | 155 | 266 | 252 | 250 | 197 | 186 | | 4 | 138 | 141 | 157 | 155 | 145 |
| 5 | 146 | 299 | 281 | 247 | 215 | 193 | | 5 | 133 | 172 | 212 | 135 | 91 |
| 6 | 125 | 243 | 274 | 271 | 194 | 163 | | 6 | 144 | 155 | 186 | 171 | 125 |
| | 139.5 | 275.3333 | 271.83333 | 257.66667 | 222.83333 | 191.16667 | | | 144.1667 | 162 | 170.6667 | 150.6667 | 122.8333 |

## Slide 68
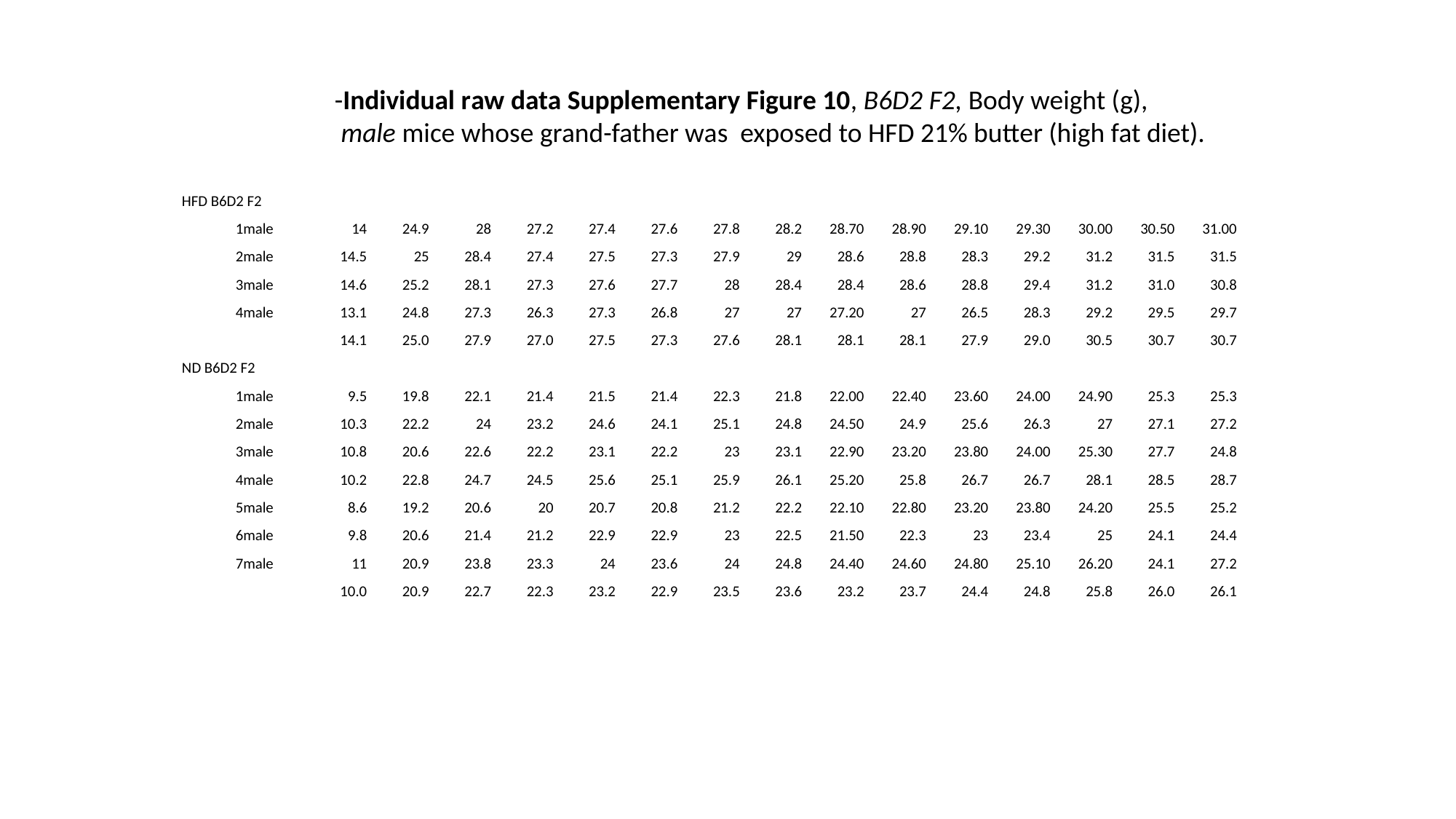

-Individual raw data Supplementary Figure 10, B6D2 F2, Body weight (g),
 male mice whose grand-father was exposed to HFD 21% butter (high fat diet).
| HFD B6D2 F2 | | | | | | | | | | | | | | | | |
| --- | --- | --- | --- | --- | --- | --- | --- | --- | --- | --- | --- | --- | --- | --- | --- | --- |
| 1 | male | 14 | 24.9 | 28 | 27.2 | 27.4 | 27.6 | 27.8 | 28.2 | 28.70 | 28.90 | 29.10 | 29.30 | 30.00 | 30.50 | 31.00 |
| 2 | male | 14.5 | 25 | 28.4 | 27.4 | 27.5 | 27.3 | 27.9 | 29 | 28.6 | 28.8 | 28.3 | 29.2 | 31.2 | 31.5 | 31.5 |
| 3 | male | 14.6 | 25.2 | 28.1 | 27.3 | 27.6 | 27.7 | 28 | 28.4 | 28.4 | 28.6 | 28.8 | 29.4 | 31.2 | 31.0 | 30.8 |
| 4 | male | 13.1 | 24.8 | 27.3 | 26.3 | 27.3 | 26.8 | 27 | 27 | 27.20 | 27 | 26.5 | 28.3 | 29.2 | 29.5 | 29.7 |
| | | 14.1 | 25.0 | 27.9 | 27.0 | 27.5 | 27.3 | 27.6 | 28.1 | 28.1 | 28.1 | 27.9 | 29.0 | 30.5 | 30.7 | 30.7 |
| ND B6D2 F2 | | | | | | | | | | | | | | | | |
| 1 | male | 9.5 | 19.8 | 22.1 | 21.4 | 21.5 | 21.4 | 22.3 | 21.8 | 22.00 | 22.40 | 23.60 | 24.00 | 24.90 | 25.3 | 25.3 |
| 2 | male | 10.3 | 22.2 | 24 | 23.2 | 24.6 | 24.1 | 25.1 | 24.8 | 24.50 | 24.9 | 25.6 | 26.3 | 27 | 27.1 | 27.2 |
| 3 | male | 10.8 | 20.6 | 22.6 | 22.2 | 23.1 | 22.2 | 23 | 23.1 | 22.90 | 23.20 | 23.80 | 24.00 | 25.30 | 27.7 | 24.8 |
| 4 | male | 10.2 | 22.8 | 24.7 | 24.5 | 25.6 | 25.1 | 25.9 | 26.1 | 25.20 | 25.8 | 26.7 | 26.7 | 28.1 | 28.5 | 28.7 |
| 5 | male | 8.6 | 19.2 | 20.6 | 20 | 20.7 | 20.8 | 21.2 | 22.2 | 22.10 | 22.80 | 23.20 | 23.80 | 24.20 | 25.5 | 25.2 |
| 6 | male | 9.8 | 20.6 | 21.4 | 21.2 | 22.9 | 22.9 | 23 | 22.5 | 21.50 | 22.3 | 23 | 23.4 | 25 | 24.1 | 24.4 |
| 7 | male | 11 | 20.9 | 23.8 | 23.3 | 24 | 23.6 | 24 | 24.8 | 24.40 | 24.60 | 24.80 | 25.10 | 26.20 | 24.1 | 27.2 |
| | | 10.0 | 20.9 | 22.7 | 22.3 | 23.2 | 22.9 | 23.5 | 23.6 | 23.2 | 23.7 | 24.4 | 24.8 | 25.8 | 26.0 | 26.1 |

## Slide 69
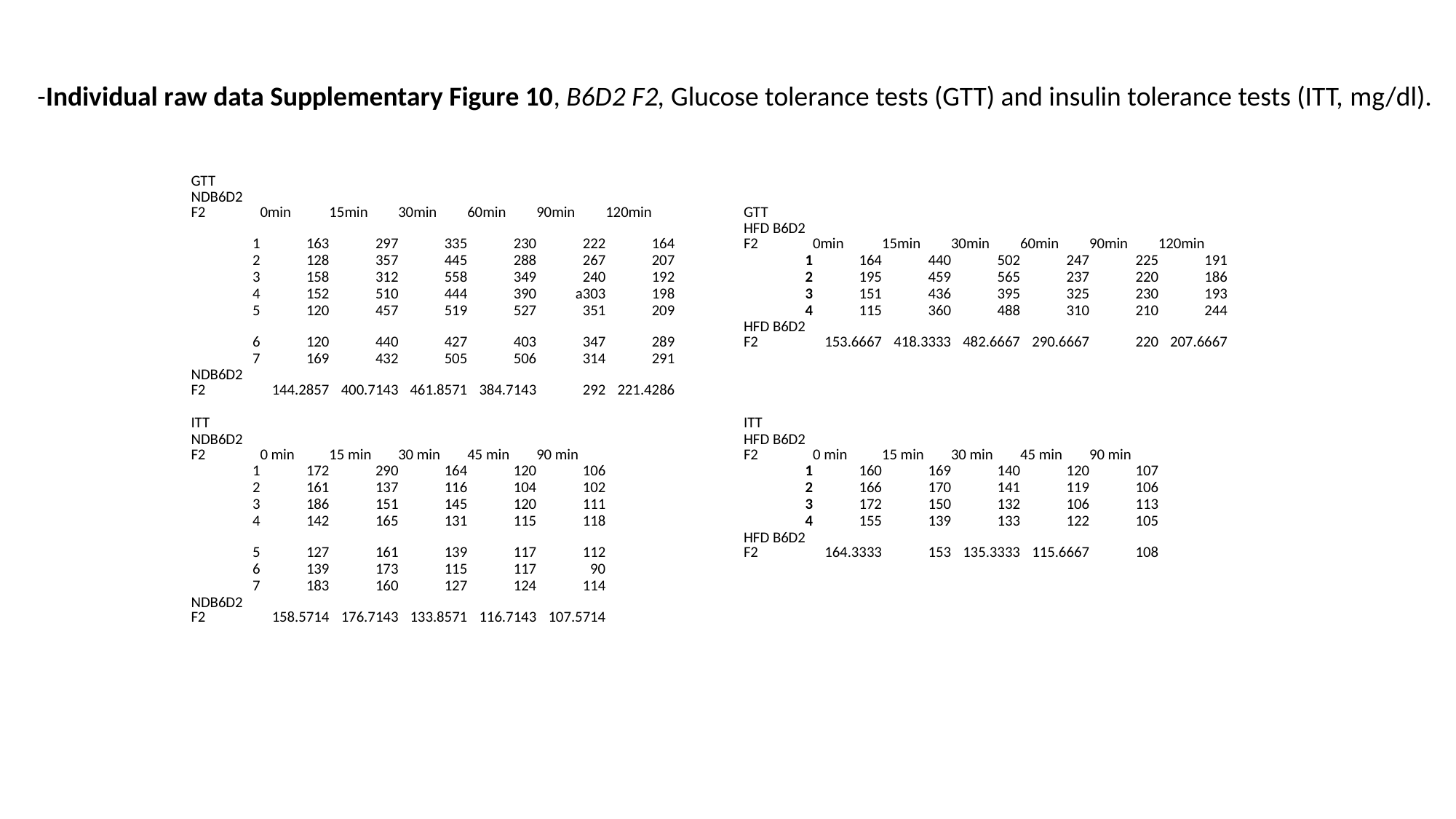

-Individual raw data Supplementary Figure 10, B6D2 F2, Glucose tolerance tests (GTT) and insulin tolerance tests (ITT, mg/dl).
| GTT | | | | | | | | | | | | | | |
| --- | --- | --- | --- | --- | --- | --- | --- | --- | --- | --- | --- | --- | --- | --- |
| NDB6D2 F2 | 0min | 15min | 30min | 60min | 90min | 120min | | GTT | | | | | | |
| 1 | 163 | 297 | 335 | 230 | 222 | 164 | | HFD B6D2 F2 | 0min | 15min | 30min | 60min | 90min | 120min |
| 2 | 128 | 357 | 445 | 288 | 267 | 207 | | 1 | 164 | 440 | 502 | 247 | 225 | 191 |
| 3 | 158 | 312 | 558 | 349 | 240 | 192 | | 2 | 195 | 459 | 565 | 237 | 220 | 186 |
| 4 | 152 | 510 | 444 | 390 | a303 | 198 | | 3 | 151 | 436 | 395 | 325 | 230 | 193 |
| 5 | 120 | 457 | 519 | 527 | 351 | 209 | | 4 | 115 | 360 | 488 | 310 | 210 | 244 |
| 6 | 120 | 440 | 427 | 403 | 347 | 289 | | HFD B6D2 F2 | 153.6667 | 418.3333 | 482.6667 | 290.6667 | 220 | 207.6667 |
| 7 | 169 | 432 | 505 | 506 | 314 | 291 | | | | | | | | |
| NDB6D2 F2 | 144.2857 | 400.7143 | 461.8571 | 384.7143 | 292 | 221.4286 | | | | | | | | |
| | | | | | | | | | | | | | | |
| ITT | | | | | | | | ITT | | | | | | |
| NDB6D2 F2 | 0 min | 15 min | 30 min | 45 min | 90 min | | | HFD B6D2 F2 | 0 min | 15 min | 30 min | 45 min | 90 min | |
| 1 | 172 | 290 | 164 | 120 | 106 | | | 1 | 160 | 169 | 140 | 120 | 107 | |
| 2 | 161 | 137 | 116 | 104 | 102 | | | 2 | 166 | 170 | 141 | 119 | 106 | |
| 3 | 186 | 151 | 145 | 120 | 111 | | | 3 | 172 | 150 | 132 | 106 | 113 | |
| 4 | 142 | 165 | 131 | 115 | 118 | | | 4 | 155 | 139 | 133 | 122 | 105 | |
| 5 | 127 | 161 | 139 | 117 | 112 | | | HFD B6D2 F2 | 164.3333 | 153 | 135.3333 | 115.6667 | 108 | |
| 6 | 139 | 173 | 115 | 117 | 90 | | | | | | | | | |
| 7 | 183 | 160 | 127 | 124 | 114 | | | | | | | | | |
| NDB6D2 F2 | 158.5714 | 176.7143 | 133.8571 | 116.7143 | 107.5714 | | | | | | | | | |

## Slide 70
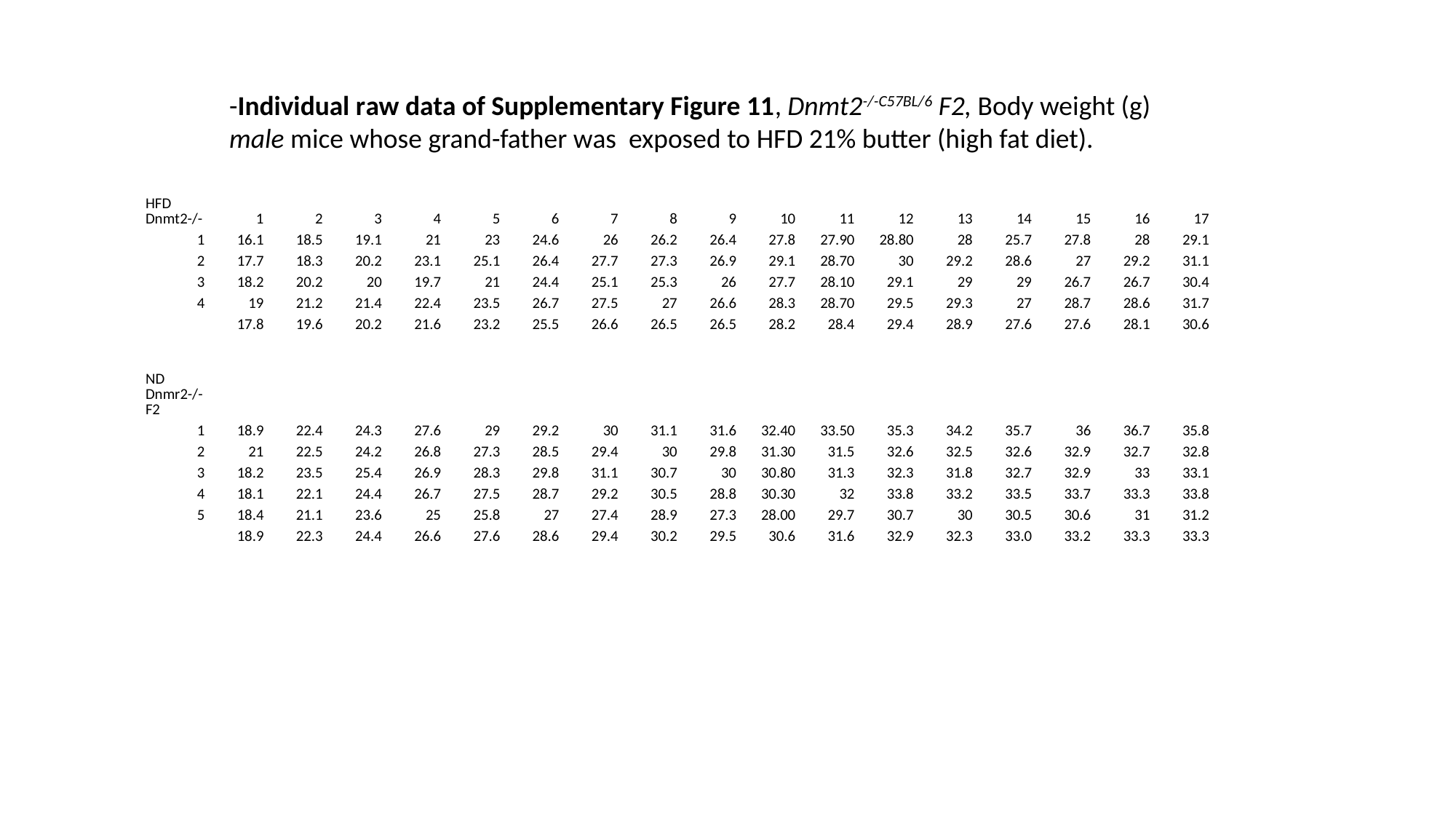

-Individual raw data of Supplementary Figure 11, Dnmt2-/-C57BL/6 F2, Body weight (g)
male mice whose grand-father was exposed to HFD 21% butter (high fat diet).
| HFD Dnmt2-/- | 1 | 2 | 3 | 4 | 5 | 6 | 7 | 8 | 9 | 10 | 11 | 12 | 13 | 14 | 15 | 16 | 17 |
| --- | --- | --- | --- | --- | --- | --- | --- | --- | --- | --- | --- | --- | --- | --- | --- | --- | --- |
| 1 | 16.1 | 18.5 | 19.1 | 21 | 23 | 24.6 | 26 | 26.2 | 26.4 | 27.8 | 27.90 | 28.80 | 28 | 25.7 | 27.8 | 28 | 29.1 |
| 2 | 17.7 | 18.3 | 20.2 | 23.1 | 25.1 | 26.4 | 27.7 | 27.3 | 26.9 | 29.1 | 28.70 | 30 | 29.2 | 28.6 | 27 | 29.2 | 31.1 |
| 3 | 18.2 | 20.2 | 20 | 19.7 | 21 | 24.4 | 25.1 | 25.3 | 26 | 27.7 | 28.10 | 29.1 | 29 | 29 | 26.7 | 26.7 | 30.4 |
| 4 | 19 | 21.2 | 21.4 | 22.4 | 23.5 | 26.7 | 27.5 | 27 | 26.6 | 28.3 | 28.70 | 29.5 | 29.3 | 27 | 28.7 | 28.6 | 31.7 |
| | 17.8 | 19.6 | 20.2 | 21.6 | 23.2 | 25.5 | 26.6 | 26.5 | 26.5 | 28.2 | 28.4 | 29.4 | 28.9 | 27.6 | 27.6 | 28.1 | 30.6 |
| | | | | | | | | | | | | | | | | | |
| ND Dnmr2-/- F2 | | | | | | | | | | | | | | | | | |
| 1 | 18.9 | 22.4 | 24.3 | 27.6 | 29 | 29.2 | 30 | 31.1 | 31.6 | 32.40 | 33.50 | 35.3 | 34.2 | 35.7 | 36 | 36.7 | 35.8 |
| 2 | 21 | 22.5 | 24.2 | 26.8 | 27.3 | 28.5 | 29.4 | 30 | 29.8 | 31.30 | 31.5 | 32.6 | 32.5 | 32.6 | 32.9 | 32.7 | 32.8 |
| 3 | 18.2 | 23.5 | 25.4 | 26.9 | 28.3 | 29.8 | 31.1 | 30.7 | 30 | 30.80 | 31.3 | 32.3 | 31.8 | 32.7 | 32.9 | 33 | 33.1 |
| 4 | 18.1 | 22.1 | 24.4 | 26.7 | 27.5 | 28.7 | 29.2 | 30.5 | 28.8 | 30.30 | 32 | 33.8 | 33.2 | 33.5 | 33.7 | 33.3 | 33.8 |
| 5 | 18.4 | 21.1 | 23.6 | 25 | 25.8 | 27 | 27.4 | 28.9 | 27.3 | 28.00 | 29.7 | 30.7 | 30 | 30.5 | 30.6 | 31 | 31.2 |
| | 18.9 | 22.3 | 24.4 | 26.6 | 27.6 | 28.6 | 29.4 | 30.2 | 29.5 | 30.6 | 31.6 | 32.9 | 32.3 | 33.0 | 33.2 | 33.3 | 33.3 |

## Slide 71
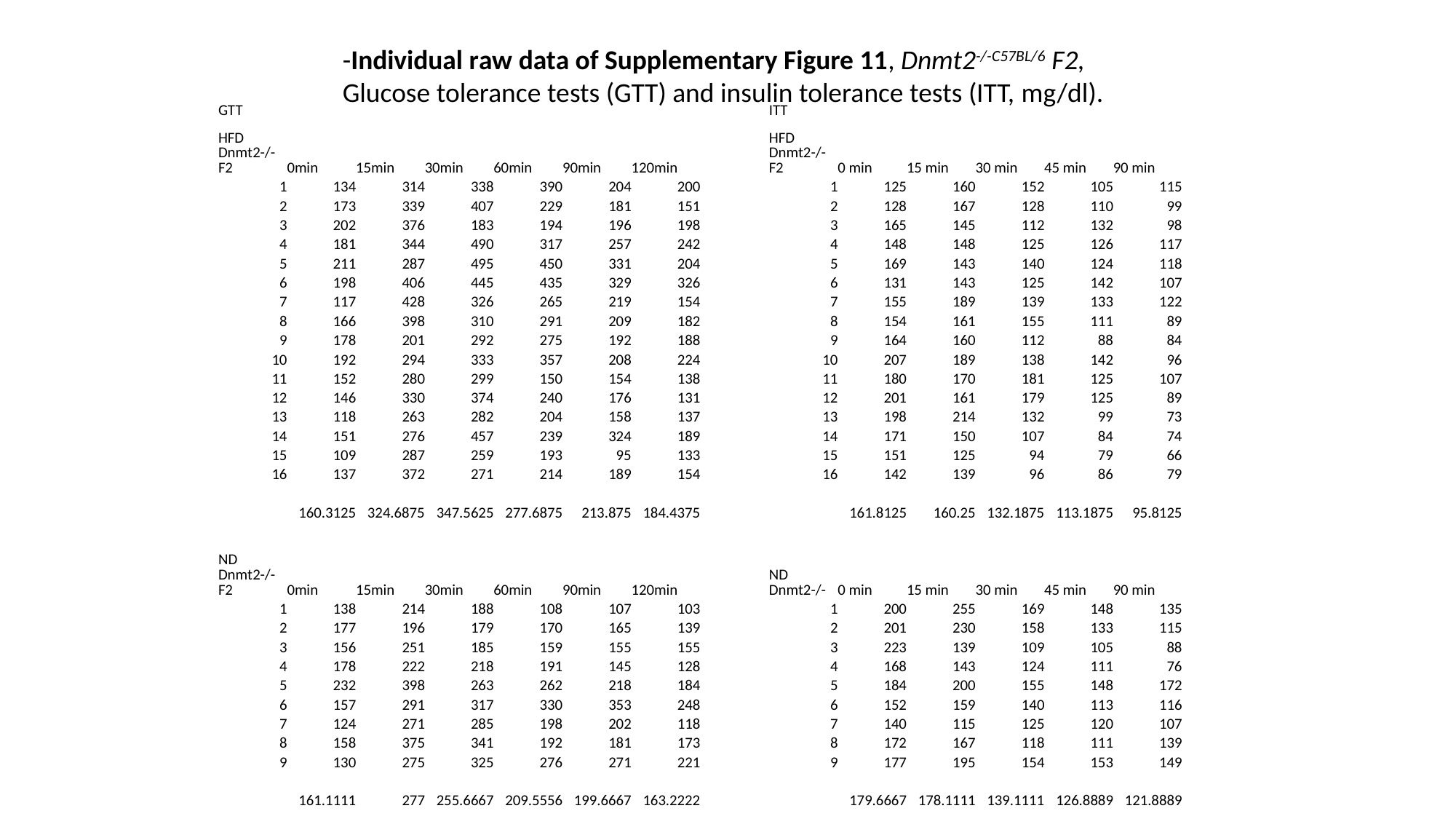

-Individual raw data of Supplementary Figure 11, Dnmt2-/-C57BL/6 F2,
Glucose tolerance tests (GTT) and insulin tolerance tests (ITT, mg/dl).
| GTT | | | | | | | | ITT | | | | | |
| --- | --- | --- | --- | --- | --- | --- | --- | --- | --- | --- | --- | --- | --- |
| HFD Dnmt2-/- F2 | 0min | 15min | 30min | 60min | 90min | 120min | | HFD Dnmt2-/- F2 | 0 min | 15 min | 30 min | 45 min | 90 min |
| 1 | 134 | 314 | 338 | 390 | 204 | 200 | | 1 | 125 | 160 | 152 | 105 | 115 |
| 2 | 173 | 339 | 407 | 229 | 181 | 151 | | 2 | 128 | 167 | 128 | 110 | 99 |
| 3 | 202 | 376 | 183 | 194 | 196 | 198 | | 3 | 165 | 145 | 112 | 132 | 98 |
| 4 | 181 | 344 | 490 | 317 | 257 | 242 | | 4 | 148 | 148 | 125 | 126 | 117 |
| 5 | 211 | 287 | 495 | 450 | 331 | 204 | | 5 | 169 | 143 | 140 | 124 | 118 |
| 6 | 198 | 406 | 445 | 435 | 329 | 326 | | 6 | 131 | 143 | 125 | 142 | 107 |
| 7 | 117 | 428 | 326 | 265 | 219 | 154 | | 7 | 155 | 189 | 139 | 133 | 122 |
| 8 | 166 | 398 | 310 | 291 | 209 | 182 | | 8 | 154 | 161 | 155 | 111 | 89 |
| 9 | 178 | 201 | 292 | 275 | 192 | 188 | | 9 | 164 | 160 | 112 | 88 | 84 |
| 10 | 192 | 294 | 333 | 357 | 208 | 224 | | 10 | 207 | 189 | 138 | 142 | 96 |
| 11 | 152 | 280 | 299 | 150 | 154 | 138 | | 11 | 180 | 170 | 181 | 125 | 107 |
| 12 | 146 | 330 | 374 | 240 | 176 | 131 | | 12 | 201 | 161 | 179 | 125 | 89 |
| 13 | 118 | 263 | 282 | 204 | 158 | 137 | | 13 | 198 | 214 | 132 | 99 | 73 |
| 14 | 151 | 276 | 457 | 239 | 324 | 189 | | 14 | 171 | 150 | 107 | 84 | 74 |
| 15 | 109 | 287 | 259 | 193 | 95 | 133 | | 15 | 151 | 125 | 94 | 79 | 66 |
| 16 | 137 | 372 | 271 | 214 | 189 | 154 | | 16 | 142 | 139 | 96 | 86 | 79 |
| | 160.3125 | 324.6875 | 347.5625 | 277.6875 | 213.875 | 184.4375 | | | 161.8125 | 160.25 | 132.1875 | 113.1875 | 95.8125 |
| | | | | | | | | | | | | | |
| ND Dnmt2-/- F2 | 0min | 15min | 30min | 60min | 90min | 120min | | ND Dnmt2-/- | 0 min | 15 min | 30 min | 45 min | 90 min |
| 1 | 138 | 214 | 188 | 108 | 107 | 103 | | 1 | 200 | 255 | 169 | 148 | 135 |
| 2 | 177 | 196 | 179 | 170 | 165 | 139 | | 2 | 201 | 230 | 158 | 133 | 115 |
| 3 | 156 | 251 | 185 | 159 | 155 | 155 | | 3 | 223 | 139 | 109 | 105 | 88 |
| 4 | 178 | 222 | 218 | 191 | 145 | 128 | | 4 | 168 | 143 | 124 | 111 | 76 |
| 5 | 232 | 398 | 263 | 262 | 218 | 184 | | 5 | 184 | 200 | 155 | 148 | 172 |
| 6 | 157 | 291 | 317 | 330 | 353 | 248 | | 6 | 152 | 159 | 140 | 113 | 116 |
| 7 | 124 | 271 | 285 | 198 | 202 | 118 | | 7 | 140 | 115 | 125 | 120 | 107 |
| 8 | 158 | 375 | 341 | 192 | 181 | 173 | | 8 | 172 | 167 | 118 | 111 | 139 |
| 9 | 130 | 275 | 325 | 276 | 271 | 221 | | 9 | 177 | 195 | 154 | 153 | 149 |
| | 161.1111 | 277 | 255.6667 | 209.5556 | 199.6667 | 163.2222 | | | 179.6667 | 178.1111 | 139.1111 | 126.8889 | 121.8889 |

## Slide 72
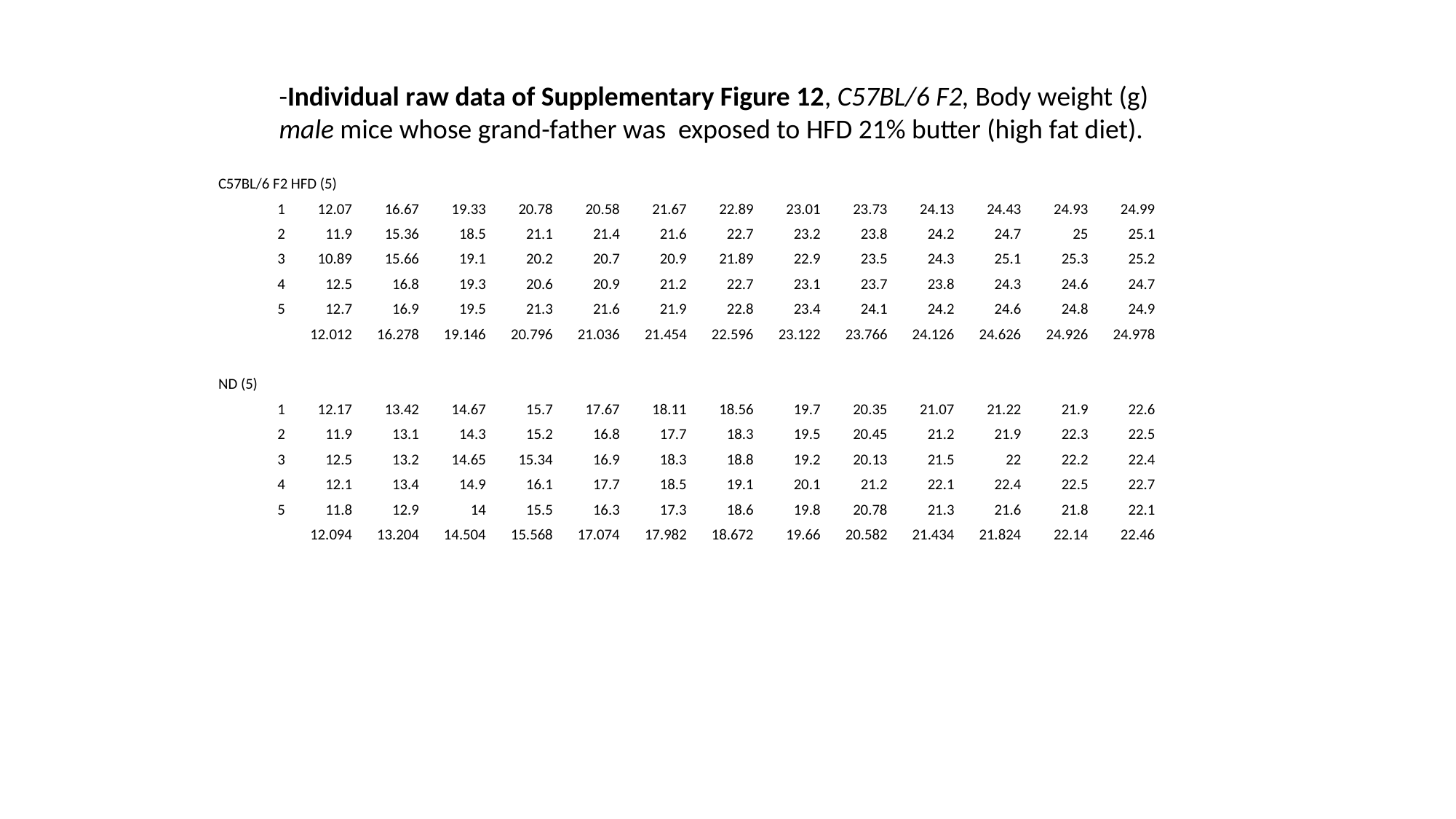

-Individual raw data of Supplementary Figure 12, C57BL/6 F2, Body weight (g)
male mice whose grand-father was exposed to HFD 21% butter (high fat diet).
| C57BL/6 F2 HFD (5) | | | | | | | | | | | | | |
| --- | --- | --- | --- | --- | --- | --- | --- | --- | --- | --- | --- | --- | --- |
| 1 | 12.07 | 16.67 | 19.33 | 20.78 | 20.58 | 21.67 | 22.89 | 23.01 | 23.73 | 24.13 | 24.43 | 24.93 | 24.99 |
| 2 | 11.9 | 15.36 | 18.5 | 21.1 | 21.4 | 21.6 | 22.7 | 23.2 | 23.8 | 24.2 | 24.7 | 25 | 25.1 |
| 3 | 10.89 | 15.66 | 19.1 | 20.2 | 20.7 | 20.9 | 21.89 | 22.9 | 23.5 | 24.3 | 25.1 | 25.3 | 25.2 |
| 4 | 12.5 | 16.8 | 19.3 | 20.6 | 20.9 | 21.2 | 22.7 | 23.1 | 23.7 | 23.8 | 24.3 | 24.6 | 24.7 |
| 5 | 12.7 | 16.9 | 19.5 | 21.3 | 21.6 | 21.9 | 22.8 | 23.4 | 24.1 | 24.2 | 24.6 | 24.8 | 24.9 |
| | 12.012 | 16.278 | 19.146 | 20.796 | 21.036 | 21.454 | 22.596 | 23.122 | 23.766 | 24.126 | 24.626 | 24.926 | 24.978 |
| | | | | | | | | | | | | | |
| ND (5) | | | | | | | | | | | | | |
| 1 | 12.17 | 13.42 | 14.67 | 15.7 | 17.67 | 18.11 | 18.56 | 19.7 | 20.35 | 21.07 | 21.22 | 21.9 | 22.6 |
| 2 | 11.9 | 13.1 | 14.3 | 15.2 | 16.8 | 17.7 | 18.3 | 19.5 | 20.45 | 21.2 | 21.9 | 22.3 | 22.5 |
| 3 | 12.5 | 13.2 | 14.65 | 15.34 | 16.9 | 18.3 | 18.8 | 19.2 | 20.13 | 21.5 | 22 | 22.2 | 22.4 |
| 4 | 12.1 | 13.4 | 14.9 | 16.1 | 17.7 | 18.5 | 19.1 | 20.1 | 21.2 | 22.1 | 22.4 | 22.5 | 22.7 |
| 5 | 11.8 | 12.9 | 14 | 15.5 | 16.3 | 17.3 | 18.6 | 19.8 | 20.78 | 21.3 | 21.6 | 21.8 | 22.1 |
| | 12.094 | 13.204 | 14.504 | 15.568 | 17.074 | 17.982 | 18.672 | 19.66 | 20.582 | 21.434 | 21.824 | 22.14 | 22.46 |

## Slide 73
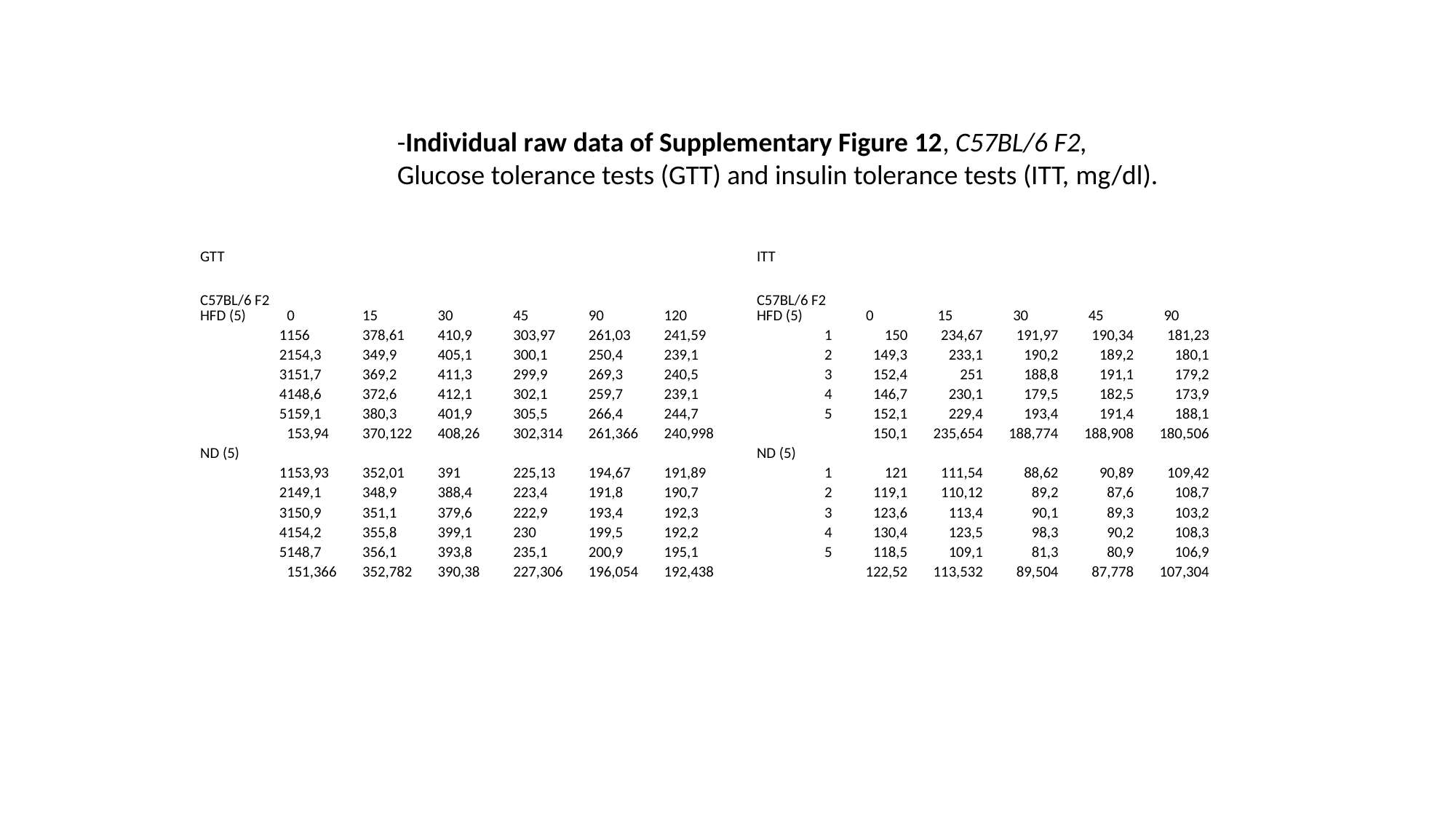

-Individual raw data of Supplementary Figure 12, C57BL/6 F2,
Glucose tolerance tests (GTT) and insulin tolerance tests (ITT, mg/dl).
| GTT | | | | | | | ITT | | | | | |
| --- | --- | --- | --- | --- | --- | --- | --- | --- | --- | --- | --- | --- |
| C57BL/6 F2 HFD (5) | 0 | 15 | 30 | 45 | 90 | 120 | C57BL/6 F2 HFD (5) | 0 | 15 | 30 | 45 | 90 |
| 1 | 156 | 378,61 | 410,9 | 303,97 | 261,03 | 241,59 | 1 | 150 | 234,67 | 191,97 | 190,34 | 181,23 |
| 2 | 154,3 | 349,9 | 405,1 | 300,1 | 250,4 | 239,1 | 2 | 149,3 | 233,1 | 190,2 | 189,2 | 180,1 |
| 3 | 151,7 | 369,2 | 411,3 | 299,9 | 269,3 | 240,5 | 3 | 152,4 | 251 | 188,8 | 191,1 | 179,2 |
| 4 | 148,6 | 372,6 | 412,1 | 302,1 | 259,7 | 239,1 | 4 | 146,7 | 230,1 | 179,5 | 182,5 | 173,9 |
| 5 | 159,1 | 380,3 | 401,9 | 305,5 | 266,4 | 244,7 | 5 | 152,1 | 229,4 | 193,4 | 191,4 | 188,1 |
| | 153,94 | 370,122 | 408,26 | 302,314 | 261,366 | 240,998 | | 150,1 | 235,654 | 188,774 | 188,908 | 180,506 |
| ND (5) | | | | | | | ND (5) | | | | | |
| 1 | 153,93 | 352,01 | 391 | 225,13 | 194,67 | 191,89 | 1 | 121 | 111,54 | 88,62 | 90,89 | 109,42 |
| 2 | 149,1 | 348,9 | 388,4 | 223,4 | 191,8 | 190,7 | 2 | 119,1 | 110,12 | 89,2 | 87,6 | 108,7 |
| 3 | 150,9 | 351,1 | 379,6 | 222,9 | 193,4 | 192,3 | 3 | 123,6 | 113,4 | 90,1 | 89,3 | 103,2 |
| 4 | 154,2 | 355,8 | 399,1 | 230 | 199,5 | 192,2 | 4 | 130,4 | 123,5 | 98,3 | 90,2 | 108,3 |
| 5 | 148,7 | 356,1 | 393,8 | 235,1 | 200,9 | 195,1 | 5 | 118,5 | 109,1 | 81,3 | 80,9 | 106,9 |
| | 151,366 | 352,782 | 390,38 | 227,306 | 196,054 | 192,438 | | 122,52 | 113,532 | 89,504 | 87,778 | 107,304 |
